# Integrative analyses identify modulators of response to neoadjuvant aromatase inhibitors in patients with early breast cancer

**DOI:** 10.1186/s13058-015-0532-0

**Published:** 2015-03-11

**Authors:** Elena López-Knowles, Paul M Wilkerson, Ricardo Ribas, Helen Anderson, Alan Mackay, Zara Ghazoui, Aradhana Rani, Peter Osin, Ash Nerurkar, Lorna Renshaw, Alexey Larionov, William R Miller, J Michael Dixon, Jorge S Reis-Filho, Anita K Dunbier, Lesley-Ann Martin, Mitch Dowsett

**Affiliations:** Royal Marsden Hospital, London, UK; Breakthrough Breast Cancer Research Centre, Institute of Cancer Research, London, UK; University of Edinburgh, Edinburgh, UK; Current affiliation: AstraZeneca, Alderley Park, Macclesfield, SK10 4TG UK; Current affiliation: Academic Laboratory of Medical Genetics, School of Clinical Medicine, University of Cambridge, Cambridge, UK; Current affiliation: Department of Pathology, Memorial Sloan-Kettering Cancer Center, New York, NY 10065 USA; Current affiliation: Department of Biochemistry, University of Otago, Dunedin, New Zealand

## Abstract

**Introduction:**

Aromatase inhibitors (AIs) are a vital component of estrogen receptor positive (ER+) breast cancer treatment. *De novo* and acquired resistance, however, is common. The aims of this study were to relate patterns of copy number aberrations to molecular and proliferative response to AIs, to study differences in the patterns of copy number aberrations between breast cancer samples pre- and post-AI neoadjuvant therapy, and to identify putative biomarkers for resistance to neoadjuvant AI therapy using an integrative analysis approach.

**Methods:**

Samples from 84 patients derived from two neoadjuvant AI therapy trials were subjected to copy number profiling by microarray-based comparative genomic hybridisation (aCGH, n = 84), gene expression profiling (n = 47), matched pre- and post-AI aCGH (n = 19 pairs) and Ki67-based AI-response analysis (n = 39).

**Results:**

Integrative analysis of these datasets identified a set of nine genes that, when amplified, were associated with a poor response to AIs, and were significantly overexpressed when amplified, including *CHKA, LRP5* and *SAPS3*. Functional validation *in vitro*, using cell lines with and without amplification of these genes (SUM44, MDA-MB134-VI, T47D and MCF7) and a model of acquired AI-resistance (MCF7-LTED) identified *CHKA* as a gene that when amplified modulates estrogen receptor (ER)-driven proliferation, ER/estrogen response element (ERE) transactivation, expression of ER-regulated genes and phosphorylation of V-AKT murine thymoma viral oncogene homolog 1 (AKT1).

**Conclusions:**

These data provide a rationale for investigation of the role of *CHKA* in further models of *de novo* and acquired resistance to AIs, and provide proof of concept that integrative genomic analyses can identify biologically relevant modulators of AI response.

**Electronic supplementary material:**

The online version of this article (doi:10.1186/s13058-015-0532-0) contains supplementary material, which is available to authorized users.

## Introduction

Aromatase inhibitors (AIs), such as anastrozole or letrozole, block the synthesis of estrogen [1]. AIs are the standard of care for the treatment of estrogen receptor (ER)-positive breast cancer in postmenopausal women [2]. Estrogen deprivation has a rapid effect on transcriptional profiles, with substantial gene expression changes identified after 15 days of treatment [3,4]. The most frequently upregulated pathways are those associated with focal adhesion, actin cytoskeleton and inflammation, while the most frequently downregulated pathways are those related to proliferation, growth and ER transcription [5].

Acquired or *de novo* resistance to AIs is common [6], and multiple putative mechanisms of resistance to AI therapy have been proposed. These include intrinsic resistance of tumors to estrogen, aromatase-independent estrogenic hormones, signal transduction by non-endocrine pathways and selection of hormone-insensitive clones during AI therapy (reviewed by Miller *et al.* [7]). A number of potential biomarkers of resistance have been suggested, including overexpression of human epidermal growth factor receptor-2 (HER2), Cyclin E1, hypoxia-inducible factor (HIF)1α and p44/42 mitogen-activated protein kinase (MAPK) [8]. These biomarkers, however, still require validation in independent cohorts [7] or are unlikely to account for resistance to AIs in the majority of tumors [9]. The identification of robust predictive biomarkers for resistance or sensitivity to AIs is therefore a research priority.

The observed changes in transcription following treatment with AIs led to the identification of gene expression signatures in pre-treatment tumor samples reported to be predictive of response to AIs, as measured by a decrease in tumor volume [6,10]. To our knowledge, neither of these signatures has been validated in a larger independent cohort. The challenges of translating predictive gene expression signatures into clinically useful tools are now well-recognized [11]. These include, but are not limited to, the facts that 1) resistance to a given agent may be mediated through multiple distinct pathways in different tumors, 2) the low sensitivity of microarray platforms for low-level changes in expression or for changes in non-modal clones may not detect the mechanism, and 3) resistance to an agent may not manifest in transcriptomic changes, but may be mediated through mutations or epigenetic aberrations that do not result in overt transcriptomic changes.

Gene amplification is a common mechanism of oncogene activation in cancer [12]. There are multiple reports describing the association between specific gene amplifications and resistance to various anti-cancer therapies. For example, in breast cancer, resistance to tamoxifen is associated with *FGFR1* amplification [13], while amplification of *CCNE1* [14] and *IGF-1R* [15] are associated with resistance to trastuzumab. Further examples abound in other tumor types, such as the association of *ERBB2* [16] and *CRKL* [17] amplification with resistance to anti-epidermal growth factor receptor (EGFR) targeted agents in non small-cell lung cancer and *YAP* amplification with resistance to doxorubicin in hepatocellular carcinoma [18].

Alternative approaches to identifying biomarkers of resistance to therapy include the use of genome-wide copy number profiling microarrays to compare the patterns of copy number aberrations (CNAs) between responders and non-responders. This approach has identified genomic loci associated with response to various chemotherapeutic agents in ovarian carcinoma [19], large B-cell lymphoma [20] and colorectal carcinoma [21], to name but a few. Amplified regions frequently encompass multiple genes and not all genes within an amplicon are overexpressed and of functional significance [22].

By integrating genome-wide copy number profiling data and gene expression data, lists of genes associated with response to specific therapies can be enriched for biologically relevant targets (for example, the identification of *FGFR1* amplification as a modulator of tamoxifen response [13]). More recently, publication of the Cancer Cell Line Encyclopedia [23] and the Genomics of drug sensitivity [24] datasets has demonstrated the power of integrative genomic and functional genomic approaches in identifying determinants of response to targeted therapies. To date, there are limited genome-wide data identifying CNAs that are associated with response to AI therapy measured by Ki67 as an intermediate endpoint. Ellis *et al*. [25] compared whole-genome analysis in resistant versus sensitive tumors, using Ki67 at surgery as the index of response; however, the study focused on mutational background and somatic structural variations and not specific copy number and expression changes.

The aims of this study were to 1) relate patterns of copy number aberrations to molecular and proliferative response to endocrine treatment, 2) study differences in the patterns of copy number aberrations between breast cancer samples pre- and post-AI neoadjuvant therapy, and 3) identify putative biomarkers for resistance to neoadjuvant AI therapy using an integrative analysis approach.

## Materials and methods

### Patients and samples

To identify molecular determinants of response to AIs, primary breast cancer samples were retrieved from a clinical trial and a clinical study in which core biopsies were taken before and after commencing neoadjuvant AIs; these were the FAIMoS (ZD1839IL/0223) study [26], and the Edinburgh study [6].

FAIMoS is a study of those tumors treated with anastrozole alone in a phase II placebo-controlled trial that compared neoadjuvant anastrozole with or without gefitinib in early breast cancer. Postmenopausal women with stage I to IIB ER-positive and/or progesterone receptor (PR)-positive non-metastatic breast cancer were eligible. All patients received neoadjuvant anastrozole for 16 weeks until surgery. Core-cut biopsies were taken at baseline, after 2 weeks on treatment, and after 16 weeks. Changes in the Ki67 labeling index were assessed by performing immunohistochemistry with the MIB1 antibody (DakoCytomation, Denmark) and used as the primary response variable. Ki67 was scored as a percentage of positive cells in 10 high-power fields chosen to represent the overall staining across the section. Sufficient material was available from 51 patients for this study, none of whom belonged to the gefitinib arm of the trial (Additional file 1: Figure S1). A reduction in Ki67 of more than 50% after 2 weeks of anastrozole was used to identify responders, as previously described [27]. The study received approval from an institutional review board at each site and written informed consent was obtained from each patient before participation.

The Edinburgh study prospectively recruited patients to receive neoadjuvant letrozole for 3 months, with baseline and 2-week core biopsies taken. For this study, samples from 52 patients were accrued from Edinburgh ECMC tissue bank but sufficient material was available from 33 patients. Paired biopsy samples before and after 3 months of letrozole were available for 19 of these patients (Additional file 1: Figure S1). Clinicopathological information for samples utilized in this study are shown in Additional file 2: Table S1. All patients provided informed consent and sample collection was approved by the local research ethics committee (Lothian Research Ethics Committee: 2001/8/80 and 2001/8/81). Both studies were conducted in accordance with the 1964 Declaration of Helsinki and International Conference on Harmonization/Good Clinical Practice guidelines.

### Cell lines

MDA-MB134-VI, MCF7, and T47D were purchased from ATCC (LGC Standards, Teddington, UK), while SUM44 was purchased from Asterand (Royston, UK). Cells were cultured at 37°C in a 5% CO_2_ atmosphere in phenol-free RPMI medium (MDA-MB134-VI and T47D) or DMEM (MCF7 and T47D) supplemented with 10% fetal bovine serum and 1nM 17β-estradiol (E2). Prior to experimentation, cell lines were stripped of estrogen by culturing for 24 hours in their appropriate medium containing 10% dextran charcoal-stripped fetal bovine serum (DCC) as previously described [28]. The generation of the long-term estrogen deprived (LTED) cell line derivative of MCF7 has been previously described [29]. All cell lines were shown to be mycoplasma-free and authenticated by means of short tandem repeat analysis (PowerPlex® 1.2 System, Promega, Fitchburg, WI, USA).

### Nucleic acid extraction

Tumor samples from FAIMoS and Edinburgh were microdissected under a stereomicroscope (Olympus SZ61, Tokyo, Japan) using a sterile needle to ensure a tumor cell content >90% as previously described [30]. DNA extraction was performed by proteinase K digestion and phenol-chloroform precipitation. Total RNA from FAIMoS samples was extracted from un-microdissected sections using the RNeasy kit (Qiagen, Hamburg, Germany) as previously described [31]. RNA purity and integrity was assessed using the Agilent Bioanalyzer (Santa Clara, CA, USA), with samples only being analyzed if RNA integrity scores were >5.0. Sample processing and RNA extraction from samples of the Edinburgh cohort has been previously described [6]. For cell lines, DNA was extracted using the DNeasy blood and tissue kit (Qiagen) and RNA was extracted using the RNeasy kit (Qiagen).

### Microarray comparative genomic hybridization (aCGH)

Fifty one FAIMoS and 33 Edinburgh patients had sufficient good-quality DNA to run aCGH. The aCGH platform used for this study comprises approximately 32,000 bacterial artificial chromosome (BAC) clones tiled across the genome, which has been shown to be as robust as, and to have comparable resolution with high-density oligonucleotide arrays [32,33]. DNA labeling, array hybridization, and image acquisition were performed as previously described [34]. aCGH data were pre-processed and analyzed using the Base.R script in R version 2.9.0 as previously described [35]. A detailed description of aCGH analysis methods is provided in the Additional file 2. Microarray data is available in Array Express [36].

### Gene expression profiling

RNA of sufficient quantity and quality was available from 47 of the baseline samples from the FAIMoS cohort. RNA amplification, labeling, and hybridization on HumanWG-6 v2 Expression BeadChips (Illumina, San Diego, CA, USA) were performed according to the manufacturer's instructions at a single Illumina BeadStation facility [37]. The Expression BeadChips cover more than 48,000 transcript probes and their annotation is publicly available. Gene expression probes were mapped to ensemble genes using ensembl build 37 assembly 55. Matched copy-number states were assigned to gene expression probes based upon the median of all BAC probes overlapping the genomic positions of each corresponding ensembl gene. The integrative analysis overlapping the aCGH and expression data is described in detail in Additional file 2. Microarray data have been deposited in the Sagebase [5,38].

### Proliferation assays

To assess the effect of silencing of *CHKA*, *SAPS3* or *LRP5* on ER-driven proliferation, cells were seeded in DCC medium and transfected with pools and individual siRNAs targeting CHKA (M-006704-01 Thermo Scientific, Epsom, UK), SAPS3 (L-014646-01-0005 Dharmacon, Lafayette, CO, USA) and LRP5 (L-003844-00-0005 Dharmacon) the following day using RNAiMAX (Invitrogen, Paisley, UK) or Dharmafect3 (Dharmacon) according to manufacturers’ instructions and as previously described [39]. Non-targeting control siRNA pools (siControl pool 2, D-001206-14-20 Dharmacon) and a SMARTpool targeting PLK1 (M-003290-01 Dharmacon) were used as negative and positive controls respectively. After 24 hours, cells were treated with E2 as indicated and cell viability assessed after 6 days using the Cell Titer-Glo luminescent cell viability assay according to manufacturer’s instructions. Cell proliferation was displayed as fold-change over siControl transfected, DCC-treated cells. Each experiment consisted of six replicates, and data presented are representative of at least two biological replicates in each case. In parallel to each assay, the same cells were transfected in 24-well plates for RNA extraction 48 hours post-transfection to confirm target gene silencing in each assay by quantitative PCR and whole cell lysates generated 72 hours post transfection. CHKA silencing was validated using a second set of independent siRNAs and four shRNAs described in detail in Additional file 2: Supplementary materials and methods and Additional file 3: Figure S2.

### Immunoblotting

Whole cell lysates and western blotting was performed as previously described [40] using antibodies (see Additional file 2: Table S2) diluted in 5% BSA/TBST according to manufacturer’s recommendations. Incubation with an horseradish peroxidase (HRP)-conjugated secondary antibody was then performed, and protein detected by chemiluminescence (Supersignal, Amersham, UK). Protein-band densitometry was measured with ImageJ software.

### Cell flow cytometry

Cells were harvested by scraping and were washed in PBS. This was followed by fixation by adding ice-cold 70% ethanol and maintaining the cells at 4°C overnight. The ethanol-fixed cells were centrifuged and resuspended in RNase A at 10 mg/mlU/ml and incubated at room temperature for at least 30 minutes. The cells were stained in PBS containing 50 μg/ml propidium iodide (PI) and stored in the dark until analysis. Cell cycle analysis of 10,000 cells per sample was carried out in a flow cytometer (LSR II, Becton, Dickinson, San Jose, CA, USA). The data were analyzed with BD FACSDiva (BD Biosciences, San Jose, CA, USA).

### Quantitative real-time PCR (qRT-PCR)

Reverse transcription was performed with Superscript III (Invitrogen, UK), using 1 μg total RNA per reaction. qRT-PCR was carried out using TaqMan® chemistry on the ABI Prism 7900HT (Life Technologies Ltd, Paisley, UK), and analyzed using the ΔΔ cycle threshold (ΔΔCt) method in triplicate, as previously described [41]. Probes for CHKA (Hs00957875_m1), *TFF1* (Hs00907239_m1) and *GREB1* (Hs00536409_m1) were purchased from Life Technologies. The expression level of a housekeeping gene (HKG), *FKBP15* (Hs00391480-m1), was also assessed.

### Copy number analysis by qRT-PCR

Copy number in cell line DNA was measured by Taqman copy number assays as described previously [42]. Twenty nanograms of DNA were run in quadruplicate for each cell line. Assays for *CHKA* (Hs03778879_cn), *LRP5* (Hs06321584_cn), *SAPS3* (Hs06297988_cn) and reference gene *TERT* were purchased from Life Technologies.

### ER/ERE transactivation assays

To study the effect of RNAi-induced silencing of *CHKA* on ER/ERE transactivation, the activity of a the EREIItkluc reporter was assessed following silencing of CHKA in DCC media and in the presence of E2, as previously described [43]. Further details are available in Additional file 2.

### Statistical analysis

Statistical analysis was carried out with PRISM v.6. Spearman correlation was used to compare the proportion of the genome altered and Ki67 levels and aCGH circular binary segmentation (cbs) ratios and Ki67 change. The Mann-Whitney *U*-test and Kruskal-Wallis analysis were used to compare Ki67 levels and Hicks scores. The *t*-test was used to compare the effect of each siRNA to siControl in DCC conditions and to compare the effect of siCHKA4 in DCC and 1nM E2 conditions in the transactivation assays. One-way analysis of variance (ANOVA) was used to analyze the effect of siRNAs and E2 escalating doses on each cell line and to analyze the effect of siCHKA on the mRNA levels of TFF1 and GREB1.

## Results

### The proportion of the genome with copy number aberrations in ER-positive breast cancer correlates with Ki67 labeling index at baseline but not with changes in Ki67 following AI therapy

Ki67 expression is a well-established marker of proliferation. Short-term change in Ki67 has been validated as an intermediate endpoint of clinical benefit from AIs [27]. To determine if Ki67-based response to AI is associated with distinct patterns of copy number aberrations, DNA samples from 84 ER-positive breast carcinomas from postmenopausal women were subjected to copy number profiling using a tiling path 32 K BAC array.

**Figure 1:**
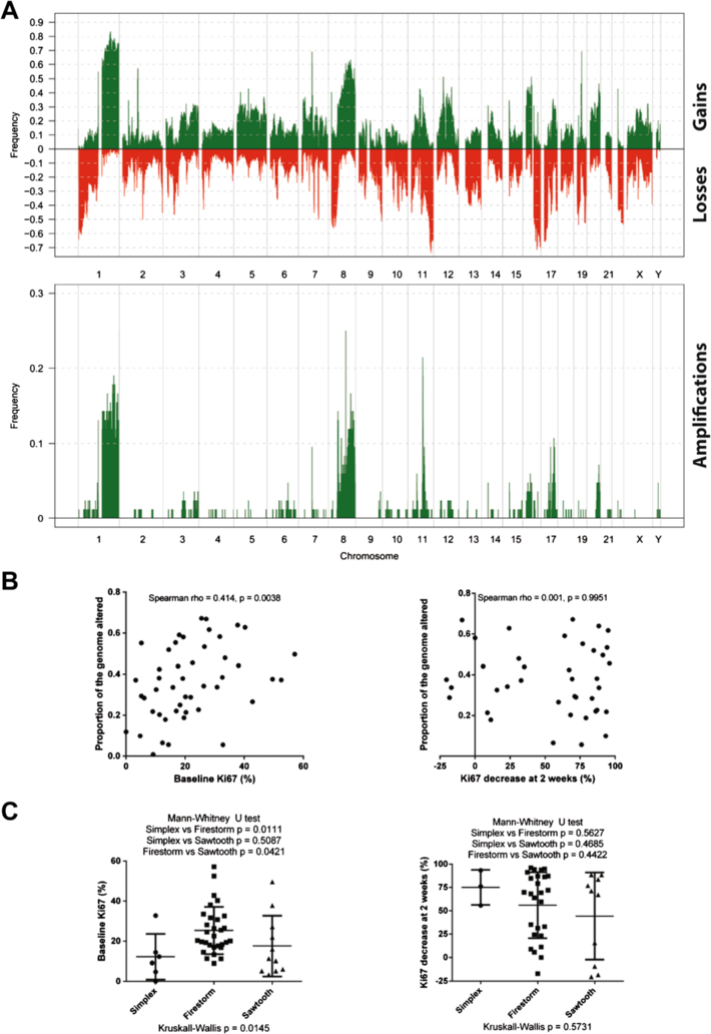
**Correlation of the proportion of the genome with copy number aberrations and the patterns of copy number aberrations with Ki67 indices of proliferation and response to aromatase inhibitors. (A)** A frequency plot of gains and losses (top panel) or amplifications (bottom panel) in 84 samples of ER-positive breast carcinomas from post-menopausal women before treatment with anastrozole. The proportion of tumors in which each bacterial artificial chromosome (BAC) clone is gained/amplified (green bars) or lost (red bars) is plotted (y-axis) for each BAC clone according to its genomic position (x-axis). **(B)** Scatter plots showing the correlation between the proportion of the genome with copy number aberrations on the y-axis and baseline Ki67 expression levels (left side) or the percentage decrease in Ki67 expression levels after 2 weeks of anastrozole (right side). Spearman correlation demonstrates a statistically significant positive correlation between proportion of the genome altered and baseline Ki67 expression levels. **(C)** Dot plots demonstrating the relationship between the distinct patterns of copy number aberrations defined by Hicks *et al.* and baseline Ki67 expression levels (left side) or the percentage decrease in Ki67 expression levels after 2 weeks of anastrozole (right side).

The most common copy number aberrations identified were gain of 1q23.1-44, 7q11.1-11.21, 8q21.2-q24.13 and 19q11, loss of 1q36.33-p36.12, 11q22.3-q25, 17p13.3-p11.2 and 16q21-q24.3 and amplification at 1q21.3-q44, 8p12-q24.3.3 and 11q13.2-q13.4 (Figure 1A and Additional file 2: Table S3). Given that all the tumors were ER-positive and predominantly of low histological grade, the frequent loss of 16q is in keeping with previous reports [44]. The most common amplified gene was *CCND1* (21%).

The proportion of the genome with copy number aberrations (proportion of the genome altered) was calculated for each case at baseline and utilized as an index of genomic instability. This was significantly correlated with Ki67 baseline levels (Spearman *r* = 0.414, *P* = 0.0038, Figure 1B, left panel), consistent with the notion that highly proliferative tumors accumulate copy number aberrations at a more rapid rate. This correlation was not due to grade (one-way ANOVA, *P* = 0.58). Furthermore, a significant correlation was not identified when comparing the proportion of the genome altered with Ki67 2-week decrease percentages (Figure 1B, right panel).

The patterns of copy number aberrations in each case were classified using the genomic pattern classification proposed by Hicks *et al.* [45]. This consists of the simplex pattern (broad segments of duplication and deletion usually entire chromosomes), the sawtooth pattern (narrow segments of duplication and deletion more or less affecting all chromosomes) and the firestorm pattern (resembling the simplex type but with at least one localized region of amplification within a single chromosome). Thirteen tumors were classified as simplex, 23 as sawtooth and 48 as firestorm. A significant difference in baseline Ki67 labeling index was identified between cases with the firestorm pattern and both sawtooth and simplex patterns (Figure 1C, left panel), but not with Ki67 2-week decrease (Figure 1C, right panel).

### Pairwise analysis of pre- and post-AI-treated samples identifies differences in copy number aberrations following treatment

**Figure 2:**
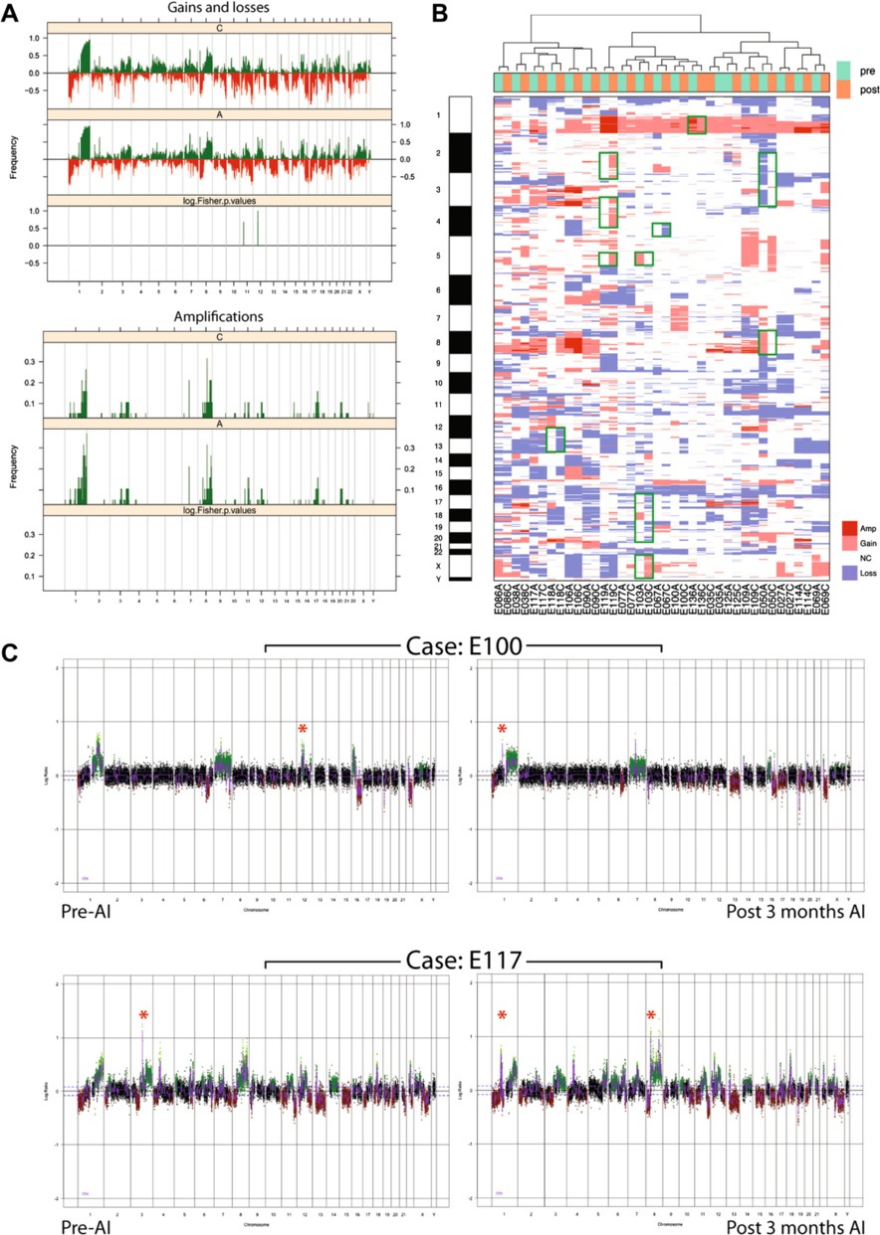
**Grouped and pairwise analysis of matched pre- and post-aromatase inhibitor (AI) therapy copy number profiles. (A)** Frequency plot of copy-number gains and losses (top) or amplifications (bottom) in 19 matched pre- and post-letrozole-treated breast cancer samples. The proportion of tumors in which each bacterial artificial chromosome (BAC) clone is gained (green bars) or lost (red bars) is plotted (y-axis) for each BAC clone according to its genomic position (x-axis). No significant differences were identified between the two components. **(B)** Hierarchical cluster analysis performed with array comparative genomic hybridization (aCGH) categorical states (that is, gains, losses and amplifications) using the Euclidean distance metric and Ward's algorithm of 19 matched pre- and post-letrozole treatment samples from estrogen receptor (ER)-positive breast carcinomas. Matched samples from each patient preferentially cluster together, and have similar patterns of copy number aberrations. In some cases, small regions show differential copy number states between matched samples (green squares), but these are private events. The heatmap displays each case along the x-axis and the genomic position along the y-axis. Amp, amplification; Gain, copy number gain; Loss, copy number loss; NC, no copy number change. **(C)** Genome plots of pre- and post-letrozole samples from two patients. The genomic position is plotted along the x-axis and circular binary segmentation (cbs)-smoothed log2 ratio on the y-axis; amplifications are shown in bright green, gains in dark green, losses in dark red and normal copy number in black. Red stars denote amplicons present in only one sample of a matched pair.

To determine if treatment with letrozole is associated with changes in the copy number profiles of ER-positive breast carcinomas, DNA from 19 matched pairs of pre- and post-treatment breast carcinoma samples were subjected to copy number profiling as described above. A grouped analysis was performed to compare the patterns of copy number aberrations in the pre-treatment samples with those in the post-treatment samples. Matched samples largely had similar patterns of copy number aberration (Figure 2A) and clustered together (Figure 2B), and no copy number aberration was significantly more frequently present in the pre- or post-AI samples.

Despite these findings, a careful inspection of the heatmap identified genomic loci affected by copy number aberrations only in the pre- or post-treatment samples of 7 out of 19 cases (Figure 2B, green boxes). Given that the heatmap is constructed from aCGH copy number states rather than continuous copy number ratios, these differences could be due to artificial changes in ratios near the cutoff for defining gains or losses. A detailed pairwise analysis of the copy number profiles confirmed that in most cases where differences in gain/loss were noted, the trend in the copy number aberration was similar in each sample of a pair, but in one or other, the threshold for gain or loss was not reached. In two cases, however, amplicons were identified that were present in only one of the matched samples from a given patient. In the first case, amplification at 12q12 was present at baseline but absent after 3 months of AI therapy while amplification at 1q31.1-q41 was apparent only after 3 months of AI therapy (Figure 2C, top panels). In the second case, amplification at 1p22.2-p22.1 and 8p11.23-p11.22 was absent at baseline but present after 3 months of AI therapy, while amplification at 3q13.11 was present at baseline and absent after 3 months of AI therapy (Figure 2C, bottom panels). These data demonstrate that while samples by and large retain similar patterns of copy number aberrations during AI treatment, there is evidence of changes in focal copy number aberrations following treatment with AIs.

### Integrative analysis of copy number and gene expression profiling data with Ki67-based response data identifies amplified genes associated with a poor response to AI therapy

**Figure 3:**
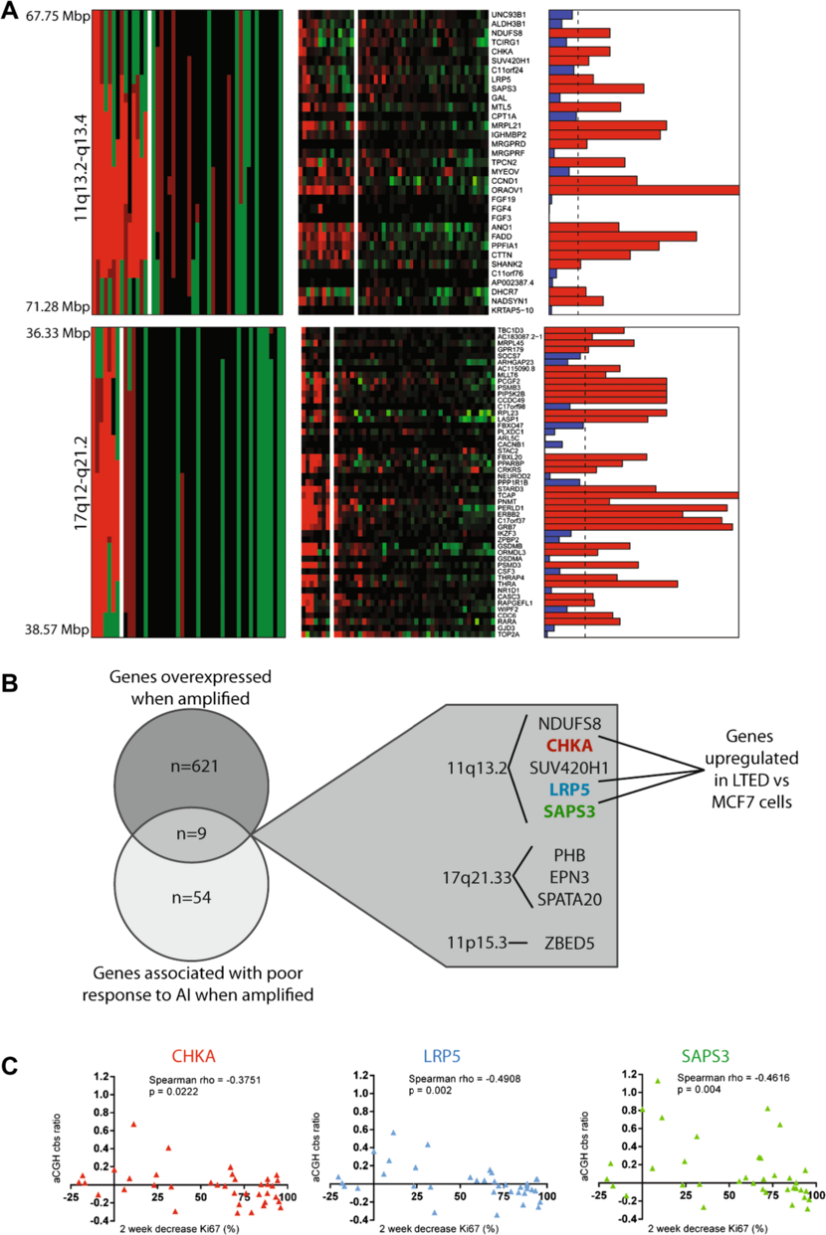
**Integrative analysis of microarray-based comparative genomic hybridization, gene expression and Ki67-based response data. (A)** Matched heatmaps of gene expression and aCGH within two amplified loci; 11q13.2-q13.4 and 17q12-q21.1. Bar plots show the result of a Mann-Whitney *U*-test for expression as a continuous variable and gene amplification as the grouping variable. Bars in red show adjusted *P*-values <0.05. aCGH, green copy number loss; black, no copy number change; dark red, copy number gain; bright red, gene amplification; gene expression: green, downregulation; red, upregulation; MWU, Mann-Whitney *U*-test; adjp, adjusted *P*-value. **(B)** Venn diagram shows the intersect between the list of genes that are overexpressed when amplified and those genes that are associated with a poor response to AI when amplified. The call-out box lists these genes and their loci, highlighting that only three genes are upregulated in long-term estrogen deprived (LTEDs). **(C)** Scatter plots demonstrating that for each of the three genes selected for functional validation, significant negative correlation was identified between the aCGH-derived cbs ratios and the percentage decrease in Ki67 following 2 weeks of AI therapy. In each plot, cbs-smoothed ratios are plotted on the y-axis while the percentage decrease in Ki67 at 2 weeks is plotted on the x-axis. Red, CHKA, Blue, LRP5, Green, SAPS3.

To determine which genes were overexpressed when amplified, an integrative analysis was first performed by overlaying gene expression data with aCGH-derived copy number data for a subset of 47 patients from which both datasets were available. In the first instance, to determine which genes had expression levels that were copy number-regulated, aCGH data were utilized as a continuous variable and cbs-smoothed ratios were correlated with microarray-derived gene expression levels. This approach identified 3,706 genes with expression levels that were significantly correlated with copy number (Pearson correlation, adjusted *P* <0.05, Additional file 2: Table S4). Next, cbs-smoothed ratios were used to define copy number states on a gene by gene basis, and this was used as a grouping variable (that is, amplified versus non-amplified), while expression levels were used as the dependent variable, as previously described [35]. This approach identified 628 genes, which were significantly overexpressed when amplified (Mann-Whitney *U*-test adjusted *P* <0.05, Additional file 2: Table S5). The two most strikingly associated regions were at chromosome 11q13.2-q13.4 and 17q12-q21.2 (Figure 3A).

Next, to identify genes with copy numbers associated with proliferative response to AI therapy, an integrative analysis of baseline aCGH with Ki67 decrease after 2 weeks of AI therapy was performed. When aCGH-derived cbs-smoothed ratios were correlated with 2-week Ki67 decrease using Spearman’s correlation, 48 genomic loci harboring a significant association with 2 week Ki67 decrease were identified. Forty-one regions positively associated (Additional file 2: Table S6A) and seven regions negatively associated (Additional file 2: Table S6B) to which a total of 734 genes mapped.

By combining the analyses above, a set of nine genes was identified that, when amplified, were associated with a poor proliferative response to AI therapy (defined as a <50% reduction in Ki67 labeling index after 2 weeks of AI therapy) and that were significantly overexpressed when amplified (Figure 3B). These genes were clustered at 11q13.2 (*NDUFS8*, *CHKA*, *SUV420H1*, *LRP5* and *SAPS3*), 17q21.32 (*CALCOCO2*, *UBE2Z*, and *SNF8*), 17q21.33 (~1.5Mbp distal to the cluster at 17q21.32, encompassing *PHB*, *EPN3,* and *SPATA20*) and 11p15.3 (*ZBED5*).

It is plausible that amplification of one or more of these genes could potentially constitute a negative predictive biomarker for AIs. To test this hypothesis, a well-studied model of AI-resistance was utilized (the MCF7-LTED model [29]). Three of the nine genes were upregulated in MCF7-LTED cells compared to MCF7 cells (*CHKA, LRP5* and *SAPS3)*. These genes that are overexpressed when amplified and are associated with a poor proliferative response to AI therapy (Figure 3C) were taken forward for functional validation.

### Functional validation identifies *CHKA* as a potential modulator of ER-driven proliferation

**Figure 4:**
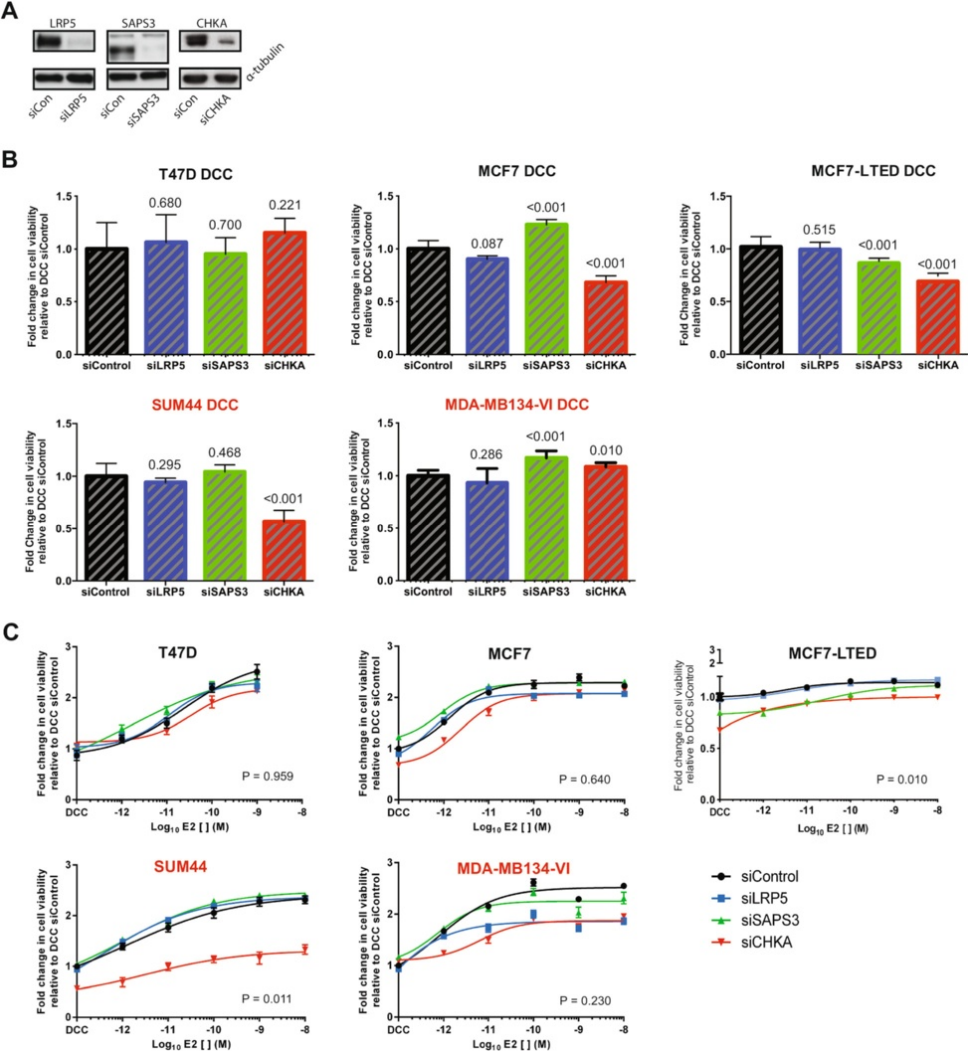
**Functional validation of genes identified as potential modulators of aromatase inhibitor (AI) response when amplified and overexpressed. (A)** Western blotting of lysates from SUM44 cells: each of the three genes, silenced and blotted for, was used to demonstrate siRNA efficacy and antibody specificity. **(B)** Panel of estrogen receptor (ER)-positive cell lines were used to assess the effect of RNA-interference-induced silencing of CHKA, LRP5 and SAPS3 on cell viability. Cell line names in red font harbor amplification of these genes; those named in black do not. Data for each knockdown (performed using SMARTpools) were normalized to readings from cells transfected with a non-targeting control and grown in dextran charcoal-stripped (DCC) media. Data are representative of six replicates from at least two independent experiments. Numbers indicate *t*-test *P*-value. **(C)** The same panel was used to assess the effect of RNA-interference-induced silencing of CHKA, LRP5 and SAPS3 on ER-driven proliferation. After growing and transfecting cell lines in DCC, cell viability was assessed as a surrogate marker of proliferation in the presence of increasing concentrations of estrogen (E2). Cell line names in red font harbor amplification of these genes; those in black do not. Data for each knockdown (performed using SMARTpools) were normalized to readings from cells transfected with a non-targeting control and grown in DCC media. Drug curves were inferred from non-linear regression. Error bars represent standard error of the mean. Data are representative of six replicates from at least two independent experiments. *P*-value is for one-way analysis of variance.

The effect of silencing of each of *CHKA*, *SAPS3* and *LRP5*, was assessed using validated siRNA SMARTpools, on ER-driven proliferation in a panel of ER-positive breast cancer cell lines that harbored amplification of *CHKA, LRP5* and *SAPS3* (that is, SUM44 and MDA-MB134-VI) or normal copy number at 11q13-q14 (that is, T47D and MCF7), as defined by copy number analysis (Additional file 4: Figure S3A). Of the three genes investigated, only CHKA appeared to constitute a potential driver, given that its RNAi-mediated silencing resulted in a consistent reduction in cell viability in SUM44 cells, but surprisingly, had no effect in the MDA-MB134-VI (Figure 4A, B and C). However, in contrast to SUM44, the MDA-MB134-VI did not show an increase in CHKA expression, providing a likely explanation for this discordance (Additional file 4: Figure S3B).

To address the role of overexpression of *CHKA, LRP5* and *SAPS3* in AI resistance, in the absence of amplification we utilized the MCF7-LTED cell line [29]. siCHKA had a significant impact on the viability of MCF7 cells, which lack amplification of the CHKA locus, however, this was only apparent in the absence of E2. In addition, silencing of CHKA in the MCF7-LTED cell line produced a significant loss of viability in the absence of E2.

Taken together, these data suggest that in cells harboring the amplification with concomitant overexpression, CHKA is required for cell proliferation. Furthermore, the cellular context may well be important. For instance, in acquired resistance to estrogen-deprivation, CHKA expression may be required for the survival of cells that rely on ligand-independent ER activity, even in the absence of *CHKA* gene amplification.

### Mechanistic investigation of the effect of CHKA on proliferation

**Figure 5:**
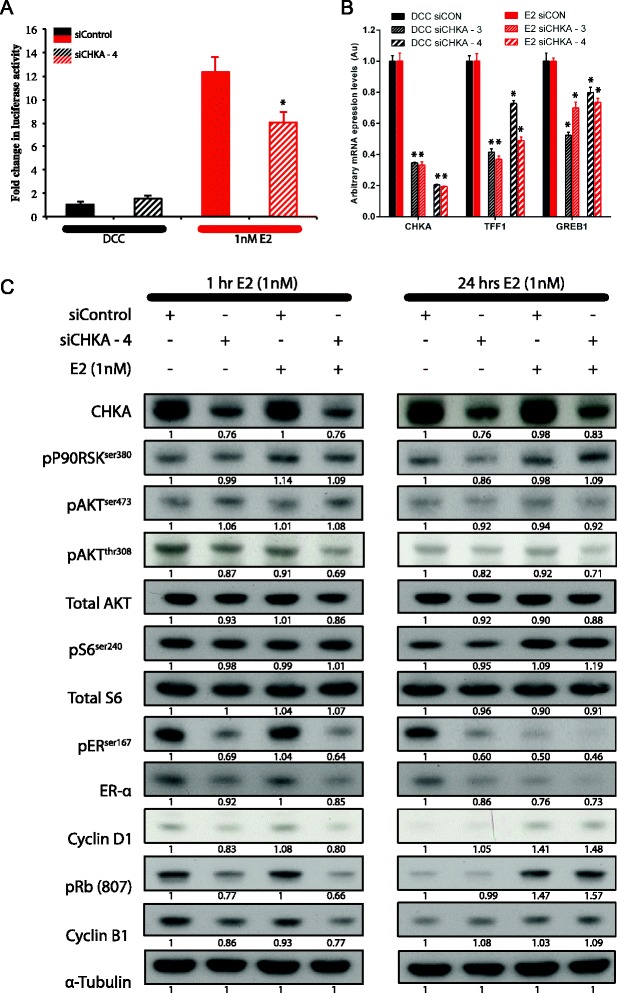
**Mechanistic assessment of the effect of CHKA in modulating response to aromatase inhibitor (AI) therapy. (A)** Following RNA-interference-mediated silencing of CHKA, SUM44 cells were transfected with an estrogen receptor (ER)/ERE luciferase reporter construct, and then treated with E2 or dextran charcoal-stripped media (DCC) for 2 days before reading luciferase activity. Data are normalized to the activity in the DCC-treated control transfected cell lines. *Significant *P*-value (<0.05) between the indicated column and corresponding siControl-equivalent. **(B)** To validate effects seen with the ER-ERE reporter assay, expression levels of two well known ER-regulated genes (*TFF1* and *GREB1*) were assessed by quantitative real-time PCR following RNA-interference-induced silencing of CHKA. Data normalized to DCC-treated control transfected cell lines; *P-value <0.01 between indicated column and corresponding siCON equivalent. **(C)** Following RNA-interference-induced silencing of CHKA, cell lysates were subjected to gel electrophoresis and western blotting using indicated antibodies. Cells were treated with 1nM E2 for 1 h or 24 h following transfection, to represent the two phases of ER dynamics (early active- and late turnover phase). Blots are representative of at least two independent experiments; numbers below each band represent densitometry analysis of intensity, measured as a ratio of the siControl with no siCHKA or E2 treatments.

To determine if the effects of CHKA silencing on proliferation may be mediated by ER transcriptional activity, an ER/ERE reporter assay was performed in the cell line that demonstrated the most profound effect on proliferation (that is, SUM44). Following silencing of CHKA, there was approximately 25% reduction in luciferase activity of the ER/ERE reporter construct in the presence of E2 (Figure 5A, red bars), suggesting that CHKA modulates ER transactivation. Consistent with these findings, the expression levels of two genes, previously reported to be estrogen-regulated (that is, GREB1 and TFF1 [37]), were reduced following silencing of CHKA (Figure 5B).

**Figure 6:**
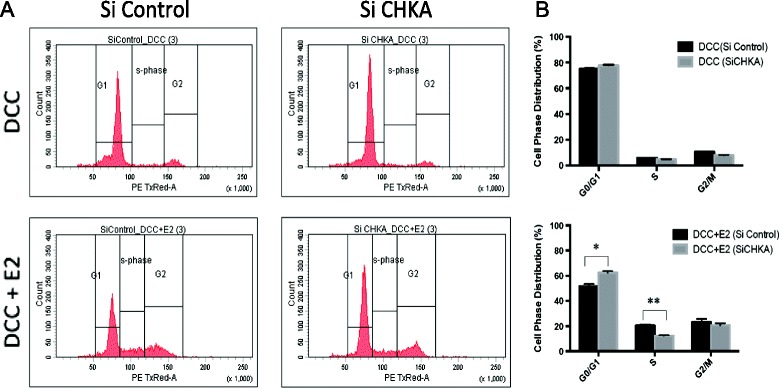
**The effect of siRNA (CHKA) on cell cycle as determined by flow cytometry. (A)** Flow cytometry was used to compare DNA content in Sum44 WT cells treated with dextran charcoal-stripped media (DCC) and DCC + E2. There was no significant difference between the cell cycle phases in the cells treated with DCC. CHKA arrests cells at the G1/S, but not at the G2/M phase of the cell cycle in cells treated with DCC + E2. **(B)** The percentage of cells at each cell phase: G0/G1, S, G2/M are shown on the bar chart as the mean ± SD. The experiment was performed in triplicate; **P <0.005 and *P <0.01.

Finally, to determine the downstream signaling pathways involved in the CHKA-mediated reduction in ER transactivation, western blotting following CHKA silencing was performed using antibodies to major signaling pathways known to influence ER activity (that is, p90RSK, AKT, and S6, Figure 5C). These experiments were performed in a time course to assess the effects of CHKA silencing during the activation phase of ER signaling (1 hour treatment with E2) and during the turnover phase of ER signaling (24 hour treatment with E2). Following silencing of CHKA, a reduction in ER-alpha expression was noted with a subsequent reduction in phosphorylation at ER^Ser167^ (apparent only when assessed after 1 hour of treatment with E2), providing further support for the finding of reduced ER/ERE transcription activity and reduced expression of key ER-regulated genes. While there was no appreciable difference noted in the expression of pp90RSK or pAKT^Ser473^ following CHKA silencing, there was a reduction in pAKT ^Thr308^, accompanied with a reduction in total AKT, which was more apparent in the presence of E2. We assessed the effect of CHKA ablation on cell-cycle progression by flow cytometry (Figure 6) and the expression of cyclin B1, pRb and cyclin D1 by immunoblotting (Figure 5C). Silencing of CHKA in both the presence and absence of E2 resulted in a marked decrease in each of these proteins. Taken together these data suggest that CHKA may regulate ER activity through AKT phosphorylation in a p90RSK-independent manner, leading to cell-cycle progression.

## Discussion

In this study, an integrative analysis of copy number profiling, gene expression microarray and Ki67-based AI response data was performed using data from samples in two cohorts of neoadjuvant AI therapy in postmenopausal patients with ER-positive early breast cancer. No significant differences in the frequency of gene copy-number aberrations were found when pre- and post-AI therapy samples were compared. This is to be expected given that there is evidence for numerous mechanisms of resistance to AIs many of which would not be expected to be dependent on gene copy-number changes [7]. Non-recurrent differences in copy number at specific loci (that is, 1p11.23-p11.22, 1q31.1-q41, 3q13.11, 8p11.23-p11.22 and 12q12) between samples before and after 3 months of letrozole therapy were observed in 6 of 19 studied cases (Figure 2B). This observation suggests that the selective pressure applied by AI therapy may result in the selection of non-modal clones, causing enrichment or loss of cells harboring specific amplicons, and that resistance to AIs may constitute a convergent phenotype [11]. An alternative explanation, however, is that these differences in gene copy-number profiles may be merely a manifestation of spatial intra-tumor genetic heterogeneity. Indeed, this study highlights the need to interrogate intra-tumor genetic heterogeneity for a full understanding of the mechanisms of resistance to specific agents, using combinations that can overcome the challenges that may be posed by intra-tumor spatial genetic heterogeneity. All the same it should be noted that in this study few differences were noted in the copy number landscape between pre-treatment and on-treatment cores: any confounding degree of genetic heterogeneity that is dependent on the small amount of a tumor sampled in cores would have been revealed by that comparison.

Overlaying gene expression data with copy-number profiling data identified a set of 628 genes, which are significantly overexpressed when amplified, including genes at two commonly amplified regions (11q13.2-q13.4 and 17q12-q21.2). Nine of these 628 genes were also negatively correlated with the decrease in Ki67 expression after 2 weeks of AI therapy (a surrogate for response to AIs). These clinical data are reflective of pre-existing, *de novo* mechanisms of resistance. To parallel these observations we assessed the possible importance of the genes in a panel of ER+ cell lines, two of which harbored the amplification and two of which did not. We prioritized the functional analysis of three of the nine genes based on differential expression between MCF7 and the LTED derivative (a model for acquired AI resistance), followed by functional validation. This pipeline identified *CHKA* as a gene that is significantly overexpressed when amplified, and when amplified is associated with a poor Ki67 response to AI therapy. Mechanistic investigations revealed that CHKA expression modulates ER transcriptional activity via AKT and S6 phosphorylation, but independently of p90RSK activity, which resulted in a reduction in cell-cycle progression markers. Antagonizing ER signaling has been shown to attenuate cyclin-dependent kinase (CDK)/cyclin complexes at multiple levels [46]. Furthermore, ER modulates transcription of cyclin D1. Hence suppression of ER signaling leads to inhibition of CDK activity and the maintenance of Rb in a phosphorylated and active state inhibiting progression to S-phase.

*CHKA* is located at 11q13.2 and encodes the protein choline kinase alpha (CHKA), which catalyses the phosphorylation of choline as the first step of the Kennedy (phospholipid synthesis) pathway [47]. Choline phosphorylation by CHKA has been shown to be upregulated in many cancer types, including breast, lung, colorectal and prostate cancer [48]. In our study we identified *CHKA* amplification in 4% of cases, confirming previous reports [49,50]. Amplification at 11q13-q14 is a complex event. It is currently accepted that at least four cores of independent amplification are found at this locus [51]. *CHKA* lies between the smallest regions of amplification of the first and second core of the 11q13-q14 amplicon. Curtis *et al.* identified a high-risk group of ER-positive luminal tumors with amplification of 11q13/14 (Int2). This group harbors amplification of several genes such as *CCND1* and *EMSY* and is in close proximity to the region containing CHKA [49]. In the large METABRIC study of CNV and gene expression CHKA amplification does not associate with survival but its overexpression did correlate with poorer survival. It should be noted, however, that analyses of survival are generally unhelpful for detecting the impact of response or resistance to a particular treatment.

Using RNAi-mediated silencing and ERE-reporter techniques, the role of CHKA on ER-driven proliferation was characterized in this study, highlighting the importance of functional characterization of genomic and transcriptomic aberration using appropriate phenotypes as experimental readouts in multiple models. In this case, for example, previous studies demonstrated a significant effect of CHKA silencing on proliferation in MCF7 cells [52]. However, those experiments were performed in complete growth medium; in the same study, silencing CHKA in serum-starved MCF7 cells produced no difference in proliferation, and an interaction between CHKA, EGFR and c-Src was demonstrated and found to be required for the pro-proliferation effect of CHKA. Assessment of the MCF7-LTED cell line showed that whilst they do not harbor amplification of CHKA the transcript level is significantly increased. Furthermore, this cell line shows elevated levels of both the EGF canonical pathway and c-Src [53]. These data provide further support for the proposed mechanism of CHKA on ER-driven proliferation proposed in this study, which warrants further investigation.

This study has a number of limitations. First, it was limited by relatively small sample size. Although the two trials utilized in the study recruited over 160 suitable patients, only a subset of these samples were available in sufficient quantity for use in this study. The use of the neoadjuvant setting with Ki67 change as its primary endpoint provides substantially greater statistical power than a similar sized study of adjuvant therapy: with the former there is a direct readout of a patient’s response or resistance to treatment, which is not the case with the latter where patients are free of clinically detectable disease by which to judge response. It is important to note that Ki67 has been validated as an intermediate marker of long-term benefit from endocrine treatment [27] and, in this respect it is a better endpoint than clinical response per se. However, differences in methodology for Ki67 immunohistochemistry and scoring between the two trials precluded combination of Ki67 response data. Likewise, given that different platforms were used for gene expression profiling in the two trials, this study was limited to the largest dataset (FAIMoS trial, n = 47) for the integrative copy number and gene expression analysis.

It should be noted that this study aimed to identify genes that have pathoclinical significance when both amplified and overexpressed. It is notable that there are reverse associations between cyclin-D1 expression with prognosis and resistance to tamoxifen or anastrozole according to whether *CCND1* is amplified or not (overexpression and amplified, poor prognosis; overexpression and non-amplified, good prognosis [54]). Thus the approach taken in this study may not identify genes that associate with clinical phenotype only according to their degree of expression.

## Conclusions

Using a combination of integrative analysis of primary tumors and functional characterization in *in vitro* models, we have provided evidence that 1) copy number profiles can alter in a subset of ER-positive breast cancers in response to AI treatment, and 2) distinct copy number aberrations (such as *CHKA* amplification) can influence the sensitivity of cancer cells to estrogen deprivation, providing evidence to suggest that specific copy-number aberrations may result in resistance to AI therapy. Finally, our study provides a proof of principle that integrative genomic analyses of primary tumors may lead to the identification of novel mechanisms of resistance to specific therapeutic agents.

## Additional files

Additional file 1: Figure S1. Overview of study design with patient numbers for each analysis used to derive the list of genes for functional validation.

Additional file 2: **Supplementary materials and methods: more detail of materials and methods, a workflow and lists of genes, regions and available demographic data.** **Table S1.** Summary of the available clinicopathological details of the samples included in this study from each neoadjuvant trial, together with Ki67 data. **Table S2.** Details of antibodies used for immunoblotting. **Table S3.** Recurrent gains, losses (in >50%) and amplifications (in >2.5%) in 84 estrogen receptor (ER)-positive breast cancer samples. **Table S4.** List of 3,706 copy number-regulated genes derived from a correlation analysis of array comparative genomic hybridization (aCGH) circular binary segmentation (cbs)-smoothed ratios with gene expression profiling data from 47 ER-positive breast cancer samples. **Table S5.** List of 628 genes that are significantly overexpressed when amplified, derived from a gene by gene Wilcoxon analysis of expression data from 47 ER-positive breast cancer samples using aCGH copy number states as a grouping variable. **Table S6.** List of regions and genes whose copy number (cbs-smoothed ratio) is positively **(A)** or negatively **(B)** correlated with the decrease in Ki67 labeling index after 2 weeks of aromatase inhibitor (AI) therapy.

Additional file 3: Figure S2. Deconvolution of CHKA siRNA and shRNA pools. SUM44 cells were transfected with Individual siRNA and shRNA from various manufacturers and the impact on proliferation measured after 6 days. CHKA knockdown was confirmed in each case by quantitative real-time PCR.

Additional file 4: Figure S3. Copy number analysis of SUM44, MDA-MB134-VI, T47D, MCF7 and long-term estrogen deprived (LTED) by quantitative real-time PCR (qRT-PCR) **(A)** and transcript levels of CHKA in the target cell lines used for functional analysis measured by qRT-PCR **(B)**.

## Abbreviations

aCGH: array comparative genomic hybridization
AI: aromatase Inhibitor
ANOVA: analysis of variance
BAC: bacterial artificial chromosome
BSA: bovine serum albumin
CNA: copy number aberration
cbs: circular binary segmentation
DCC: dextran charcoal-stripped
DMEM: Dulbecco's modified Eagle's medium
E2: 17β-estradiol
EGFR: epidermal growth factor receptor
ER: estrogen receptor
HER: human epidermal growth factor receptor
LTED: long-term estrogen deprived
PBS: phosphate-buffered saline
PR: progesterone receptor
qRT-PCR: quantitative real-time PCR
RPMI: Roswell Park Memorial Institute
