# Supplementary material for: Integrative analyses identify modulators of response to neoadjuvant aromatase inhibitors in patients with early breast cancer
Source: Breast Cancer Res. 2015 Mar 11;17(1):35. doi: 10.1186/s13058-015-0532-0 (PMC4406016; doi:10.1186/s13058-015-0532-0)
Supplement: Supplementary file 2 — Supplementary materials and methods: more detail of materials and methods, a workflow and lists of genes, regions and available demographic data. Table S1. Summary of the available clinicopathological details of the samples included in this study from each neoadjuvant trial, together with Ki67 data. Table S2. Details of antibodies used for immunoblotting. Table S3. Recurrent gains, losses (in >50%) and amplifications (in >2.5%) in 84 estrogen receptor (ER)-positive breast cancer samples. Table S4. List of 3,706 copy number-regulated genes derived from a correlation analysis of array comparative genomic hybridization (aCGH) circular binary segmentation (cbs)-smoothed ratios with gene expression profiling data from 47 ER-positive breast cancer samples. Table S5. List of 628 genes that are significantly overexpressed when amplified, derived from a gene by gene Wilcoxon analysis of expression data from 47 ER-positive breast cancer samples using aCGH copy number states as a grouping variable. Table S6. List of regions and genes whose copy number (cbs-smoothed ratio) is positively (A) or negatively (B) correlated with the decrease in Ki67 labeling index after 2 weeks of aromatase inhibitor (AI) therapy. [file 13058_2015_532_MOESM2_ESM.docx]

# LIST OF SUPPLEMENTARY MATERIALS (SUPPORTING ONLINE INFORMATION)

**Supplementary materials and methods.** A more detailed material and methods, a workflow and lists of genes, regions and available demographic data.

**Figure Legends**

**Table S1.** Summary of the available clinicopathological details of the samples included in this study from each neoadjuvant trial, together with Ki67 data.

**Table S2.** Details of antibodies used for immunoblotting.

**Table S3.** Recurrent gains, losses (in >50%) and amplifications (in >2.5%) in 84 ER-positive breast cancer samples.

**Table S4.** List of 3706 copy number-regulated genes derived from a correlation analysis of aCGH cbs-smoothed ratios with gene expression profiling data from 47 ER-positive breast cancer samples.

**Table S5.** List of 628 genes that are significantly overexpressed when amplified, derived from a gene by gene Wilcoxon analysis of expression data from 47 ER-positive breast cancer samples using aCGH copy number states as a grouping variable.

**Table S6.** List of regions and genes whose copy number (cbs-smoothed ratio) is positively (6A) or negatively (6B) correlated with the decrease in Ki67 labelling index after 2 weeks of AI therapy.

# SUPPLEMENTARY Materials and METHODS

**Microarray comparative genomic hybridisation (aCGH)**

After filtering polymorphic BACs and BACs mapping to chromosome Y, a final dataset of clones with unambiguous mapping information according to build hg19 of the human genome (http://www.ensembl.org) was smoothed using the circular binary segmentation (cbs) algorithm [[1](#_ENREF_1), [2](#_ENREF_2)]. A categorical analysis was applied to the BACs after classifying them as representing amplification (>0.45), gain (>0.08 and ≤0.45), loss (<-0.08), or no change according to their cbs-smoothed log2 ratio values [[3](#_ENREF_3), [4](#_ENREF_4)]. Threshold values were determined and validated as previously described [[3](#_ENREF_3), [5](#_ENREF_5)]. Categorical data were subjected to a multi-Fisher’s exact test with adjustment for multiple-testing using the step-down permutation procedure maxT, which provides strong control of the family-wise type I error rate (FWER), as previously described [[3](#_ENREF_3), [6-8](#_ENREF_6)], to identify statistically significant differences between the genomic profiles of pre- and post-AI therapy samples. Unsupervised hierarchical clustering analysis was performed as previously described [[3](#_ENREF_3), [8](#_ENREF_8)]. Briefly, categorical aCGH states (i.e. gains, losses, and amplifications) were used for clustering, employing Ward’s clustering algorithm and correlation distance. Patterns of genome rearrangements were assigned as sawtooth, firestorm or simplex as previously described [[9](#_ENREF_9)].

**Integrative analyses**

To identify genes whose expression levels correlate with copy number changes, cbs-smoothed Log2 ratios from aCGH data were used to assign the aCGH states for each of the genes in the gene expression dataset using the median values for all BACs that overlap with the genomic position of each gene. This resulted in a 1:1 matrix of expression values and aCGH cbs values, which were used for downstream statistical analysis. A Pearson correlation was then performed and a *p* value < 0.05 was considered significant. To define genes that were overexpressed when amplified, a Mann-Whitney U test was performed using categorical aCGH states (that is, amplification versus no amplification) as the grouping variable and the expression of genes as the dependent variable, as previously described [16,29]. *P* values < 0.05 were considered significant.

Comparisons of Ki67 endpoints and copy number states were performed in a similar manner, with an FDR-adjusted Spearman correlation between cbs-smoothed log ratios and Ki67 values. A grouped analysis of Ki67 values was also performed in a gene by gene basis, using aCGH-defined amplification status as a grouping variable. Contiguous regions of significant probes were assigned in each case and plotted as frequency plots with associated LOD scores beneath.

**CHKA silencing validation**

To validate the effect of CHKA silencing on proliferation, a second set of independent siRNAs were tested from Qiagen (GS1119 Qiagen, Manchester, UK) using the same protocol as described in the materials and methods, and a set of shRNAs were used. A set of 4 p.GIPZ plasmids containing shRNAs targeting distinct sequences of CHKA mRNA were purchased from Open Biosystems (RHS4531-EG1119). Agar stabs were streaked on agar plates and single colonies picked for culture and midi-prep using the Nucleobond Xtra midi prep kit (Machery-Nagel), according to manufacturer’s instructions. Plasmids were packaged into Lentiviral particles by co-transfection with packaging plasmids p.PAX2 and p.MD2 into 293T cells using Lipofectamine 2000, according to manufacturer’s instructions. Virus media was harvested on days 2 and 3 post-transfection and used to transduce target cells directly, with the addition of 10µg/µl Polybrene (Millipore). Following two serial infections, media on target cells was refreshed and selection with puromycin commenced 2 days later, for 48-72 hours. GFP expression was used to assess transduction efficiency. Five oligonucleotides targeting distinct sequences within CHKA produced a reduction in proliferation that correlated with a reduction in CHKA transcript levels. These data suggest that the phenotype observed following RNAi-mediated CHKA silencing is unlikely to be due to off target effects (Additional file 4: Figure S3).

**ER/ERE transactivation assays**

To study the effect of RNAi-induced silencing of *CHKA* on ER/ERE transactivation, cells were steroid-stripped for 3 days prior to seeding in DCC medium in 24 well plates. Transfections were performed 24 hours after seeding. The following day medium was exchanged and cells were transfected with 0.25 µg of EREIItkluc (reporter) and 0.25 µg of pCH110 (β-galactosidase for normalization) per well using Fugene 6 according to manufacturer’s instructions. Treatments with steroids or vehicle were performed for 24 hours and luciferase (Promega) and β-galactosidase (Galacton Star, PE Biosystems) activity were measured using a luminometer (TD20/20). Luciferase activity from triplicates or quadruplicates was normalized and expressed relative to vehicle treated siControl.

**Figure S1:** Overview of study design with patient numbers for each analysis used to derive the list of genes for functional validation.

Breast cancer cases from two trials of neoadjuvant aromatase inhibitor (AI) were available for this study (F and E). Sufficient pre-treatment material was available for 84 cases, and DNA from these was subjected to aCGH copy number profiling using a 32K BAC array. RNA of sufficient quality and quantity was available for 47 of 51 cases from the F trial, and was subjected to gene expression profiling using an Illumina WG6 v2.0 bead array. For 19 of the 33 samples from the E trial subjected to aCGH, sufficient material was available for aCGH and matched samples were compared. Reliable Ki67 data was available for 39 of the 84 cases subjected to aCGH, and these data were integrated to define a list of genes associated with a significant decrease in Ki67 expression after 2 weeks of AI treatment. Integration of aCGH and gene expression data for 47 patients allowed the identification of copy number regulated genes (n=3706 genes) and those genes significantly overexpressed when amplified (n=628 genes). These data were integrated with Ki67 data correlation with copy number to generate a list of 9 genes that were amplified, overexpressed when amplified and negatively associated with a significant decrease in Ki67 expression. Of these, 3 genes (CHKA, LRP5 and SAPS3) were identified as overexpressed in a model of AI-resistance (MCF7-LTED cell lines).

**Figure S2:** Validation of CHKA silencing.

Validation of results from SMARTpools using deconvolution of Dharmacon siRNA oligonucleotides, as well as a set of 4 Qiagen siRNAs and a set of 4 shRNAs. Among the siRNAs only 1 overlap in target sequence was identified. shRNAs were used to create a set of SUM44 derivative cell lines with stable knockdown of CHKA prior to assessment of proliferation and CHKA knockdown. Top panels demonstrate the effect of CHKA silencing using individual oligonucleotides on E2-driven proliferation using 1nM E2. For each experiment RNA was extracted and qRTPCR performed (bottom panels) to identify those oligonucleotides that not only reproduced the phenotype observed with SMARTpools, but also reduced the expression of CHKA (red stars denote the 5 oligonucleotides satisfying these criteria).

**Figure S3.** Copy number analysis of SUM44, MDA-MB134-VI, T47D, MCF7 and LTED by qRTPCR.

Copy number was analysed by Taqman copy number assays. The y axis represents copy number and the error bars represent the minimum and maximum copy number per sample. Each bar represents 4 wells.

**Table S1. Summary of the available clinicopathological details of the samples included in this study from each neoadjuvant trial, together with Ki67 data.**

| Case id | Cohort | aCGH? | Pairs? | Expression? | 2 week ΔKi67 (% decrease) | Response classification at 2 weeks | ER | HER2 | PR | Grade | Hicks genomic pattern |
| --- | --- | --- | --- | --- | --- | --- | --- | --- | --- | --- | --- |
| F028 | F | Y | NA | NA | 76.92 | R | 225.70 | 0 | 1 | 2 | sawtooth |
| F219 | F | Y | NA | NA | 87.1 | R | 164.54 | 0 | 1 | 1 | firestorm |
| F020 | F | Y | NA | Y | 31.34 | DN | 233.60 | 0 | 1 | 3 | firestorm |
| F037 | F | Y | NA | Y | -18.26 | DN | 195.40 | 0 | 1 | 2 | sawtooth |
| F039 | F | Y | NA | Y | 5.77 | DN | 10.47 | 0 | 0 | 3 | firestorm |
| F082 | F | Y | NA | Y | -20.4 | DN | 139.70 | 0 | 0 | 2 | sawtooth |
| F169 | F | Y | NA | Y | 24.32 | DN | 175.10 | 0 | 1 | 1 | firestorm |
| F173 | F | Y | NA | Y | 11.28 | DN | 214.56 | 0 | 1 | 2 | firestorm |
| F177 | F | Y | NA | Y | 35.23 | DN | 146.21 | 0 | 1 | 2 | firestorm |
| F201 | F | Y | NA | Y | 0 | DN | 167.33 | 0 | 1 | 2 | firestorm |
| F202 | F | Y | NA | Y | -9.23 | DN | 147.30 | 0 | 0 | 2 | sawtooth |
| F205 | F | Y | NA | Y | -16.88 | DN | 243.43 | 0 | 1 | 3 | firestorm |
| F206 | F | Y | NA | Y | 8.87 | DN | 230.38 | 0 | 1 | 2 | firestorm |
| F036 | F | Y | NA | Y | NA | NA | 251.30 | 0 | 1 | 2 | firestorm |
| F094 | F | Y | NA | Y | NA | NA | NA | 0 | NA | 2 | firestorm |
| F101 | F | Y | NA | Y | NA | NA | 172.02 | 0 | 0 | NA | simplex |
| F126 | F | Y | NA | Y | NA | NA | 240.89 | 0 | 1 | 1 | simplex |
| F174 | F | Y | NA | Y | NA | NA | 199.30 | 0 | 1 | 3 | firestorm |
| F189 | F | Y | NA | Y | NA | NA | 193.29 | 0 | 1 | 3 | simplex |
| F195 | F | Y | NA | Y | NA | NA | 231.08 | 0 | 1 | 2 | simplex |
| F203 | F | Y | NA | Y | NA | NA | 153.18 | 0 | 1 | 2 | sawtooth |
| F002 | F | Y | NA | Y | 67.11 | R | 214.40 | 0 | 0 | 3 | sawtooth |
| F006 | F | Y | NA | Y | 68.14 | R | 226.50 | 0 | 1 | 2 | firestorm |
| F010 | F | Y | NA | Y | 71.15 | R | 287.90 | 0 | 1 | 2 | firestorm |
| F014 | F | Y | NA | Y | 96 | R | 282.20 | 0 | 1 | 3 | firestorm |
| F018 | F | Y | NA | Y | 72.15 | R | 286.30 | 0 | 1 | 2 | firestorm |
| F044 | F | Y | NA | Y | 91.26 | R | 165.88 | 0 | 1 | 3 | firestorm |
| F048 | F | Y | NA | Y | 95.02 | R | 123.56 | 0 | 0 | 3 | firestorm |
| F087 | F | Y | NA | Y | 84.73 | R | 274.78 | 0 | 1 | 2 | sawtooth |
| F100 | F | Y | NA | Y | 88.49 | R | 178.87 | 0 | 1 | 1 | sawtooth |
| F109 | F | Y | NA | Y | 86.24 | R | 227.75 | 0 | 0 | 2 | firestorm |
| F115 | F | Y | NA | Y | 69.73 | R | 154.70 | 0 | 0 | 2 | firestorm |
| F125 | F | Y | NA | Y | 63.89 | R | 221.04 | 0 | 1 | 2 | firestorm |
| F127 | F | Y | NA | Y | 55.77 | R | 243.66 | 0 | 1 | 2 | simplex |
| F165 | F | Y | NA | Y | 93.29 | R | 249.41 | 0 | 1 | 1 | simplex |
| F170 | F | Y | NA | Y | 59.58 | R | 168.88 | 0 | 1 | 3 | firestorm |
| F191 | F | Y | NA | Y | 87.05 | R | 170.65 | 0 | 1 | 2 | firestorm |
| F194 | F | Y | NA | Y | 76.11 | R | 228.36 | 0 | 1 | 1 | simplex |
| F208 | F | Y | NA | Y | 79.23 | R | 225.74 | 0 | 1 | 2 | firestorm |
| F209 | F | Y | NA | Y | 69.32 | R | 200.73 | 0 | 1 | 3 | firestorm |
| F211 | F | Y | NA | Y | 83.55 | R | 217.82 | 0 | 1 | 1 | sawtooth |
| F212 | F | Y | NA | Y | 93.78 | R | 255.21 | 0 | 1 | 1 | firestorm |
| F061 | F | Y | NA | NA | 15.69 | DN | 279.90 | 1 | 0 | 2 | sawtooth |
| F091 | F | Y | NA | Y | 23.08 | DN | 223.30 | 1 | 0 | 2 | firestorm |
| F204 | F | Y | NA | Y | 32.95 | DN | 216.52 | 1 | 0 | 3 | firestorm |
| F128 | F | Y | NA | Y | NA | NA | 187.86 | 1 | 0 | 3 | firestorm |
| F132 | F | Y | NA | Y | NA | NA | 164.54 | 1 | 0 | 2 | firestorm |
| F171 | F | Y | NA | NA | NA | NA | NA | NA | NA | 3 | sawtooth |
| F185 | F | Y | NA | Y | NA | NA | 215.03 | 1 | 1 | 2 | firestorm |
| F021 | F | Y | NA | Y | 88.36 | R | 222.90 | 1 | 0 | 1 | sawtooth |
| F187 | F | Y | NA | Y | 94.26 | R | 219.74 | 1 | 1 | 3 | firestorm |
| E017 | E | Y | NA | NA | 38.33 | DN | NA | NA | NA | NA | simplex |
| E087 | E | Y | NA | NA | -22.12 | DN | NA | NA | NA | NA | sawtooth |
| E021 | E | Y | NA | NA | NA | NA | NA | NA | NA | NA | sawtooth |
| E056 | E | Y | NA | NA | 33.54 | NA | NA | NA | NA | NA | simplex |
| E062 | E | Y | NA | NA | NA | NA | NA | NA | NA | NA | simplex |
| E070 | E | Y | NA | NA | NA | NA | NA | NA | NA | NA | firestorm |
| E072 | E | Y | NA | NA | NA | NA | NA | NA | NA | NA | simplex |
| E082 | E | Y | NA | NA | NA | NA | NA | NA | NA | NA | firestorm |
| E007 | E | Y | NA | NA | 84.87 | R | NA | NA | NA | NA | sawtooth |
| E026 | E | Y | NA | NA | 92.55 | R | NA | NA | NA | NA | simplex |
| E061 | E | Y | NA | NA | 100.00 | R | NA | NA | NA | NA | sawtooth |
| E078 | E | Y | NA | NA | 75.51 | R | NA | NA | NA | NA | simplex |
| E116 | E | Y | NA | NA | 75.03 | R | NA | NA | NA | NA | firestorm |
| E132 | E | Y | NA | NA | 89.80 | R | NA | NA | NA | NA | firestorm |
| E109 | E | Y | Y | NA | 28.00 | DN | NA | NA | NA | NA | firestorm |
| E125 | E | Y | Y | NA | 16.85 | DN | NA | NA | NA | NA | firestorm |
| E086 | E | Y | Y | NA | NA | NA | NA | NA | NA | NA | sawtooth |
| E103 | E | Y | Y | NA | NA | NA | NA | NA | NA | NA | sawtooth |
| E106 | E | Y | Y | NA | NA | NA | NA | NA | NA | NA | firestorm |
| E114 | E | Y | Y | NA | NA | NA | NA | NA | NA | NA | firestorm |
| E027 | E | Y | Y | NA | 64.91 | R | NA | NA | NA | NA | sawtooth |
| E035 | E | Y | Y | NA | 75.36 | R | NA | NA | NA | NA | firestorm |
| E038 | E | Y | Y | NA | 72.56 | R | NA | NA | NA | NA | firestorm |
| E050 | E | Y | Y | NA | 82.34 | R | NA | NA | NA | NA | firestorm |
| E067 | E | Y | Y | NA | 96.87 | R | NA | NA | NA | NA | sawtooth |
| E069 | E | Y | Y | NA | 70.86 | R | NA | NA | NA | NA | sawtooth |
| E077 | E | Y | Y | NA | 71.31 | R | NA | NA | NA | NA | sawtooth |
| E090 | E | Y | Y | NA | 56.11 | R | NA | NA | NA | NA | firestorm |
| E100 | E | Y | Y | NA | 100.00 | R | NA | NA | NA | NA | simplex |
| E117 | E | Y | Y | NA | 94.26 | R | NA | NA | NA | NA | firestorm |
| E118 | E | Y | Y | NA | 84.87 | R | NA | NA | NA | NA | firestorm |
| E119 | E | Y | Y | NA | 91.90 | R | NA | NA | NA | NA | firestorm |
| E136 | E | Y | Y | NA | 90.04 | R | NA | NA | NA | NA | simplex |

F - FAIMoS, E - Edinburgh, Y - yes, NA - not available, R - Responder, DN - De Novo Resistance, ER - oestrogen receptor, PR -

**Table S2. Details of antibodies used for immunoblotting.**

| **Target protein** | **Antibody clone** | **Host** | **Dilution** | **Predicted MW (kDa)** | **Source** |
| --- | --- | --- | --- | --- | --- |
| Akt | Polyclonal | Rabbit | 1:1000 | 60 | Cell Signalling Technologies (#9272) |
| pAkt [Ser473] | Polyclonal | Rabbit | 1:1000 | 60 | Cell Signalling Technologies (#9271) |
| pAKT [Thr308] | Polyclonal | Rabbit | 1:1000 | 60 | Cell Signalling Technologies (#9275) |
| α-tubulin | DM1A | Mouse | 1:500 | 55 | Sigma (T9026) |
| Choline Kinase A | Polyclonal | Rabbit | 1:1000 | 52 | Abcam (ab88053) |
| Cyclin D1 | Polyclonal | Rabbit | 1:1000 | 36 | Cell Signalling Technologies (#2922) |
| Erα | F-10 | Mouse | 1:1000 | 68 | Santa Cruz Biotech (Sc-8002) |
| pERα [Ser167] | D1A3 | Rabbit | 1:1000 | 68 | Cell Signalling Technologies (#5587) |
| LRP5 | D80F2 | Rabbit | 1:1000 | 200 | Cell Signalling Technologies (#5731) |
| pp90RSK [Ser380] | Polyclonal | Rabbit | 1:1000 | 90 | Cell Signalling Technologies (#9341) |
| pRB | Monoclonal | Rabbit | 1:1000 | 110 | Cell Signalling Technologies (#8516) |
| SAPS3 | Polyclonal | Rabbit | 1:2500 | 98 | Abcam (ab72034) |
| S6K | 5G10 | Rabbit | 1:1000 | 32 | Cell Signalling Technologies (#2217) |
| pS6K [Ser240/244] | 61H9 | Rabbit | 1:1000 | 32 | Cell Signalling Technologies (#4838) |

**Table S3. Recurrent gains, losses (in >50%) and amplifications (in >2.5%) in 84 ER-positive breast cancers.**

Gains (Threshold 0.08)

| **Chromosome** | **Cytobands** | **Start** | **End** | **BACs** | **Length MB** | **maxM** | **Max overlap** |
| --- | --- | --- | --- | --- | --- | --- | --- |
| 1 | p12 | 120449470 | 120650514 | 4 | 0.20 | 1.37 | 46 |
| 1 | q21.1-q21.2 | 143510356 | 149753920 | 48 | 6.24 | 1.49 | 54 |
| 1 | q21.3 | 151376896 | 153503482 | 22 | 2.13 | 1.20 | 53 |
| 1 | q21.3 | 154414666 | 154932673 | 4 | 0.52 | 0.83 | 44 |
| 1 | q23.1-q44 | 156919444 | 248876423 | 957 | 91.96 | 2.55 | 70 |
| 2 | p11.1-q11.1 | 90230658 | 91940831 | 13 | 1.71 | 0.56 | 47 |
| 2 | q11.1 | 92245958 | 92315522 | 3 | 0.07 | 0.49 | 48 |
| 7 | q11.1-q11.21 | 61058297 | 62149667 | 6 | 1.09 | 0.97 | 58 |
| 8 | q21.11-q21.12 | 76882768 | 78474313 | 21 | 1.59 | 2.00 | 45 |
| 8 | q21.2-q24.13 | 86371819 | 123907596 | 368 | 37.54 | 2.87 | 53 |
| 8 | q24.21 | 127694062 | 128571966 | 7 | 0.88 | 1.60 | 42 |
| 8 | q24.21 | 129790235 | 130059243 | 3 | 0.27 | 1.79 | 42 |
| 8 | q24.22 | 135379112 | 135868800 | 6 | 0.49 | 1.44 | 42 |
| 8 | q24.23 | 136402287 | 139126068 | 31 | 2.72 | 1.02 | 48 |
| 12 | q11-q12 | 38016955 | 38447050 | 5 | 0.43 | 0.41 | 43 |
| 19 | q11 | 27733070 | 28204598 | 6 | 0.47 | 0.42 | 58 |

Losses (Threshold -0.08)

| **Chromosome** | **Cytobands** | **Start** | **End** | **BACs** | **Length MB** | **maxM** | **Max overlap** |
| --- | --- | --- | --- | --- | --- | --- | --- |
| 1 | p36.33-p36.12 | 1633798 | 23563019 | 228 | 21.93 | -0.77 | 54 |
| 1 | p36.11 | 24190016 | 24522112 | 4 | 0.33 | -0.45 | 42 |
| 1 | p36.11-p35.3 | 24905319 | 29252730 | 53 | 4.35 | -0.70 | 46 |
| 1 | p35.2 | 30219417 | 31891189 | 16 | 1.67 | -0.74 | 42 |
| 2 | q14.2 | 121325546 | 121916458 | 5 | 0.59 | -0.52 | 42 |
| 7 | q22.1 | 101018742 | 101376032 | 3 | 0.36 | -0.42 | 42 |
| 8 | p23.3 | 475607 | 1657335 | 13 | 1.18 | -0.93 | 45 |
| 8 | p23.1 | 6597345 | 7854756 | 8 | 1.26 | -1.05 | 46 |
| 8 | p23.1 | 7972660 | 12685657 | 37 | 4.71 | -0.82 | 47 |
| 8 | p21.3-p21.2 | 19484318 | 23932973 | 48 | 4.45 | -0.68 | 46 |
| 8 | p21.2 | 24373349 | 25209076 | 6 | 0.84 | -0.54 | 42 |
| 8 | p21.2 | 25744703 | 26498605 | 9 | 0.75 | -0.59 | 43 |
| 9 | q34.2-q34.3 | 136583961 | 138450575 | 21 | 1.87 | -0.63 | 43 |
| 11 | q22.3-q25 | 107294539 | 134927296 | 278 | 27.63 | -1.50 | 62 |
| 16 | q12.1 | 48900991 | 51173060 | 22 | 2.27 | -0.82 | 45 |
| 16 | q12.1 | 51295586 | 51764554 | 10 | 0.47 | -0.77 | 43 |
| 16 | q12.1-q12.2 | 52365724 | 53111591 | 11 | 0.75 | -0.72 | 43 |
| 16 | q12.2-q21 | 53377747 | 60376380 | 97 | 7.00 | -0.95 | 55 |
| 16 | q21-q22.1 | 60715507 | 69909978 | 96 | 9.19 | -0.90 | 60 |
| 16 | q22.2-q24.3 | 71230692 | 90044587 | 279 | 18.81 | -0.86 | 59 |
| 17 | p13.3-p11.2 | 1002 | 19490093 | 211 | 19.49 | -0.66 | 55 |
| 17 | p11.2 | 20246028 | 20506788 | 5 | 0.26 | -0.64 | 44 |
| 19 | p13.2 | 8769145 | 8963694 | 10 | 0.19 | -1.32 | 45 |
| 19 | q13.32-q13.33 | 45202080 | 48204670 | 35 | 3.00 | -0.41 | 44 |
| 19 | q13.33 | 48552987 | 50392555 | 30 | 1.84 | -0.42 | 44 |
| 22 | q12.3-q13.1 | 36548602 | 40131998 | 36 | 3.58 | -0.67 | 44 |
| 22 | q13.2-q13.33 | 41520291 | 50425673 | 86 | 8.91 | -0.85 | 45 |

Amplifications (Threshold >0.45)

| **Chromosome** | **Cytobands** | **Start** | **End** | **BACs** | **Length MB** | **maxM** | **Max overlap** |
| --- | --- | --- | --- | --- | --- | --- | --- |
| 1 | p34.2 | 41884252 | 43355031 | 12 | 1.47 | 0.95 | 2 |
| 1 | p32.1 | 60159577 | 60522849 | 4 | 0.36 | 1.32 | 2 |
| 1 | p31.3 | 67093089 | 67498359 | 5 | 0.41 | 1.72 | 2 |
| 1 | p31.3-p31.1 | 68687688 | 70024173 | 13 | 1.34 | 1.64 | 2 |
| 1 | p31.1 | 70490587 | 70957718 | 4 | 0.47 | 1.56 | 2 |
| 1 | p21.1 | 104025830 | 104426934 | 3 | 0.40 | 0.62 | 2 |
| 1 | p13.2-p13.1 | 115265167 | 116555334 | 11 | 1.29 | 1.72 | 4 |
| 1 | p12-p11.2 | 118853231 | 121345350 | 22 | 2.49 | 1.51 | 11 |
| 1 | q21.1-q21.3 | 143510356 | 153837214 | 90 | 10.33 | 1.68 | 12 |
| 1 | q21.3-q44 | 154068207 | 248876423 | 987 | 94.81 | 2.55 | 21 |
| 3 | q11.2-q12.1 | 97633846 | 99372723 | 16 | 1.74 | 0.74 | 2 |
| 3 | q12.3-q13.11 | 101488021 | 102878204 | 15 | 1.39 | 0.75 | 2 |
| 3 | q13.11-q13.13 | 105071893 | 108780739 | 64 | 3.71 | 1.24 | 3 |
| 3 | q13.13-q13.31 | 109221593 | 115405024 | 91 | 6.18 | 0.66 | 2 |
| 3 | q26.1 | 165695522 | 166202719 | 6 | 0.51 | 0.75 | 2 |
| 3 | q26.2-q26.31 | 167689814 | 172181958 | 41 | 4.49 | 1.19 | 3 |
| 3 | q26.31-q26.32 | 174627861 | 175873389 | 15 | 1.25 | 0.77 | 2 |
| 3 | q26.32-q26.33 | 177812622 | 179149667 | 15 | 1.34 | 1.28 | 2 |
| 3 | q27.1-q27.2 | 184340926 | 185066257 | 8 | 0.73 | 0.93 | 2 |
| 3 | q28-q29 | 189818252 | 193150220 | 33 | 3.33 | 0.81 | 3 |
| 3 | q29 | 196649601 | 197036560 | 6 | 0.39 | 0.81 | 2 |
| 5 | q13.2 | 68855238 | 70144801 | 11 | 1.29 | 0.74 | 2 |
| 6 | p12.1 | 53194213 | 55890130 | 34 | 2.70 | 0.77 | 2 |
| 6 | q12 | 66519140 | 67578288 | 10 | 1.06 | 0.88 | 2 |
| 6 | q16.1 | 96095998 | 96915216 | 6 | 0.82 | 1.79 | 2 |
| 6 | q16.3 | 103099590 | 103422398 | 5 | 0.32 | 1.36 | 2 |
| 6 | q21 | 106793765 | 109891017 | 35 | 3.10 | 1.64 | 4 |
| 6 | q24.2 | 142971134 | 143947060 | 10 | 0.98 | 0.84 | 2 |
| 7 | q11.1-q11.21 | 61058297 | 62159280 | 7 | 1.10 | 0.97 | 8 |
| 8 | p23.1 | 7048666 | 7854756 | 4 | 0.81 | 0.85 | 2 |
| 8 | p12-q24.3 | 34169179 | 144008274 | 1064 | 109.84 | 2.87 | 21 |
| 9 | q33.1 | 121016293 | 121345942 | 3 | 0.33 | 0.92 | 2 |
| 9 | q33.3-q34.11 | 129041210 | 130504357 | 16 | 1.46 | 1.36 | 3 |
| 10 | p14 | 8689042 | 11345917 | 25 | 2.66 | 0.95 | 2 |
| 10 | q26.3 | 134958100 | 135056216 | 3 | 0.10 | 1.31 | 2 |
| 11 | p15.3 | 10773659 | 11336903 | 5 | 0.56 | 0.66 | 2 |
| 11 | p13 | 31654797 | 32187106 | 5 | 0.53 | 1.58 | 2 |
| 11 | p13 | 32507182 | 36102748 | 42 | 3.60 | 1.04 | 5 |
| 11 | p12 | 37791454 | 38447352 | 7 | 0.66 | 1.33 | 2 |
| 11 | q13.2 | 66252208 | 67630930 | 13 | 1.38 | 1.10 | 4 |
| 11 | q13.2-q13.4 | 67755774 | 73938461 | 65 | 6.18 | 2.50 | 18 |
| 11 | q13.4 | 74330120 | 74950484 | 6 | 0.62 | 1.24 | 2 |
| 11 | q13.5-q14.2 | 76033726 | 85602981 | 85 | 9.57 | 1.98 | 8 |
| 11 | q21 | 93225368 | 94989575 | 17 | 1.76 | 1.80 | 2 |
| 12 | p12.2-p12.1 | 21170043 | 23286925 | 21 | 2.12 | 1.17 | 2 |
| 12 | p12.1 | 23803151 | 25398008 | 15 | 1.59 | 0.87 | 2 |
| 12 | p11.23 | 26800755 | 27129877 | 3 | 0.33 | 0.65 | 2 |
| 12 | q14.2-q14.3 | 64875176 | 65737940 | 9 | 0.86 | 1.53 | 2 |
| 12 | q14.3 | 66359700 | 66816271 | 8 | 0.46 | 1.00 | 2 |
| 12 | q14.3-q21.1 | 67537800 | 72323650 | 59 | 4.79 | 2.52 | 2 |
| 12 | q21.1 | 73529034 | 74862855 | 13 | 1.33 | 1.51 | 2 |
| 12 | q21.2-q21.31 | 80057369 | 81563257 | 13 | 1.51 | 0.73 | 2 |
| 12 | q21.31 | 81757111 | 82151688 | 3 | 0.39 | 0.51 | 2 |
| 12 | q21.31 | 82862494 | 84824140 | 21 | 1.96 | 1.36 | 2 |
| 13 | q12.3 | 31275891 | 31951813 | 5 | 0.68 | 0.83 | 2 |
| 14 | q11.2 | 19968167 | 20524999 | 6 | 0.56 | 0.79 | 4 |
| 15 | q11.2 | 20828305 | 22568791 | 17 | 1.74 | 0.81 | 4 |
| 15 | q26.2 | 96438188 | 97083841 | 5 | 0.65 | 0.88 | 2 |
| 15 | q26.3 | 98560308 | 99978982 | 17 | 1.42 | 1.75 | 2 |
| 16 | p13.3 | 865893 | 1760582 | 10 | 0.89 | 0.71 | 2 |
| 16 | p13.3-p11.2 | 5433882 | 30682232 | 259 | 25.25 | 1.31 | 5 |
| 16 | p11.2-p11.1 | 31442988 | 33784671 | 20 | 2.34 | 0.87 | 4 |
| 16 | q11.1-q12.1 | 34501228 | 49061821 | 33 | 14.56 | 0.88 | 3 |
| 16 | q12.1-q12.2 | 50348570 | 52875697 | 38 | 2.53 | 0.81 | 2 |
| 16 | q22.1-q22.2 | 70780902 | 71315829 | 7 | 0.53 | 1.76 | 3 |
| 17 | q11.1-q11.2 | 25264007 | 28632216 | 29 | 3.37 | 1.47 | 4 |
| 17 | q11.2 | 28721395 | 29001730 | 3 | 0.28 | 1.04 | 2 |
| 17 | q12 | 34109006 | 34913630 | 7 | 0.80 | 2.34 | 2 |
| 17 | q12-q21.2 | 36034611 | 38579643 | 28 | 2.55 | 3.43 | 8 |
| 17 | q21.32-q24.2 | 45932091 | 66918192 | 268 | 20.99 | 2.18 | 9 |
| 18 | p11.21 | 12692485 | 13652492 | 10 | 0.96 | 1.05 | 2 |
| 19 | p13.2 | 8769145 | 8963694 | 10 | 0.19 | 0.68 | 2 |
| 20 | q11.23-q12 | 37455936 | 38574495 | 10 | 1.12 | 0.99 | 2 |
| 20 | q12-q13.12 | 38928401 | 44335575 | 60 | 5.41 | 1.45 | 4 |
| 20 | q13.12-q13.2 | 45213999 | 50309885 | 58 | 5.10 | 1.30 | 4 |
| 20 | q13.2-q13.31 | 50797877 | 55765737 | 58 | 4.97 | 2.38 | 6 |
| 20 | q13.31-q13.32 | 56162747 | 58181249 | 20 | 2.02 | 1.88 | 4 |
| 20 | q13.33 | 59042975 | 62250093 | 35 | 3.21 | 1.30 | 3 |
| 22 | q11.1 | 16061103 | 16505264 | 13 | 0.44 | 0.72 | 2 |

**Table S4. List of 3706 copy number-regulated genes derived from a correlation analysis of aCGH cbs-smoothed ratios with gene expression profiling data from 47 ER-positive breast cancer samples.**

| **Symbol** | **Chromosome** | **Cytoband** | **pearson.p** | **pearson.adjp** | **pearson.cor** | **gain.fold** | **loss.fold** | **amp.fold** |
| --- | --- | --- | --- | --- | --- | --- | --- | --- |
| MAN1A2 | 1 | p12 | 1.02E-07 | 1.09E-05 | 0.69 | 1.26 | 0.80 | 2.85 |
| GDAP2 | 1 | p12 | 1.24E-05 | 4.34E-04 | 0.59 | 1.18 | 0.87 | 1.90 |
| NOTCH2 | 1 | p12 | 2.79E-05 | 8.02E-04 | 0.57 | 1.04 |  | 1.68 |
| WDR3 | 1 | p12 | 9.66E-05 | 2.08E-03 | 0.54 | 1.12 | 0.96 | 1.37 |
| HAO2 | 1 | p12 | 5.34E-03 | 4.09E-02 | 0.40 | 1.04 | 0.96 | 1.16 |
| TTF2 | 1 | p13.1 | 1.04E-07 | 1.10E-05 | 0.69 | 1.77 | 0.79 | 2.93 |
| CD58 | 1 | p13.1 | 1.09E-06 | 6.76E-05 | 0.64 | 1.46 | 0.76 | 2.48 |
| TRIM45 | 1 | p13.1 | 1.09E-05 | 3.93E-04 | 0.59 | 1.40 | 0.80 | 4.84 |
| SLC22A15 | 1 | p13.1 | 5.59E-05 | 1.37E-03 | 0.55 | 1.11 | 0.93 | 2.00 |
| PTGFRN | 1 | p13.1 | 8.76E-05 | 1.93E-03 | 0.54 | 1.19 | 0.96 | 3.23 |
| ATP1A1 | 1 | p13.1 | 3.49E-04 | 5.52E-03 | 0.50 | 1.48 | 0.84 | 4.09 |
| VANGL1 | 1 | p13.1 | 4.48E-04 | 6.71E-03 | 0.49 | 1.12 | 1.01 | 1.28 |
| IGSF3 | 1 | p13.1 | 3.13E-03 | 2.79E-02 | 0.42 | 1.70 | 1.01 | 5.15 |
| TRIM33 | 1 | p13.2 | 3.01E-11 | 1.56E-08 | 0.79 | 1.49 | 0.72 | 3.96 |
| RSBN1 | 1 | p13.2 | 6.04E-07 | 4.28E-05 | 0.65 | 1.36 | 0.70 |  |
| ATP5F1 | 1 | p13.2 | 9.36E-07 | 6.03E-05 | 0.65 | 1.27 | 0.66 |  |
| AP4B1 | 1 | p13.2 | 1.46E-06 | 8.53E-05 | 0.64 | 1.49 | 0.73 |  |
| NRAS | 1 | p13.2 | 1.81E-05 | 5.69E-04 | 0.58 | 1.23 | 0.86 | 2.70 |
| CTTNBP2NL | 1 | p13.2 | 7.78E-05 | 1.78E-03 | 0.54 | 1.08 | 0.90 |  |
| LRIG2 | 1 | p13.2 | 1.75E-04 | 3.25E-03 | 0.52 | 1.22 | 0.88 |  |
| RAP1A | 1 | p13.2 | 3.28E-04 | 5.28E-03 | 0.50 | 1.05 | 0.92 |  |
| RHOC | 1 | p13.2 | 4.39E-04 | 6.60E-03 | 0.49 | 1.53 | 0.85 |  |
| MOV10 | 1 | p13.2 | 7.73E-04 | 9.99E-03 | 0.47 | 1.55 | 0.86 |  |
| DCLRE1B | 1 | p13.2 | 1.90E-03 | 1.96E-02 | 0.44 | 1.11 | 0.93 |  |
| ST7L | 1 | p13.2 | 3.05E-03 | 2.74E-02 | 0.42 | 1.10 | 0.91 |  |
| PPM1J | 1 | p13.2 | 3.39E-03 | 2.94E-02 | 0.42 | 1.74 | 0.86 |  |
| SARS | 1 | p13.3 | 4.36E-08 | 5.54E-06 | 0.70 | 1.16 | 0.77 |  |
| PSMA5 | 1 | p13.3 | 5.60E-06 | 2.35E-04 | 0.61 | 1.36 | 0.74 |  |
| FAM40A | 1 | p13.3 | 1.94E-05 | 6.00E-04 | 0.58 | 1.29 | 0.86 | 1.86 |
| PSRC1 | 1 | p13.3 | 1.40E-04 | 2.74E-03 | 0.53 | 1.42 | 0.77 |  |
| CEPT1 | 1 | p13.3 | 1.44E-04 | 2.81E-03 | 0.53 | 1.18 | 0.76 |  |
| GSTM4 | 1 | p13.3 | 6.68E-04 | 8.98E-03 | 0.48 | 1.42 | 0.84 |  |
| AHCYL1 | 1 | p13.3 | 7.75E-04 | 1.00E-02 | 0.47 | 1.42 | 0.82 | 2.82 |
| TMEM167B | 1 | p13.3 | 8.47E-04 | 1.06E-02 | 0.47 | 1.13 | 0.82 |  |
| GNAI3 | 1 | p13.3 | 8.82E-04 | 1.10E-02 | 0.47 | 1.30 | 0.72 |  |
| DENND2D | 1 | p13.3 | 2.84E-03 | 2.60E-02 | 0.43 | 1.11 | 0.74 |  |
| STXBP3 | 1 | p13.3 | 3.14E-03 | 2.80E-02 | 0.42 | 1.13 | 0.74 |  |
| SLC25A24 | 1 | p13.3 | 3.91E-03 | 3.27E-02 | 0.41 | 1.19 | 0.93 |  |
| CLCC1 | 1 | p13.3 | 4.44E-03 | 3.58E-02 | 0.41 | 1.16 | 0.85 |  |
| DPH5 | 1 | p21.2 | 9.79E-06 | 3.63E-04 | 0.60 | 1.28 | 0.72 | 2.21 |
| DBT | 1 | p21.2 | 1.45E-05 | 4.86E-04 | 0.59 | 1.61 | 0.76 |  |
| CCDC76 | 1 | p21.2 | 1.90E-05 | 5.91E-04 | 0.58 | 1.23 | 0.76 |  |
| RTCD1 | 1 | p21.2 | 3.51E-04 | 5.54E-03 | 0.50 | 1.17 | 0.72 |  |
| HIAT1 | 1 | p21.2 | 5.20E-04 | 7.50E-03 | 0.49 | 1.08 | 0.76 |  |
| SLC35A3 | 1 | p21.2 | 5.59E-03 | 4.22E-02 | 0.40 | 1.55 | 0.72 |  |
| ALG14 | 1 | p21.3 | 6.42E-03 | 4.67E-02 | 0.39 | 1.23 | 0.85 |  |
| GLMN | 1 | p22.1 | 6.79E-09 | 1.30E-06 | 0.73 | 1.86 | 0.73 | 3.97 |
| BRDT | 1 | p22.1 | 7.94E-09 | 1.48E-06 | 0.73 | 1.55 | 0.90 | 3.24 |
| KIAA1107 | 1 | p22.1 | 2.54E-08 | 3.67E-06 | 0.71 | 1.35 | 0.93 | 2.63 |
| RPAP2 | 1 | p22.1 | 2.40E-07 | 2.14E-05 | 0.67 | 1.59 | 0.79 | 3.39 |
| ABHD7 | 1 | p22.1 | 6.20E-07 | 4.36E-05 | 0.65 | 2.46 | 0.77 | 9.70 |
| RPL5 | 1 | p22.1 | 3.71E-05 | 9.98E-04 | 0.56 | 1.19 | 0.83 |  |
| TMED5 | 1 | p22.1 | 2.28E-03 | 2.22E-02 | 0.43 | 0.97 | 0.71 |  |
| BTBD8 | 1 | p22.1 | 3.37E-03 | 2.93E-02 | 0.42 | 1.09 | 1.00 | 1.38 |
| ZNF644 | 1 | p22.2 | 1.69E-11 | 1.01E-08 | 0.80 | 1.79 | 0.84 | 3.13 |
| GTF2B | 1 | p22.2 | 3.00E-08 | 4.19E-06 | 0.71 | 1.42 | 0.72 | 1.74 |
| CCBL2 | 1 | p22.2 | 9.80E-06 | 3.63E-04 | 0.60 | 1.36 | 0.70 | 3.19 |
| PKN2 | 1 | p22.2 | 1.56E-05 | 5.15E-04 | 0.59 | 1.38 | 0.72 | 2.50 |
| CDC7 | 1 | p22.2 | 1.83E-05 | 5.74E-04 | 0.58 | 1.56 | 0.82 | 2.66 |
| GBP2 | 1 | p22.2 | 7.03E-04 | 9.30E-03 | 0.48 | 1.38 | 0.58 | 1.69 |
| LRRC8B | 1 | p22.2 | 6.99E-03 | 4.96E-02 | 0.39 | 1.33 | 0.91 | 2.05 |
| BCL10 | 1 | p22.3 | 1.20E-03 | 1.38E-02 | 0.46 | 1.03 | 0.84 |  |
| ZNHIT6 | 1 | p22.3 | 2.32E-03 | 2.25E-02 | 0.43 | 1.00 | 0.83 |  |
| DDAH1 | 1 | p22.3 | 2.48E-03 | 2.36E-02 | 0.43 | 1.02 | 0.70 |  |
| GNG5 | 1 | p22.3 | 2.58E-03 | 2.42E-02 | 0.43 | 1.12 | 0.79 |  |
| RP4-604K5.1 | 1 | p22.3 | 2.87E-03 | 2.63E-02 | 0.43 | 1.02 | 0.66 |  |
| BXDC5 | 1 | p22.3 | 4.71E-03 | 3.72E-02 | 0.41 | 0.95 | 0.76 |  |
| CTBS | 1 | p22.3 | 5.43E-03 | 4.14E-02 | 0.40 | 1.31 | 0.75 |  |
| RABGGTB | 1 | p31.1 | 1.93E-09 | 4.47E-07 | 0.74 | 1.23 | 0.63 |  |
| HHLA3 | 1 | p31.1 | 5.03E-08 | 6.13E-06 | 0.70 | 1.26 | 0.92 | 2.37 |
| ANKRD13C | 1 | p31.1 | 5.69E-06 | 2.37E-04 | 0.61 | 1.06 | 0.88 | 1.73 |
| ZRANB2 | 1 | p31.1 | 6.64E-05 | 1.56E-03 | 0.55 | 1.51 | 0.72 |  |
| USP33 | 1 | p31.1 | 1.90E-03 | 1.95E-02 | 0.44 | 1.12 | 0.78 |  |
| TYW3 | 1 | p31.1 | 1.91E-03 | 1.96E-02 | 0.44 | 1.29 | 0.75 |  |
| LRRC7 | 1 | p31.1 | 5.30E-03 | 4.07E-02 | 0.40 | 1.03 | 0.99 | 1.22 |
| MIER1 | 1 | p31.3 | 7.11E-12 | 5.19E-09 | 0.81 | 1.55 | 0.74 | 3.99 |
| SLC35D1 | 1 | p31.3 | 4.75E-06 | 2.05E-04 | 0.61 | 1.26 | 0.92 | 1.99 |
| WDR78 | 1 | p31.3 | 1.75E-05 | 5.53E-04 | 0.58 | 1.10 | 0.97 | 1.56 |
| LEPROT | 1 | p31.3 | 4.89E-04 | 7.17E-03 | 0.49 | 1.53 | 0.74 |  |
| GADD45A | 1 | p31.3 | 1.58E-03 | 1.69E-02 | 0.45 | 1.24 | 0.75 |  |
| DOCK7 | 1 | p31.3 | 2.13E-03 | 2.12E-02 | 0.44 | 1.11 | 0.77 |  |
| AK3L1 | 1 | p31.3 | 2.77E-03 | 2.56E-02 | 0.43 | 1.46 | 0.78 |  |
| INADL | 1 | p31.3 | 4.28E-03 | 3.48E-02 | 0.41 | 1.15 | 0.82 |  |
| ALG6 | 1 | p31.3 | 5.33E-03 | 4.09E-02 | 0.40 | 1.06 | 0.84 |  |
| ATG4C | 1 | p31.3 | 5.65E-03 | 4.26E-02 | 0.40 | 1.00 | 0.84 |  |
| HOOK1 | 1 | p32.1 | 9.20E-04 | 1.13E-02 | 0.47 | 2.13 | 0.72 |  |
| MRPL37 | 1 | p32.3 | 4.06E-08 | 5.21E-06 | 0.70 | 1.81 | 0.73 |  |
| FAF1 | 1 | p32.3 | 2.13E-07 | 1.95E-05 | 0.67 | 3.27 | 0.76 |  |
| C1orf175 | 1 | p32.3 | 3.13E-06 | 1.48E-04 | 0.62 | 1.65 | 0.82 |  |
| USP24 | 1 | p32.3 | 6.19E-06 | 2.53E-04 | 0.61 | 1.53 | 0.81 |  |
| C1orf163 | 1 | p32.3 | 8.05E-06 | 3.14E-04 | 0.60 |  | 0.83 |  |
| PRPF38A | 1 | p32.3 | 8.49E-06 | 3.25E-04 | 0.60 |  | 0.78 |  |
| SSBP3 | 1 | p32.3 | 1.02E-05 | 3.75E-04 | 0.60 | 1.65 | 0.83 |  |
| LRRC42 | 1 | p32.3 | 7.67E-05 | 1.76E-03 | 0.54 | 1.22 | 0.80 |  |
| NRD1 | 1 | p32.3 | 1.03E-04 | 2.17E-03 | 0.54 |  | 0.80 |  |
| RNF11 | 1 | p32.3 | 1.08E-04 | 2.27E-03 | 0.53 | 1.73 | 0.83 |  |
| PARS2 | 1 | p32.3 | 1.18E-04 | 2.43E-03 | 0.53 | 1.15 | 0.91 |  |
| C1orf123 | 1 | p32.3 | 2.24E-04 | 3.94E-03 | 0.51 |  | 0.85 |  |
| TXNDC12 | 1 | p32.3 | 3.35E-04 | 5.36E-03 | 0.50 |  | 0.86 |  |
| TMEM48 | 1 | p32.3 | 3.67E-04 | 5.74E-03 | 0.50 | 2.20 | 0.86 |  |
| YIPF1 | 1 | p32.3 | 4.94E-04 | 7.22E-03 | 0.49 | 1.52 | 0.78 |  |
| LRP8 | 1 | p32.3 | 1.90E-03 | 1.96E-02 | 0.44 | 1.46 | 0.91 |  |
| MAGOH | 1 | p32.3 | 3.84E-03 | 3.23E-02 | 0.41 | 1.85 | 0.75 |  |
| TMEM59 | 1 | p32.3 | 5.72E-03 | 4.30E-02 | 0.40 | 1.11 | 0.83 |  |
| KIAA0494 | 1 | p33 | 5.35E-04 | 7.65E-03 | 0.49 | 1.42 | 0.78 |  |
| FAAH | 1 | p33 | 4.58E-03 | 3.65E-02 | 0.41 | 1.69 | 0.74 |  |
| POMGNT1 | 1 | p34.1 | 2.56E-08 | 3.68E-06 | 0.71 | 1.85 | 0.68 |  |
| UROD | 1 | p34.1 | 1.91E-07 | 1.80E-05 | 0.68 | 1.58 | 0.82 |  |
| DMAP1 | 1 | p34.1 | 3.14E-07 | 2.67E-05 | 0.67 | 2.00 | 0.72 |  |
| DPH2 | 1 | p34.1 | 5.94E-07 | 4.25E-05 | 0.65 | 1.75 | 0.73 |  |
| ERI3 | 1 | p34.1 | 2.56E-06 | 1.28E-04 | 0.63 | 1.91 | 0.72 |  |
| AKR1A1 | 1 | p34.1 | 7.45E-06 | 2.95E-04 | 0.60 | 1.54 | 0.75 |  |
| ATP6V0B | 1 | p34.1 | 1.43E-05 | 4.83E-04 | 0.59 | 1.66 | 0.84 |  |
| EIF2B3 | 1 | p34.1 | 2.83E-05 | 8.11E-04 | 0.57 | 1.63 | 0.83 |  |
| HECTD3 | 1 | p34.1 | 3.21E-05 | 9.00E-04 | 0.57 | 1.76 | 0.84 |  |
| IPO13 | 1 | p34.1 | 3.62E-05 | 9.82E-04 | 0.56 | 1.81 | 0.94 |  |
| GPBP1L1 | 1 | p34.1 | 5.97E-05 | 1.44E-03 | 0.55 | 1.77 | 0.79 |  |
| MUTYH | 1 | p34.1 | 1.65E-04 | 3.10E-03 | 0.52 | 1.98 | 0.79 |  |
| LRRC41 | 1 | p34.1 | 2.08E-04 | 3.74E-03 | 0.52 | 1.53 | 0.72 |  |
| TMEM69 | 1 | p34.1 | 7.20E-04 | 9.48E-03 | 0.48 | 1.40 | 0.78 |  |
| SNORD55 | 1 | p34.1 | 1.26E-03 | 1.43E-02 | 0.46 | 1.06 | 0.89 |  |
| SNORD38 | 1 | p34.1 | 1.71E-03 | 1.80E-02 | 0.45 | 1.22 | 0.93 |  |
| RAD54L | 1 | p34.1 | 2.22E-03 | 2.18E-02 | 0.44 | 2.24 | 0.87 |  |
| B4GALT2 | 1 | p34.1 | 3.46E-03 | 2.99E-02 | 0.42 | 2.14 | 0.98 |  |
| KIF2C | 1 | p34.1 | 3.61E-03 | 3.08E-02 | 0.42 | 1.60 | 0.83 |  |
| RNF220 | 1 | p34.1 | 6.08E-03 | 4.49E-02 | 0.39 | 1.23 | 0.85 |  |
| NASP | 1 | p34.1 | 6.96E-03 | 4.94E-02 | 0.39 | 3.20 | 0.83 |  |
| RLF | 1 | p34.2 | 3.05E-08 | 4.23E-06 | 0.71 | 1.58 | 0.86 |  |
| PPIH | 1 | p34.2 | 3.10E-07 | 2.66E-05 | 0.67 | 2.15 | 0.84 | 2.37 |
| C1orf50 | 1 | p34.2 | 3.36E-07 | 2.81E-05 | 0.67 | 2.13 | 0.84 | 2.23 |
| NFYC | 1 | p34.2 | 1.10E-06 | 6.76E-05 | 0.64 | 1.71 | 0.71 |  |
| EBNA1BP2 | 1 | p34.2 | 7.46E-06 | 2.95E-04 | 0.60 | 1.87 | 0.76 | 2.85 |
| TMEM125 | 1 | p34.2 | 3.53E-05 | 9.65E-04 | 0.56 | 2.24 | 0.66 | 2.07 |
| FOXJ3 | 1 | p34.2 | 8.22E-05 | 1.85E-03 | 0.54 | 1.92 | 0.83 | 2.39 |
| DEM1 | 1 | p34.2 | 8.63E-05 | 1.91E-03 | 0.54 | 1.53 | 0.88 |  |
| MED8 | 1 | p34.2 | 5.82E-04 | 8.17E-03 | 0.48 | 1.35 | 0.84 |  |
| PPIE | 1 | p34.2 | 7.73E-04 | 9.99E-03 | 0.47 | 1.65 | 0.74 | 2.04 |
| PPCS | 1 | p34.2 | 1.12E-03 | 1.32E-02 | 0.46 | 2.02 | 0.98 | 2.31 |
| SMAP2 | 1 | p34.2 | 1.39E-03 | 1.54E-02 | 0.45 | 1.38 | 0.77 |  |
| MFSD2 | 1 | p34.2 | 1.75E-03 | 1.83E-02 | 0.44 | 1.58 | 0.88 |  |
| CAP1 | 1 | p34.2 | 2.05E-03 | 2.06E-02 | 0.44 | 1.74 | 0.94 |  |
| ZNF643 | 1 | p34.2 | 2.23E-03 | 2.18E-02 | 0.44 | 1.11 | 0.96 |  |
| CTPS | 1 | p34.2 | 2.29E-03 | 2.23E-02 | 0.43 | 2.09 | 0.87 |  |
| SCMH1 | 1 | p34.2 | 2.45E-03 | 2.35E-02 | 0.43 | 1.87 | 0.86 |  |
| HYI | 1 | p34.2 | 3.64E-03 | 3.11E-02 | 0.42 | 1.44 | 0.79 |  |
| EIF2C3 | 1 | p34.3 | 2.22E-08 | 3.25E-06 | 0.71 | 2.56 | 0.97 | 2.56 |
| MRPS15 | 1 | p34.3 | 6.91E-08 | 7.87E-06 | 0.69 | 1.99 | 0.87 |  |
| EIF2C1 | 1 | p34.3 | 1.09E-07 | 1.15E-05 | 0.68 | 3.58 | 0.86 | 3.58 |
| EIF2C4 | 1 | p34.3 | 1.51E-07 | 1.49E-05 | 0.68 | 2.27 | 0.95 | 2.27 |
| GNL2 | 1 | p34.3 | 3.13E-07 | 2.67E-05 | 0.67 | 2.51 | 0.74 | 2.51 |
| PSMB2 | 1 | p34.3 | 9.75E-07 | 6.21E-05 | 0.65 | 2.95 | 0.79 | 2.95 |
| MTF1 | 1 | p34.3 | 1.75E-06 | 9.82E-05 | 0.63 | 2.17 | 0.77 | 2.17 |
| KIAA0319L | 1 | p34.3 | 1.90E-06 | 1.04E-04 | 0.63 | 2.07 | 0.91 | 2.07 |
| PABPC4 | 1 | p34.3 | 4.05E-06 | 1.82E-04 | 0.62 | 2.16 | 0.72 | 2.55 |
| SNIP1 | 1 | p34.3 | 7.88E-06 | 3.08E-04 | 0.60 | 2.54 | 0.87 | 2.54 |
| ADPRHL2 | 1 | p34.3 | 2.11E-05 | 6.41E-04 | 0.58 | 1.94 | 0.82 | 1.94 |
| TRAPPC3 | 1 | p34.3 | 3.80E-05 | 1.02E-03 | 0.56 | 1.87 | 0.77 | 1.87 |
| TEKT2 | 1 | p34.3 | 6.38E-05 | 1.52E-03 | 0.55 | 1.42 | 0.97 | 1.42 |
| NDUFS5 | 1 | p34.3 | 9.05E-05 | 1.98E-03 | 0.54 | 1.70 | 0.76 | 1.69 |
| INPP5B | 1 | p34.3 | 1.43E-04 | 2.79E-03 | 0.53 | 1.91 | 0.92 | 1.91 |
| ZMYM4 | 1 | p34.3 | 1.44E-04 | 2.81E-03 | 0.53 | 2.02 | 0.93 | 4.00 |
| MYCBP | 1 | p34.3 | 1.48E-04 | 2.86E-03 | 0.53 | 1.33 | 0.87 | 1.68 |
| SF3A3 | 1 | p34.3 | 2.26E-04 | 3.97E-03 | 0.51 | 1.78 | 0.74 | 1.94 |
| RRAGC | 1 | p34.3 | 3.06E-04 | 5.01E-03 | 0.50 | 1.62 | 0.89 | 1.70 |
| CDCA8 | 1 | p34.3 | 7.03E-04 | 9.30E-03 | 0.48 | 3.08 | 0.87 | 3.08 |
| AKIRIN1 | 1 | p34.3 | 9.42E-04 | 1.15E-02 | 0.47 | 1.50 | 0.82 | 1.86 |
| C1orf212 | 1 | p34.3 | 1.10E-03 | 1.30E-02 | 0.46 | 1.14 | 0.92 |  |
| C1orf113 | 1 | p34.3 | 1.45E-03 | 1.58E-02 | 0.45 | 1.20 | 0.98 | 1.20 |
| C1orf216 | 1 | p34.3 | 2.01E-03 | 2.03E-02 | 0.44 | 2.25 | 0.88 | 2.25 |
| MACF1 | 1 | p34.3 | 2.91E-03 | 2.65E-02 | 0.42 | 1.88 | 0.86 | 2.91 |
| GJB4 | 1 | p34.3 | 3.73E-03 | 3.16E-02 | -0.41 | 0.99 | 1.06 |  |
| LSM10 | 1 | p34.3 | 5.46E-03 | 4.15E-02 | 0.40 | 1.24 | 0.88 |  |
| ZMYM1 | 1 | p34.3 | 5.85E-03 | 4.37E-02 | 0.40 | 1.48 | 0.93 | 2.98 |
| MANEAL | 1 | p34.3 | 6.39E-03 | 4.65E-02 | 0.39 | 2.48 | 0.78 | 2.48 |
| KPNA6 | 1 | p35.1 | 1.98E-05 | 6.09E-04 | 0.58 | 2.30 | 0.70 | 2.30 |
| HDAC1 | 1 | p35.1 | 2.57E-05 | 7.52E-04 | 0.57 | 1.83 | 0.74 | 1.83 |
| EIF3I | 1 | p35.1 | 1.06E-04 | 2.24E-03 | 0.54 | 1.59 | 0.79 | 1.59 |
| BSDC1 | 1 | p35.1 | 1.30E-04 | 2.62E-03 | 0.53 | 1.46 | 0.76 |  |
| TMEM39B | 1 | p35.1 | 4.31E-04 | 6.50E-03 | 0.49 | 1.48 | 0.83 | 1.48 |
| TXLNA | 1 | p35.1 | 4.48E-04 | 6.71E-03 | 0.49 | 1.76 | 0.77 | 1.76 |
| IQCC | 1 | p35.1 | 1.20E-03 | 1.38E-02 | 0.46 | 2.38 | 0.89 | 2.38 |
| MARCKSL1 | 1 | p35.1 | 1.22E-03 | 1.39E-02 | 0.46 | 2.70 | 0.74 | 2.70 |
| KIAA1522 | 1 | p35.1 | 1.82E-03 | 1.89E-02 | 0.44 | 2.02 | 0.71 |  |
| YARS | 1 | p35.1 | 1.95E-03 | 1.99E-02 | 0.44 | 1.01 | 0.85 |  |
| PHC2 | 1 | p35.1 | 5.49E-03 | 4.17E-02 | 0.40 |  | 0.92 |  |
| SNRNP40 | 1 | p35.2 | 2.59E-07 | 2.27E-05 | 0.67 |  | 0.74 |  |
| PUM1 | 1 | p35.2 | 2.29E-04 | 4.00E-03 | 0.51 |  | 0.76 |  |
| PEF1 | 1 | p35.2 | 4.27E-04 | 6.45E-03 | 0.49 | 2.37 | 0.90 | 2.37 |
| STX12 | 1 | p35.3 | 1.93E-05 | 5.98E-04 | 0.58 |  | 0.82 |  |
| ATPIF1 | 1 | p35.3 | 1.99E-04 | 3.61E-03 | 0.52 |  | 0.70 |  |
| TAF12 | 1 | p35.3 | 1.06E-03 | 1.26E-02 | 0.46 |  | 0.83 |  |
| DNAJC8 | 1 | p35.3 | 1.39E-03 | 1.54E-02 | 0.45 |  | 0.87 |  |
| PPP1R8 | 1 | p35.3 | 2.00E-03 | 2.03E-02 | 0.44 |  | 0.92 |  |
| RPA2 | 1 | p35.3 | 3.82E-03 | 3.22E-02 | 0.41 |  | 0.83 |  |
| ZNF593 | 1 | p36.11 | 6.57E-08 | 7.54E-06 | 0.69 |  | 0.69 |  |
| LYPLA2 | 1 | p36.11 | 4.50E-06 | 1.98E-04 | 0.61 |  | 0.75 |  |
| DHDDS | 1 | p36.11 | 8.58E-05 | 1.90E-03 | 0.54 |  | 0.81 |  |
| NUDC | 1 | p36.11 | 8.72E-05 | 1.92E-03 | 0.54 |  | 0.82 |  |
| C1orf63 | 1 | p36.11 | 1.10E-04 | 2.29E-03 | 0.53 | 1.31 | 0.72 |  |
| RPS6KA1 | 1 | p36.11 | 2.65E-04 | 4.47E-03 | 0.51 |  | 0.73 |  |
| GALE | 1 | p36.11 | 3.52E-04 | 5.56E-03 | 0.50 |  | 0.65 |  |
| HMGCL | 1 | p36.11 | 3.85E-04 | 5.95E-03 | 0.50 |  | 0.80 |  |
| C1orf201 | 1 | p36.11 | 5.37E-04 | 7.68E-03 | 0.49 |  | 0.83 |  |
| UBXN11 | 1 | p36.11 | 1.99E-03 | 2.03E-02 | 0.44 |  | 0.94 |  |
| C1orf128 | 1 | p36.11 | 2.70E-03 | 2.51E-02 | 0.43 |  | 0.81 |  |
| FUSIP1 | 1 | p36.11 | 2.78E-03 | 2.56E-02 | 0.43 |  | 0.80 |  |
| SH3BGRL3 | 1 | p36.11 | 2.80E-03 | 2.58E-02 | 0.43 |  | 0.87 |  |
| WASF2 | 1 | p36.11 | 4.39E-03 | 3.54E-02 | 0.41 |  | 0.76 |  |
| PIGV | 1 | p36.11 | 4.86E-03 | 3.81E-02 | 0.40 |  | 0.84 |  |
| DDOST | 1 | p36.12 | 2.61E-06 | 1.29E-04 | 0.63 | 1.39 | 0.80 |  |
| MUL1 | 1 | p36.12 | 8.28E-06 | 3.21E-04 | 0.60 | 1.78 | 0.88 |  |
| AOF2 | 1 | p36.12 | 1.77E-03 | 1.86E-02 | 0.44 |  | 0.79 |  |
| HP1BP3 | 1 | p36.12 | 2.23E-03 | 2.18E-02 | 0.44 | 1.04 | 0.82 |  |
| PLA2G2C | 1 | p36.12 | 3.35E-03 | 2.92E-02 | 0.42 | 1.13 | 0.92 |  |
| UBXN10 | 1 | p36.12 | 3.90E-03 | 3.26E-02 | 0.41 | 1.13 | 0.89 |  |
| UBR4 | 1 | p36.13 | 3.29E-06 | 1.54E-04 | 0.62 |  | 0.76 |  |
| SDHB | 1 | p36.13 | 1.58E-05 | 5.18E-04 | 0.58 |  | 0.73 |  |
| ATP13A2 | 1 | p36.13 | 2.34E-05 | 6.97E-04 | 0.58 |  | 0.67 |  |
| CAPZB | 1 | p36.13 | 3.74E-05 | 1.00E-03 | 0.56 |  | 0.74 |  |
| C1orf144 | 1 | p36.13 | 2.44E-04 | 4.20E-03 | 0.51 |  | 0.88 |  |
| ZBTB17 | 1 | p36.13 | 3.86E-03 | 3.24E-02 | 0.41 |  | 0.88 |  |
| CASP9 | 1 | p36.21 | 3.63E-04 | 5.68E-03 | 0.50 |  | 0.92 |  |
| RP5-845O24.3 | 1 | p36.21 | 5.14E-04 | 7.45E-03 | -0.49 |  | 1.08 |  |
| DNAJC16 | 1 | p36.21 | 2.06E-03 | 2.07E-02 | 0.44 |  | 0.95 |  |
| RP1-21O18.1 | 1 | p36.21 | 3.99E-03 | 3.31E-02 | 0.41 |  | 0.79 |  |
| UBE4B | 1 | p36.22 | 7.70E-07 | 5.17E-05 | 0.65 | 2.01 | 0.74 |  |
| MFN2 | 1 | p36.22 | 1.84E-06 | 1.02E-04 | 0.63 |  | 0.67 |  |
| KIAA2013 | 1 | p36.22 | 6.66E-06 | 2.67E-04 | 0.60 |  | 0.84 |  |
| PGD | 1 | p36.22 | 1.08E-05 | 3.91E-04 | 0.59 | 1.45 | 0.67 |  |
| MAD2L2 | 1 | p36.22 | 1.48E-04 | 2.86E-03 | 0.53 |  | 0.85 |  |
| LZIC | 1 | p36.22 | 3.81E-04 | 5.91E-03 | 0.50 | 1.64 | 0.87 |  |
| FRAP1 | 1 | p36.22 | 4.10E-04 | 6.27E-03 | 0.49 | 0.73 | 0.77 |  |
| DFFA | 1 | p36.22 | 4.23E-04 | 6.41E-03 | 0.49 | 1.59 | 0.78 |  |
| MIIP | 1 | p36.22 | 6.46E-04 | 8.80E-03 | 0.48 |  | 0.78 |  |
| AGTRAP | 1 | p36.22 | 6.91E-04 | 9.18E-03 | 0.48 |  | 0.77 |  |
| APITD1 | 1 | p36.22 | 1.40E-03 | 1.54E-02 | 0.45 | 2.00 | 0.80 |  |
| CLSTN1 | 1 | p36.22 | 1.56E-03 | 1.68E-02 | 0.45 |  | 0.82 |  |
| CTNNBIP1 | 1 | p36.22 | 1.89E-03 | 1.95E-02 | 0.44 | 1.88 | 0.80 |  |
| EXOSC10 | 1 | p36.22 | 2.05E-03 | 2.06E-02 | 0.44 | 1.21 | 0.75 |  |
| NMNAT1 | 1 | p36.22 | 2.16E-03 | 2.14E-02 | 0.44 | 1.64 | 0.95 |  |
| PEX14 | 1 | p36.22 | 4.52E-03 | 3.62E-02 | 0.41 | 1.21 | 0.87 |  |
| VPS13D | 1 | p36.22 | 4.74E-03 | 3.74E-02 | 0.41 |  | 0.88 |  |
| UBIAD1 | 1 | p36.22 | 6.36E-03 | 4.64E-02 | 0.39 | 0.79 | 0.86 |  |
| PARK7 | 1 | p36.23 | 1.03E-04 | 2.18E-03 | 0.54 |  | 0.85 |  |
| ENO1 | 1 | p36.23 | 7.35E-04 | 9.63E-03 | 0.48 |  | 0.84 |  |
| RERE | 1 | p36.23 | 1.81E-03 | 1.88E-02 | 0.44 |  | 0.75 |  |
| NPHP4 | 1 | p36.31 | 2.55E-04 | 4.35E-03 | 0.51 |  | 0.90 |  |
| KLHL21 | 1 | p36.31 | 9.49E-04 | 1.15E-02 | 0.47 |  | 0.79 |  |
| ACOT7 | 1 | p36.31 | 1.81E-03 | 1.89E-02 | 0.44 |  | 0.77 |  |
| LRRC47 | 1 | p36.32 | 1.08E-05 | 3.91E-04 | 0.59 |  | 0.78 |  |
| WDR8 | 1 | p36.32 | 7.48E-05 | 1.73E-03 | 0.54 |  | 0.81 |  |
| TPRG1L | 1 | p36.32 | 1.09E-03 | 1.29E-02 | 0.46 |  | 0.79 |  |
| C1orf174 | 1 | p36.32 | 3.02E-03 | 2.72E-02 | 0.42 |  | 0.86 |  |
| PRKCZ | 1 | p36.33 | 1.56E-03 | 1.68E-02 | 0.45 |  | 0.76 |  |
| ATAD3A | 1 | p36.33 | 3.59E-03 | 3.07E-02 | 0.42 |  | 0.78 |  |
| VWA1 | 1 | p36.33 | 5.25E-03 | 4.04E-02 | 0.40 |  | 0.96 |  |
| RP4-758J18.6 | 1 | p36.33 | 6.87E-03 | 4.91E-02 | 0.39 | 1.05 | 0.97 |  |
| BCL9 | 1 | q21.1 | 3.89E-07 | 3.11E-05 | 0.66 | 1.63 | 0.58 | 1.96 |
| CHD1L | 1 | q21.1 | 9.14E-06 | 3.44E-04 | 0.60 | 1.28 | 0.68 | 1.43 |
| ACP6 | 1 | q21.1 | 4.44E-05 | 1.15E-03 | 0.56 | 1.36 | 0.64 | 1.93 |
| PRKAB2 | 1 | q21.1 | 4.48E-05 | 1.16E-03 | 0.56 | 1.28 | 0.94 | 1.25 |
| BX248398.1 | 1 | q21.1 | 1.58E-04 | 2.99E-03 | 0.52 | 1.07 | 0.96 | 1.17 |
| AL592284.2 | 1 | q21.1 | 8.88E-04 | 1.10E-02 | 0.47 | 1.03 |  | 1.25 |
| BX284650.4 | 1 | q21.1 | 1.24E-03 | 1.41E-02 | 0.46 | 1.07 | 0.98 | 1.20 |
| PEX11B | 1 | q21.1 | 1.78E-03 | 1.86E-02 | 0.44 | 1.41 | 0.65 | 1.29 |
| BX284650.1 | 1 | q21.1 | 2.00E-03 | 2.03E-02 | 0.44 | 1.04 | 0.98 | 1.11 |
| FMO5 | 1 | q21.1 | 3.11E-03 | 2.78E-02 | 0.42 | 1.37 | 0.61 | 1.79 |
| GPR89A | 1 | q21.1 | 5.46E-03 | 4.15E-02 | 0.40 | 1.23 | 0.77 | 1.18 |
| WI2-1896O14.1 | 1 | q21.1 | 5.48E-03 | 4.16E-02 | 0.40 | 1.03 |  | 1.13 |
| VPS45 | 1 | q21.2 | 3.80E-10 | 1.29E-07 | 0.77 | 1.56 | 0.63 | 2.02 |
| RPRD2 | 1 | q21.2 | 1.77E-09 | 4.25E-07 | 0.75 | 1.41 | 0.80 | 1.53 |
| SF3B4 | 1 | q21.2 | 1.62E-08 | 2.56E-06 | 0.72 | 1.73 | 0.52 | 1.50 |
| BOLA1 | 1 | q21.2 | 6.08E-08 | 7.10E-06 | 0.69 | 1.34 | 0.72 | 1.50 |
| APH1A | 1 | q21.2 | 1.40E-07 | 1.39E-05 | 0.68 | 1.47 | 0.62 | 1.85 |
| TARS2 | 1 | q21.2 | 5.65E-07 | 4.11E-05 | 0.66 | 1.42 | 0.80 | 1.97 |
| RP11-403I13.6 | 1 | q21.2 | 2.81E-05 | 8.07E-04 | 0.57 | 1.04 | 0.98 | 1.13 |
| PRPF3 | 1 | q21.2 | 5.21E-05 | 1.30E-03 | 0.55 | 1.58 | 0.41 | 1.60 |
| FAM91A2 | 1 | q21.2 | 8.21E-05 | 1.85E-03 | 0.54 | 1.16 | 0.94 | 2.09 |
| MRPS21 | 1 | q21.2 | 6.64E-03 | 4.78E-02 | 0.39 | 1.43 | 0.90 | 1.63 |
| ARNT | 1 | q21.3 | 1.97E-08 | 2.95E-06 | 0.71 | 1.44 | 1.03 | 1.86 |
| SNX27 | 1 | q21.3 | 3.34E-07 | 2.81E-05 | 0.67 | 1.59 | 0.57 | 1.52 |
| SELENBP1 | 1 | q21.3 | 1.88E-06 | 1.03E-04 | 0.63 | 1.56 | 0.56 | 1.28 |
| MRPL9 | 1 | q21.3 | 5.66E-06 | 2.36E-04 | 0.61 | 1.35 | 0.64 | 1.46 |
| SNAPIN | 1 | q21.3 | 5.98E-06 | 2.46E-04 | 0.61 | 1.29 | 0.77 | 1.63 |
| SLC39A1 | 1 | q21.3 | 5.98E-06 | 2.46E-04 | 0.61 | 1.49 | 0.75 |  |
| GOLPH3L | 1 | q21.3 | 1.14E-05 | 4.07E-04 | 0.59 | 1.38 | 0.85 | 2.08 |
| CRTC2 | 1 | q21.3 | 1.85E-05 | 5.79E-04 | 0.58 | 1.28 | 0.85 |  |
| VPS72 | 1 | q21.3 | 2.73E-05 | 7.89E-04 | 0.57 | 1.49 | 0.52 | 1.30 |
| CREB3L4 | 1 | q21.3 | 5.33E-05 | 1.32E-03 | 0.55 | 1.67 | 0.62 |  |
| ENSA | 1 | q21.3 | 6.19E-05 | 1.48E-03 | 0.55 | 1.38 | 1.17 | 3.21 |
| TUFT1 | 1 | q21.3 | 9.00E-05 | 1.97E-03 | 0.54 | 1.69 | 0.60 | 1.65 |
| PSMD4 | 1 | q21.3 | 1.00E-04 | 2.14E-03 | 0.54 | 1.38 | 0.60 | 1.55 |
| LASS2 | 1 | q21.3 | 1.84E-04 | 3.39E-03 | 0.52 | 1.65 | 0.61 | 1.55 |
| POGZ | 1 | q21.3 | 2.02E-04 | 3.66E-03 | 0.52 | 1.23 | 0.60 | 1.29 |
| PSMB4 | 1 | q21.3 | 2.69E-04 | 4.52E-03 | 0.51 | 1.38 | 0.76 | 1.19 |
| UBAP2L | 1 | q21.3 | 3.53E-04 | 5.57E-03 | 0.50 | 1.37 | 0.67 | 0.89 |
| INTS3 | 1 | q21.3 | 4.17E-04 | 6.36E-03 | 0.49 | 1.31 | 1.02 |  |
| C1orf56 | 1 | q21.3 | 5.72E-04 | 8.08E-03 | 0.48 | 1.17 | 0.82 | 1.19 |
| ZNF687 | 1 | q21.3 | 6.14E-04 | 8.49E-03 | 0.48 | 1.18 | 0.85 | 1.17 |
| LYSMD1 | 1 | q21.3 | 6.54E-04 | 8.87E-03 | 0.48 | 1.24 | 0.91 | 1.14 |
| BNIPL | 1 | q21.3 | 6.56E-04 | 8.88E-03 | 0.48 | 1.28 | 0.93 | 1.87 |
| PRUNE | 1 | q21.3 | 7.85E-04 | 1.01E-02 | 0.47 | 1.43 | 0.66 | 1.23 |
| ILF2 | 1 | q21.3 | 1.05E-03 | 1.25E-02 | 0.46 | 1.26 | 0.89 | 1.24 |
| SCNM1 | 1 | q21.3 | 1.22E-03 | 1.39E-02 | 0.46 | 1.32 | 0.76 | 1.46 |
| SETDB1 | 1 | q21.3 | 1.35E-03 | 1.50E-02 | 0.45 | 1.46 | 0.65 | 1.14 |
| GATAD2B | 1 | q21.3 | 1.46E-03 | 1.59E-02 | 0.45 | 1.30 | 0.74 |  |
| FLAD1 | 1 | q21.3 | 1.54E-03 | 1.66E-02 | 0.45 | 1.41 | 0.90 | 1.18 |
| MCL1 | 1 | q21.3 | 1.90E-03 | 1.96E-02 | 0.44 | 1.18 | 0.97 | 1.89 |
| PMVK | 1 | q21.3 | 2.14E-03 | 2.12E-02 | 0.44 | 1.37 | 0.60 | 1.50 |
| SLC27A3 | 1 | q21.3 | 2.90E-03 | 2.64E-02 | 0.43 | 1.46 | 0.79 |  |
| SHC1 | 1 | q21.3 | 3.27E-03 | 2.87E-02 | 0.42 | 1.23 | 1.31 | 1.76 |
| PI4KB | 1 | q21.3 | 3.64E-03 | 3.11E-02 | 0.42 | 1.26 | 0.78 | 1.10 |
| CDC42SE1 | 1 | q21.3 | 3.76E-03 | 3.18E-02 | 0.41 | 1.21 | 0.72 | 1.09 |
| RAB13 | 1 | q21.3 | 4.40E-03 | 3.55E-02 | 0.41 | 1.33 | 0.66 |  |
| C1orf77 | 1 | q21.3 | 4.53E-03 | 3.62E-02 | 0.41 | 1.30 | 0.89 | 1.23 |
| PYGO2 | 1 | q21.3 | 5.01E-03 | 3.89E-02 | 0.40 | 1.27 | 0.84 | 1.19 |
| PIP5K1A | 1 | q21.3 | 5.02E-03 | 3.90E-02 | 0.40 | 1.11 | 0.95 | 0.96 |
| UBQLN4 | 1 | q22 | 3.84E-07 | 3.11E-05 | 0.66 | 1.59 | 0.68 | 1.44 |
| PMF1 | 1 | q22 | 1.95E-06 | 1.05E-04 | 0.63 | 1.46 | 0.67 | 1.65 |
| DAP3 | 1 | q22 | 2.04E-06 | 1.08E-04 | 0.63 | 1.38 | 0.66 | 1.21 |
| ROBLD3 | 1 | q22 | 1.09E-05 | 3.93E-04 | 0.59 | 1.52 | 0.75 | 1.61 |
| CLK2 | 1 | q22 | 1.21E-05 | 4.24E-04 | 0.59 | 1.41 | 0.71 | 1.34 |
| MTX1 | 1 | q22 | 1.23E-05 | 4.31E-04 | 0.59 | 1.42 | 0.75 | 1.36 |
| RUSC1 | 1 | q22 | 5.73E-05 | 1.40E-03 | 0.55 | 1.55 | 0.58 | 1.47 |
| YY1AP1 | 1 | q22 | 6.50E-05 | 1.54E-03 | 0.55 | 1.38 | 0.86 | 1.45 |
| SLC25A44 | 1 | q22 | 1.34E-04 | 2.66E-03 | 0.53 | 1.33 | 0.93 | 1.43 |
| KIAA0907 | 1 | q22 | 2.17E-04 | 3.85E-03 | 0.51 | 1.40 | 0.85 | 1.68 |
| C1orf2 | 1 | q22 | 5.90E-04 | 8.23E-03 | 0.48 | 1.39 | 0.69 | 1.15 |
| EFNA1 | 1 | q22 | 6.18E-04 | 8.51E-03 | 0.48 | 1.51 | 0.71 | 2.21 |
| GBA | 1 | q22 | 6.79E-04 | 9.08E-03 | 0.48 | 1.31 | 0.66 | 1.51 |
| RAG1AP1 | 1 | q22 | 6.89E-04 | 9.17E-03 | 0.48 | 1.43 | 0.48 | 1.65 |
| MUC1 | 1 | q22 | 7.69E-04 | 9.96E-03 | 0.47 | 1.41 | 0.62 | 3.63 |
| HCN3 | 1 | q22 | 1.36E-03 | 1.52E-02 | 0.45 | 1.25 | 0.88 | 1.32 |
| RAB25 | 1 | q22 | 1.39E-03 | 1.53E-02 | 0.45 | 1.40 | 0.65 | 1.57 |
| SSR2 | 1 | q22 | 1.68E-03 | 1.78E-02 | 0.45 | 1.36 | 0.79 | 1.92 |
| MEF2D | 1 | q22 | 2.08E-03 | 2.08E-02 | 0.44 | 1.23 | 0.73 | 1.39 |
| FDPS | 1 | q22 | 2.24E-03 | 2.19E-02 | 0.44 | 1.32 | 1.01 | 1.96 |
| EFNA4 | 1 | q22 | 3.42E-03 | 2.96E-02 | 0.42 | 1.23 | 0.69 | 1.52 |
| LMNA | 1 | q22 | 3.86E-03 | 3.24E-02 | 0.41 | 1.35 | 0.76 | 1.76 |
| C1orf66 | 1 | q23.1 | 5.55E-06 | 2.33E-04 | 0.61 | 1.56 | 0.67 | 1.63 |
| ARHGEF11 | 1 | q23.1 | 2.61E-05 | 7.62E-04 | 0.57 | 1.25 | 0.85 | 1.29 |
| MRPL24 | 1 | q23.1 | 1.89E-04 | 3.46E-03 | 0.52 | 1.52 | 0.83 | 1.31 |
| ISG20L2 | 1 | q23.1 | 9.28E-04 | 1.14E-02 | 0.47 | 1.22 | 0.82 | 1.38 |
| PRCC | 1 | q23.1 | 2.56E-03 | 2.42E-02 | 0.43 | 1.24 | 0.64 | 1.22 |
| GPATCH4 | 1 | q23.1 | 5.90E-03 | 4.39E-02 | 0.40 | 1.31 | 0.86 | 1.10 |
| NCSTN | 1 | q23.2 | 6.52E-05 | 1.54E-03 | 0.55 | 1.32 | 0.97 | 1.54 |
| PIGM | 1 | q23.2 | 1.12E-04 | 2.33E-03 | 0.53 | 1.34 | 0.69 | 1.47 |
| IGSF9 | 1 | q23.2 | 1.16E-04 | 2.40E-03 | 0.53 | 1.55 | 0.34 | 1.67 |
| COPA | 1 | q23.2 | 2.10E-04 | 3.76E-03 | 0.52 | 1.22 | 0.71 | 1.37 |
| VANGL2 | 1 | q23.2 | 2.90E-04 | 4.80E-03 | 0.51 | 1.77 | 0.48 | 2.44 |
| WDR42A | 1 | q23.2 | 3.17E-04 | 5.14E-03 | 0.50 | 1.36 | 0.83 | 1.47 |
| RP11-226L15.1 | 1 | q23.2 | 8.54E-04 | 1.07E-02 | 0.47 | 1.05 | 0.93 | 1.09 |
| DUSP23 | 1 | q23.2 | 2.66E-03 | 2.48E-02 | 0.43 | 1.45 | 0.61 | 1.21 |
| PEX19 | 1 | q23.2 | 6.32E-03 | 4.63E-02 | 0.39 | 1.21 | 0.85 | 1.35 |
| USP21 | 1 | q23.3 | 2.98E-08 | 4.18E-06 | 0.71 | 1.41 | 0.62 | 1.64 |
| SDHC | 1 | q23.3 | 7.21E-06 | 2.87E-04 | 0.60 | 1.37 | 0.61 | 1.93 |
| UFC1 | 1 | q23.3 | 7.46E-06 | 2.95E-04 | 0.60 | 1.34 | 0.67 | 1.50 |
| ATF6 | 1 | q23.3 | 1.18E-05 | 4.18E-04 | 0.59 | 1.24 | 0.77 | 1.38 |
| PPOX | 1 | q23.3 | 1.28E-05 | 4.43E-04 | 0.59 | 1.37 | 0.65 | 1.71 |
| DUSP12 | 1 | q23.3 | 1.13E-04 | 2.34E-03 | 0.53 | 1.23 | 0.68 | 1.40 |
| PBX1 | 1 | q23.3 | 4.28E-04 | 6.46E-03 | 0.49 | 1.77 | 0.50 | 1.53 |
| KLHDC9 | 1 | q23.3 | 8.93E-04 | 1.11E-02 | 0.47 | 1.63 | 0.56 | 1.82 |
| NOS1AP | 1 | q23.3 | 1.15E-03 | 1.34E-02 | 0.46 | 1.06 |  | 1.09 |
| B4GALT3 | 1 | q23.3 | 1.73E-03 | 1.82E-02 | 0.44 | 1.34 | 0.74 | 1.31 |
| PVRL4 | 1 | q23.3 | 3.99E-03 | 3.31E-02 | 0.41 | 1.28 | 1.15 | 1.43 |
| NDUFS2 | 1 | q23.3 | 6.08E-03 | 4.49E-02 | 0.39 | 1.16 | 0.81 | 1.25 |
| POGK | 1 | q24.1 | 8.85E-06 | 3.36E-04 | 0.60 | 1.34 |  | 1.43 |
| ALDH9A1 | 1 | q24.1 | 1.62E-05 | 5.27E-04 | 0.58 | 1.26 | 0.58 | 1.57 |
| TMCO1 | 1 | q24.1 | 1.07E-04 | 2.24E-03 | 0.54 | 1.47 | 0.59 | 1.80 |
| MGST3 | 1 | q24.1 | 2.68E-03 | 2.50E-02 | 0.43 | 1.28 | 0.74 | 1.31 |
| SCYL3 | 1 | q24.2 | 2.91E-07 | 2.52E-05 | 0.67 | 1.22 |  | 1.57 |
| BLZF1 | 1 | q24.2 | 2.61E-06 | 1.29E-04 | 0.63 | 1.22 |  | 1.44 |
| C1orf112 | 1 | q24.2 | 3.92E-06 | 1.79E-04 | 0.62 | 1.36 |  | 1.61 |
| C1orf156 | 1 | q24.2 | 1.45E-05 | 4.86E-04 | 0.59 | 1.10 |  | 1.29 |
| MPZL1 | 1 | q24.2 | 3.74E-05 | 1.00E-03 | 0.56 | 1.39 | 0.60 | 1.54 |
| GORAB | 1 | q24.2 | 1.06E-04 | 2.23E-03 | 0.54 | 1.18 | 1.03 | 1.39 |
| ATP1B1 | 1 | q24.2 | 2.22E-04 | 3.93E-03 | 0.51 | 1.27 | 0.24 | 2.46 |
| IQWD1 | 1 | q24.2 | 6.71E-04 | 9.00E-03 | 0.48 | 1.36 | 0.49 | 1.77 |
| AL033532.36 | 1 | q24.2 | 1.08E-03 | 1.28E-02 | 0.46 | 1.08 | 0.92 | 1.10 |
| NME7 | 1 | q24.2 | 1.23E-03 | 1.40E-02 | 0.46 | 1.16 | 0.90 | 1.44 |
| TIPRL | 1 | q24.2 | 1.49E-03 | 1.62E-02 | 0.45 | 1.23 | 0.76 | 1.30 |
| KIFAP3 | 1 | q24.2 | 3.48E-03 | 3.00E-02 | 0.42 | 1.26 | 0.86 | 1.15 |
| METTL13 | 1 | q24.3 | 9.33E-15 | 2.02E-11 | 0.86 | 1.29 | 0.90 | 1.63 |
| VAMP4 | 1 | q24.3 | 1.77E-11 | 1.01E-08 | 0.80 | 1.27 | 1.30 | 1.67 |
| PIGC | 1 | q24.3 | 7.03E-06 | 2.81E-04 | 0.60 | 1.36 |  | 1.66 |
| MRPS14 | 1 | q25.1 | 5.88E-08 | 6.96E-06 | 0.70 | 1.15 |  | 1.27 |
| CENPL | 1 | q25.1 | 5.28E-06 | 2.24E-04 | 0.61 | 1.19 |  | 1.31 |
| KIAA0040 | 1 | q25.1 | 8.08E-06 | 3.14E-04 | 0.60 | 1.29 |  | 1.61 |
| DARS2 | 1 | q25.1 | 3.79E-05 | 1.01E-03 | 0.56 | 1.54 |  | 1.74 |
| PRDX6 | 1 | q25.1 | 4.39E-05 | 1.14E-03 | 0.56 | 1.16 |  | 1.35 |
| KLHL20 | 1 | q25.1 | 8.16E-05 | 1.84E-03 | 0.54 | 1.08 |  | 1.26 |
| CACYBP | 1 | q25.1 | 1.46E-04 | 2.84E-03 | 0.53 | 1.04 |  | 1.72 |
| SERPINC1 | 1 | q25.1 | 3.89E-04 | 6.00E-03 | 0.50 | 1.03 |  | 1.08 |
| RFWD2 | 1 | q25.1-q25.2 | 2.08E-07 | 1.92E-05 | 0.67 | 1.41 |  | 1.39 |
| CEP350 | 1 | q25.2 | 1.96E-06 | 1.06E-04 | 0.63 | 1.35 | 0.44 | 1.56 |
| FAM20B | 1 | q25.2 | 1.38E-05 | 4.70E-04 | 0.59 | 1.37 |  | 1.53 |
| RALGPS2 | 1 | q25.2 | 3.59E-05 | 9.78E-04 | 0.56 | 1.13 |  | 1.34 |
| TOR3A | 1 | q25.2 | 3.77E-04 | 5.86E-03 | 0.50 | 1.32 |  | 1.45 |
| TDRD5 | 1 | q25.2 | 1.25E-03 | 1.42E-02 | 0.46 | 1.18 |  | 1.40 |
| TOR1AIP1 | 1 | q25.2 | 2.02E-03 | 2.04E-02 | 0.44 | 1.10 |  | 1.60 |
| ACBD6 | 1 | q25.2-q25.3 | 1.73E-04 | 3.21E-03 | 0.52 | 1.29 | 0.67 | 1.22 |
| C1orf26 | 1 | q25.3 | 5.81E-07 | 4.19E-05 | 0.66 | 1.22 | 0.75 | 1.46 |
| STX6 | 1 | q25.3 | 5.38E-04 | 7.69E-03 | 0.49 | 1.20 | 0.76 | 1.44 |
| GS1-115G20.1 | 1 | q25.3 | 7.56E-04 | 9.83E-03 | 0.47 | 1.11 | 0.98 | 1.18 |
| RNASEL | 1 | q25.3 | 7.91E-04 | 1.02E-02 | 0.47 | 1.16 | 0.79 | 1.26 |
| C1orf25 | 1 | q25.3 | 1.53E-03 | 1.65E-02 | 0.45 | 1.10 | 0.79 | 1.28 |
| MR1 | 1 | q25.3 | 3.02E-03 | 2.72E-02 | 0.42 | 1.13 | 0.55 | 1.45 |
| FAM129A | 1 | q25.3 | 3.07E-03 | 2.75E-02 | 0.42 | 1.40 | 0.49 | 2.64 |
| XPR1 | 1 | q25.3 | 4.93E-03 | 3.85E-02 | 0.40 | 1.32 | 0.56 | 1.22 |
| TSEN15 | 1 | q25.3 | 5.39E-03 | 4.12E-02 | 0.40 | 1.25 | 0.43 | 1.43 |
| SMG7 | 1 | q25.3 | 6.03E-03 | 4.46E-02 | 0.39 | 1.19 | 0.69 | 1.21 |
| TPR | 1 | q31.1 | 7.82E-07 | 5.23E-05 | 0.65 | 1.38 | 0.44 | 1.61 |
| FAM5C | 1 | q31.1 | 1.21E-05 | 4.24E-04 | 0.59 | 1.45 | 0.76 | 3.49 |
| C1orf27 | 1 | q31.1 | 9.19E-04 | 1.13E-02 | 0.47 | 1.03 | 0.84 | 1.24 |
| NEK7 | 1 | q31.3 | 3.26E-06 | 1.53E-04 | 0.62 | 0.97 |  | 1.17 |
| ZBTB41 | 1 | q31.3 | 6.33E-03 | 4.63E-02 | 0.39 | 1.15 |  | 1.51 |
| SOX13 | 1 | q32.1 | 2.56E-07 | 2.25E-05 | 0.67 | 1.58 | 0.55 | 1.59 |
| LGTN | 1 | q32.1 | 6.74E-07 | 4.67E-05 | 0.65 | 1.37 | 0.61 | 1.58 |
| DDX59 | 1 | q32.1 | 1.22E-06 | 7.33E-05 | 0.64 | 1.34 |  | 1.48 |
| JARID1B | 1 | q32.1 | 1.80E-06 | 1.00E-04 | 0.63 | 1.59 | 0.55 | 1.57 |
| PRELP | 1 | q32.1 | 2.46E-06 | 1.24E-04 | 0.63 | 1.07 | 1.00 | 1.20 |
| IPO9 | 1 | q32.1 | 4.71E-06 | 2.04E-04 | 0.61 | 1.28 | 0.57 | 1.30 |
| KLHL12 | 1 | q32.1 | 7.79E-06 | 3.06E-04 | 0.60 | 1.27 | 0.70 | 1.43 |
| CYB5R1 | 1 | q32.1 | 7.80E-06 | 3.06E-04 | 0.60 | 1.35 | 0.56 | 1.50 |
| PLEKHA6 | 1 | q32.1 | 1.39E-05 | 4.71E-04 | 0.59 | 1.53 | 0.69 | 1.82 |
| NUCKS1 | 1 | q32.1 | 1.57E-05 | 5.16E-04 | 0.59 | 1.46 | 0.72 | 1.43 |
| RNPEP | 1 | q32.1 | 2.18E-05 | 6.58E-04 | 0.58 | 1.33 | 0.60 | 1.48 |
| DSTYK | 1 | q32.1 | 4.49E-05 | 1.16E-03 | 0.56 | 1.26 | 0.61 | 1.19 |
| RBBP5 | 1 | q32.1 | 1.12E-04 | 2.33E-03 | 0.53 | 1.24 | 0.78 | 1.29 |
| TIMM17A | 1 | q32.1 | 1.96E-04 | 3.57E-03 | 0.52 | 1.08 | 0.96 | 1.12 |
| ADIPOR1 | 1 | q32.1 | 2.02E-04 | 3.66E-03 | 0.52 | 1.45 | 0.56 | 1.31 |
| SLC41A1 | 1 | q32.1 | 2.69E-04 | 4.52E-03 | 0.51 | 1.33 | 0.72 | 1.13 |
| ZNF281 | 1 | q32.1 | 8.92E-04 | 1.11E-02 | 0.47 | 1.40 |  | 1.55 |
| TMEM9 | 1 | q32.1 | 1.03E-03 | 1.23E-02 | 0.46 | 1.47 | 0.47 | 1.27 |
| SRGAP2 | 1 | q32.1 | 1.14E-03 | 1.33E-02 | 0.46 | 1.13 | 0.86 | 1.26 |
| KIF14 | 1 | q32.1 | 1.33E-03 | 1.49E-02 | 0.45 | 1.14 |  | 1.25 |
| UBE2T | 1 | q32.1 | 1.62E-03 | 1.72E-02 | 0.45 | 1.19 | 0.55 | 1.12 |
| MAPKAPK2 | 1 | q32.1 | 1.71E-03 | 1.80E-02 | 0.45 | 1.34 | 0.69 | 1.32 |
| TMEM183A | 1 | q32.1 | 2.43E-03 | 2.33E-02 | 0.43 | 1.22 | 0.62 | 1.21 |
| LRRN2 | 1 | q32.1 | 2.57E-03 | 2.42E-02 | 0.43 | 1.55 | 0.49 | 1.51 |
| ELK4 | 1 | q32.1 | 5.80E-03 | 4.34E-02 | 0.40 | 1.04 | 0.92 | 1.06 |
| C1orf74 | 1 | q32.2 | 1.23E-06 | 7.40E-05 | 0.64 | 1.19 | 0.76 | 1.44 |
| RCOR3 | 1 | q32.2 | 4.60E-06 | 2.01E-04 | 0.61 | 1.33 | 0.47 | 1.93 |
| HHAT | 1 | q32.2 | 1.77E-05 | 5.59E-04 | 0.58 | 1.36 | 0.58 | 1.54 |
| CD46 | 1 | q32.2 | 3.70E-05 | 9.97E-04 | 0.56 | 1.26 | 0.92 | 1.73 |
| IRF6 | 1 | q32.2 | 1.14E-04 | 2.35E-03 | 0.53 | 1.21 | 1.01 | 1.56 |
| hsa-mir-29c | 1 | q32.2 | 1.61E-03 | 1.71E-02 | 0.45 | 1.11 | 0.59 | 1.46 |
| TRAF5 | 1 | q32.2-q32.3 | 1.48E-04 | 2.86E-03 | 0.53 | 1.24 | 0.59 | 1.79 |
| NSL1 | 1 | q32.3 | 1.29E-04 | 2.59E-03 | 0.53 | 1.49 | 0.48 | 1.54 |
| RP11-348H3.6 | 1 | q32.3 | 3.58E-04 | 5.63E-03 | 0.50 | 1.12 | 0.90 | 1.15 |
| PPP2R5A | 1 | q32.3 | 5.15E-04 | 7.45E-03 | 0.49 | 1.33 | 0.45 | 1.45 |
| C1orf97 | 1 | q32.3 | 8.06E-04 | 1.03E-02 | 0.47 | 1.28 | 0.66 | 1.57 |
| LPGAT1 | 1 | q32.3 | 9.13E-04 | 1.12E-02 | 0.47 | 1.17 | 0.58 | 1.53 |
| NENF | 1 | q32.3 | 2.59E-03 | 2.44E-02 | 0.43 | 1.18 | 0.80 | 1.17 |
| RPS6KC1 | 1 | q32.3 | 2.77E-03 | 2.56E-02 | 0.43 | 1.07 | 0.85 | 1.25 |
| DTL | 1 | q32.3 | 5.60E-03 | 4.23E-02 | 0.40 | 1.44 | 0.39 | 1.36 |
| GPATCH2 | 1 | q41 | 2.29E-11 | 1.26E-08 | 0.80 | 1.34 | 0.60 | 1.56 |
| SPATA17 | 1 | q41 | 1.02E-06 | 6.41E-05 | 0.64 | 1.16 | 0.75 | 1.30 |
| TP53BP2 | 1 | q41 | 2.86E-06 | 1.38E-04 | 0.62 | 1.43 | 0.62 | 1.46 |
| RAB3GAP2 | 1 | q41 | 8.14E-06 | 3.16E-04 | 0.60 | 1.35 |  | 1.46 |
| IARS2 | 1 | q41 | 1.33E-05 | 4.54E-04 | 0.59 | 1.33 |  | 1.70 |
| CAPN2 | 1 | q41 | 3.67E-05 | 9.92E-04 | 0.56 | 1.35 | 0.97 | 1.38 |
| EPRS | 1 | q41 | 7.13E-05 | 1.66E-03 | 0.55 | 1.34 |  | 1.37 |
| CAPN8 | 1 | q41 | 2.11E-04 | 3.77E-03 | 0.52 | 1.59 | 0.94 | 3.48 |
| ESRRG | 1 | q41 | 5.24E-04 | 7.53E-03 | 0.49 | 1.15 | 0.55 | 2.49 |
| TLR5 | 1 | q41 | 1.67E-03 | 1.77E-02 | 0.45 | 1.46 | 0.65 | 1.34 |
| MOSC1 | 1 | q41 | 2.01E-03 | 2.04E-02 | 0.44 | 1.35 | 0.67 | 1.42 |
| MOSC2 | 1 | q41 | 3.78E-03 | 3.19E-02 | 0.41 | 1.36 | 0.87 | 1.37 |
| BPNT1 | 1 | q41 | 3.87E-03 | 3.25E-02 | 0.41 | 1.18 |  | 1.33 |
| TAF1A | 1 | q41 | 5.29E-03 | 4.06E-02 | 0.40 | 1.09 | 0.78 | 1.15 |
| KCTD3 | 1 | q41 | 6.76E-03 | 4.84E-02 | 0.39 | 1.01 | 0.74 | 1.68 |
| FBXO28 | 1 | q42.11 | 7.09E-04 | 9.36E-03 | 0.48 | 1.31 | 0.59 | 1.37 |
| DEGS1 | 1 | q42.11 | 8.87E-04 | 1.10E-02 | 0.47 | 1.50 | 0.75 | 1.28 |
| NVL | 1 | q42.11 | 1.45E-03 | 1.58E-02 | 0.45 | 1.46 | 0.65 | 1.51 |
| WDR26 | 1 | q42.11-q42.12 | 1.03E-03 | 1.23E-02 | 0.46 | 1.34 | 0.84 | 1.37 |
| LEFTY1 | 1 | q42.12 | 1.17E-04 | 2.41E-03 | 0.53 | 1.30 | 0.69 | 1.39 |
| PARP1 | 1 | q42.12 | 1.19E-04 | 2.44E-03 | 0.53 | 1.30 | 0.65 | 1.26 |
| ENAH | 1 | q42.12 | 1.34E-03 | 1.50E-02 | 0.45 | 1.36 | 0.68 | 1.15 |
| NUP133 | 1 | q42.13 | 5.39E-08 | 6.47E-06 | 0.70 | 1.30 | 0.58 | 1.49 |
| C1orf35 | 1 | q42.13 | 1.22E-07 | 1.26E-05 | 0.68 | 1.44 | 0.60 | 1.38 |
| ARF1 | 1 | q42.13 | 7.47E-05 | 1.73E-03 | 0.54 | 1.28 | 0.83 | 1.31 |
| MRPL55 | 1 | q42.13 | 7.78E-05 | 1.78E-03 | 0.54 | 1.38 | 0.83 | 1.44 |
| URB2 | 1 | q42.13 | 8.65E-05 | 1.92E-03 | 0.54 | 1.16 | 0.78 | 1.25 |
| SNAP47 | 1 | q42.13 | 1.81E-04 | 3.34E-03 | 0.52 | 1.33 | 0.63 | 1.36 |
| C1orf69 | 1 | q42.13 | 2.41E-04 | 4.17E-03 | 0.51 | 1.10 | 0.93 | 1.20 |
| TRIM11 | 1 | q42.13 | 7.68E-04 | 9.96E-03 | 0.47 | 1.29 | 0.69 | 1.17 |
| PSEN2 | 1 | q42.13 | 8.91E-04 | 1.11E-02 | 0.47 | 1.34 | 0.77 | 1.32 |
| TAF5L | 1 | q42.13 | 2.74E-03 | 2.54E-02 | 0.43 | 1.24 | 0.71 | 1.21 |
| COG2 | 1 | q42.2 | 3.87E-07 | 3.11E-05 | 0.66 | 1.39 | 0.70 | 1.55 |
| C1orf57 | 1 | q42.2 | 8.47E-07 | 5.54E-05 | 0.65 | 1.51 | 0.34 | 1.76 |
| GNPAT | 1 | q42.2 | 4.78E-06 | 2.06E-04 | 0.61 | 1.27 | 0.66 | 1.35 |
| TTC13 | 1 | q42.2 | 1.30E-05 | 4.49E-04 | 0.59 | 1.27 | 0.74 | 1.30 |
| ARV1 | 1 | q42.2 | 3.09E-05 | 8.74E-04 | 0.57 | 1.37 | 0.69 | 1.39 |
| TARBP1 | 1 | q42.2 | 2.52E-04 | 4.31E-03 | 0.51 | 1.25 | 0.79 | 1.68 |
| C1orf131 | 1 | q42.2 | 1.04E-03 | 1.25E-02 | 0.46 | 1.26 | 0.74 | 1.41 |
| EXOC8 | 1 | q42.2 | 2.86E-03 | 2.62E-02 | 0.43 | 1.07 | 0.88 | 1.36 |
| CAPN9 | 1 | q42.2 | 2.96E-03 | 2.68E-02 | 0.42 | 2.04 | 1.12 | 1.70 |
| C1orf198 | 1 | q42.2 | 3.92E-03 | 3.27E-02 | 0.41 | 1.41 | 0.82 | 1.17 |
| GGPS1 | 1 | q42.3 | 1.04E-04 | 2.20E-03 | 0.54 | 1.25 | 0.77 | 1.31 |
| B3GALNT2 | 1 | q42.3 | 8.39E-04 | 1.06E-02 | 0.47 | 1.17 | 0.78 | 1.15 |
| TOMM20 | 1 | q42.3 | 2.44E-03 | 2.34E-02 | 0.43 | 1.29 | 0.60 | 1.41 |
| TBCE | 1 | q42.3 | 4.18E-03 | 3.42E-02 | 0.41 | 1.24 | 1.04 | 1.42 |
| MTR | 1 | q43 | 2.12E-03 | 2.12E-02 | 0.44 | 1.19 | 0.84 | 1.25 |
| SDCCAG8 | 1 | q43 | 2.32E-03 | 2.25E-02 | 0.43 | 1.07 | 0.87 | 1.18 |
| FH | 1 | q43 | 3.42E-03 | 2.96E-02 | 0.42 | 1.32 | 0.75 | 1.38 |
| AHCTF1 | 1 | q44 | 1.34E-05 | 4.58E-04 | 0.59 | 1.14 |  | 1.33 |
| ZNF672 | 1 | q44 | 2.12E-05 | 6.43E-04 | 0.58 | 1.35 |  | 1.49 |
| PPPDE1 | 1 | q44 | 4.07E-05 | 1.08E-03 | 0.56 | 1.26 | 0.65 | 1.54 |
| FAM36A | 1 | q44 | 1.99E-04 | 3.62E-03 | 0.52 | 1.22 | 0.61 | 1.45 |
| ZNF669 | 1 | q44 | 4.81E-04 | 7.07E-03 | 0.49 | 1.10 |  | 1.15 |
| ZNF692 | 1 | q44 | 5.81E-04 | 8.17E-03 | 0.48 | 1.51 |  | 1.42 |
| SH3BP5L | 1 | q44 | 5.98E-04 | 8.31E-03 | 0.48 | 1.53 |  | 1.45 |
| RP11-439E19.3 | 1 | q44 | 3.04E-03 | 2.73E-02 | 0.42 | 1.64 |  | 1.21 |
| HNRNPU | 1 | q44 | 4.39E-03 | 3.54E-02 | 0.41 | 1.05 | 0.83 | 1.32 |
| C1orf71 | 1 | q44 | 4.40E-03 | 3.55E-02 | 0.41 | 1.45 |  | 1.32 |
| PGBD2 | 1 | q44 | 5.78E-03 | 4.33E-02 | 0.40 | 1.17 |  | 1.17 |
| SCCPDH | 1 | q44 | 6.29E-03 | 4.61E-02 | 0.39 | 1.46 |  | 1.38 |
| USP39 | 2 | p11.2 | 2.11E-03 | 2.11E-02 | 0.44 |  | 0.85 |  |
| HK2 | 2 | p12 | 3.18E-07 | 2.69E-05 | 0.67 | 5.87 | 0.76 | 21.82 |
| POLE4 | 2 | p12 | 3.69E-06 | 1.70E-04 | 0.62 | 2.05 | 0.70 | 4.18 |
| LBX2 | 2 | p13.1 | 2.86E-06 | 1.38E-04 | 0.62 | 1.31 | 0.85 |  |
| INO80B | 2 | p13.1 | 2.81E-05 | 8.07E-04 | 0.57 | 1.57 | 0.73 |  |
| DGUOK | 2 | p13.1 | 5.44E-05 | 1.34E-03 | 0.55 | 1.23 | 0.79 |  |
| MRPL53 | 2 | p13.1 | 3.07E-04 | 5.01E-03 | 0.50 | 1.46 | 0.84 |  |
| TTC31 | 2 | p13.1 | 5.04E-04 | 7.32E-03 | 0.49 | 1.44 | 0.85 |  |
| RTKN | 2 | p13.1 | 8.15E-04 | 1.03E-02 | 0.47 | 1.63 | 0.77 |  |
| PCGF1 | 2 | p13.1 | 1.10E-03 | 1.29E-02 | 0.46 | 1.53 | 0.92 |  |
| GCS1 | 2 | p13.1 | 1.70E-03 | 1.79E-02 | 0.45 | 1.48 | 0.86 |  |
| STAMBP | 2 | p13.1 | 3.02E-03 | 2.72E-02 | 0.42 | 1.86 | 0.91 |  |
| SEMA4F | 2 | p13.1 | 4.32E-03 | 3.50E-02 | 0.41 | 1.40 | 0.79 |  |
| BOLA3 | 2 | p13.1 | 4.36E-03 | 3.52E-02 | 0.41 | 1.51 | 0.76 |  |
| ALMS1 | 2 | p13.1 | 5.79E-03 | 4.34E-02 | 0.40 | 1.36 | 0.85 |  |
| ZNF638 | 2 | p13.2 | 3.12E-03 | 2.78E-02 | 0.42 | 1.63 | 0.87 | 1.63 |
| FAM136A | 2 | p13.3 | 6.41E-07 | 4.47E-05 | 0.65 | 1.84 | 0.88 | 3.31 |
| MPHOSPH10 | 2 | p13.3 | 8.34E-06 | 3.22E-04 | 0.60 | 3.14 | 0.94 | 3.14 |
| TEX261 | 2 | p13.3 | 2.37E-05 | 7.03E-04 | 0.57 | 1.81 | 0.82 | 1.81 |
| MCEE | 2 | p13.3 | 1.37E-04 | 2.72E-03 | 0.53 | 2.89 | 0.76 | 2.89 |
| TIA1 | 2 | p13.3 | 3.44E-04 | 5.47E-03 | 0.50 | 1.95 | 0.91 |  |
| PCYOX1 | 2 | p13.3 | 8.71E-04 | 1.09E-02 | 0.47 | 1.57 | 0.84 | 2.71 |
| PCBP1 | 2 | p13.3 | 1.56E-03 | 1.68E-02 | 0.45 | 1.69 | 0.98 |  |
| VAX2 | 2 | p13.3 | 3.99E-03 | 3.31E-02 | 0.41 | 2.00 | 0.96 | 2.00 |
| COMMD1 | 2 | p15 | 3.15E-03 | 2.80E-02 | 0.42 | 1.32 | 0.82 |  |
| PSME4 | 2 | p16.2 | 1.12E-03 | 1.31E-02 | 0.46 | 1.70 | 0.83 |  |
| RPL23AP32 | 2 | p16.2 | 2.71E-03 | 2.52E-02 | 0.43 | 1.09 | 0.84 |  |
| C2orf30 | 2 | p16.2 | 6.34E-03 | 4.64E-02 | 0.39 | 1.27 | 0.79 |  |
| MTA3 | 2 | p21 | 7.24E-07 | 4.92E-05 | 0.65 | 1.25 | 0.60 |  |
| EPCAM | 2 | p21 | 2.94E-03 | 2.67E-02 | 0.42 | 1.74 | 0.59 |  |
| EML4 | 2 | p21 | 5.82E-03 | 4.35E-02 | 0.40 | 1.06 | 0.86 |  |
| COX7A2L | 2 | p21 | 6.09E-03 | 4.49E-02 | 0.39 | 0.92 | 0.81 |  |
| TMEM178 | 2 | p22.1 | 4.56E-04 | 6.82E-03 | 0.49 | 1.43 | 0.89 |  |
| SOS1 | 2 | p22.1 | 5.76E-04 | 8.12E-03 | 0.48 | 1.30 | 0.90 |  |
| MRPL33 | 2 | p23.2 | 5.79E-03 | 4.33E-02 | 0.40 | 0.80 | 0.82 |  |
| AC013403.10 | 2 | p23.3 | 2.09E-06 | 1.10E-04 | 0.63 | 1.59 | 0.76 |  |
| SNX17 | 2 | p23.3 | 6.70E-05 | 1.57E-03 | 0.55 | 0.75 | 0.81 |  |
| MPV17 | 2 | p23.3 | 1.39E-04 | 2.74E-03 | 0.53 | 1.11 | 0.82 |  |
| ZNF512 | 2 | p23.3 | 4.12E-04 | 6.30E-03 | 0.49 | 1.18 | 0.83 |  |
| PREB | 2 | p23.3 | 4.21E-04 | 6.40E-03 | 0.49 | 1.35 | 0.87 |  |
| PPM1G | 2 | p23.3 | 8.77E-04 | 1.10E-02 | 0.47 | 0.99 | 0.83 |  |
| C2orf28 | 2 | p23.3 | 1.32E-03 | 1.48E-02 | 0.45 | 0.98 | 0.85 |  |
| EIF2B4 | 2 | p23.3 | 2.11E-03 | 2.11E-02 | 0.44 | 1.40 | 0.87 |  |
| DNAJC27 | 2 | p23.3 | 2.17E-03 | 2.15E-02 | 0.44 | 1.25 | 0.86 |  |
| UBXN2A | 2 | p23.3 | 4.64E-03 | 3.68E-02 | 0.41 | 1.18 | 0.83 |  |
| TMEM214 | 2 | p23.3 | 4.94E-03 | 3.85E-02 | 0.40 | 1.05 | 0.79 |  |
| AC010896.3 | 2 | p23.3 | 5.10E-03 | 3.95E-02 | 0.40 | 1.71 | 0.86 |  |
| DDX1 | 2 | p24.3 | 1.03E-03 | 1.23E-02 | 0.46 | 1.29 | 0.87 |  |
| ASAP2 | 2 | p25.1 | 1.90E-05 | 5.91E-04 | 0.58 | 1.70 | 0.68 |  |
| YWHAQ | 2 | p25.1 | 3.38E-05 | 9.36E-04 | 0.57 | 2.23 | 0.71 |  |
| CPSF3 | 2 | p25.1 | 3.82E-05 | 1.02E-03 | 0.56 | 1.38 | 0.83 |  |
| KLF11 | 2 | p25.1 | 9.05E-05 | 1.98E-03 | 0.54 | 1.78 | 0.82 |  |
| HPCAL1 | 2 | p25.1 | 3.02E-04 | 4.95E-03 | 0.50 | 1.89 | 0.85 |  |
| LPIN1 | 2 | p25.1 | 1.20E-03 | 1.38E-02 | 0.46 | 1.57 | 0.78 |  |
| ROCK2 | 2 | p25.1 | 1.69E-03 | 1.78E-02 | 0.45 | 1.21 | 0.80 |  |
| TSSC1 | 2 | p25.3 | 4.83E-05 | 1.22E-03 | 0.56 | 1.27 | 0.78 |  |
| COLEC11 | 2 | p25.3 | 1.11E-03 | 1.31E-02 | 0.46 | 1.11 | 0.87 |  |
| RNASEH1 | 2 | p25.3 | 4.50E-03 | 3.61E-02 | 0.41 | 1.19 | 0.81 |  |
| TMEM18 | 2 | p25.3 | 6.12E-03 | 4.51E-02 | 0.39 | 1.27 | 0.88 |  |
| C2orf64 | 2 | q11.2 | 2.33E-09 | 5.22E-07 | 0.74 | 2.71 | 0.77 | 4.21 |
| TBC1D8 | 2 | q11.2 | 6.50E-08 | 7.50E-06 | 0.69 | 1.97 | 0.73 | 4.30 |
| PDCL3 | 2 | q11.2 | 8.57E-08 | 9.42E-06 | 0.69 | 2.49 | 0.63 | 5.30 |
| COX5B | 2 | q11.2 | 2.51E-07 | 2.22E-05 | 0.67 | 1.45 | 0.71 | 2.76 |
| C2orf29 | 2 | q11.2 | 8.17E-07 | 5.40E-05 | 0.65 | 1.95 | 0.69 | 3.17 |
| FAM178B | 2 | q11.2 | 9.94E-07 | 6.32E-05 | 0.64 | 4.27 | 0.81 | 9.26 |
| ACTR1B | 2 | q11.2 | 2.32E-06 | 1.18E-04 | 0.63 | 1.68 | 0.70 | 2.93 |
| CHST10 | 2 | q11.2 | 4.65E-06 | 2.02E-04 | 0.61 | 1.74 | 0.81 | 4.42 |
| EIF5B | 2 | q11.2 | 8.40E-06 | 3.23E-04 | 0.60 | 1.62 | 0.78 | 1.83 |
| INPP4A | 2 | q11.2 | 2.72E-05 | 7.87E-04 | 0.57 | 1.24 | 0.90 | 1.37 |
| UNC50 | 2 | q11.2 | 3.19E-05 | 8.96E-04 | 0.57 | 1.56 | 0.79 | 2.32 |
| C2orf15 | 2 | q11.2 | 9.84E-05 | 2.11E-03 | 0.54 | 1.75 | 0.73 | 3.27 |
| LONRF2 | 2 | q11.2 | 1.07E-04 | 2.25E-03 | 0.53 | 1.85 | 0.69 | 5.36 |
| STARD7 | 2 | q11.2 | 2.16E-04 | 3.84E-03 | 0.51 | 1.36 | 0.79 |  |
| RNF149 | 2 | q11.2 | 2.48E-04 | 4.26E-03 | 0.51 | 1.56 | 0.77 | 1.99 |
| AC005035.2 | 2 | q11.2 | 2.62E-04 | 4.44E-03 | 0.51 | 1.30 | 0.99 | 3.26 |
| AFF3 | 2 | q11.2 | 7.80E-04 | 1.01E-02 | 0.47 | 2.63 | 0.82 | 3.08 |
| LMAN2L | 2 | q11.2 | 2.00E-03 | 2.03E-02 | 0.44 | 1.12 | 0.77 |  |
| LYG2 | 2 | q11.2 | 3.30E-03 | 2.89E-02 | 0.42 | 1.13 | 1.00 | 1.21 |
| AC008268.3 | 2 | q11.2 | 6.10E-03 | 4.50E-02 | 0.39 | 1.93 | 0.71 | 1.66 |
| KIAA1310 | 2 | q11.2 | 6.68E-03 | 4.80E-02 | 0.39 | 1.35 | 0.81 |  |
| TMEM182 | 2 | q12.1 | 6.93E-04 | 9.20E-03 | 0.48 | 1.09 | 1.02 | 1.35 |
| C2orf49 | 2 | q12.1 | 9.50E-04 | 1.15E-02 | 0.47 | 1.50 | 0.87 | 1.72 |
| AC007278.2 | 2 | q12.1 | 4.87E-03 | 3.82E-02 | -0.40 | 0.94 | 1.05 | 0.87 |
| RANBP2 | 2 | q12.3 | 1.89E-03 | 1.95E-02 | 0.44 | 1.20 | 0.99 | 1.52 |
| AC019100.5 | 2 | q12.3 | 4.71E-03 | 3.72E-02 | -0.41 | 0.93 | 1.09 | 0.93 |
| PSD4 | 2 | q13 | 2.96E-08 | 4.17E-06 | 0.71 | 1.30 | 0.95 | 1.98 |
| TTL | 2 | q13 | 2.11E-06 | 1.11E-04 | 0.63 | 1.70 | 0.90 |  |
| SLC20A1 | 2 | q13 | 1.14E-04 | 2.36E-03 | 0.53 | 1.79 | 0.70 |  |
| CKAP2L | 2 | q13 | 1.46E-04 | 2.84E-03 | 0.53 | 2.54 | 0.77 | 3.58 |
| ZC3H8 | 2 | q13 | 2.28E-04 | 3.99E-03 | 0.51 | 1.78 | 0.93 |  |
| POLR1B | 2 | q13 | 4.03E-04 | 6.18E-03 | 0.50 | 1.27 | 0.93 |  |
| TMEM87B | 2 | q13 | 6.20E-03 | 4.55E-02 | 0.39 | 1.28 | 0.86 |  |
| DDX18 | 2 | q14.1 | 1.14E-03 | 1.33E-02 | 0.46 | 1.39 | 0.83 |  |
| ACTR3 | 2 | q14.1 | 4.17E-03 | 3.42E-02 | 0.41 | 2.15 | 0.67 |  |
| TMEM185B | 2 | q14.2 | 2.40E-04 | 4.15E-03 | 0.51 | 1.58 | 0.77 |  |
| TMEM177 | 2 | q14.2 | 3.94E-03 | 3.28E-02 | 0.41 | 1.26 | 0.82 |  |
| CLASP1 | 2 | q14.2-q14.3 | 2.88E-04 | 4.77E-03 | 0.51 | 1.15 | 0.80 |  |
| MKI67IP | 2 | q14.3 | 1.59E-03 | 1.69E-02 | 0.45 | 2.16 | 0.83 |  |
| WDR33 | 2 | q14.3 | 1.76E-03 | 1.84E-02 | 0.44 | 2.55 | 0.84 |  |
| ERCC3 | 2 | q14.3 | 2.65E-03 | 2.48E-02 | 0.43 | 1.31 | 0.89 |  |
| IWS1 | 2 | q14.3 | 4.90E-03 | 3.83E-02 | 0.40 | 1.10 | 0.91 |  |
| SFT2D3 | 2 | q14.3 | 4.92E-03 | 3.84E-02 | 0.40 | 1.52 | 0.87 |  |
| IMP4 | 2 | q21.1 | 1.76E-04 | 3.27E-03 | 0.52 |  | 0.71 |  |
| FAM168B | 2 | q21.1 | 1.63E-03 | 1.73E-02 | 0.45 |  | 0.81 |  |
| DARS | 2 | q21.3 | 1.21E-04 | 2.48E-03 | 0.53 |  | 0.74 |  |
| UBXD2 | 2 | q21.3 | 2.63E-04 | 4.46E-03 | 0.51 |  | 0.71 |  |
| HNMT | 2 | q22.1 | 3.77E-03 | 3.19E-02 | 0.41 | 1.19 | 0.68 |  |
| AC016723.4 | 2 | q24.3 | 3.68E-03 | 3.13E-02 | 0.42 | 1.04 | 0.93 |  |
| AC015976.3 | 2 | q31.1 | 2.77E-04 | 4.62E-03 | 0.51 | 1.11 | 0.90 |  |
| GORASP2 | 2 | q31.1 | 3.11E-03 | 2.78E-02 | 0.42 | 1.09 | 0.84 |  |
| ZAK | 2 | q31.1 | 3.91E-03 | 3.27E-02 | 0.41 | 1.45 | 0.78 |  |
| PHOSPHO2 | 2 | q31.1 | 4.77E-03 | 3.75E-02 | 0.40 | 1.01 | 0.79 |  |
| DFNB59 | 2 | q31.2 | 4.70E-03 | 3.71E-02 | 0.41 | 1.17 | 0.87 |  |
| FKBP7 | 2 | q31.2 | 6.59E-03 | 4.76E-02 | 0.39 | 1.06 | 0.93 |  |
| CERKL | 2 | q31.3 | 7.39E-04 | 9.66E-03 | 0.48 | 1.06 | 0.97 | 1.50 |
| SSFA2 | 2 | q31.3 | 1.39E-03 | 1.54E-02 | 0.45 | 1.26 | 0.73 | 7.35 |
| HIBCH | 2 | q32.2 | 6.90E-04 | 9.17E-03 | 0.48 | 1.07 | 0.70 |  |
| ORMDL1 | 2 | q32.2 | 1.95E-03 | 1.99E-02 | 0.44 | 1.02 | 0.82 |  |
| NIF3L1 | 2 | q33.1 | 1.48E-03 | 1.61E-02 | 0.45 | 1.07 | 0.77 |  |
| C2orf47 | 2 | q33.1 | 3.72E-03 | 3.16E-02 | 0.42 | 1.24 | 0.78 |  |
| ALS2CR4 | 2 | q33.1 | 6.34E-03 | 4.63E-02 | 0.39 | 1.67 | 0.81 |  |
| XRCC5 | 2 | q35 | 6.29E-06 | 2.57E-04 | 0.61 | 1.38 | 0.74 |  |
| TTLL4 | 2 | q35 | 5.03E-05 | 1.27E-03 | 0.56 | 2.93 | 0.82 |  |
| DNPEP | 2 | q35 | 8.94E-05 | 1.96E-03 | 0.54 | 1.76 | 0.85 |  |
| RPL37A | 2 | q35 | 6.01E-04 | 8.34E-03 | 0.48 | 1.22 | 0.94 |  |
| BCS1L | 2 | q35 | 1.25E-03 | 1.42E-02 | 0.46 | 1.59 | 0.84 |  |
| ZFAND2B | 2 | q35 | 1.49E-03 | 1.62E-02 | 0.45 | 1.62 | 0.88 |  |
| PECR | 2 | q35 | 1.71E-03 | 1.80E-02 | 0.45 | 1.13 | 0.73 |  |
| FAM134A | 2 | q35 | 2.61E-03 | 2.45E-02 | 0.43 | 1.76 | 0.97 |  |
| ANKZF1 | 2 | q35 | 3.02E-03 | 2.72E-02 | 0.42 | 1.44 | 0.83 |  |
| CTDSP1 | 2 | q35 | 3.91E-03 | 3.27E-02 | 0.41 | 1.33 | 0.91 |  |
| SMARCAL1 | 2 | q35 | 4.69E-03 | 3.71E-02 | 0.41 | 1.49 | 0.92 |  |
| TMBIM1 | 2 | q35 | 4.74E-03 | 3.74E-02 | 0.41 | 1.52 | 0.79 |  |
| DES | 2 | q35 | 4.85E-03 | 3.80E-02 | -0.40 | 0.93 | 1.16 |  |
| AAMP | 2 | q35 | 5.46E-03 | 4.15E-02 | 0.40 | 1.95 | 0.94 |  |
| MRPL44 | 2 | q36.1 | 3.02E-03 | 2.72E-02 | 0.42 | 1.27 | 0.79 |  |
| EIF4E2 | 2 | q37.1 | 1.96E-05 | 6.05E-04 | 0.58 |  | 0.81 |  |
| NDUFA10 | 2 | q37.3 | 2.19E-04 | 3.87E-03 | 0.51 |  | 0.73 |  |
| STK25 | 2 | q37.3 | 1.32E-03 | 1.48E-02 | 0.45 |  | 0.88 |  |
| PPP1R7 | 2 | q37.3 | 1.45E-03 | 1.58E-02 | 0.45 |  | 0.86 |  |
| KIF1A | 2 | q37.3 | 4.69E-03 | 3.71E-02 | 0.41 |  | 0.58 |  |
| ANO7 | 2 | q37.3 | 4.72E-03 | 3.73E-02 | 0.41 |  | 0.95 |  |
| SHQ1 | 3 | p13 | 1.27E-05 | 4.42E-04 | 0.59 | 1.48 | 0.78 |  |
| LRIG1 | 3 | p14.1 | 2.68E-06 | 1.31E-04 | 0.62 | 0.95 | 0.53 |  |
| SUCLG2 | 3 | p14.1 | 1.04E-05 | 3.78E-04 | 0.59 | 1.13 | 0.67 |  |
| THOC7 | 3 | p14.1 | 5.39E-04 | 7.69E-03 | 0.49 | 1.23 | 0.74 |  |
| UBA3 | 3 | p14.1 | 4.80E-03 | 3.77E-02 | 0.40 | 1.14 | 0.72 |  |
| ARF4 | 3 | p14.3 | 3.11E-05 | 8.77E-04 | 0.57 | 1.42 | 0.76 |  |
| PDHB | 3 | p14.3 | 2.34E-04 | 4.07E-03 | 0.51 | 1.14 | 0.70 |  |
| SLMAP | 3 | p14.3 | 3.84E-04 | 5.93E-03 | 0.50 | 1.23 | 0.70 |  |
| PDE12 | 3 | p14.3 | 4.81E-04 | 7.07E-03 | 0.49 | 1.41 | 0.78 |  |
| FLNB | 3 | p14.3 | 6.43E-04 | 8.78E-03 | 0.48 | 1.96 | 0.52 |  |
| CCDC66 | 3 | p14.3 | 1.43E-03 | 1.57E-02 | 0.45 | 1.26 | 0.77 |  |
| FAM116A | 3 | p14.3 | 2.91E-03 | 2.65E-02 | 0.42 | 1.23 | 0.73 |  |
| KCTD6 | 3 | p14.3 | 3.41E-03 | 2.96E-02 | 0.42 | 1.31 | 0.62 |  |
| C3orf63 | 3 | p14.3 | 6.00E-03 | 4.45E-02 | 0.40 | 1.06 | 0.90 |  |
| SPCS1 | 3 | p21.1 | 1.70E-05 | 5.45E-04 | 0.58 | 1.72 | 0.68 |  |
| PRKCD | 3 | p21.1 | 2.76E-04 | 4.62E-03 | 0.51 | 1.82 | 0.73 |  |
| GNL3 | 3 | p21.1 | 3.90E-04 | 6.01E-03 | 0.50 | 1.74 | 0.71 |  |
| TKT | 3 | p21.1 | 1.49E-03 | 1.62E-02 | 0.45 | 2.08 | 0.77 |  |
| BAP1 | 3 | p21.1 | 4.38E-03 | 3.54E-02 | 0.41 | 1.58 | 0.85 |  |
| GLT8D1 | 3 | p21.1 | 6.50E-03 | 4.71E-02 | 0.39 | 1.74 | 0.75 |  |
| MAPKAPK3 | 3 | p21.2 | 2.15E-06 | 1.12E-04 | 0.63 | 1.59 | 0.75 |  |
| RRP9 | 3 | p21.2 | 2.16E-04 | 3.85E-03 | 0.51 | 1.45 | 0.79 |  |
| ABHD14B | 3 | p21.2 | 3.49E-04 | 5.52E-03 | 0.50 | 1.16 | 0.70 |  |
| RBM15B | 3 | p21.2 | 5.93E-04 | 8.26E-03 | 0.48 | 1.39 | 0.76 |  |
| TEX264 | 3 | p21.2 | 9.50E-04 | 1.15E-02 | 0.47 | 1.12 | 0.67 |  |
| ARMET | 3 | p21.2 | 1.41E-03 | 1.55E-02 | 0.45 | 1.31 | 0.80 |  |
| ACY1 | 3 | p21.2 | 2.22E-03 | 2.18E-02 | 0.44 | 1.04 | 0.63 |  |
| WDR51A | 3 | p21.2 | 3.06E-03 | 2.75E-02 | 0.42 | 1.85 | 0.85 |  |
| RPL29 | 3 | p21.2 | 5.25E-03 | 4.04E-02 | 0.40 | 1.62 | 0.79 |  |
| CCDC51 | 3 | p21.31 | 1.64E-07 | 1.59E-05 | 0.68 | 1.50 | 0.76 |  |
| UQCRC1 | 3 | p21.31 | 8.69E-06 | 3.31E-04 | 0.60 | 1.17 | 0.70 |  |
| APEH | 3 | p21.31 | 1.05E-05 | 3.82E-04 | 0.59 | 1.50 | 0.77 |  |
| SETD2 | 3 | p21.31 | 1.32E-05 | 4.52E-04 | 0.59 | 1.66 | 0.79 |  |
| CCDC72 | 3 | p21.31 | 1.59E-05 | 5.19E-04 | 0.58 | 1.75 | 0.69 |  |
| IMPDH2 | 3 | p21.31 | 2.41E-05 | 7.14E-04 | 0.57 | 1.70 | 0.65 |  |
| TMEM115 | 3 | p21.31 | 2.44E-05 | 7.21E-04 | 0.57 | 1.29 | 0.74 |  |
| ATRIP | 3 | p21.31 | 4.40E-05 | 1.14E-03 | 0.56 | 1.28 | 0.74 |  |
| SLC26A6 | 3 | p21.31 | 4.65E-05 | 1.19E-03 | 0.56 | 3.04 | 0.75 |  |
| IFRD2 | 3 | p21.31 | 5.07E-05 | 1.28E-03 | 0.56 | 1.21 | 0.71 |  |
| TUSC2 | 3 | p21.31 | 5.64E-05 | 1.38E-03 | 0.55 | 1.07 | 0.83 |  |
| CCDC12 | 3 | p21.31 | 1.45E-04 | 2.83E-03 | 0.53 | 1.47 | 0.85 |  |
| GMPPB | 3 | p21.31 | 1.47E-04 | 2.85E-03 | 0.53 | 1.40 | 0.84 |  |
| FYCO1 | 3 | p21.31 | 1.85E-04 | 3.40E-03 | 0.52 | 2.24 | 0.74 |  |
| DAG1 | 3 | p21.31 | 2.01E-04 | 3.65E-03 | 0.52 | 1.46 | 0.77 |  |
| SMARCC1 | 3 | p21.31 | 2.06E-04 | 3.72E-03 | 0.52 | 2.26 | 0.76 |  |
| C3orf75 | 3 | p21.31 | 2.09E-04 | 3.74E-03 | 0.52 | 1.10 | 0.80 |  |
| ZDHHC3 | 3 | p21.31 | 2.25E-04 | 3.97E-03 | 0.51 | 1.10 | 0.80 |  |
| RNF123 | 3 | p21.31 | 2.67E-04 | 4.50E-03 | 0.51 | 1.49 | 0.80 |  |
| LZTFL1 | 3 | p21.31 | 3.73E-04 | 5.80E-03 | 0.50 | 1.34 | 0.66 |  |
| NICN1 | 3 | p21.31 | 4.81E-04 | 7.07E-03 | 0.49 | 1.67 | 0.85 |  |
| KLHL18 | 3 | p21.31 | 5.18E-04 | 7.47E-03 | 0.49 | 1.01 | 0.86 |  |
| ZMYND10 | 3 | p21.31 | 5.21E-04 | 7.50E-03 | 0.49 | 1.34 | 0.79 |  |
| PTPN23 | 3 | p21.31 | 6.59E-04 | 8.90E-03 | 0.48 | 0.99 | 0.79 |  |
| USP4 | 3 | p21.31 | 8.65E-04 | 1.08E-02 | 0.47 | 1.63 | 0.91 |  |
| EXOSC7 | 3 | p21.31 | 8.96E-04 | 1.11E-02 | 0.47 | 1.35 | 0.86 |  |
| IP6K2 | 3 | p21.31 | 1.39E-03 | 1.54E-02 | 0.45 | 1.25 | 0.77 |  |
| CYB561D2 | 3 | p21.31 | 1.53E-03 | 1.65E-02 | 0.45 | 1.19 | 0.81 |  |
| QARS | 3 | p21.31 | 2.22E-03 | 2.18E-02 | 0.44 | 1.35 | 0.87 |  |
| RHOA | 3 | p21.31 | 2.36E-03 | 2.28E-02 | 0.43 | 1.21 | 0.90 |  |
| LARS2 | 3 | p21.31 | 2.73E-03 | 2.53E-02 | 0.43 | 1.55 | 0.82 |  |
| SCAP | 3 | p21.31 | 3.54E-03 | 3.04E-02 | 0.42 | 1.36 | 0.88 |  |
| PFKFB4 | 3 | p21.31 | 3.91E-03 | 3.27E-02 | 0.41 | 2.03 | 0.83 |  |
| TMEM42 | 3 | p21.31 | 3.93E-03 | 3.27E-02 | 0.41 | 1.00 | 0.73 |  |
| CSPG5 | 3 | p21.31 | 4.85E-03 | 3.80E-02 | 0.40 | 1.36 | 0.93 |  |
| RASSF1 | 3 | p21.31 | 5.02E-03 | 3.90E-02 | 0.40 | 1.19 | 0.90 |  |
| WDR6 | 3 | p21.31 | 5.65E-03 | 4.26E-02 | 0.40 | 1.37 | 0.80 |  |
| ZNF621 | 3 | p22.1 | 1.22E-04 | 2.48E-03 | 0.53 | 1.48 | 0.73 |  |
| SLC25A38 | 3 | p22.1 | 1.93E-04 | 3.53E-03 | 0.52 | 1.82 | 0.77 |  |
| C3orf39 | 3 | p22.1 | 2.53E-04 | 4.32E-03 | 0.51 | 1.04 | 0.71 |  |
| SEC22C | 3 | p22.1 | 2.84E-04 | 4.72E-03 | 0.51 | 1.32 | 0.75 |  |
| RPSA | 3 | p22.1 | 2.19E-03 | 2.16E-02 | 0.44 | 1.17 | 0.89 |  |
| SS18L2 | 3 | p22.1 | 2.44E-03 | 2.34E-02 | 0.43 | 1.21 | 0.76 |  |
| EIF1B | 3 | p22.1 | 4.31E-03 | 3.49E-02 | 0.41 | 1.82 | 0.70 |  |
| RPL14 | 3 | p22.1 | 6.18E-03 | 4.54E-02 | 0.39 | 2.15 | 0.49 |  |
| LRRFIP2 | 3 | p22.2 | 7.46E-05 | 1.72E-03 | 0.55 | 1.30 | 0.68 |  |
| GORASP1 | 3 | p22.2 | 2.53E-04 | 4.32E-03 | 0.51 | 1.44 | 0.73 |  |
| EPM2AIP1 | 3 | p22.2 | 2.98E-04 | 4.90E-03 | 0.50 | 1.29 | 0.66 |  |
| MLH1 | 3 | p22.2 | 3.04E-04 | 4.98E-03 | 0.50 | 1.31 | 0.72 |  |
| OXSR1 | 3 | p22.2 | 8.63E-04 | 1.08E-02 | 0.47 | 1.31 | 0.73 |  |
| CTDSPL | 3 | p22.2 | 8.88E-04 | 1.10E-02 | 0.47 | 1.28 | 0.58 |  |
| AXUD1 | 3 | p22.2 | 8.98E-04 | 1.11E-02 | 0.47 | 1.23 | 0.68 |  |
| MYD88 | 3 | p22.2 | 3.36E-03 | 2.93E-02 | 0.42 | 1.22 | 0.90 |  |
| WDR48 | 3 | p22.2 | 3.75E-03 | 3.17E-02 | 0.41 | 1.53 | 0.83 |  |
| CNOT10 | 3 | p22.3 | 2.36E-06 | 1.20E-04 | 0.63 | 1.26 | 0.70 |  |
| GPD1L | 3 | p22.3 | 1.32E-04 | 2.65E-03 | 0.53 | 1.53 | 0.55 |  |
| UBP1 | 3 | p22.3 | 1.33E-04 | 2.65E-03 | 0.53 | 1.31 | 0.60 |  |
| GLB1 | 3 | p22.3 | 1.02E-03 | 1.22E-02 | 0.46 | 1.30 | 0.83 |  |
| DYNC1LI1 | 3 | p22.3 | 3.53E-03 | 3.03E-02 | 0.42 | 1.15 | 0.87 |  |
| CRTAP | 3 | p22.3 | 4.54E-03 | 3.63E-02 | 0.41 | 1.17 | 0.80 |  |
| CMC1 | 3 | p24.1 | 1.53E-03 | 1.65E-02 | 0.45 | 1.35 | 0.72 |  |
| AZI2 | 3 | p24.1 | 2.07E-03 | 2.08E-02 | 0.44 | 1.18 | 0.76 |  |
| NGLY1 | 3 | p24.2 | 2.16E-05 | 6.53E-04 | 0.58 | 1.28 | 0.69 |  |
| OXSM | 3 | p24.2 | 4.60E-04 | 6.85E-03 | 0.49 | 1.13 | 0.73 |  |
| NKIRAS1 | 3 | p24.2 | 5.88E-04 | 8.23E-03 | 0.48 | 1.21 | 0.71 |  |
| RAB5A | 3 | p24.3 | 6.37E-03 | 4.65E-02 | 0.39 | 1.21 | 0.70 |  |
| C3orf19 | 3 | p25.1 | 9.70E-06 | 3.60E-04 | 0.60 | 1.40 | 0.89 |  |
| NUP210 | 3 | p25.1 | 5.27E-04 | 7.56E-03 | 0.49 | 1.50 | 0.70 |  |
| ZFYVE20 | 3 | p25.1 | 6.79E-04 | 9.07E-03 | 0.48 | 1.27 | 0.75 |  |
| HDAC11 | 3 | p25.1 | 9.59E-04 | 1.16E-02 | 0.47 | 1.51 | 0.76 |  |
| DPH3 | 3 | p25.1 | 1.99E-03 | 2.02E-02 | 0.44 | 1.27 | 0.82 |  |
| EAF1 | 3 | p25.1 | 2.00E-03 | 2.03E-02 | 0.44 | 1.54 | 0.74 |  |
| HACL1 | 3 | p25.1 | 6.55E-03 | 4.74E-02 | 0.39 | 1.14 | 0.70 |  |
| RAF1 | 3 | p25.2 | 4.55E-04 | 6.80E-03 | 0.49 | 1.33 | 0.87 |  |
| RPL32 | 3 | p25.2 | 7.55E-04 | 9.83E-03 | 0.47 | 1.25 | 0.86 |  |
| TMEM40 | 3 | p25.2 | 6.21E-03 | 4.56E-02 | 0.39 | 1.17 | 0.94 |  |
| TATDN2 | 3 | p25.3 | 8.95E-07 | 5.82E-05 | 0.65 | 1.52 | 0.79 |  |
| SEC13L1 | 3 | p25.3 | 2.32E-04 | 4.04E-03 | 0.51 | 1.42 | 0.89 |  |
| MTMR14 | 3 | p25.3 | 6.53E-04 | 8.87E-03 | 0.48 | 1.43 | 0.87 |  |
| FANCD2 | 3 | p25.3 | 2.18E-03 | 2.16E-02 | 0.44 | 1.29 | 0.81 |  |
| THUMPD3 | 3 | p25.3 | 2.62E-03 | 2.45E-02 | 0.43 | 1.22 | 0.93 |  |
| ATG7 | 3 | p25.3 | 2.70E-03 | 2.51E-02 | 0.43 | 1.41 | 0.93 |  |
| CRELD1 | 3 | p25.3 | 3.71E-03 | 3.15E-02 | 0.42 | 1.35 | 0.80 |  |
| TADA3L | 3 | p25.3 | 5.53E-03 | 4.19E-02 | 0.40 | 1.38 | 0.88 |  |
| VHL | 3 | p25.3 | 6.87E-03 | 4.91E-02 | 0.39 | 1.22 | 0.84 |  |
| ARL8B | 3 | p26.1 | 1.44E-04 | 2.80E-03 | 0.53 | 1.46 | 0.54 |  |
| CRBN | 3 | p26.2 | 2.55E-03 | 2.41E-02 | 0.43 | 1.18 | 0.75 |  |
| TRNT1 | 3 | p26.2 | 4.02E-03 | 3.32E-02 | 0.41 | 1.13 | 0.70 |  |
| SUMF1 | 3 | p26.2-p26.1 | 7.80E-06 | 3.06E-04 | 0.60 | 1.36 | 0.53 |  |
| CHMP2B | 3 | q11.1 | 1.60E-03 | 1.71E-02 | 0.45 | 1.54 | 0.66 |  |
| CGGBP1 | 3 | q11.1 | 6.07E-03 | 4.49E-02 | 0.39 | 1.33 | 0.47 |  |
| EPHA6 | 3 | q11.2 | 2.77E-03 | 2.56E-02 | 0.43 | 1.04 | 0.89 |  |
| MINA | 3 | q11.2 | 4.16E-03 | 3.41E-02 | 0.41 | 1.32 | 0.76 |  |
| RG9MTD1 | 3 | q12.3 | 3.78E-03 | 3.19E-02 | 0.41 | 1.26 | 1.09 |  |
| ATG3 | 3 | q13.2 | 2.92E-03 | 2.65E-02 | 0.42 | 1.21 | 0.80 |  |
| ZDHHC23 | 3 | q13.31 | 3.59E-04 | 5.63E-03 | 0.50 | 1.39 | 0.72 |  |
| B4GALT4 | 3 | q13.32 | 4.14E-03 | 3.40E-02 | 0.41 | 1.30 | 0.77 |  |
| COX17 | 3 | q13.33 | 2.08E-04 | 3.74E-03 | 0.52 | 1.34 | 0.74 |  |
| C3orf1 | 3 | q13.33 | 2.72E-04 | 4.56E-03 | 0.51 | 1.30 | 0.83 |  |
| ILDR1 | 3 | q13.33 | 5.89E-04 | 8.23E-03 | 0.48 | 1.48 | 0.53 |  |
| GTF2E1 | 3 | q13.33 | 1.38E-03 | 1.53E-02 | 0.45 | 1.29 | 0.81 |  |
| GSK3B | 3 | q13.33 | 1.86E-03 | 1.92E-02 | 0.44 | 1.30 | 0.79 |  |
| GOLGB1 | 3 | q13.33 | 5.60E-03 | 4.23E-02 | 0.40 | 1.45 | 0.55 |  |
| LRRC58 | 3 | q13.33 | 6.84E-03 | 4.89E-02 | 0.39 | 1.28 | 0.80 |  |
| SEC22A | 3 | q21.1 | 8.56E-05 | 1.90E-03 | 0.54 | 1.48 | 0.83 |  |
| KPNA1 | 3 | q21.1 | 2.80E-04 | 4.67E-03 | 0.51 | 1.50 | 0.80 |  |
| CCDC58 | 3 | q21.1 | 1.81E-03 | 1.89E-02 | 0.44 | 1.55 | 0.77 |  |
| OSBPL11 | 3 | q21.2 | 1.00E-03 | 1.21E-02 | 0.46 | 1.37 | 0.88 |  |
| SNX4 | 3 | q21.2 | 4.53E-03 | 3.62E-02 | 0.41 | 1.61 | 0.75 |  |
| SLC41A3 | 3 | q21.2-q21.3 | 2.60E-03 | 2.44E-02 | 0.43 | 1.30 | 0.78 |  |
| RAB43 | 3 | q21.3 | 1.15E-05 | 4.08E-04 | 0.59 | 1.37 | 0.93 |  |
| COPG | 3 | q21.3 | 1.19E-05 | 4.18E-04 | 0.59 | 1.59 | 0.73 |  |
| C3orf37 | 3 | q21.3 | 1.61E-04 | 3.05E-03 | 0.52 | 1.78 | 0.95 |  |
| EEFSEC | 3 | q21.3 | 2.61E-04 | 4.42E-03 | 0.51 | 1.21 | 1.00 |  |
| ACAD9 | 3 | q21.3 | 2.93E-04 | 4.84E-03 | 0.51 | 1.35 | 0.85 |  |
| RPN1 | 3 | q21.3 | 5.45E-04 | 7.76E-03 | 0.49 | 1.39 | 0.91 |  |
| CHCHD6 | 3 | q21.3 | 2.04E-03 | 2.06E-02 | 0.44 | 1.40 | 0.96 |  |
| GPR175 | 3 | q21.3 | 2.56E-03 | 2.42E-02 | 0.43 | 1.23 | 0.81 |  |
| RUVBL1 | 3 | q21.3 | 3.19E-03 | 2.83E-02 | 0.42 | 1.48 | 0.90 |  |
| SEC61A1 | 3 | q21.3 | 3.36E-03 | 2.93E-02 | 0.42 | 1.37 | 0.76 |  |
| ABTB1 | 3 | q21.3 | 4.89E-03 | 3.82E-02 | 0.40 | 1.32 | 0.94 |  |
| RAB7A | 3 | q21.3 | 5.42E-03 | 4.13E-02 | 0.40 | 1.43 | 0.83 |  |
| SRPRB | 3 | q22.1 | 2.23E-04 | 3.94E-03 | 0.51 | 1.29 | 0.85 |  |
| TOPBP1 | 3 | q22.1 | 3.81E-04 | 5.91E-03 | 0.50 | 1.63 | 0.55 |  |
| PIK3R4 | 3 | q22.1 | 8.17E-04 | 1.04E-02 | 0.47 | 1.30 | 0.79 |  |
| ASTE1 | 3 | q22.1 | 2.93E-03 | 2.66E-02 | 0.42 | 1.27 | 0.90 |  |
| NEK11 | 3 | q22.1 | 3.40E-03 | 2.95E-02 | 0.42 | 1.11 | 0.78 |  |
| MRPL3 | 3 | q22.1 | 4.11E-03 | 3.38E-02 | 0.41 | 1.35 | 0.81 |  |
| RAB6B | 3 | q22.1 | 4.86E-03 | 3.81E-02 | 0.40 | 1.21 | 1.02 |  |
| ATP2C1 | 3 | q22.1 | 5.94E-03 | 4.41E-02 | 0.40 | 1.32 | 0.96 |  |
| KY | 3 | q22.2 | 2.39E-04 | 4.14E-03 | 0.51 | 1.34 | 0.99 |  |
| ANAPC13 | 3 | q22.2 | 1.10E-03 | 1.29E-02 | 0.46 | 1.68 | 0.71 |  |
| CEP70 | 3 | q22.3 | 5.31E-03 | 4.08E-02 | 0.40 | 1.47 | 0.76 |  |
| MRPS22 | 3 | q23 | 7.71E-04 | 9.98E-03 | 0.47 | 1.14 | 0.81 |  |
| COPB2 | 3 | q23 | 1.16E-03 | 1.35E-02 | 0.46 | 1.51 | 0.71 |  |
| AC130416.4-1 | 3 | q23 | 6.34E-03 | 4.63E-02 | -0.39 | 0.94 | 1.07 |  |
| AADACL2 | 3 | q25.1 | 2.22E-03 | 2.18E-02 | -0.44 | 0.96 | 1.70 |  |
| DHX36 | 3 | q25.2 | 2.08E-03 | 2.08E-02 | 0.44 | 1.13 | 0.80 |  |
| PLCH1 | 3 | q25.31 | 1.33E-04 | 2.65E-03 | 0.53 | 1.36 | 0.76 |  |
| GMPS | 3 | q25.31 | 8.43E-04 | 1.06E-02 | 0.47 | 1.31 | 0.65 |  |
| SLC33A1 | 3 | q25.31 | 2.06E-03 | 2.07E-02 | 0.44 | 1.30 | 0.90 |  |
| RSRC1 | 3 | q25.32 | 8.60E-06 | 3.29E-04 | 0.60 | 1.36 | 0.78 |  |
| PLD1 | 3 | q26.31 | 8.12E-06 | 3.15E-04 | 0.60 | 1.08 |  | 1.87 |
| AADACL1 | 3 | q26.31 | 5.50E-05 | 1.35E-03 | 0.55 | 1.41 |  |  |
| TNFSF10 | 3 | q26.31 | 1.12E-03 | 1.32E-02 | 0.46 | 1.11 |  |  |
| PIK3CA | 3 | q26.32 | 9.81E-04 | 1.19E-02 | 0.47 | 1.17 | 0.67 |  |
| MFN1 | 3 | q26.33 | 4.10E-03 | 3.38E-02 | 0.41 | 1.10 | 0.94 |  |
| KLHL24 | 3 | q27.1 | 3.80E-04 | 5.91E-03 | 0.50 | 1.32 | 0.84 |  |
| ECE2 | 3 | q27.1 | 1.49E-03 | 1.62E-02 | 0.45 | 1.73 | 0.77 |  |
| POLR2H | 3 | q27.1 | 3.46E-03 | 2.99E-02 | 0.42 | 1.61 | 0.93 |  |
| CAMK2N2 | 3 | q27.1 | 3.55E-03 | 3.04E-02 | 0.42 | 1.72 | 0.89 |  |
| PSMD2 | 3 | q27.1 | 4.64E-03 | 3.68E-02 | 0.41 | 1.29 | 0.85 |  |
| SFRS10 | 3 | q27.2 | 3.41E-03 | 2.96E-02 | 0.42 | 1.49 | 0.41 |  |
| ETV5 | 3 | q27.2-q27.3 | 7.05E-03 | 4.99E-02 | -0.39 | 0.95 | 1.09 |  |
| DNAJB11 | 3 | q27.3 | 2.94E-04 | 4.85E-03 | 0.51 | 1.72 | 0.77 |  |
| RFC4 | 3 | q27.3 | 7.69E-04 | 9.96E-03 | 0.47 | 1.46 | 0.83 |  |
| SENP5 | 3 | q29 | 3.17E-04 | 5.14E-03 | 0.50 | 1.45 | 0.98 |  |
| FYTTD1 | 3 | q29 | 4.58E-04 | 6.83E-03 | 0.49 | 1.97 | 0.68 |  |
| IQCG | 3 | q29 | 8.34E-04 | 1.05E-02 | 0.47 | 2.11 | 0.74 |  |
| TFRC | 3 | q29 | 1.44E-03 | 1.57E-02 | 0.45 | 1.83 | 0.58 |  |
| WDR53 | 3 | q29 | 2.51E-03 | 2.38E-02 | 0.43 | 1.26 |  |  |
| APOD | 3 | q29 | 3.11E-03 | 2.78E-02 | -0.42 | 0.36 | 4.62 |  |
| PCYT1A | 3 | q29 | 4.54E-03 | 3.63E-02 | 0.41 | 1.05 | 0.97 |  |
| LSG1 | 3 | q29 | 4.57E-03 | 3.64E-02 | 0.41 | 1.45 | 0.87 |  |
| PAK2 | 3 | q29 | 4.87E-03 | 3.82E-02 | 0.40 | 1.29 |  |  |
| OCIAD2 | 4 | p11 | 1.06E-03 | 1.26E-02 | 0.46 | 1.22 | 0.60 |  |
| NFXL1 | 4 | p12 | 3.23E-03 | 2.85E-02 | 0.42 | 1.07 | 0.69 |  |
| GNPDA2 | 4 | p12 | 4.18E-03 | 3.42E-02 | 0.41 | 1.24 | 0.78 |  |
| SLC30A9 | 4 | p13 | 1.75E-05 | 5.53E-04 | 0.58 | 1.52 | 0.70 |  |
| TMEM33 | 4 | p13 | 1.94E-03 | 1.99E-02 | 0.44 | 2.04 | 0.79 |  |
| RFC1 | 4 | p14 | 4.28E-04 | 6.46E-03 | 0.49 | 1.39 | 0.86 |  |
| LIAS | 4 | p14 | 1.32E-03 | 1.48E-02 | 0.45 | 1.18 | 0.81 |  |
| UBE2K | 4 | p14 | 2.86E-03 | 2.62E-02 | 0.43 | 1.42 | 0.72 |  |
| WDR19 | 4 | p14 | 6.16E-03 | 4.53E-02 | 0.39 | 1.10 | 0.66 |  |
| AC007073.2 | 4 | p15.2 | 6.10E-04 | 8.44E-03 | 0.48 | 1.23 | 0.85 |  |
| RBPSUH | 4 | p15.2 | 1.84E-03 | 1.91E-02 | 0.44 | 1.21 | 0.77 |  |
| AC114744.7-1 | 4 | p15.32 | 9.01E-04 | 1.11E-02 | 0.47 | 1.25 | 0.83 |  |
| AC110088.5 | 4 | p15.32 | 6.10E-03 | 4.50E-02 | 0.39 | 1.23 | 0.78 |  |
| RAB28 | 4 | p15.33 | 4.15E-03 | 3.40E-02 | 0.41 | 1.05 | 0.74 |  |
| MRFAP1L1 | 4 | p16.1 | 9.45E-06 | 3.52E-04 | 0.60 | 1.31 | 0.80 |  |
| TBC1D14 | 4 | p16.1 | 5.16E-04 | 7.46E-03 | 0.49 | 1.01 | 0.80 |  |
| CNO | 4 | p16.1 | 5.88E-04 | 8.23E-03 | 0.48 | 1.34 | 0.90 |  |
| AC093323.3 | 4 | p16.1 | 7.15E-04 | 9.42E-03 | 0.48 | 1.30 | 0.77 |  |
| TETRAN | 4 | p16.3 | 1.99E-05 | 6.10E-04 | 0.58 | 1.43 | 0.86 |  |
| TNIP2 | 4 | p16.3 | 2.68E-03 | 2.50E-02 | 0.43 | 1.28 | 0.88 |  |
| MAEA | 4 | p16.3 | 3.56E-03 | 3.05E-02 | 0.42 | 1.36 | 0.92 |  |
| USP46 | 4 | q12 | 3.94E-04 | 6.05E-03 | 0.50 | 1.12 | 0.75 |  |
| C4orf14 | 4 | q12 | 1.29E-03 | 1.46E-02 | 0.46 | 1.17 | 0.80 |  |
| FIP1L1 | 4 | q12 | 1.68E-03 | 1.77E-02 | 0.45 | 1.22 | 0.68 |  |
| AASDH | 4 | q12 | 1.81E-03 | 1.88E-02 | 0.44 | 1.01 | 0.74 |  |
| LNX1 | 4 | q12 | 2.06E-03 | 2.07E-02 | 0.44 | 1.14 | 0.93 |  |
| PAICS | 4 | q12 | 6.49E-03 | 4.71E-02 | 0.39 | 1.47 | 0.84 |  |
| CENPC1 | 4 | q13.2 | 1.04E-03 | 1.24E-02 | 0.46 | 1.12 | 0.83 |  |
| MTHFD2L | 4 | q13.3 | 2.90E-06 | 1.39E-04 | 0.62 | 1.36 | 0.72 |  |
| ANKRD17 | 4 | q13.3 | 5.91E-06 | 2.44E-04 | 0.61 | 1.15 | 0.73 |  |
| NPFFR2 | 4 | q13.3 | 1.39E-04 | 2.74E-03 | 0.53 | 1.13 | 0.90 |  |
| CSN2 | 4 | q13.3 | 2.71E-04 | 4.54E-03 | 0.51 | 1.05 | 0.91 |  |
| GRSF1 | 4 | q13.3 | 8.16E-04 | 1.03E-02 | 0.47 | 1.18 | 0.66 |  |
| MRPL1 | 4 | q21.1 | 8.85E-05 | 1.94E-03 | 0.54 | 1.33 | 0.81 | 3.06 |
| SDAD1 | 4 | q21.1 | 1.13E-04 | 2.35E-03 | 0.53 | 1.25 | 0.82 |  |
| USO1 | 4 | q21.1 | 1.48E-03 | 1.61E-02 | 0.45 | 1.13 | 0.66 |  |
| G3BP2 | 4 | q21.1 | 1.92E-03 | 1.97E-02 | 0.44 | 1.24 | 0.78 |  |
| SCARB2 | 4 | q21.1 | 1.96E-03 | 2.00E-02 | 0.44 | 1.19 | 0.78 |  |
| NUP54 | 4 | q21.1 | 3.94E-03 | 3.28E-02 | 0.41 | 1.14 | 0.75 |  |
| SHROOM3 | 4 | q21.1 | 4.53E-03 | 3.62E-02 | 0.41 | 1.15 | 0.60 |  |
| CCNI | 4 | q21.1 | 4.68E-03 | 3.71E-02 | 0.41 | 1.22 | 0.83 |  |
| PAQR3 | 4 | q21.21 | 1.72E-05 | 5.47E-04 | 0.58 | 1.36 | 0.81 | 2.72 |
| FRAS1 | 4 | q21.21 | 4.53E-05 | 1.17E-03 | 0.56 | 1.42 | 0.81 | 5.57 |
| COPS4 | 4 | q21.22 | 1.89E-06 | 1.03E-04 | 0.63 | 1.25 | 0.65 | 2.58 |
| ENOPH1 | 4 | q21.22 | 6.59E-04 | 8.91E-03 | 0.48 | 1.33 | 0.79 | 2.34 |
| HNRPDL | 4 | q21.22 | 1.57E-03 | 1.68E-02 | 0.45 | 1.19 | 0.75 | 1.63 |
| THAP9 | 4 | q21.22 | 3.32E-03 | 2.91E-02 | 0.42 | 1.03 | 0.89 | 1.17 |
| WDFY3 | 4 | q21.23 | 1.55E-05 | 5.13E-04 | 0.59 | 1.19 | 0.84 | 1.76 |
| MRPS18C | 4 | q21.23 | 1.31E-04 | 2.62E-03 | 0.53 | 1.20 | 0.64 | 2.67 |
| CDS1 | 4 | q21.23 | 3.14E-04 | 5.09E-03 | 0.50 | 1.36 | 0.65 | 2.75 |
| NUDT9 | 4 | q22.1 | 1.69E-04 | 3.18E-03 | 0.52 | 1.38 | 0.70 |  |
| KLHL8 | 4 | q22.1 | 2.09E-03 | 2.09E-02 | 0.44 | 1.27 | 0.80 |  |
| PIG-Y | 4 | q22.1 | 6.19E-03 | 4.55E-02 | 0.39 | 1.14 | 0.70 |  |
| GRID2 | 4 | q22.1-q22.2 | 4.15E-03 | 3.40E-02 | 0.41 | 1.06 | 0.98 |  |
| METAP1 | 4 | q23 | 6.64E-04 | 8.94E-03 | 0.48 | 1.19 | 0.75 |  |
| RG9MTD2 | 4 | q23 | 4.97E-03 | 3.86E-02 | 0.40 | 1.11 | 0.84 |  |
| AC004053.1 | 4 | q24 | 4.34E-06 | 1.92E-04 | 0.61 | 1.19 | 0.70 |  |
| INTS12 | 4 | q24 | 1.45E-05 | 4.86E-04 | 0.59 | 1.14 | 0.66 |  |
| CISD2 | 4 | q24 | 6.46E-03 | 4.69E-02 | 0.39 | 1.11 | 0.76 |  |
| HADH | 4 | q25 | 5.79E-04 | 8.15E-03 | 0.48 | 1.11 | 0.52 |  |
| SEC24B | 4 | q25 | 2.00E-03 | 2.03E-02 | 0.44 | 1.15 | 0.92 |  |
| PAPSS1 | 4 | q25 | 4.23E-03 | 3.45E-02 | 0.41 | 1.22 | 0.76 |  |
| C4orf16 | 4 | q25 | 6.10E-03 | 4.50E-02 | 0.39 | 1.17 | 0.60 |  |
| TRAM1L1 | 4 | q26 | 5.44E-03 | 4.14E-02 | 0.40 | 1.20 | 0.91 |  |
| EXOSC9 | 4 | q27 | 1.50E-03 | 1.62E-02 | 0.45 | 1.20 | 0.72 |  |
| INTU | 4 | q28.1 | 6.59E-04 | 8.90E-03 | 0.48 | 1.13 | 0.87 |  |
| PGRMC2 | 4 | q28.2 | 1.42E-04 | 2.79E-03 | 0.53 | 1.26 | 0.53 |  |
| PHF17 | 4 | q28.2 | 1.17E-03 | 1.36E-02 | 0.46 | 1.19 | 0.65 |  |
| LARP2 | 4 | q28.2 | 3.08E-03 | 2.76E-02 | 0.42 | 1.14 | 0.79 |  |
| ELF2 | 4 | q31.1 | 1.39E-04 | 2.74E-03 | 0.53 | 1.13 | 0.69 |  |
| NARG1 | 4 | q31.1 | 1.49E-03 | 1.62E-02 | 0.45 | 1.18 | 0.69 |  |
| SCOC | 4 | q31.1 | 4.19E-03 | 3.42E-02 | 0.41 | 1.23 | 0.61 |  |
| ANAPC10P | 4 | q31.21 | 1.36E-04 | 2.70E-03 | 0.53 | 1.17 | 0.66 |  |
| ZNF330 | 4 | q31.21 | 1.80E-04 | 3.32E-03 | 0.52 | 1.23 | 0.69 |  |
| ABCE1 | 4 | q31.21 | 1.89E-03 | 1.95E-02 | 0.44 | 1.23 | 0.68 |  |
| TBC1D9 | 4 | q31.21 | 5.92E-03 | 4.41E-02 | 0.40 | 1.43 | 0.35 |  |
| PRMT10 | 4 | q31.23 | 5.50E-04 | 7.82E-03 | 0.49 | 1.18 | 0.80 |  |
| PLRG1 | 4 | q31.3 | 1.61E-04 | 3.05E-03 | 0.52 | 1.15 | 0.72 |  |
| PET112L | 4 | q31.3 | 2.98E-04 | 4.90E-03 | 0.50 | 1.28 | 0.78 |  |
| FHDC1 | 4 | q31.3 | 2.34E-03 | 2.26E-02 | 0.43 | 1.65 | 0.86 |  |
| ARFIP1 | 4 | q31.3 | 3.51E-03 | 3.02E-02 | 0.42 | 1.26 | 0.73 |  |
| SH3D19 | 4 | q31.3 | 4.76E-03 | 3.75E-02 | 0.40 | 1.01 | 0.63 |  |
| TMEM144 | 4 | q32.1 | 2.81E-04 | 4.67E-03 | 0.51 | 1.27 | 0.47 |  |
| ETFDH | 4 | q32.1 | 4.10E-04 | 6.27E-03 | 0.49 | 1.13 | 0.64 |  |
| CBR4 | 4 | q32.3 | 3.27E-06 | 1.53E-04 | 0.62 | 1.39 | 0.63 |  |
| TMEM192 | 4 | q32.3 | 4.18E-03 | 3.42E-02 | 0.41 | 1.16 | 0.81 |  |
| SH3RF1 | 4 | q32.3-q33 | 3.06E-03 | 2.74E-02 | 0.42 | 1.39 | 0.78 |  |
| CLCN3 | 4 | q33 | 5.36E-05 | 1.33E-03 | 0.55 | 1.21 | 0.57 |  |
| AC106878.3 | 4 | q33 | 1.26E-04 | 2.55E-03 | 0.53 | 1.23 | 0.70 |  |
| FBXO8 | 4 | q34.1 | 4.74E-05 | 1.21E-03 | 0.56 | 1.26 | 0.69 |  |
| AGA | 4 | q34.3 | 7.88E-04 | 1.01E-02 | 0.47 | 1.22 | 0.49 |  |
| ING2 | 4 | q35.1 | 7.77E-05 | 1.78E-03 | 0.54 | 1.28 | 0.72 |  |
| C4orf41 | 4 | q35.1 | 2.20E-04 | 3.89E-03 | 0.51 | 1.23 | 0.78 |  |
| RWDD4A | 4 | q35.1 | 7.54E-04 | 9.83E-03 | 0.47 | 1.18 | 0.81 |  |
| UFSP2 | 4 | q35.1 | 1.11E-03 | 1.31E-02 | 0.46 | 1.09 | 0.66 |  |
| CASP3 | 4 | q35.1 | 1.58E-03 | 1.69E-02 | 0.45 | 1.16 | 0.70 |  |
| MLF1IP | 4 | q35.1 | 3.26E-03 | 2.87E-02 | 0.42 | 1.41 | 0.77 |  |
| DCTD | 4 | q35.1 | 4.57E-03 | 3.65E-02 | 0.41 | 1.17 | 0.82 |  |
| C4orf47 | 4 | q35.1 | 5.11E-03 | 3.95E-02 | 0.40 | 1.09 | 0.86 |  |
| CDKN2AIP | 4 | q35.1 | 6.91E-03 | 4.92E-02 | 0.39 | 1.07 | 0.83 |  |
| TUBB4Q | 4 | q35.2 | 5.05E-03 | 3.92E-02 | -0.40 | 0.89 | 1.14 |  |
| C5orf51 | 5 | p13.1 | 2.89E-06 | 1.39E-04 | 0.62 | 1.28 | 0.58 |  |
| WDR70 | 5 | p13.2 | 5.24E-04 | 7.53E-03 | 0.49 | 1.15 | 0.79 |  |
| DNAJA5 | 5 | p13.2 | 6.58E-04 | 8.90E-03 | 0.48 | 1.21 | 0.67 |  |
| RNASEN | 5 | p13.3 | 8.68E-06 | 3.31E-04 | 0.60 | 1.34 | 0.78 |  |
| TARS | 5 | p13.3 | 1.39E-04 | 2.74E-03 | 0.53 | 1.46 | 0.60 |  |
| AC138951.2-1 | 5 | p14.3 | 3.78E-04 | 5.88E-03 | 0.50 | 1.42 | 1.14 |  |
| ZNF622 | 5 | p15.1 | 2.04E-03 | 2.06E-02 | 0.44 | 1.10 | 0.78 |  |
| ROPN1L | 5 | p15.2 | 1.13E-03 | 1.32E-02 | 0.46 | 1.14 | 0.87 |  |
| CCT5 | 5 | p15.2 | 1.51E-03 | 1.64E-02 | 0.45 | 1.32 | 0.77 |  |
| FAM173B | 5 | p15.2 | 5.85E-03 | 4.37E-02 | 0.40 | 1.13 | 0.78 |  |
| NSUN2 | 5 | p15.31 | 2.34E-03 | 2.26E-02 | 0.43 | 1.20 | 0.78 |  |
| POLS | 5 | p15.31 | 5.01E-03 | 3.89E-02 | 0.40 | 1.20 | 0.79 |  |
| CEP72 | 5 | p15.33 | 3.81E-05 | 1.02E-03 | 0.56 | 1.15 | 0.89 |  |
| MRPL36 | 5 | p15.33 | 2.36E-04 | 4.10E-03 | 0.51 | 1.36 | 0.70 |  |
| ZDHHC11 | 5 | p15.33 | 4.61E-04 | 6.85E-03 | 0.49 | 1.07 | 0.95 |  |
| BRD9 | 5 | p15.33 | 1.18E-03 | 1.37E-02 | 0.46 | 1.22 | 0.85 |  |
| AC114291.2 | 5 | p15.33 | 2.27E-03 | 2.22E-02 | 0.43 | 1.31 | 0.88 |  |
| CCDC127 | 5 | p15.33 | 2.97E-03 | 2.69E-02 | 0.42 | 1.14 | 0.72 |  |
| TRIP13 | 5 | p15.33 | 6.66E-03 | 4.79E-02 | 0.39 | 1.58 | 0.64 |  |
| NDUFS6 | 5 | p15.33 | 6.91E-03 | 4.92E-02 | 0.39 | 1.11 | 0.73 |  |
| DHX29 | 5 | q11.2 | 2.06E-06 | 1.09E-04 | 0.63 | 1.43 | 0.61 |  |
| NDUFS4 | 5 | q11.2 | 3.96E-06 | 1.80E-04 | 0.62 | 1.40 | 0.57 |  |
| CDC20B | 5 | q11.2 | 4.19E-05 | 1.10E-03 | 0.56 | 1.21 | 0.96 |  |
| GPBP1 | 5 | q11.2 | 1.44E-04 | 2.80E-03 | 0.53 | 1.27 | 0.61 |  |
| PELO | 5 | q11.2 | 2.78E-04 | 4.64E-03 | 0.51 | 1.18 | 0.75 |  |
| ITGA2 | 5 | q11.2 | 3.42E-03 | 2.96E-02 | 0.42 | 1.34 | 0.78 |  |
| GAPT | 5 | q11.2 | 3.81E-03 | 3.20E-02 | 0.41 | 1.23 | 0.70 |  |
| KIF2A | 5 | q12.1 | 6.06E-05 | 1.46E-03 | 0.55 | 1.27 | 0.82 |  |
| NDUFAF2 | 5 | q12.1 | 3.51E-03 | 3.02E-02 | 0.42 | 1.17 | 0.68 |  |
| TRIM23 | 5 | q12.3 | 2.67E-04 | 4.49E-03 | 0.51 | 1.18 | 0.88 |  |
| C5orf44 | 5 | q12.3 | 3.82E-04 | 5.92E-03 | 0.50 | 1.23 | 0.81 |  |
| ERBB2IP | 5 | q12.3 | 9.46E-04 | 1.15E-02 | 0.47 | 1.08 | 0.95 |  |
| SDCCAG10 | 5 | q12.3 | 5.85E-03 | 4.37E-02 | 0.40 | 1.19 | 0.81 |  |
| PIK3R1 | 5 | q13.1 | 6.23E-03 | 4.57E-02 | 0.39 | 1.14 | 0.56 |  |
| SLC30A5 | 5 | q13.1-q13.2 | 1.85E-03 | 1.91E-02 | 0.44 | 1.28 | 0.74 |  |
| BIRC1 | 5 | q13.2 | 1.02E-06 | 6.41E-05 | 0.64 | 1.34 | 0.81 | 2.05 |
| MRPS27 | 5 | q13.2 | 1.35E-06 | 7.95E-05 | 0.64 | 1.81 | 0.61 |  |
| AC140134.2-1 | 5 | q13.2 | 2.26E-06 | 1.16E-04 | 0.63 | 1.40 | 0.81 | 1.43 |
| CDK7 | 5 | q13.2 | 7.90E-06 | 3.08E-04 | 0.60 | 1.22 | 0.73 |  |
| PTCD2 | 5 | q13.2 | 1.56E-05 | 5.15E-04 | 0.59 | 1.24 | 0.88 |  |
| SERF1B | 5 | q13.2 | 2.38E-05 | 7.06E-04 | 0.57 | 1.47 | 0.71 | 2.08 |
| OCLN | 5 | q13.2 | 6.66E-05 | 1.56E-03 | 0.55 | 1.47 | 0.67 |  |
| AC145146.2-1 | 5 | q13.2 | 2.08E-04 | 3.74E-03 | 0.52 | 1.62 | 0.70 | 1.60 |
| BTF3 | 5 | q13.2 | 3.13E-04 | 5.09E-03 | 0.50 | 1.96 | 0.54 |  |
| CCDC125 | 5 | q13.2 | 1.07E-03 | 1.27E-02 | 0.46 | 1.25 | 0.85 |  |
| RAD17 | 5 | q13.2 | 1.14E-03 | 1.33E-02 | 0.46 | 1.22 | 0.86 |  |
| AC140134.2-2 | 5 | q13.2 | 1.66E-03 | 1.76E-02 | 0.45 | 1.66 | 0.65 | 1.25 |
| MRPS36 | 5 | q13.2 | 4.75E-03 | 3.74E-02 | 0.40 | 1.18 | 0.84 |  |
| ANKRA2 | 5 | q13.2 | 6.33E-03 | 4.63E-02 | 0.39 | 1.14 | 0.76 |  |
| C5orf37 | 5 | q13.3 | 2.97E-06 | 1.42E-04 | 0.62 | 1.26 | 0.82 |  |
| TINP1 | 5 | q13.3 | 2.37E-04 | 4.12E-03 | 0.51 | 1.75 | 0.62 |  |
| HEXB | 5 | q13.3 | 4.15E-03 | 3.40E-02 | 0.41 | 1.05 | 0.75 |  |
| SERINC5 | 5 | q14.1 | 5.93E-05 | 1.44E-03 | 0.55 | 1.13 | 0.88 |  |
| MSH3 | 5 | q14.1 | 8.04E-04 | 1.03E-02 | 0.47 | 1.17 | 0.72 |  |
| AP3B1 | 5 | q14.1 | 1.35E-03 | 1.51E-02 | 0.45 | 1.22 | 0.73 |  |
| PAPD4 | 5 | q14.1 | 1.91E-03 | 1.96E-02 | 0.44 | 1.20 | 0.57 |  |
| JMY | 5 | q14.1 | 6.56E-03 | 4.75E-02 | 0.39 | 1.08 | 0.89 |  |
| ATP6AP1L | 5 | q14.2 | 5.10E-05 | 1.28E-03 | 0.56 | 1.28 | 0.88 | 1.50 |
| RPS23 | 5 | q14.2 | 3.93E-03 | 3.28E-02 | 0.41 | 1.26 | 0.72 | 2.85 |
| COX7 | 5 | q14.3 | 7.23E-07 | 4.92E-05 | 0.65 | 1.38 | 0.62 |  |
| GPR98 | 5 | q14.3 | 6.07E-03 | 4.49E-02 | 0.39 | 1.43 | 0.78 |  |
| TTC37 | 5 | q15 | 1.48E-06 | 8.57E-05 | 0.64 | 1.33 | 0.66 |  |
| RFESD | 5 | q15 | 1.27E-04 | 2.57E-03 | 0.53 | 1.09 | 0.89 |  |
| FAM172A | 5 | q15 | 7.77E-04 | 1.00E-02 | 0.47 | 1.19 | 0.75 |  |
| RIOK2 | 5 | q15 | 2.87E-03 | 2.63E-02 | 0.43 | 1.22 | 0.70 |  |
| GLRX | 5 | q15 | 3.05E-03 | 2.74E-02 | 0.42 | 1.55 | 0.57 |  |
| C5orf36 | 5 | q15 | 3.56E-03 | 3.05E-02 | 0.42 | 1.04 | 0.91 |  |
| ARSK | 5 | q15 | 3.78E-03 | 3.19E-02 | 0.41 | 1.05 | 0.94 |  |
| FAM174A | 5 | q21.1 | 5.41E-03 | 4.13E-02 | 0.40 | 1.43 | 0.63 |  |
| YTHDC2 | 5 | q22.2 | 9.02E-04 | 1.11E-02 | 0.47 | 1.12 | 0.84 |  |
| SRP19 | 5 | q22.2 | 1.58E-03 | 1.69E-02 | 0.45 | 1.24 | 0.77 |  |
| MCC | 5 | q22.2 | 2.89E-03 | 2.64E-02 | 0.43 | 1.12 | 0.95 |  |
| CCDC112 | 5 | q22.3 | 3.42E-03 | 2.96E-02 | 0.42 | 1.21 | 0.83 |  |
| HSD17B4 | 5 | q23.1 | 4.25E-03 | 3.46E-02 | 0.41 | 1.19 | 0.84 |  |
| PHAX | 5 | q23.2 | 1.06E-03 | 1.26E-02 | 0.46 | 1.23 | 0.87 |  |
| Mar-03 | 5 | q23.2 | 3.01E-03 | 2.71E-02 | 0.42 | 1.35 | 0.87 |  |
| HINT1 | 5 | q23.3 | 1.32E-04 | 2.65E-03 | 0.53 | 1.33 | 0.64 |  |
| UQCRQ | 5 | q31.1 | 4.58E-07 | 3.52E-05 | 0.66 | 1.26 | 0.65 |  |
| RAD50 | 5 | q31.1 | 2.98E-05 | 8.47E-04 | 0.57 | 1.22 | 0.76 |  |
| C5orf15 | 5 | q31.1 | 5.07E-05 | 1.28E-03 | 0.56 | 1.20 | 0.67 |  |
| DDX46 | 5 | q31.1 | 5.31E-05 | 1.32E-03 | 0.55 | 1.34 | 0.72 |  |
| SLC22A5 | 5 | q31.1 | 1.01E-04 | 2.15E-03 | 0.54 | 1.33 | 0.73 |  |
| H2AFY | 5 | q31.1 | 3.35E-04 | 5.36E-03 | 0.50 | 1.30 | 0.72 |  |
| AFF4 | 5 | q31.1 | 4.00E-04 | 6.14E-03 | 0.50 | 1.56 | 0.71 |  |
| HSPA4 | 5 | q31.1 | 4.24E-04 | 6.42E-03 | 0.49 | 1.37 | 0.84 |  |
| RAPGEF6 | 5 | q31.1 | 8.81E-04 | 1.10E-02 | 0.47 | 1.37 | 0.74 |  |
| C5orf24 | 5 | q31.1 | 1.02E-03 | 1.23E-02 | 0.46 | 1.20 | 0.85 |  |
| SKP1A | 5 | q31.1 | 1.55E-03 | 1.67E-02 | 0.45 | 1.34 | 0.70 |  |
| VDAC1 | 5 | q31.1 | 1.81E-03 | 1.88E-02 | 0.44 | 1.25 | 0.78 |  |
| FNIP1 | 5 | q31.1 | 2.73E-03 | 2.53E-02 | 0.43 | 1.20 | 0.71 |  |
| SHROOM1 | 5 | q31.1 | 3.77E-03 | 3.19E-02 | 0.41 | 1.56 | 0.51 |  |
| PHF15 | 5 | q31.1 | 4.10E-03 | 3.37E-02 | 0.41 | 1.40 | 0.71 |  |
| SLC22A4 | 5 | q31.1 | 4.57E-03 | 3.65E-02 | 0.41 | 1.18 | 0.95 |  |
| CTNNA1 | 5 | q31.2 | 9.34E-07 | 6.03E-05 | 0.65 | 1.43 | 0.69 |  |
| ETF1 | 5 | q31.2 | 3.10E-04 | 5.05E-03 | 0.50 | 1.33 | 0.73 |  |
| AC135457.2-3 | 5 | q31.2 | 4.68E-04 | 6.92E-03 | 0.49 | 1.08 | 0.88 |  |
| KDM3B | 5 | q31.2 | 1.67E-03 | 1.77E-02 | 0.45 | 1.22 | 0.74 |  |
| BRD8 | 5 | q31.2 | 2.13E-03 | 2.12E-02 | 0.44 | 1.29 | 0.81 |  |
| UBE2D2 | 5 | q31.2 | 3.67E-03 | 3.12E-02 | 0.42 | 0.99 | 0.81 |  |
| NDUFA2 | 5 | q31.3 | 2.37E-09 | 5.27E-07 | 0.74 | 1.57 | 0.72 |  |
| HARS2 | 5 | q31.3 | 5.38E-08 | 6.47E-06 | 0.70 | 1.28 | 0.77 |  |
| PFDN1 | 5 | q31.3 | 4.32E-07 | 3.34E-05 | 0.66 | 1.27 | 0.67 |  |
| ANKHD1 | 5 | q31.3 | 8.41E-06 | 3.23E-04 | 0.60 | 1.21 | 0.76 |  |
| ZMAT2 | 5 | q31.3 | 1.62E-04 | 3.06E-03 | 0.52 | 1.12 | 0.66 |  |
| C5orf32 | 5 | q31.3 | 1.84E-04 | 3.40E-03 | 0.52 | 1.39 | 0.73 |  |
| HARS | 5 | q31.3 | 1.92E-04 | 3.51E-03 | 0.52 | 1.52 | 0.76 |  |
| FCHSD1 | 5 | q31.3 | 4.44E-04 | 6.67E-03 | 0.49 | 1.10 | 0.96 |  |
| SLC35A4 | 5 | q31.3 | 8.00E-04 | 1.02E-02 | 0.47 | 1.38 | 0.80 |  |
| PCDH1 | 5 | q31.3 | 2.72E-03 | 2.53E-02 | 0.43 | 1.60 | 0.92 |  |
| SRA1 | 5 | q31.3 | 2.86E-03 | 2.62E-02 | 0.43 | 1.52 | 0.73 |  |
| HDAC3 | 5 | q31.3 | 3.08E-03 | 2.76E-02 | 0.42 | 1.08 | 0.88 |  |
| KIAA0141 | 5 | q31.3 | 4.17E-03 | 3.42E-02 | 0.41 | 1.06 | 0.86 |  |
| FBXO38 | 5 | q32 | 3.09E-07 | 2.66E-05 | 0.67 | 1.29 | 0.73 |  |
| TIGD6 | 5 | q32 | 6.15E-04 | 8.49E-03 | 0.48 | 1.08 | 0.91 |  |
| TCOF1 | 5 | q32 | 6.56E-04 | 8.88E-03 | 0.48 | 1.09 | 0.96 |  |
| RPS14 | 5 | q33.1 | 9.81E-06 | 3.63E-04 | 0.60 | 1.28 | 0.77 |  |
| TNIP1 | 5 | q33.1 | 1.24E-04 | 2.51E-03 | 0.53 | 1.24 | 0.86 |  |
| G3BP1 | 5 | q33.1 | 2.53E-03 | 2.40E-02 | 0.43 | 1.20 | 0.75 |  |
| NDST1 | 5 | q33.1 | 3.73E-03 | 3.16E-02 | 0.41 | 1.39 | 0.77 |  |
| SYNPO | 5 | q33.1 | 5.44E-03 | 4.14E-02 | 0.40 | 1.06 | 0.96 |  |
| ATOX1 | 5 | q33.1 | 6.61E-03 | 4.77E-02 | 0.39 | 1.35 | 0.75 |  |
| GEMIN5 | 5 | q33.2 | 4.19E-04 | 6.38E-03 | 0.49 | 1.15 | 0.80 |  |
| MRPL22 | 5 | q33.2 | 8.13E-04 | 1.03E-02 | 0.47 | 1.08 | 0.69 |  |
| LARP1 | 5 | q33.2 | 4.26E-03 | 3.47E-02 | 0.41 | 1.06 | 0.80 |  |
| HAND1 | 5 | q33.2 | 6.47E-03 | 4.70E-02 | -0.39 | 0.97 | 1.10 |  |
| TTC1 | 5 | q33.3 | 3.34E-04 | 5.36E-03 | 0.50 | 1.17 | 0.80 |  |
| PWWP2A | 5 | q33.3 | 3.57E-04 | 5.63E-03 | 0.50 | 1.11 | 0.85 |  |
| CRSP9 | 5 | q33.3 | 4.30E-04 | 6.48E-03 | 0.49 | 1.21 | 0.71 |  |
| SLU7 | 5 | q33.3 | 5.24E-04 | 7.53E-03 | 0.49 | 1.18 | 0.78 |  |
| MAT2B | 5 | q34 | 1.45E-03 | 1.59E-02 | 0.45 | 1.11 | 0.78 |  |
| ATP6V0E | 5 | q35.1 | 3.32E-04 | 5.34E-03 | 0.50 | 1.39 | 0.82 |  |
| BNIP1 | 5 | q35.1 | 7.34E-04 | 9.62E-03 | 0.48 | 1.26 | 0.94 |  |
| UBTD2 | 5 | q35.1 | 9.33E-04 | 1.14E-02 | 0.47 | 1.29 | 0.91 |  |
| ERGIC1 | 5 | q35.1 | 3.24E-03 | 2.85E-02 | 0.42 | 1.33 | 0.89 |  |
| UIMC1 | 5 | q35.2 | 1.82E-07 | 1.74E-05 | 0.68 | 1.30 | 0.73 |  |
| CLTB | 5 | q35.2 | 8.36E-06 | 3.23E-04 | 0.60 | 1.42 | 0.78 |  |
| KIAA1191 | 5 | q35.2 | 7.65E-05 | 1.76E-03 | 0.54 | 1.21 | 0.60 |  |
| THOC3 | 5 | q35.2 | 1.52E-04 | 2.93E-03 | 0.52 | 1.43 | 0.55 | 2.99 |
| ZNF346 | 5 | q35.2 | 6.81E-04 | 9.09E-03 | 0.48 | 1.30 | 0.93 |  |
| HIGD2A | 5 | q35.2 | 8.02E-04 | 1.02E-02 | 0.47 | 1.20 | 0.90 |  |
| NOP16 | 5 | q35.2 | 4.32E-03 | 3.50E-02 | 0.41 | 1.27 | 1.01 |  |
| FAM44B | 5 | q35.2 | 4.71E-03 | 3.72E-02 | 0.41 | 1.22 | 0.70 |  |
| SNCB | 5 | q35.2 | 5.00E-03 | 3.88E-02 | -0.40 | 0.95 | 1.02 |  |
| RNF44 | 5 | q35.2 | 5.87E-03 | 4.38E-02 | 0.40 | 1.33 | 0.84 |  |
| C5orf25 | 5 | q35.2 | 6.91E-03 | 4.92E-02 | 0.39 | 1.21 | 0.77 |  |
| NSD1 | 5 | q35.2-q35.3 | 7.17E-04 | 9.44E-03 | 0.48 | 1.24 | 0.92 |  |
| TRIM41 | 5 | q35.3 | 6.14E-07 | 4.34E-05 | 0.65 | 1.58 | 0.79 |  |
| TBC1D9B | 5 | q35.3 | 2.13E-06 | 1.12E-04 | 0.63 | 1.45 | 0.75 |  |
| MGAT4B | 5 | q35.3 | 2.31E-06 | 1.18E-04 | 0.63 | 1.87 | 0.83 |  |
| MAPK9 | 5 | q35.3 | 2.16E-05 | 6.53E-04 | 0.58 | 1.38 | 0.54 |  |
| PRELID1 | 5 | q35.3 | 2.53E-05 | 7.40E-04 | 0.57 | 1.32 | 0.83 |  |
| LTC4S | 5 | q35.3 | 6.34E-05 | 1.51E-03 | 0.55 | 1.14 | 0.96 |  |
| AC136632.3-2 | 5 | q35.3 | 8.54E-05 | 1.90E-03 | 0.54 | 1.45 | 0.81 |  |
| TRIM52 | 5 | q35.3 | 9.23E-05 | 2.01E-03 | 0.54 | 1.32 | 0.65 |  |
| B4GALT7 | 5 | q35.3 | 1.11E-04 | 2.30E-03 | 0.53 | 1.39 | 1.00 |  |
| AGXT2L2 | 5 | q35.3 | 2.11E-04 | 3.77E-03 | 0.52 | 1.19 | 0.90 |  |
| PRR7 | 5 | q35.3 | 3.18E-04 | 5.14E-03 | 0.50 | 1.69 | 0.78 |  |
| DDX41 | 5 | q35.3 | 3.55E-04 | 5.60E-03 | 0.50 | 1.39 | 1.10 |  |
| MAML1 | 5 | q35.3 | 3.56E-04 | 5.61E-03 | 0.50 | 1.40 | 0.93 |  |
| RGS14 | 5 | q35.3 | 5.50E-04 | 7.82E-03 | 0.49 | 1.17 | 0.89 |  |
| NHP2 | 5 | q35.3 | 1.10E-03 | 1.30E-02 | 0.46 | 1.54 | 0.84 |  |
| CNOT6 | 5 | q35.3 | 2.54E-03 | 2.40E-02 | 0.43 | 1.14 | 0.64 |  |
| RAB24 | 5 | q35.3 | 2.56E-03 | 2.42E-02 | 0.43 | 1.18 | 0.90 |  |
| RMND5B | 5 | q35.3 | 3.04E-03 | 2.73E-02 | 0.42 | 1.36 | 1.02 |  |
| HNRNPAB | 5 | q35.3 | 4.48E-03 | 3.60E-02 | 0.41 | 1.40 | 0.72 |  |
| TMED9 | 5 | q35.3 | 5.27E-03 | 4.06E-02 | 0.40 | 1.30 | 0.94 |  |
| DBN1 | 5 | q35.3 | 5.90E-03 | 4.39E-02 | 0.40 | 1.63 | 0.79 |  |
| FBXO9 | 6 | p12.1 | 5.02E-03 | 3.90E-02 | 0.40 | 1.12 | 0.93 | 1.44 |
| ZNF451 | 6 | p12.1-p11.2 | 8.57E-04 | 1.08E-02 | 0.47 | 1.12 | 0.86 |  |
| MCM3 | 6 | p12.2 | 4.59E-03 | 3.66E-02 | 0.41 | 1.25 | 0.82 | 2.19 |
| ICK | 6 | p12.2-p12.1 | 6.23E-04 | 8.56E-03 | 0.48 | 1.16 | 0.73 | 1.95 |
| MUT | 6 | p12.3 | 6.60E-03 | 4.76E-02 | 0.39 | 1.21 | 0.76 |  |
| YIPF3 | 6 | p21.1 | 1.37E-05 | 4.66E-04 | 0.59 | 1.59 | 0.74 |  |
| C6orf49 | 6 | p21.1 | 1.67E-05 | 5.42E-04 | 0.58 | 1.24 | 0.79 |  |
| PPP2R5D | 6 | p21.1 | 1.95E-05 | 6.02E-04 | 0.58 | 1.43 | 0.84 |  |
| SLC35B2 | 6 | p21.1 | 3.52E-05 | 9.63E-04 | 0.56 | 1.69 | 0.85 |  |
| MRPL14 | 6 | p21.1 | 4.71E-05 | 1.21E-03 | 0.56 | 1.23 | 0.86 |  |
| AARS2 | 6 | p21.1 | 7.62E-05 | 1.76E-03 | 0.54 | 1.36 | 0.88 |  |
| POLH | 6 | p21.1 | 8.06E-05 | 1.83E-03 | 0.54 | 1.20 | 0.91 |  |
| TBCC | 6 | p21.1 | 1.89E-04 | 3.46E-03 | 0.52 | 1.51 | 0.81 |  |
| NFYA | 6 | p21.1 | 4.09E-04 | 6.27E-03 | 0.49 | 1.27 | 0.90 |  |
| TMEM63B | 6 | p21.1 | 6.99E-04 | 9.26E-03 | 0.48 | 1.54 | 0.77 |  |
| HSP90AB1 | 6 | p21.1 | 1.62E-03 | 1.72E-02 | 0.45 | 1.38 | 0.67 |  |
| BYSL | 6 | p21.1 | 1.96E-03 | 2.00E-02 | 0.44 | 1.21 | 0.85 |  |
| GUCA1B | 6 | p21.1 | 6.52E-03 | 4.72E-02 | 0.39 | 1.20 | 0.96 |  |
| TBC1D22B | 6 | p21.2 | 7.05E-08 | 8.00E-06 | 0.69 | 1.55 | 0.88 | 3.17 |
| MTCH1 | 6 | p21.2 | 5.78E-05 | 1.41E-03 | 0.55 | 1.47 | 0.89 | 2.28 |
| TMEM217 | 6 | p21.2 | 2.25E-03 | 2.20E-02 | 0.43 | 1.03 | 0.95 | 1.12 |
| PPARD | 6 | p21.31 | 7.56E-07 | 5.10E-05 | 0.65 | 1.68 | 0.99 | 5.92 |
| SRPK1 | 6 | p21.31 | 1.59E-06 | 9.13E-05 | 0.64 | 1.47 | 0.84 | 3.26 |
| TEAD3 | 6 | p21.31 | 1.60E-06 | 9.19E-05 | 0.64 | 1.35 | 0.83 | 2.55 |
| ZNF76 | 6 | p21.31 | 4.34E-06 | 1.92E-04 | 0.61 | 1.31 | 0.84 | 2.23 |
| ANKS1A | 6 | p21.31 | 3.41E-04 | 5.45E-03 | 0.50 | 1.35 | 0.93 | 1.88 |
| KCTD20 | 6 | p21.31 | 4.46E-04 | 6.69E-03 | 0.49 | 1.30 | 0.87 | 1.91 |
| MAPK13 | 6 | p21.31 | 7.68E-04 | 9.96E-03 | 0.47 | 1.59 | 0.69 | 1.69 |
| FANCE | 6 | p21.31 | 7.77E-04 | 1.00E-02 | 0.47 | 1.38 | 0.87 | 3.04 |
| C6orf106 | 6 | p21.31 | 9.06E-04 | 1.12E-02 | 0.47 | 1.15 | 1.01 |  |
| BRPF3 | 6 | p21.31 | 1.20E-03 | 1.38E-02 | 0.46 | 1.58 | 0.86 | 1.64 |
| STK38 | 6 | p21.31 | 1.91E-03 | 1.96E-02 | 0.44 | 1.31 | 0.87 | 3.42 |
| HMGA1 | 6 | p21.31 | 2.54E-03 | 2.41E-02 | 0.43 | 1.04 | 0.96 |  |
| ITPR3 | 6 | p21.31 | 4.67E-03 | 3.70E-02 | 0.41 | 1.59 | 0.77 |  |
| SNRPC | 6 | p21.31 | 4.89E-03 | 3.82E-02 | 0.40 | 1.29 | 0.88 |  |
| NUDT3 | 6 | p21.31 | 5.48E-03 | 4.16E-02 | 0.40 | 1.31 | 0.89 |  |
| PBX2 | 6 | p21.32 | 8.49E-04 | 1.07E-02 | 0.47 | 1.42 | 0.73 |  |
| BAT2 | 6 | p21.33 | 1.84E-03 | 1.91E-02 | 0.44 | 1.43 | 0.80 |  |
| ZNF193 | 6 | p22.1 | 4.69E-06 | 2.04E-04 | 0.61 | 1.57 | 0.72 |  |
| ZNF435 | 6 | p22.1 | 8.38E-06 | 3.23E-04 | 0.60 | 1.42 | 0.75 |  |
| AL022393.3 | 6 | p22.1 | 3.35E-05 | 9.30E-04 | 0.57 | 1.18 | 0.90 |  |
| ZKSCAN4 | 6 | p22.1 | 7.22E-04 | 9.49E-03 | 0.48 | 1.25 | 0.92 |  |
| ZKSCAN3 | 6 | p22.1 | 5.04E-03 | 3.91E-02 | 0.40 | 1.15 | 0.84 |  |
| LRRC16A | 6 | p22.2 | 3.24E-03 | 2.86E-02 | 0.42 | 1.18 | 0.75 |  |
| SOX4 | 6 | p22.3 | 8.00E-04 | 1.02E-02 | 0.47 | 1.69 | 0.64 |  |
| E2F3 | 6 | p22.3 | 1.02E-03 | 1.23E-02 | 0.46 | 1.54 | 0.87 |  |
| DTNBP1 | 6 | p22.3 | 2.01E-03 | 2.03E-02 | 0.44 | 1.21 | 0.95 | 1.63 |
| TPMT | 6 | p22.3 | 3.96E-03 | 3.29E-02 | 0.41 | 1.14 | 0.86 |  |
| MRS2L | 6 | p22.3 | 6.97E-03 | 4.95E-02 | 0.39 | 1.13 | 0.89 |  |
| CCDC90A | 6 | p23 | 5.10E-04 | 7.40E-03 | 0.49 | 1.74 | 0.99 |  |
| RANBP9 | 6 | p23 | 1.32E-03 | 1.48E-02 | 0.45 | 1.57 | 0.84 |  |
| HIVEP1 | 6 | p24.1 | 6.06E-03 | 4.48E-02 | 0.39 | 1.24 | 0.86 |  |
| RP4-761I2.3 | 6 | p24.2 | 2.90E-05 | 8.29E-04 | 0.57 | 1.18 | 0.88 |  |
| SNRNP48 | 6 | p24.3 | 3.16E-03 | 2.81E-02 | 0.42 | 1.40 | 0.93 |  |
| FARS2 | 6 | p25.1 | 1.13E-03 | 1.32E-02 | 0.46 | 1.39 | 0.77 |  |
| SLC22A23 | 6 | p25.2 | 4.61E-04 | 6.85E-03 | 0.49 | 1.67 | 0.71 |  |
| NQO2 | 6 | p25.2 | 1.08E-03 | 1.27E-02 | 0.46 | 1.14 | 0.90 |  |
| WRNIP1 | 6 | p25.2 | 3.28E-03 | 2.88E-02 | 0.42 | 1.38 | 0.80 |  |
| DUSP22 | 6 | p25.3 | 2.35E-03 | 2.27E-02 | -0.43 | 0.78 | 1.27 |  |
| EXOC2 | 6 | p25.3 | 6.68E-03 | 4.80E-02 | 0.39 | 1.98 | 0.64 |  |
| RP11-74E24.2 | 6 | q12 | 1.12E-03 | 1.31E-02 | 0.46 | 1.14 | 0.87 | 3.45 |
| SMAP1 | 6 | q13 | 5.07E-03 | 3.93E-02 | 0.40 | 1.21 | 0.80 |  |
| COX7A2 | 6 | q14.1 | 1.70E-05 | 5.45E-04 | 0.58 | 1.40 | 0.81 |  |
| SENP6 | 6 | q14.1 | 2.47E-03 | 2.36E-02 | 0.43 | 1.14 | 0.79 |  |
| DOPEY1 | 6 | q14.1 | 2.51E-03 | 2.38E-02 | 0.43 | 1.13 | 0.91 |  |
| PHIP | 6 | q14.1 | 5.47E-03 | 4.15E-02 | 0.40 | 1.06 | 0.57 |  |
| SNX14 | 6 | q14.3 | 2.27E-03 | 2.21E-02 | 0.43 | 1.17 | 0.69 |  |
| RARS2 | 6 | q15 | 1.64E-04 | 3.08E-03 | 0.52 | 1.21 | 0.73 |  |
| CASP8AP2 | 6 | q15 | 4.94E-04 | 7.22E-03 | 0.49 | 1.27 | 0.80 |  |
| MAP3K7 | 6 | q15 | 4.06E-03 | 3.35E-02 | 0.41 | 1.26 | 0.75 |  |
| COQ3 | 6 | q16.2 | 1.95E-08 | 2.94E-06 | 0.71 | 1.44 | 0.75 | 3.16 |
| SFRS18 | 6 | q16.2 | 3.87E-08 | 5.01E-06 | 0.70 | 1.32 | 0.72 | 5.06 |
| C6orf168 | 6 | q16.2 | 4.81E-08 | 5.91E-06 | 0.70 | 1.34 | 0.92 | 4.29 |
| ASCC3 | 6 | q16.3 | 7.06E-03 | 5.00E-02 | 0.39 | 1.03 | 0.95 | 1.42 |
| SLC22A16 | 6 | q21 | 1.10E-11 | 7.46E-09 | 0.80 | 1.25 | 1.00 | 2.78 |
| SEC63 | 6 | q21 | 2.64E-10 | 9.53E-08 | 0.77 | 2.23 | 0.63 | 3.35 |
| C6orf185 | 6 | q21 | 4.66E-10 | 1.49E-07 | 0.76 | 1.37 | 0.94 | 1.93 |
| GPR6 | 6 | q21 | 1.58E-08 | 2.52E-06 | 0.72 | 1.12 | 0.97 | 1.89 |
| PDSS2 | 6 | q21 | 1.67E-08 | 2.62E-06 | 0.71 | 1.93 | 0.68 | 2.09 |
| CDC40 | 6 | q21 | 1.79E-08 | 2.77E-06 | 0.71 | 1.52 | 0.78 | 2.81 |
| FOXO3 | 6 | q21 | 3.78E-07 | 3.07E-05 | 0.66 | 2.26 | 0.59 | 3.55 |
| FIG4 | 6 | q21 | 9.72E-07 | 6.21E-05 | 0.65 | 1.64 | 0.73 | 3.84 |
| LACE1 | 6 | q21 | 1.56E-06 | 8.97E-05 | 0.64 | 1.37 | 0.93 | 1.55 |
| BXDC1 | 6 | q21 | 2.66E-06 | 1.31E-04 | 0.62 | 1.74 | 0.71 | 5.06 |
| CDC2L6 | 6 | q21 | 2.72E-06 | 1.33E-04 | 0.62 | 2.38 | 0.86 | 7.02 |
| ARMC2 | 6 | q21 | 5.16E-06 | 2.20E-04 | 0.61 | 1.16 | 0.95 | 1.30 |
| TUBE1 | 6 | q21 | 6.38E-06 | 2.59E-04 | 0.61 | 1.35 | 0.82 | 1.67 |
| C6orf203 | 6 | q21 | 6.50E-06 | 2.62E-04 | 0.61 | 1.40 | 0.80 | 2.08 |
| C6orf199 | 6 | q21 | 1.05E-05 | 3.83E-04 | 0.59 | 1.24 | 0.92 | 2.36 |
| OSTM1 | 6 | q21 | 1.07E-05 | 3.90E-04 | 0.59 | 1.48 | 0.88 | 2.10 |
| SNX3 | 6 | q21 | 4.16E-05 | 1.10E-03 | 0.56 | 1.12 | 0.97 | 1.23 |
| QRSL1 | 6 | q21 | 5.80E-05 | 1.41E-03 | 0.55 | 1.14 | 0.96 | 1.58 |
| HDAC2 | 6 | q21 | 8.91E-05 | 1.95E-03 | 0.54 | 1.54 | 0.62 |  |
| SMPD2 | 6 | q21 | 1.32E-04 | 2.65E-03 | 0.53 | 1.27 | 0.86 | 1.81 |
| MICAL1 | 6 | q21 | 1.96E-04 | 3.57E-03 | 0.52 | 1.37 | 0.83 | 2.60 |
| ZBTB24 | 6 | q21 | 2.94E-04 | 4.86E-03 | 0.51 | 1.26 | 0.83 | 1.52 |
| CD164 | 6 | q21 | 3.22E-04 | 5.20E-03 | 0.50 | 1.71 | 0.56 | 2.77 |
| C6orf182 | 6 | q21 | 3.63E-04 | 5.68E-03 | 0.50 | 1.14 | 0.92 | 1.24 |
| AMD1 | 6 | q21 | 4.58E-04 | 6.83E-03 | 0.49 | 2.00 | 0.83 | 4.51 |
| AIM1 | 6 | q21 | 6.01E-04 | 8.34E-03 | 0.48 | 1.49 | 0.80 | 2.36 |
| REV3L | 6 | q21 | 9.43E-04 | 1.15E-02 | 0.47 | 1.25 | 0.89 | 1.87 |
| RTN4IP1 | 6 | q21 | 1.75E-03 | 1.84E-02 | 0.44 | 1.51 | 0.78 | 2.54 |
| WISP3 | 6 | q21 | 5.33E-03 | 4.09E-02 | 0.40 | 1.07 | 0.91 | 1.00 |
| RWDD1 | 6 | q22.1 | 8.07E-07 | 5.36E-05 | 0.65 | 1.20 | 0.88 |  |
| ZUFSP | 6 | q22.1 | 1.95E-06 | 1.05E-04 | 0.63 | 1.31 | 0.80 | 1.76 |
| NUS1 | 6 | q22.1 | 8.52E-05 | 1.90E-03 | 0.54 | 1.27 | 0.85 | 3.25 |
| GOPC | 6 | q22.1 | 9.67E-04 | 1.17E-02 | 0.47 | 1.12 | 0.76 | 1.61 |
| DCBLD1 | 6 | q22.1 | 2.58E-03 | 2.42E-02 | 0.43 | 1.04 | 0.96 | 1.23 |
| HSF2 | 6 | q22.31 | 1.22E-07 | 1.26E-05 | 0.68 | 1.30 | 0.80 |  |
| FAM184A | 6 | q22.31 | 1.56E-03 | 1.68E-02 | 0.45 | 1.20 | 0.93 | 2.83 |
| RNF217 | 6 | q22.31 | 5.12E-03 | 3.96E-02 | 0.40 | 1.04 | 0.98 | 1.78 |
| C6orf173 | 6 | q22.32 | 8.45E-05 | 1.89E-03 | 0.54 | 1.34 | 0.74 | 3.69 |
| SNORD100 | 6 | q23.2 | 4.22E-04 | 6.40E-03 | 0.49 | 1.19 | 0.78 |  |
| STX7 | 6 | q23.2 | 5.36E-04 | 7.67E-03 | 0.49 | 1.20 | 0.82 |  |
| ENPP1 | 6 | q23.2 | 7.69E-04 | 9.96E-03 | 0.47 | 1.42 | 0.51 |  |
| TBPL1 | 6 | q23.2 | 1.37E-03 | 1.53E-02 | 0.45 | 1.35 | 0.76 |  |
| CRSP3 | 6 | q23.2 | 2.28E-03 | 2.22E-02 | 0.43 | 1.30 | 0.64 |  |
| NHSL1 | 6 | q23.3 | 2.17E-04 | 3.85E-03 | 0.51 | 1.20 | 0.91 |  |
| HEBP2 | 6 | q23.3 | 1.01E-03 | 1.21E-02 | 0.46 | 1.56 | 0.62 |  |
| HBS1L | 6 | q23.3 | 2.69E-03 | 2.50E-02 | 0.43 | 1.10 | 0.93 |  |
| GPR126 | 6 | q24.1 | 5.83E-07 | 4.19E-05 | 0.66 | 1.41 | 0.91 | 2.21 |
| VTA1 | 6 | q24.1 | 4.16E-05 | 1.10E-03 | 0.56 | 1.50 | 0.74 | 2.57 |
| C6orf115 | 6 | q24.1 | 1.03E-04 | 2.19E-03 | 0.54 | 1.37 | 0.65 |  |
| HECA | 6 | q24.1 | 1.79E-03 | 1.87E-02 | 0.44 | 1.12 | 0.89 |  |
| CCDC28A | 6 | q24.1 | 5.77E-03 | 4.32E-02 | 0.40 | 1.07 | 0.84 |  |
| HIVEP2 | 6 | q24.2 | 6.54E-11 | 2.96E-08 | 0.78 | 1.37 | 0.83 | 2.61 |
| LTV1 | 6 | q24.2 | 3.13E-08 | 4.30E-06 | 0.71 | 1.33 | 0.75 | 2.79 |
| PEX3 | 6 | q24.2 | 1.96E-07 | 1.83E-05 | 0.67 | 1.23 | 0.69 | 2.23 |
| AIG1 | 6 | q24.2 | 1.51E-06 | 8.76E-05 | 0.64 | 1.42 | 0.75 | 2.24 |
| PHACTR2 | 6 | q24.2 | 5.72E-04 | 8.08E-03 | 0.48 | 1.33 | 0.76 | 1.67 |
| FUCA2 | 6 | q24.2 | 1.27E-03 | 1.44E-02 | 0.46 | 1.31 | 0.84 | 2.11 |
| ADAT2 | 6 | q24.2 | 1.85E-03 | 1.91E-02 | 0.44 | 1.04 | 0.94 | 1.19 |
| SHPRH | 6 | q24.3 | 2.07E-04 | 3.73E-03 | 0.52 | 1.16 | 0.96 | 1.49 |
| SAMD5 | 6 | q24.3 | 4.97E-04 | 7.24E-03 | 0.49 | 1.04 | 0.96 | 1.62 |
| ARID1B | 6 | q25.3 | 3.07E-04 | 5.02E-03 | 0.50 | 1.52 | 0.80 |  |
| MAP3K4 | 6 | q26 | 2.68E-03 | 2.50E-02 | 0.43 | 1.32 | 0.87 |  |
| FAM120B | 6 | q27 | 3.47E-05 | 9.51E-04 | 0.57 | 1.48 | 0.63 |  |
| PDCD2 | 6 | q27 | 3.26E-04 | 5.26E-03 | 0.50 | 1.20 | 0.77 |  |
| PSMB1 | 6 | q27 | 1.30E-03 | 1.46E-02 | 0.46 | 1.26 | 0.81 |  |
| DLL1 | 6 | q27 | 3.28E-03 | 2.88E-02 | 0.42 | 1.77 | 0.75 |  |
| TBP | 6 | q27 | 6.36E-03 | 4.64E-02 | 0.39 | 1.11 | 0.81 |  |
| C6orf120 | 6 | q27 | 6.89E-03 | 4.91E-02 | 0.39 | 1.16 | 0.82 |  |
| RP11-310H4.5 | 7 | p11.2 | 1.99E-03 | 2.02E-02 | 0.44 | 1.11 | 0.83 |  |
| CCT6A | 7 | p11.2 | 3.11E-03 | 2.78E-02 | 0.42 | 1.50 | 0.61 |  |
| DDX56 | 7 | p13 | 8.35E-05 | 1.87E-03 | 0.54 | 1.49 | 0.79 |  |
| TBRG4 | 7 | p13 | 1.13E-04 | 2.35E-03 | 0.53 | 1.50 | 0.77 |  |
| OGDH | 7 | p13 | 5.13E-04 | 7.43E-03 | 0.49 | 1.21 | 0.74 |  |
| ZMIZ2 | 7 | p13 | 5.59E-03 | 4.22E-02 | 0.40 | 1.53 | 0.89 |  |
| RALA | 7 | p14.1 | 8.56E-04 | 1.07E-02 | 0.47 | 1.22 | 1.05 |  |
| GARS | 7 | p14.3 | 1.02E-04 | 2.17E-03 | 0.54 | 1.31 | 0.85 |  |
| AVL9 | 7 | p14.3 | 2.00E-03 | 2.03E-02 | 0.44 | 1.19 |  |  |
| CBX3 | 7 | p15.2 | 1.04E-04 | 2.20E-03 | 0.54 | 1.34 | 0.43 |  |
| TAX1BP1 | 7 | p15.2 | 4.01E-03 | 3.32E-02 | 0.41 | 1.19 | 0.62 |  |
| C7orf30 | 7 | p15.3 | 3.80E-04 | 5.91E-03 | 0.50 | 1.25 | 1.09 |  |
| RPA3 | 7 | p21.3 | 5.02E-03 | 3.90E-02 | 0.40 | 1.25 | 0.72 |  |
| RNF216L | 7 | p22.1 | 4.01E-03 | 3.32E-02 | 0.41 |  | 0.79 |  |
| ACTB | 7 | p22.1 | 5.76E-03 | 4.32E-02 | 0.40 |  | 0.80 |  |
| C7orf50 | 7 | p22.3 | 4.62E-05 | 1.19E-03 | 0.56 | 1.87 | 0.81 |  |
| FTSJ2 | 7 | p22.3 | 4.77E-05 | 1.21E-03 | 0.56 | 1.19 | 0.88 |  |
| PSMG3 | 7 | p22.3 | 5.32E-05 | 1.32E-03 | 0.55 | 1.85 | 0.82 |  |
| UNC84A | 7 | p22.3 | 6.42E-04 | 8.77E-03 | 0.48 | 1.05 | 0.70 |  |
| HEATR2 | 7 | p22.3 | 3.64E-03 | 3.10E-02 | 0.42 | 0.96 | 0.75 |  |
| EIF3S9 | 7 | p22.3 | 4.65E-03 | 3.69E-02 | 0.41 | 1.13 | 0.75 |  |
| ADAP1 | 7 | p22.3 | 4.93E-03 | 3.85E-02 | 0.40 | 1.67 | 0.95 |  |
| AUTS2 | 7 | q11.22 | 1.49E-03 | 1.62E-02 | 0.45 | 2.11 | 0.78 |  |
| PION | 7 | q11.23 | 7.54E-04 | 9.83E-03 | 0.47 | 1.32 | 0.76 |  |
| PCLO | 7 | q21.11 | 3.70E-03 | 3.15E-02 | 0.42 | 1.09 | 0.97 |  |
| GNAI1 | 7 | q21.11 | 5.07E-03 | 3.93E-02 | 0.40 | 1.09 | 0.96 |  |
| CROT | 7 | q21.12 | 1.13E-05 | 4.04E-04 | 0.59 | 1.57 | 0.60 | 1.42 |
| C7orf64 | 7 | q21.2 | 2.19E-03 | 2.16E-02 | 0.44 | 1.10 | 0.80 |  |
| ASNS | 7 | q21.3 | 2.66E-03 | 2.48E-02 | 0.43 | 2.06 | 0.80 | 4.08 |
| CCDC132 | 7 | q21.3 | 3.03E-03 | 2.72E-02 | 0.42 | 1.13 | 0.77 |  |
| LMTK2 | 7 | q21.3 | 3.51E-03 | 3.02E-02 | 0.42 | 1.46 | 0.97 | 1.46 |
| ARMC10 | 7 | q22.1 | 2.18E-05 | 6.58E-04 | 0.58 | 1.28 | 0.73 |  |
| AP1S1 | 7 | q22.1 | 1.07E-03 | 1.27E-02 | 0.46 |  | 0.72 |  |
| POLR2J3 | 7 | q22.1 | 4.75E-03 | 3.74E-02 | 0.40 | 1.17 | 0.81 |  |
| RASA4 | 7 | q22.1 | 5.89E-03 | 4.39E-02 | 0.40 | 1.04 | 0.93 |  |
| ORC5L | 7 | q22.1-q22.2 | 5.56E-03 | 4.20E-02 | 0.40 | 1.13 | 0.86 |  |
| RINT1 | 7 | q22.3 | 4.27E-03 | 3.47E-02 | 0.41 | 1.08 | 0.89 |  |
| ST7 | 7 | q31.2 | 5.69E-03 | 4.28E-02 | 0.40 | 1.11 | 0.71 |  |
| TNPO3 | 7 | q32.1 | 2.50E-03 | 2.38E-02 | 0.43 | 0.99 | 0.73 |  |
| ARF5 | 7 | q32.1 | 6.18E-03 | 4.54E-02 | 0.39 | 1.11 | 0.80 |  |
| MKLN1 | 7 | q32.3 | 2.99E-03 | 2.70E-02 | 0.42 | 1.43 | 0.90 | 1.75 |
| CREB3L2 | 7 | q33 | 3.88E-03 | 3.25E-02 | 0.41 | 1.23 | 0.91 |  |
| TRIM24 | 7 | q33-q34 | 6.36E-03 | 4.64E-02 | 0.39 | 1.46 | 0.88 |  |
| SLC37A3 | 7 | q34 | 1.87E-04 | 3.43E-03 | 0.52 | 1.23 | 0.88 |  |
| AC092214.7 | 7 | q34 | 2.08E-03 | 2.08E-02 | 0.44 | 1.53 | 0.81 |  |
| TMEM213 | 7 | q34 | 3.23E-03 | 2.85E-02 | 0.42 | 2.14 | 0.92 |  |
| CUL1 | 7 | q36.1 | 5.90E-06 | 2.44E-04 | 0.61 | 1.16 | 0.89 |  |
| CDK5 | 7 | q36.1 | 1.19E-04 | 2.44E-03 | 0.53 | 1.47 | 1.01 |  |
| AGAP3 | 7 | q36.1 | 2.18E-04 | 3.87E-03 | 0.51 | 1.37 | 0.91 |  |
| ATP6V0E2 | 7 | q36.1 | 2.12E-03 | 2.12E-02 | 0.44 | 1.64 | 0.76 |  |
| GALNT11 | 7 | q36.1 | 2.29E-03 | 2.22E-02 | 0.43 | 1.46 | 0.77 |  |
| ZNF398 | 7 | q36.1 | 2.53E-03 | 2.40E-02 | 0.43 | 1.30 | 0.89 |  |
| AC004877.1-2 | 7 | q36.1 | 2.64E-03 | 2.47E-02 | 0.43 | 1.16 | 0.90 |  |
| AC021097.5-1 | 7 | q36.1 | 2.73E-03 | 2.53E-02 | 0.43 | 1.22 | 0.85 |  |
| ABCB8 | 7 | q36.1 | 3.20E-03 | 2.83E-02 | 0.42 | 1.29 | 0.93 |  |
| HTR5A | 7 | q36.2 | 2.64E-03 | 2.47E-02 | -0.43 | 0.94 | 1.06 |  |
| LMBR1 | 7 | q36.3 | 3.97E-03 | 3.30E-02 | 0.41 | 1.53 | 0.82 |  |
| FNTA | 8 | p11.1 | 1.18E-04 | 2.43E-03 | 0.53 | 1.36 | 0.89 | 1.96 |
| HGSNAT | 8 | p11.1 | 5.66E-03 | 4.26E-02 | 0.40 | 1.07 | 1.00 | 1.31 |
| MYST3 | 8 | p11.21 | 1.51E-05 | 5.02E-04 | 0.59 | 1.54 | 0.80 | 1.81 |
| C8orf40 | 8 | p11.21 | 5.41E-05 | 1.34E-03 | 0.55 | 1.38 | 0.86 | 2.27 |
| SLC20A2 | 8 | p11.21 | 6.76E-05 | 1.58E-03 | 0.55 | 1.51 | 0.86 | 1.68 |
| GOLGA7 | 8 | p11.21 | 2.25E-04 | 3.96E-03 | 0.51 | 1.41 | 0.85 | 2.49 |
| POLB | 8 | p11.21 | 2.45E-04 | 4.22E-03 | 0.51 | 1.44 | 0.64 | 1.81 |
| VDAC3 | 8 | p11.21 | 1.24E-03 | 1.41E-02 | 0.46 | 1.42 | 0.82 | 1.73 |
| AP3M2 | 8 | p11.21 | 2.21E-03 | 2.17E-02 | 0.44 | 1.58 | 0.91 | 1.97 |
| IKBKB | 8 | p11.21 | 3.36E-03 | 2.93E-02 | 0.42 | 1.36 | 0.78 | 1.71 |
| THAP1 | 8 | p11.21 | 4.02E-03 | 3.32E-02 | 0.41 | 1.06 | 0.90 | 1.15 |
| RNF170 | 8 | p11.21 | 4.20E-03 | 3.43E-02 | 0.41 | 1.41 | 1.09 | 1.91 |
| HOOK3 | 8 | p11.21 | 5.03E-03 | 3.90E-02 | 0.40 | 1.28 | 0.95 | 1.20 |
| GINS4 | 8 | p11.21 | 5.37E-03 | 4.11E-02 | 0.40 | 1.23 | 0.90 | 1.51 |
| TM2D2 | 8 | p11.22 | 6.34E-06 | 2.58E-04 | 0.61 | 1.45 | 0.80 | 1.93 |
| TACC1 | 8 | p11.22 | 1.31E-03 | 1.48E-02 | 0.45 | 1.31 | 0.83 | 1.61 |
| ADAM9 | 8 | p11.22 | 4.91E-03 | 3.83E-02 | 0.40 | 1.13 | 0.92 | 1.10 |
| WHSC1L1 | 8 | p11.23 | 3.13E-13 | 3.42E-10 | 0.83 | 1.19 | 0.91 | 1.58 |
| LSM1 | 8 | p11.23 | 6.23E-12 | 4.80E-09 | 0.81 | 2.28 | 0.59 | 4.38 |
| BRF2 | 8 | p11.23 | 6.88E-12 | 5.16E-09 | 0.81 | 2.06 | 0.58 | 3.56 |
| ASH2L | 8 | p11.23 | 1.53E-11 | 9.67E-09 | 0.80 | 1.93 | 0.54 | 3.10 |
| DDHD2 | 8 | p11.23 | 3.40E-10 | 1.18E-07 | 0.77 | 1.76 | 0.54 | 2.55 |
| PROSC | 8 | p11.23 | 3.01E-09 | 6.45E-07 | 0.74 | 1.71 | 0.59 | 2.71 |
| EIF4EBP1 | 8 | p11.23 | 8.75E-09 | 1.58E-06 | 0.72 | 1.53 | 0.71 | 2.50 |
| PPAPDC1B | 8 | p11.23 | 2.38E-08 | 3.45E-06 | 0.71 | 1.11 | 0.93 | 1.23 |
| ERLIN2 | 8 | p11.23 | 1.83E-07 | 1.74E-05 | 0.68 | 1.40 | 0.82 | 1.80 |
| ZNF703 | 8 | p11.23 | 4.09E-07 | 3.21E-05 | 0.66 | 2.02 | 0.77 | 2.68 |
| RAB11FIP1 | 8 | p11.23 | 5.37E-07 | 3.99E-05 | 0.66 | 1.72 | 0.67 | 2.24 |
| BAG4 | 8 | p11.23 | 5.50E-05 | 1.35E-03 | 0.55 | 1.66 | 0.70 | 2.27 |
| LETM2 | 8 | p11.23 | 1.22E-03 | 1.39E-02 | 0.46 | 1.04 | 0.96 | 1.14 |
| FGFR1 | 8 | p11.23-p11.22 | 1.06E-08 | 1.83E-06 | 0.72 | 1.19 | 0.98 | 1.66 |
| C8orf41 | 8 | p12 | 7.23E-06 | 2.87E-04 | 0.60 | 1.50 | 0.76 |  |
| GTF2E2 | 8 | p12 | 3.42E-04 | 5.45E-03 | 0.50 | 1.30 | 0.84 |  |
| KIF13B | 8 | p12 | 5.91E-04 | 8.24E-03 | 0.48 | 1.55 | 0.54 |  |
| TMEM66 | 8 | p12 | 1.06E-03 | 1.26E-02 | 0.46 | 1.29 | 0.77 |  |
| GSR | 8 | p12 | 1.25E-03 | 1.42E-02 | 0.46 | 1.42 | 0.87 |  |
| PPP2CB | 8 | p12 | 2.35E-03 | 2.27E-02 | 0.43 | 1.26 | 0.76 |  |
| DCTN6 | 8 | p12 | 2.39E-03 | 2.30E-02 | 0.43 | 1.18 | 0.73 |  |
| WRN | 8 | p12 | 3.69E-03 | 3.13E-02 | 0.42 | 1.14 | 0.87 |  |
| snoU13 | 8 | p12 | 4.97E-03 | 3.87E-02 | 0.40 | 1.66 | 0.58 |  |
| CCDC25 | 8 | p21.1 | 2.53E-07 | 2.23E-05 | 0.67 | 1.26 | 0.64 |  |
| ELP3 | 8 | p21.1 | 2.66E-06 | 1.31E-04 | 0.62 | 1.46 | 0.72 |  |
| INTS9 | 8 | p21.1 | 2.84E-06 | 1.38E-04 | 0.62 | 1.23 | 0.78 |  |
| ZNF395 | 8 | p21.1 | 1.60E-04 | 3.04E-03 | 0.52 | 1.29 | 0.56 |  |
| EXTL3 | 8 | p21.1 | 9.88E-04 | 1.19E-02 | 0.47 | 1.35 | 0.78 |  |
| FBXO16 | 8 | p21.1 | 1.36E-03 | 1.52E-02 | 0.45 | 1.18 | 0.86 |  |
| C8orf80 | 8 | p21.1 | 4.89E-03 | 3.82E-02 | 0.40 | 1.05 | 0.95 |  |
| SCARA3 | 8 | p21.1 | 5.61E-03 | 4.23E-02 | 0.40 | 1.12 | 0.86 |  |
| HMBOX1 | 8 | p21.1-p12 | 2.39E-03 | 2.30E-02 | 0.43 | 1.17 | 0.78 |  |
| PPP2R2A | 8 | p21.2 | 5.23E-08 | 6.33E-06 | 0.70 | 1.49 | 0.64 |  |
| SLC25A37 | 8 | p21.2 | 9.38E-05 | 2.03E-03 | 0.54 | 1.41 | 0.72 |  |
| GNRH1 | 8 | p21.2 | 9.49E-05 | 2.05E-03 | 0.54 | 1.11 | 0.86 |  |
| KCTD9 | 8 | p21.2 | 2.10E-04 | 3.76E-03 | 0.52 | 1.57 | 0.79 |  |
| AC051642.5-4 | 8 | p21.2 | 4.77E-03 | 3.75E-02 | 0.40 | 1.50 | 0.79 |  |
| DOCK5 | 8 | p21.2 | 5.48E-03 | 4.16E-02 | 0.40 | 1.15 | 0.92 |  |
| EPHX2 | 8 | p21.2-p21.1 | 5.52E-03 | 4.18E-02 | 0.40 | 1.10 | 0.56 |  |
| R3HCC1 | 8 | p21.3 | 8.30E-07 | 5.47E-05 | 0.65 | 1.27 | 0.74 |  |
| CHMP7 | 8 | p21.3 | 1.99E-06 | 1.07E-04 | 0.63 | 1.26 | 0.72 |  |
| KIAA1967 | 8 | p21.3 | 3.66E-06 | 1.70E-04 | 0.62 | 1.25 | 0.76 |  |
| PDLIM2 | 8 | p21.3 | 5.13E-06 | 2.20E-04 | 0.61 | 1.15 | 0.89 |  |
| FAM160B2 | 8 | p21.3 | 8.66E-06 | 3.31E-04 | 0.60 | 1.48 | 0.74 |  |
| TNFRSF10B | 8 | p21.3 | 1.33E-03 | 1.48E-02 | 0.45 | 1.29 | 0.79 |  |
| BIN3 | 8 | p21.3 | 2.13E-03 | 2.12E-02 | 0.44 | 1.22 | 0.84 |  |
| REEP4 | 8 | p21.3 | 2.58E-03 | 2.42E-02 | 0.43 | 1.15 | 0.93 |  |
| ATP6V1B2 | 8 | p21.3 | 3.38E-03 | 2.94E-02 | 0.42 | 1.29 | 0.86 |  |
| INTS10 | 8 | p21.3 | 6.60E-03 | 4.76E-02 | 0.39 | 1.32 | 0.80 |  |
| SORBS3 | 8 | p21.3 | 6.69E-03 | 4.80E-02 | 0.39 | 1.11 | 0.74 |  |
| CNOT7 | 8 | p22 | 3.53E-04 | 5.57E-03 | 0.50 | 1.45 | 0.74 |  |
| NEIL2 | 8 | p23.1 | 1.02E-05 | 3.75E-04 | 0.60 | 1.43 | 0.73 |  |
| SOX7 | 8 | p23.1 | 1.48E-03 | 1.61E-02 | 0.45 | 1.08 | 0.91 |  |
| MSRA | 8 | p23.1 | 1.84E-03 | 1.91E-02 | 0.44 | 0.92 | 0.88 |  |
| AGPAT5 | 8 | p23.1 | 3.04E-03 | 2.73E-02 | 0.42 | 2.15 | 0.68 |  |
| MFHAS1 | 8 | p23.1 | 6.51E-03 | 4.72E-02 | 0.39 | 1.39 | 0.83 |  |
| ERICH1 | 8 | p23.3 | 7.45E-04 | 9.73E-03 | 0.47 | 1.22 | 0.84 |  |
| ARHGEF10 | 8 | p23.3 | 1.18E-03 | 1.37E-02 | 0.46 | 1.27 | 0.82 |  |
| MYOM2 | 8 | p23.3 | 6.51E-03 | 4.72E-02 | 0.39 | 1.58 | 0.86 |  |
| UBE2V2 | 8 | q11.21 | 5.31E-07 | 3.96E-05 | 0.66 | 1.51 | 0.64 | 2.26 |
| PRKDC | 8 | q11.21 | 7.10E-07 | 4.88E-05 | 0.65 | 1.49 | 0.56 | 1.30 |
| KIAA0146 | 8 | q11.21 | 8.24E-05 | 1.85E-03 | 0.54 | 1.44 | 0.54 | 1.82 |
| MCM4 | 8 | q11.21 | 9.86E-05 | 2.11E-03 | 0.54 | 1.35 | 0.60 | 1.94 |
| ATP6V1H | 8 | q11.23 | 3.80E-08 | 4.97E-06 | 0.70 | 1.50 | 0.65 | 2.38 |
| TCEA1 | 8 | q11.23 | 2.07E-06 | 1.09E-04 | 0.63 | 1.15 | 0.92 | 1.55 |
| MRPL15 | 8 | q11.23 | 5.63E-06 | 2.36E-04 | 0.61 | 1.45 | 0.60 | 1.58 |
| RB1CC1 | 8 | q11.23 | 2.56E-05 | 7.50E-04 | 0.57 | 1.44 | 0.70 | 1.65 |
| LYPLA1 | 8 | q11.23 | 7.13E-04 | 9.40E-03 | 0.48 | 1.25 | 0.71 | 2.09 |
| PCMTD1 | 8 | q11.23 | 6.93E-03 | 4.93E-02 | 0.39 | 1.23 | 0.52 | 1.52 |
| IMPAD1 | 8 | q12.1 | 1.01E-04 | 2.14E-03 | 0.54 | 1.31 | 0.86 | 1.59 |
| SDCBP | 8 | q12.1 | 6.53E-04 | 8.87E-03 | 0.48 | 1.41 | 0.72 | 1.51 |
| RAB2 | 8 | q12.1 | 1.29E-03 | 1.45E-02 | 0.46 | 1.32 | 0.83 | 1.74 |
| RPS20 | 8 | q12.1 | 2.01E-03 | 2.03E-02 | 0.44 | 1.41 | 0.69 | 1.27 |
| AC104350.6 | 8 | q12.1 | 2.91E-03 | 2.65E-02 | 0.42 | 1.05 | 0.95 | 1.04 |
| NSMAF | 8 | q12.1 | 3.31E-03 | 2.90E-02 | 0.42 | 1.34 | 0.80 | 1.16 |
| YTHDF3 | 8 | q12.3 | 2.31E-04 | 4.04E-03 | 0.51 | 1.26 | 0.57 | 1.72 |
| ASPH | 8 | q12.3 | 1.20E-03 | 1.38E-02 | 0.46 | 1.06 | 0.91 | 1.26 |
| COPS5 | 8 | q13.1 | 1.33E-12 | 1.22E-09 | 0.82 | 1.63 | 0.58 | 2.12 |
| RRS1 | 8 | q13.1 | 1.22E-07 | 1.26E-05 | 0.68 | 1.61 | 0.47 | 2.39 |
| MTFR1 | 8 | q13.1 | 6.27E-07 | 4.40E-05 | 0.65 | 1.68 | 0.66 | 1.67 |
| VCPIP1 | 8 | q13.1 | 2.34E-05 | 6.97E-04 | 0.58 | 1.39 | 0.64 | 1.38 |
| ARMC1 | 8 | q13.1 | 3.08E-05 | 8.71E-04 | 0.57 | 1.42 | 0.71 | 1.51 |
| C8orf44 | 8 | q13.1 | 8.16E-05 | 1.84E-03 | 0.54 | 1.21 | 0.76 | 1.26 |
| SGK3 | 8 | q13.1 | 4.31E-03 | 3.49E-02 | 0.41 | 1.05 | 0.95 | 1.05 |
| CSPP1 | 8 | q13.1-q13.2 | 7.88E-04 | 1.01E-02 | 0.47 | 1.13 | 0.89 | 1.23 |
| ARFGEF1 | 8 | q13.2 | 9.47E-09 | 1.66E-06 | 0.72 | 1.65 | 0.57 | 2.02 |
| LACTB2 | 8 | q13.3 | 4.66E-07 | 3.55E-05 | 0.66 | 1.55 | 0.61 | 2.74 |
| TRAM1 | 8 | q13.3 | 3.51E-03 | 3.02E-02 | 0.42 | 1.24 | 0.72 | 1.50 |
| TCEB1 | 8 | q21.11 | 4.29E-07 | 3.34E-05 | 0.66 | 1.57 | 0.54 | 1.94 |
| PXMP3 | 8 | q21.11 | 5.40E-07 | 4.01E-05 | 0.66 | 1.36 | 0.50 | 2.07 |
| STAU2 | 8 | q21.11 | 1.85E-06 | 1.02E-04 | 0.63 | 1.37 | 0.61 | 1.86 |
| UBE2W | 8 | q21.11 | 1.97E-06 | 1.06E-04 | 0.63 | 1.26 | 0.75 | 1.89 |
| TERF1 | 8 | q21.11 | 2.49E-06 | 1.25E-04 | 0.63 | 1.49 | 0.59 | 1.61 |
| TMEM70 | 8 | q21.11 | 6.89E-04 | 9.17E-03 | 0.48 | 1.36 | 0.57 | 1.40 |
| ZFAND1 | 8 | q21.13 | 2.16E-07 | 1.97E-05 | 0.67 | 1.35 | 0.57 | 1.94 |
| MRPS28 | 8 | q21.13 | 8.95E-07 | 5.82E-05 | 0.65 | 1.35 | 0.65 | 1.90 |
| IMPA1 | 8 | q21.13 | 8.08E-04 | 1.03E-02 | 0.47 | 1.18 | 0.67 | 1.79 |
| SNX16 | 8 | q21.13 | 2.57E-03 | 2.42E-02 | 0.43 | 1.23 | 0.64 | 1.18 |
| TPD52 | 8 | q21.13 | 3.36E-03 | 2.93E-02 | 0.42 | 1.03 | 0.95 | 1.05 |
| E2F5 | 8 | q21.2 | 5.48E-07 | 4.05E-05 | 0.66 | 1.52 | 0.57 | 2.04 |
| C8orf59 | 8 | q21.2 | 3.19E-04 | 5.17E-03 | 0.50 | 1.35 | 0.59 | 1.52 |
| OTUD6B | 8 | q21.3 | 1.19E-10 | 4.98E-08 | 0.78 | 1.23 | 0.72 | 1.62 |
| CPNE3 | 8 | q21.3 | 8.10E-07 | 5.37E-05 | 0.65 | 1.39 | 0.43 | 1.55 |
| FAM82B | 8 | q21.3 | 1.03E-06 | 6.42E-05 | 0.64 | 1.39 | 0.69 | 1.71 |
| OSGIN2 | 8 | q21.3 | 1.75E-06 | 9.82E-05 | 0.63 | 1.26 | 0.62 | 1.50 |
| RIPK2 | 8 | q21.3 | 2.31E-06 | 1.18E-04 | 0.63 | 1.50 | 0.77 | 1.79 |
| DECR1 | 8 | q21.3 | 5.13E-05 | 1.28E-03 | 0.56 | 1.28 | 0.72 | 1.73 |
| WWP1 | 8 | q21.3 | 1.33E-04 | 2.65E-03 | 0.53 | 1.40 | 0.48 | 1.52 |
| TMEM55A | 8 | q21.3 | 4.94E-04 | 7.22E-03 | 0.49 | 1.18 | 0.75 | 1.83 |
| NBN | 8 | q21.3 | 2.02E-03 | 2.04E-02 | 0.44 | 1.28 | 0.38 | 1.57 |
| PTDSS1 | 8 | q22.1 | 3.25E-11 | 1.60E-08 | 0.79 | 1.39 | 0.66 | 1.92 |
| MTERFD1 | 8 | q22.1 | 7.84E-10 | 2.21E-07 | 0.76 | 1.42 | 0.48 | 1.83 |
| AP003117.2 | 8 | q22.1 | 5.11E-07 | 3.86E-05 | 0.66 | 1.15 | 0.67 | 1.77 |
| UQCRB | 8 | q22.1 | 4.51E-06 | 1.98E-04 | 0.61 | 1.49 | 0.40 | 1.51 |
| C8orf38 | 8 | q22.1 | 5.18E-06 | 2.21E-04 | 0.61 | 1.65 | 0.60 | 1.93 |
| CCNE2 | 8 | q22.1 | 1.83E-05 | 5.73E-04 | 0.58 | 1.37 | 0.77 | 2.11 |
| LAPTM4B | 8 | q22.1 | 1.40E-04 | 2.75E-03 | 0.53 | 1.79 | 0.53 | 2.75 |
| RAD54B | 8 | q22.1 | 2.19E-04 | 3.87E-03 | 0.51 | 1.23 | 0.79 | 1.53 |
| TP53INP1 | 8 | q22.1 | 2.91E-04 | 4.82E-03 | 0.51 | 1.36 | 0.69 | 2.26 |
| KIAA1429 | 8 | q22.1 | 3.27E-04 | 5.27E-03 | 0.50 | 1.21 | 0.72 | 1.41 |
| TSPYL5 | 8 | q22.1 | 3.48E-04 | 5.52E-03 | 0.50 | 1.60 | 0.57 | 2.67 |
| TMEM67 | 8 | q22.1 | 4.33E-04 | 6.52E-03 | 0.49 | 1.06 | 1.01 | 1.40 |
| DPY19L4 | 8 | q22.1 | 7.26E-04 | 9.53E-03 | 0.48 | 1.23 | 0.57 | 1.77 |
| PLEKHF2 | 8 | q22.1 | 1.20E-03 | 1.38E-02 | 0.46 | 1.46 | 0.46 | 1.68 |
| MTDH | 8 | q22.1 | 1.37E-03 | 1.52E-02 | 0.45 | 1.36 | 0.58 | 1.73 |
| GEM | 8 | q22.1 | 3.94E-03 | 3.28E-02 | 0.41 | 1.08 | 0.80 | 1.40 |
| C8orf37 | 8 | q22.1 | 6.26E-03 | 4.59E-02 | 0.39 | 1.07 | 0.95 | 1.02 |
| HRSP12 | 8 | q22.2 | 1.81E-09 | 4.29E-07 | 0.75 | 1.43 | 0.55 | 1.94 |
| ANKRD46 | 8 | q22.2 | 2.29E-07 | 2.06E-05 | 0.67 | 1.46 | 0.45 | 2.23 |
| RPL30 | 8 | q22.2 | 9.72E-07 | 6.21E-05 | 0.65 | 1.36 | 0.57 | 1.76 |
| STK3 | 8 | q22.2 | 2.51E-06 | 1.25E-04 | 0.63 | 1.47 | 0.50 | 1.73 |
| POLR2K | 8 | q22.2 | 1.32E-04 | 2.65E-03 | 0.53 | 1.23 | 0.80 | 1.42 |
| SPAG1 | 8 | q22.2 | 1.37E-04 | 2.72E-03 | 0.53 | 1.06 | 0.96 | 1.31 |
| AZIN1 | 8 | q22.3 | 7.38E-09 | 1.40E-06 | 0.73 | 1.72 | 0.41 | 2.30 |
| ZNF706 | 8 | q22.3 | 1.46E-08 | 2.38E-06 | 0.72 | 1.60 | 0.52 | 2.41 |
| UBR5 | 8 | q22.3 | 3.21E-08 | 4.35E-06 | 0.70 | 1.43 | 0.48 | 2.00 |
| RRM2B | 8 | q22.3 | 1.55E-06 | 8.96E-05 | 0.64 | 1.26 | 0.53 | 1.74 |
| PABPCP5 | 8 | q22.3 | 5.44E-06 | 2.29E-04 | 0.61 | 1.38 | 0.64 | 1.61 |
| ATP6V1C1 | 8 | q22.3 | 5.32E-05 | 1.32E-03 | 0.55 | 1.44 | 0.45 | 1.44 |
| YWHAZ | 8 | q22.3 | 1.64E-04 | 3.09E-03 | 0.52 | 1.06 | 1.02 | 1.27 |
| WDSOF1 | 8 | q22.3 | 2.18E-04 | 3.86E-03 | 0.51 | 1.40 | 0.75 | 1.50 |
| FZD6 | 8 | q22.3 | 6.36E-04 | 8.71E-03 | 0.48 | 1.34 | 0.80 | 1.98 |
| GRHL2 | 8 | q22.3 | 6.51E-04 | 8.86E-03 | 0.48 | 1.33 | 0.63 | 1.34 |
| KLF10 | 8 | q22.3 | 2.56E-03 | 2.42E-02 | 0.43 | 1.16 | 0.83 | 1.28 |
| ENY2 | 8 | q23.1 | 8.88E-16 | 3.61E-12 | 0.88 | 1.65 | 0.55 | 2.24 |
| NUDCD1 | 8 | q23.1 | 3.46E-06 | 1.62E-04 | 0.62 | 1.17 | 0.81 | 1.42 |
| TTC35 | 8 | q23.1 | 4.20E-05 | 1.10E-03 | 0.56 | 1.25 | 0.71 | 1.31 |
| OXR1 | 8 | q23.1 | 2.50E-03 | 2.38E-02 | 0.43 | 1.17 | 0.50 | 1.74 |
| EBAG9 | 8 | q23.2 | 4.07E-11 | 1.93E-08 | 0.79 | 1.53 | 0.37 | 1.89 |
| MED30 | 8 | q24.11 | 3.23E-08 | 4.35E-06 | 0.70 | 1.54 | 0.49 | 2.32 |
| UTP23 | 8 | q24.11 | 1.27E-05 | 4.42E-04 | 0.59 | 1.50 | 0.76 | 1.56 |
| RAD21 | 8 | q24.11 | 2.30E-03 | 2.24E-02 | 0.43 | 1.80 | 0.54 | 1.39 |
| C8orf85 | 8 | q24.11 | 2.50E-03 | 2.38E-02 | 0.43 | 1.47 | 0.55 | 2.16 |
| TAF2 | 8 | q24.12 | 7.15E-07 | 4.90E-05 | 0.65 | 1.52 | 0.69 | 1.71 |
| MRPL13 | 8 | q24.12 | 1.02E-06 | 6.41E-05 | 0.64 | 1.69 | 0.45 | 1.84 |
| MAL2 | 8 | q24.12 | 9.32E-06 | 3.49E-04 | 0.60 | 1.64 | 0.42 | 2.24 |
| DSCC1 | 8 | q24.12 | 2.04E-04 | 3.68E-03 | 0.52 | 1.44 | 0.74 | 1.35 |
| MTBP | 8 | q24.12 | 1.07E-03 | 1.27E-02 | 0.46 | 1.07 | 0.91 | 1.16 |
| DEPDC6 | 8 | q24.12 | 4.42E-03 | 3.56E-02 | 0.41 | 1.29 | 0.72 | 1.44 |
| DERL1 | 8 | q24.13 | 5.07E-10 | 1.59E-07 | 0.76 | 1.48 | 0.59 | 1.67 |
| C8orf76 | 8 | q24.13 | 8.15E-10 | 2.25E-07 | 0.76 | 1.62 | 0.56 | 1.88 |
| NSMCE2 | 8 | q24.13 | 9.72E-08 | 1.05E-05 | 0.69 | 1.60 | 0.49 | 1.54 |
| ATAD2 | 8 | q24.13 | 2.42E-07 | 2.16E-05 | 0.67 | 1.56 | 0.60 | 1.91 |
| TATDN1 | 8 | q24.13 | 3.20E-07 | 2.70E-05 | 0.67 | 1.52 | 0.51 | 1.64 |
| KIAA0196 | 8 | q24.13 | 3.99E-06 | 1.81E-04 | 0.62 | 1.66 | 0.45 | 1.89 |
| WDYHV1 | 8 | q24.13 | 6.51E-06 | 2.62E-04 | 0.61 | 1.40 | 0.67 | 1.49 |
| TRMT12 | 8 | q24.13 | 3.39E-05 | 9.37E-04 | 0.57 | 1.41 | 0.44 | 1.34 |
| WDR67 | 8 | q24.13 | 1.17E-04 | 2.41E-03 | 0.53 | 1.25 | 0.84 | 1.36 |
| SQLE | 8 | q24.13 | 1.42E-04 | 2.77E-03 | 0.53 | 1.36 | 0.85 | 1.69 |
| ZHX2 | 8 | q24.13 | 6.03E-04 | 8.36E-03 | 0.48 | 1.17 | 0.70 | 1.44 |
| TRIB1 | 8 | q24.13 | 1.30E-03 | 1.47E-02 | 0.46 | 1.40 | 0.54 | 1.30 |
| FAM91A1 | 8 | q24.13 | 1.56E-03 | 1.67E-02 | 0.45 | 1.14 | 0.85 | 1.16 |
| FBXO32 | 8 | q24.13 | 1.58E-03 | 1.69E-02 | 0.45 | 1.31 | 0.88 | 1.88 |
| NDUFB9 | 8 | q24.13 | 3.80E-03 | 3.20E-02 | 0.41 | 1.49 | 0.66 | 1.18 |
| ZNF572 | 8 | q24.13 | 6.11E-03 | 4.50E-02 | 0.39 | 1.06 | 0.98 | 1.10 |
| AC103819.3-2 | 8 | q24.21 | 4.53E-09 | 9.21E-07 | 0.73 | 1.21 | 0.85 | 1.24 |
| FAM84B | 8 | q24.21 | 8.50E-09 | 1.54E-06 | 0.72 | 1.84 | 0.50 | 2.51 |
| MYC | 8 | q24.21 | 4.68E-04 | 6.93E-03 | 0.49 | 1.69 | 0.46 | 1.84 |
| AF186191.6-2 | 8 | q24.22 | 1.67E-07 | 1.62E-05 | 0.68 | 1.64 | 0.47 | 1.87 |
| PHF20L1 | 8 | q24.22 | 3.35E-05 | 9.31E-04 | 0.57 | 1.44 | 0.62 | 1.61 |
| EFR3A | 8 | q24.22 | 1.03E-04 | 2.17E-03 | 0.54 | 1.39 | 0.64 | 1.60 |
| CYC1 | 8 | q24.3 | 4.57E-08 | 5.71E-06 | 0.70 | 1.66 | 0.61 |  |
| C8orf33 | 8 | q24.3 | 1.02E-07 | 1.09E-05 | 0.69 | 1.67 | 0.76 |  |
| CHRAC1 | 8 | q24.3 | 1.17E-07 | 1.22E-05 | 0.68 | 1.27 | 0.72 | 1.29 |
| BOP1 | 8 | q24.3 | 2.30E-07 | 2.06E-05 | 0.67 | 1.75 | 0.65 |  |
| ZNF7 | 8 | q24.3 | 7.08E-07 | 4.88E-05 | 0.65 | 1.44 | 0.69 |  |
| HSF1 | 8 | q24.3 | 2.02E-06 | 1.08E-04 | 0.63 | 1.53 | 0.77 |  |
| ZNF696 | 8 | q24.3 | 2.21E-06 | 1.14E-04 | 0.63 | 1.51 | 0.76 | 1.44 |
| GPR172A | 8 | q24.3 | 2.30E-06 | 1.18E-04 | 0.63 | 1.71 | 0.75 |  |
| RPL8 | 8 | q24.3 | 3.03E-06 | 1.44E-04 | 0.62 | 1.98 | 0.71 |  |
| TIGD5 | 8 | q24.3 | 4.35E-06 | 1.92E-04 | 0.61 | 1.57 | 0.71 |  |
| PYCRL | 8 | q24.3 | 1.35E-05 | 4.60E-04 | 0.59 | 1.29 | 0.79 |  |
| LRRC24 | 8 | q24.3 | 1.58E-05 | 5.18E-04 | 0.58 | 1.74 | 0.56 |  |
| PUF60 | 8 | q24.3 | 1.75E-05 | 5.53E-04 | 0.58 | 1.40 | 0.88 |  |
| C8orf30A | 8 | q24.3 | 2.45E-05 | 7.24E-04 | 0.57 | 1.58 | 0.65 |  |
| SLC39A4 | 8 | q24.3 | 3.17E-05 | 8.91E-04 | 0.57 | 2.01 | 0.79 |  |
| ZNF250 | 8 | q24.3 | 3.22E-05 | 9.00E-04 | 0.57 | 1.23 | 0.77 |  |
| AC145291.3 | 8 | q24.3 | 3.41E-05 | 9.39E-04 | 0.57 | 1.11 | 0.92 |  |
| FAM83H | 8 | q24.3 | 5.09E-05 | 1.28E-03 | 0.56 | 1.86 | 0.68 |  |
| TRAPPC9 | 8 | q24.3 | 5.25E-05 | 1.31E-03 | 0.55 | 1.54 | 0.74 | 1.58 |
| VPS28 | 8 | q24.3 | 6.19E-05 | 1.48E-03 | 0.55 | 1.44 | 0.60 |  |
| GSDMD | 8 | q24.3 | 7.92E-05 | 1.80E-03 | 0.54 | 1.57 | 0.71 |  |
| C8orf55 | 8 | q24.3 | 8.46E-05 | 1.89E-03 | 0.54 | 1.59 | 0.78 | 1.41 |
| SCRIB | 8 | q24.3 | 8.63E-05 | 1.91E-03 | 0.54 | 1.11 | 0.94 |  |
| EEF1D | 8 | q24.3 | 1.19E-04 | 2.44E-03 | 0.53 | 1.22 | 0.85 |  |
| ZNF34 | 8 | q24.3 | 1.22E-04 | 2.49E-03 | 0.53 | 1.27 | 0.78 |  |
| ZFP41 | 8 | q24.3 | 1.50E-04 | 2.89E-03 | 0.53 | 1.35 | 0.73 | 1.10 |
| LRRC14 | 8 | q24.3 | 1.62E-04 | 3.06E-03 | 0.52 | 1.41 | 0.70 |  |
| ZC3H3 | 8 | q24.3 | 1.63E-04 | 3.07E-03 | 0.52 | 1.36 | 0.77 |  |
| EXOSC4 | 8 | q24.3 | 1.71E-04 | 3.20E-03 | 0.52 | 1.60 | 0.91 |  |
| GRINA | 8 | q24.3 | 1.92E-04 | 3.52E-03 | 0.52 | 1.62 | 0.76 |  |
| C8orf51 | 8 | q24.3 | 2.11E-04 | 3.76E-03 | 0.52 | 1.55 | 0.79 |  |
| TOP1MT | 8 | q24.3 | 2.23E-04 | 3.94E-03 | 0.51 | 1.37 | 0.78 |  |
| CPSF1 | 8 | q24.3 | 2.54E-04 | 4.33E-03 | 0.51 | 1.49 | 0.76 |  |
| MFSD3 | 8 | q24.3 | 4.05E-04 | 6.21E-03 | 0.49 | 1.75 | 0.68 |  |
| MAF1 | 8 | q24.3 | 4.33E-04 | 6.53E-03 | 0.49 | 1.48 | 0.72 |  |
| PPP1R16A | 8 | q24.3 | 5.88E-04 | 8.23E-03 | 0.48 | 1.65 | 0.75 |  |
| SHARPIN | 8 | q24.3 | 6.48E-04 | 8.83E-03 | 0.48 | 1.57 | 0.70 |  |
| NRBP2 | 8 | q24.3 | 6.52E-04 | 8.87E-03 | 0.48 | 1.61 | 0.63 |  |
| ZNF517 | 8 | q24.3 | 6.54E-04 | 8.87E-03 | 0.48 | 1.23 | 0.85 |  |
| PTK2 | 8 | q24.3 | 8.54E-04 | 1.07E-02 | 0.47 | 1.34 | 0.50 | 1.29 |
| GPAA1 | 8 | q24.3 | 1.22E-03 | 1.40E-02 | 0.46 | 1.58 | 0.82 |  |
| FBXL6 | 8 | q24.3 | 1.25E-03 | 1.42E-02 | 0.46 | 1.45 | 0.90 |  |
| HEATR7A | 8 | q24.3 | 1.43E-03 | 1.57E-02 | 0.45 | 1.29 | 0.90 |  |
| RECQL4 | 8 | q24.3 | 1.48E-03 | 1.61E-02 | 0.45 | 1.59 | 0.74 |  |
| NAPRT1 | 8 | q24.3 | 1.84E-03 | 1.91E-02 | 0.44 | 1.38 | 0.81 |  |
| AC084125.9 | 8 | q24.3 | 2.05E-03 | 2.06E-02 | 0.44 | 1.71 | 0.71 |  |
| OPLAH | 8 | q24.3 | 2.36E-03 | 2.28E-02 | 0.43 | 1.40 | 0.85 |  |
| C8orf77 | 8 | q24.3 | 2.44E-03 | 2.34E-02 | 0.43 | 1.09 | 0.96 |  |
| DGAT1 | 8 | q24.3 | 2.54E-03 | 2.40E-02 | 0.43 | 1.43 | 0.78 |  |
| CYHR1 | 8 | q24.3 | 2.77E-03 | 2.56E-02 | 0.43 | 1.40 | 0.80 |  |
| ADCK5 | 8 | q24.3 | 3.98E-03 | 3.31E-02 | 0.41 | 1.29 | 0.86 |  |
| ZNF707 | 8 | q24.3 | 4.27E-03 | 3.47E-02 | 0.41 | 1.35 | 0.83 |  |
| JRK | 8 | q24.3 | 4.51E-03 | 3.61E-02 | 0.41 | 1.14 | 0.95 | 1.00 |
| ZNF16 | 8 | q24.3 | 4.54E-03 | 3.63E-02 | 0.41 | 1.16 | 0.86 |  |
| KIFC2 | 8 | q24.3 | 5.21E-03 | 4.02E-02 | 0.40 | 1.90 | 0.57 |  |
| SHB | 9 | p13.1 | 1.38E-04 | 2.72E-03 | 0.53 | 2.17 | 0.82 |  |
| GRHPR | 9 | p13.2 | 1.40E-06 | 8.19E-05 | 0.64 | 1.49 | 0.73 |  |
| POLR1E | 9 | p13.2 | 8.48E-05 | 1.89E-03 | 0.54 | 1.64 | 0.80 |  |
| TOMM5 | 9 | p13.2 | 2.58E-04 | 4.39E-03 | 0.51 | 3.79 | 0.73 |  |
| ZBTB5 | 9 | p13.2 | 4.68E-03 | 3.70E-02 | 0.41 | 1.42 | 0.84 |  |
| UBAP1 | 9 | p13.3 | 5.39E-06 | 2.28E-04 | 0.61 | 1.58 | 0.73 |  |
| CLTA | 9 | p13.3 | 3.09E-05 | 8.73E-04 | 0.57 | 1.56 | 0.81 |  |
| STOML2 | 9 | p13.3 | 8.94E-05 | 1.96E-03 | 0.54 | 2.22 | 0.79 |  |
| HINT2 | 9 | p13.3 | 2.85E-04 | 4.74E-03 | 0.51 | 1.46 | 0.73 |  |
| WDR40A | 9 | p13.3 | 4.79E-04 | 7.05E-03 | 0.49 | 2.39 | 0.81 |  |
| NOL6 | 9 | p13.3 | 5.27E-04 | 7.56E-03 | 0.49 | 1.15 | 0.83 |  |
| C9orf23 | 9 | p13.3 | 9.37E-04 | 1.14E-02 | 0.47 | 1.94 | 0.75 |  |
| NFX1 | 9 | p13.3 | 4.94E-03 | 3.85E-02 | 0.40 | 1.80 | 0.89 |  |
| APTX | 9 | p21.1 | 1.24E-04 | 2.51E-03 | 0.53 | 1.19 | 0.82 |  |
| TOPORS | 9 | p21.1 | 2.71E-03 | 2.52E-02 | 0.43 | 1.20 | 0.86 |  |
| MTAP | 9 | p21.3 | 1.31E-03 | 1.47E-02 | 0.46 | 1.15 | 0.77 |  |
| CDC37L1 | 9 | p24.1 | 7.33E-04 | 9.62E-03 | 0.48 | 1.12 | 0.68 |  |
| KIAA2026 | 9 | p24.1 | 2.39E-03 | 2.30E-02 | 0.43 | 1.13 | 0.90 |  |
| RFX3 | 9 | p24.2 | 1.40E-03 | 1.54E-02 | 0.45 | 1.03 | 0.94 |  |
| KANK1 | 9 | p24.3 | 4.49E-03 | 3.60E-02 | 0.41 | 1.53 | 0.73 |  |
| RP11-111F5.3 | 9 | q12 | 4.29E-03 | 3.49E-02 | 0.41 | 1.04 | 0.94 |  |
| TJP2 | 9 | q21.11 | 1.06E-04 | 2.23E-03 | 0.54 | 1.27 | 0.65 |  |
| SMC5 | 9 | q21.12 | 9.62E-05 | 2.08E-03 | -0.54 | 0.92 | 1.06 |  |
| C9orf61 | 9 | q21.12 | 3.59E-03 | 3.07E-02 | 0.42 | 1.20 | 0.69 |  |
| ZFAND5 | 9 | q21.13 | 5.21E-04 | 7.51E-03 | 0.49 | 1.27 | 0.75 |  |
| GNAQ | 9 | q21.2 | 5.42E-03 | 4.13E-02 | 0.40 | 0.98 | 0.74 |  |
| UBQLN1 | 9 | q21.32 | 7.63E-05 | 1.76E-03 | 0.54 | 1.07 | 0.74 |  |
| C9orf103 | 9 | q21.32 | 1.34E-03 | 1.50E-02 | 0.45 | 0.95 | 0.69 |  |
| AL160279.21-1 | 9 | q21.33-q22.1 | 2.59E-04 | 4.40E-03 | -0.51 | 1.02 | 1.10 |  |
| SECISBP2 | 9 | q22.2 | 2.03E-03 | 2.05E-02 | 0.44 | 0.89 | 0.68 |  |
| PHF2 | 9 | q22.31 | 3.58E-04 | 5.63E-03 | 0.50 | 1.02 | 0.77 |  |
| NOL8 | 9 | q22.31 | 6.18E-04 | 8.51E-03 | 0.48 | 1.02 | 0.74 |  |
| IARS | 9 | q22.31 | 1.56E-03 | 1.68E-02 | 0.45 | 0.74 | 0.61 |  |
| HIATL1 | 9 | q22.32 | 2.46E-05 | 7.25E-04 | 0.57 | 1.19 | 0.75 |  |
| SLC35D2 | 9 | q22.32 | 5.77E-03 | 4.32E-02 | 0.40 | 1.15 | 0.78 |  |
| C9orf156 | 9 | q22.33 | 1.40E-07 | 1.39E-05 | 0.68 | 1.31 | 0.77 |  |
| CORO2A | 9 | q22.33 | 7.83E-05 | 1.79E-03 | 0.54 | 2.27 | 0.66 |  |
| TDRD7 | 9 | q22.33 | 1.94E-04 | 3.53E-03 | 0.52 | 0.91 | 0.79 |  |
| ANP32B | 9 | q22.33 | 3.46E-04 | 5.49E-03 | 0.50 | 1.26 | 0.77 |  |
| TRIM14 | 9 | q22.33 | 7.97E-04 | 1.02E-02 | 0.47 | 1.10 | 0.95 |  |
| ALG2 | 9 | q22.33 | 1.46E-03 | 1.59E-02 | 0.45 | 0.96 | 0.92 |  |
| SEC61B | 9 | q22.33 | 4.99E-03 | 3.88E-02 | 0.40 | 1.21 | 0.79 |  |
| ZNF189 | 9 | q31.1 | 5.31E-04 | 7.60E-03 | 0.49 | 1.14 | 0.73 |  |
| MRPL50 | 9 | q31.1 | 7.70E-04 | 9.97E-03 | 0.47 | 1.04 | 0.72 |  |
| RNF20 | 9 | q31.1 | 7.95E-04 | 1.02E-02 | 0.47 | 1.07 | 0.71 |  |
| TEX10 | 9 | q31.1 | 8.89E-04 | 1.10E-02 | 0.47 | 0.91 | 0.72 |  |
| C9orf30 | 9 | q31.1 | 2.64E-03 | 2.47E-02 | 0.43 | 1.06 | 0.74 |  |
| RAD23B | 9 | q31.2 | 4.72E-04 | 6.98E-03 | 0.49 | 1.10 | 0.68 |  |
| CTNNAL1 | 9 | q31.3 | 2.41E-03 | 2.32E-02 | 0.43 | 0.89 | 0.73 |  |
| C9orf5 | 9 | q31.3 | 2.84E-03 | 2.61E-02 | 0.43 | 0.96 | 0.68 |  |
| LPAR1 | 9 | q31.3 | 5.68E-03 | 4.27E-02 | -0.40 | 0.88 | 1.05 |  |
| C9orf91 | 9 | q32 | 5.42E-05 | 1.34E-03 | 0.55 | 1.34 | 0.73 |  |
| WDR31 | 9 | q32 | 1.53E-04 | 2.93E-03 | 0.52 | 1.36 | 0.81 |  |
| SNX30 | 9 | q32 | 2.06E-04 | 3.72E-03 | 0.52 | 1.09 | 0.77 |  |
| C9orf80 | 9 | q32 | 2.28E-04 | 3.99E-03 | 0.51 | 1.13 | 0.76 |  |
| FKBP15 | 9 | q32 | 7.25E-04 | 9.52E-03 | 0.48 | 1.09 | 0.83 |  |
| ALAD | 9 | q32 | 2.63E-03 | 2.46E-02 | 0.43 | 1.42 | 0.81 |  |
| RGS3 | 9 | q32 | 3.20E-03 | 2.83E-02 | 0.42 | 1.07 | 0.96 |  |
| TRIM32 | 9 | q33.1 | 2.91E-06 | 1.40E-04 | 0.62 | 1.18 | 0.78 | 2.34 |
| ASTN2 | 9 | q33.1 | 3.88E-03 | 3.25E-02 | 0.41 | 1.08 | 0.99 | 1.25 |
| MRRF | 9 | q33.2 | 1.44E-08 | 2.38E-06 | 0.72 | 1.52 | 0.75 | 3.36 |
| RBM18 | 9 | q33.2 | 3.43E-08 | 4.57E-06 | 0.70 | 1.50 | 0.73 | 2.74 |
| RC3H2 | 9 | q33.2 | 3.20E-06 | 1.51E-04 | 0.62 | 1.48 | 0.83 | 3.11 |
| NDUFA8 | 9 | q33.2 | 3.27E-05 | 9.11E-04 | 0.57 | 1.79 | 0.70 | 3.30 |
| C5 | 9 | q33.2 | 6.76E-04 | 9.06E-03 | 0.48 | 1.15 | 0.68 | 5.08 |
| PDCL | 9 | q33.2 | 6.83E-03 | 4.88E-02 | 0.39 | 1.13 | 0.93 | 1.09 |
| PSMB7 | 9 | q33.3 | 1.54E-10 | 5.99E-08 | 0.78 | 1.64 | 0.77 | 3.49 |
| MAPKAP1 | 9 | q33.3 | 7.43E-10 | 2.12E-07 | 0.76 | 1.78 | 0.89 | 3.68 |
| ARPC5L | 9 | q33.3 | 1.89E-09 | 4.46E-07 | 0.75 | 1.80 | 0.90 | 3.84 |
| RABEPK | 9 | q33.3 | 1.72E-06 | 9.72E-05 | 0.63 | 1.89 | 0.99 | 4.10 |
| GOLGA1 | 9 | q33.3 | 1.11E-05 | 3.97E-04 | 0.59 | 1.28 | 0.87 | 1.89 |
| ZBTB43 | 9 | q33.3 | 1.28E-05 | 4.44E-04 | 0.59 | 1.45 | 0.92 | 4.13 |
| PPP6C | 9 | q33.3 | 2.73E-05 | 7.89E-04 | 0.57 | 1.78 | 0.96 | 4.01 |
| LRSAM1 | 9 | q33.3 | 3.64E-04 | 5.69E-03 | 0.50 | 1.72 | 0.95 | 3.14 |
| GARNL3 | 9 | q33.3 | 4.27E-04 | 6.45E-03 | 0.49 | 1.52 | 0.96 | 3.09 |
| C9orf126 | 9 | q33.3 | 2.04E-03 | 2.06E-02 | 0.44 | 1.02 | 0.95 | 1.24 |
| LHX2 | 9 | q33.3 | 2.60E-03 | 2.44E-02 | 0.43 | 1.28 | 0.80 | 2.19 |
| ZNF79 | 9 | q33.3 | 2.85E-03 | 2.61E-02 | 0.43 | 1.30 | 1.03 | 1.90 |
| LMX1B | 9 | q33.3 | 3.24E-03 | 2.86E-02 | 0.42 | 1.18 | 1.02 | 1.37 |
| GAPVD1 | 9 | q33.3 | 4.13E-03 | 3.39E-02 | 0.41 | 1.76 | 0.97 | 2.98 |
| FAM125B | 9 | q33.3 | 4.67E-03 | 3.70E-02 | 0.41 | 1.29 | 0.93 | 2.29 |
| RALGPS1 | 9 | q33.3 | 6.17E-03 | 4.54E-02 | 0.39 | 1.01 | 0.95 | 2.69 |
| ZBTB34 | 9 | q33.3 | 6.62E-03 | 4.77E-02 | 0.39 | 1.16 | 0.98 | 1.81 |
| FAM129B | 9 | q33.3-q34.11 | 1.09E-04 | 2.27E-03 | 0.53 | 2.16 | 0.89 | 3.85 |
| ZDHHC12 | 9 | q34.11 | 1.73E-05 | 5.51E-04 | 0.58 | 1.65 | 0.67 |  |
| URM1 | 9 | q34.11 | 2.94E-05 | 8.39E-04 | 0.57 | 1.60 | 0.80 |  |
| PTGES2 | 9 | q34.11 | 3.46E-05 | 9.49E-04 | 0.57 | 1.70 | 0.74 |  |
| SLC25A25 | 9 | q34.11 | 2.78E-04 | 4.64E-03 | 0.51 | 2.20 | 0.84 |  |
| TOR1A | 9 | q34.11 | 2.17E-03 | 2.15E-02 | 0.44 | 1.08 | 0.89 |  |
| NUP188 | 9 | q34.11 | 3.47E-03 | 2.99E-02 | 0.42 | 1.04 | 0.80 |  |
| ODF2 | 9 | q34.11 | 4.95E-03 | 3.86E-02 | 0.40 | 1.30 | 0.82 |  |
| DOLPP1 | 9 | q34.11 | 5.15E-03 | 3.98E-02 | 0.40 | 1.22 | 0.87 |  |
| GOLGA2 | 9 | q34.11 | 6.43E-03 | 4.68E-02 | 0.39 | 1.52 | 0.80 |  |
| UCK1 | 9 | q34.13 | 8.87E-07 | 5.79E-05 | 0.65 | 1.69 | 0.75 |  |
| MED27 | 9 | q34.13 | 4.34E-06 | 1.92E-04 | 0.61 | 1.39 | 0.80 |  |
| C9orf9 | 9 | q34.13 | 1.76E-04 | 3.27E-03 | 0.52 | 3.77 | 0.78 |  |
| TTF1 | 9 | q34.13 | 1.87E-04 | 3.43E-03 | 0.52 | 1.22 | 0.82 |  |
| NUP214 | 9 | q34.13 | 9.19E-04 | 1.13E-02 | 0.47 | 1.36 | 0.91 |  |
| GTF3C4 | 9 | q34.13 | 4.61E-03 | 3.67E-02 | 0.41 | 1.21 | 0.93 |  |
| REXO4 | 9 | q34.2 | 1.34E-05 | 4.57E-04 | 0.59 | 2.12 | 0.84 |  |
| RPL7A | 9 | q34.2 | 7.37E-04 | 9.64E-03 | 0.48 | 1.83 | 0.74 |  |
| GTF3C5 | 9 | q34.2 | 1.90E-03 | 1.96E-02 | 0.44 | 1.05 | 0.87 |  |
| MED22 | 9 | q34.2 | 3.77E-03 | 3.19E-02 | 0.41 | 1.98 | 0.89 |  |
| MRPS2 | 9 | q34.3 | 9.59E-06 | 3.56E-04 | 0.60 | 1.57 | 0.88 |  |
| CACNA1B | 9 | q34.3 | 1.35E-03 | 1.50E-02 | 0.45 | 1.09 | 0.94 |  |
| SSNA1 | 9 | q34.3 | 1.61E-03 | 1.71E-02 | 0.45 | 2.39 | 0.78 |  |
| AGPAT2 | 9 | q34.3 | 2.31E-03 | 2.24E-02 | 0.43 | 3.58 | 0.75 |  |
| ZMYND19 | 9 | q34.3 | 3.90E-03 | 3.26E-02 | 0.41 | 2.05 | 0.79 |  |
| MAN1B1 | 9 | q34.3 | 5.21E-03 | 4.02E-02 | 0.40 | 3.26 | 0.93 |  |
| TMEM210 | 9 | q34.3 | 5.27E-03 | 4.06E-02 | 0.40 | 1.21 | 0.94 |  |
| NDOR1 | 9 | q34.3 | 5.72E-03 | 4.30E-02 | 0.40 | 2.70 | 0.83 |  |
| CUL2 | 10 | p11.21 | 2.41E-03 | 2.32E-02 | 0.43 | 1.23 | 0.78 |  |
| KIF5B | 10 | p11.22 | 7.97E-05 | 1.81E-03 | 0.54 | 1.41 | 0.74 |  |
| RP11-192P3.1 | 10 | p11.22 | 7.26E-04 | 9.53E-03 | 0.48 | 1.52 | 0.84 |  |
| MTPAP | 10 | p11.23 | 6.81E-03 | 4.88E-02 | 0.39 | 1.23 | 0.96 |  |
| PDSS1 | 10 | p12.1 | 2.25E-06 | 1.16E-04 | 0.63 | 1.29 | 0.87 |  |
| THNSL1 | 10 | p12.1 | 5.04E-05 | 1.27E-03 | 0.56 | 1.29 | 0.67 |  |
| YME1L1 | 10 | p12.1 | 1.61E-04 | 3.05E-03 | 0.52 | 1.42 | 0.63 |  |
| PRTFDC1 | 10 | p12.1 | 2.10E-04 | 3.76E-03 | 0.52 | 1.55 | 0.81 |  |
| SSH3BP | 10 | p12.1 | 2.40E-04 | 4.15E-03 | 0.51 | 1.56 | 0.81 |  |
| MASTL | 10 | p12.1 | 3.39E-04 | 5.41E-03 | 0.50 | 1.33 | 0.88 |  |
| WAC | 10 | p12.1 | 4.40E-04 | 6.62E-03 | 0.49 | 1.30 | 0.74 |  |
| RAB18 | 10 | p12.1 | 3.71E-03 | 3.15E-02 | 0.42 | 1.32 | 0.69 |  |
| OTUD1 | 10 | p12.2 | 5.47E-04 | 7.78E-03 | 0.49 | 1.11 | 0.79 |  |
| NSUN6 | 10 | p12.31 | 4.56E-05 | 1.18E-03 | 0.56 | 1.28 | 0.77 |  |
| ARL5B | 10 | p12.31 | 8.10E-05 | 1.83E-03 | 0.54 | 1.18 | 0.92 |  |
| NEBL | 10 | p12.31 | 1.15E-03 | 1.34E-02 | 0.46 | 1.31 | 0.55 |  |
| MLLT10 | 10 | p12.31 | 2.62E-03 | 2.46E-02 | 0.43 | 1.09 | 0.80 |  |
| STAM | 10 | p12.33 | 2.23E-03 | 2.18E-02 | 0.44 | 1.22 | 0.74 |  |
| PTPLA | 10 | p12.33 | 3.22E-03 | 2.85E-02 | 0.42 | 1.43 | 0.95 |  |
| CDC123 | 10 | p13 | 2.87E-07 | 2.50E-05 | 0.67 | 1.69 | 0.71 |  |
| NUDT5 | 10 | p13 | 7.54E-07 | 5.10E-05 | 0.65 | 1.59 | 0.68 |  |
| SEPHS1 | 10 | p13 | 9.16E-07 | 5.93E-05 | 0.65 | 1.61 | 0.79 |  |
| HSPA14 | 10 | p13 | 1.76E-06 | 9.82E-05 | 0.63 | 1.64 | 0.82 |  |
| PRPF18 | 10 | p13 | 7.92E-06 | 3.09E-04 | 0.60 | 1.55 | 0.77 |  |
| MCM10 | 10 | p13 | 5.83E-05 | 1.41E-03 | 0.55 | 1.76 | 0.79 |  |
| RSU1 | 10 | p13 | 3.33E-04 | 5.35E-03 | 0.50 | 1.61 | 0.79 |  |
| OPTN | 10 | p13 | 4.01E-04 | 6.15E-03 | 0.50 | 2.63 | 0.82 |  |
| CAMK1D | 10 | p13 | 1.32E-03 | 1.48E-02 | 0.45 | 1.38 | 0.88 |  |
| ARMETL1 | 10 | p13 | 1.88E-03 | 1.95E-02 | 0.44 | 1.13 | 0.91 |  |
| PHYH | 10 | p13 | 2.19E-03 | 2.16E-02 | 0.44 | 1.59 | 0.87 |  |
| NMT2 | 10 | p13 | 4.83E-03 | 3.79E-02 | 0.40 | 1.88 | 0.89 |  |
| ATP5C1 | 10 | p14 | 3.05E-06 | 1.45E-04 | 0.62 | 1.39 | 0.71 |  |
| CUGBP2 | 10 | p14 | 9.35E-06 | 3.49E-04 | 0.60 | 1.37 | 0.95 | 7.82 |
| DHTKD1 | 10 | p14 | 3.68E-04 | 5.75E-03 | 0.50 | 1.78 | 0.86 |  |
| UPF2 | 10 | p14 | 1.40E-03 | 1.54E-02 | 0.45 | 1.54 | 0.81 |  |
| RBM17 | 10 | p15.1 | 2.60E-03 | 2.45E-02 | 0.43 | 1.24 | 0.83 |  |
| PFKP | 10 | p15.2 | 3.03E-04 | 4.97E-03 | 0.50 | 1.84 | 0.75 | 6.41 |
| PITRM1 | 10 | p15.2 | 5.92E-04 | 8.25E-03 | 0.48 | 1.12 | 0.83 | 1.79 |
| GTPBP4 | 10 | p15.3 | 6.87E-05 | 1.61E-03 | 0.55 | 1.57 | 0.78 | 3.06 |
| WDR37 | 10 | p15.3 | 3.01E-04 | 4.94E-03 | 0.50 | 1.32 | 0.95 | 2.15 |
| IDI1 | 10 | p15.3 | 9.33E-04 | 1.14E-02 | 0.47 | 1.35 | 0.87 | 2.90 |
| ZMYND11 | 10 | p15.3 | 2.44E-03 | 2.34E-02 | 0.43 | 1.06 | 1.00 | 1.25 |
| LARP5 | 10 | p15.3 | 3.02E-03 | 2.72E-02 | 0.42 | 1.19 | 0.84 | 1.98 |
| ADARB2 | 10 | p15.3 | 6.50E-03 | 4.72E-02 | 0.39 | 1.05 | 0.96 | 1.07 |
| ZNF32 | 10 | q11.21 | 2.73E-04 | 4.58E-03 | 0.51 | 1.95 | 0.84 |  |
| PCDH15 | 10 | q21.1 | 2.22E-03 | 2.18E-02 | 0.44 | 1.07 | 0.96 |  |
| CCDC6 | 10 | q21.2 | 6.38E-04 | 8.73E-03 | 0.48 | 1.14 | 0.76 |  |
| LRRTM3 | 10 | q21.3 | 6.88E-03 | 4.91E-02 | -0.39 | 0.90 | 1.08 |  |
| PPA1 | 10 | q22.1 | 1.50E-03 | 1.63E-02 | 0.45 |  | 0.80 |  |
| VPS26A | 10 | q22.1 | 1.60E-03 | 1.71E-02 | 0.45 |  | 0.83 |  |
| DDX50 | 10 | q22.1 | 5.37E-03 | 4.11E-02 | 0.40 |  | 0.82 |  |
| DNAJB12 | 10 | q22.1 | 6.68E-03 | 4.80E-02 | 0.39 |  | 0.84 |  |
| SEC24C | 10 | q22.2 | 9.06E-04 | 1.12E-02 | 0.47 |  | 0.81 |  |
| VCL | 10 | q22.2 | 9.29E-04 | 1.14E-02 | 0.47 | 2.36 | 0.85 |  |
| CHCHD1 | 10 | q22.2 | 1.39E-03 | 1.54E-02 | 0.45 |  | 0.82 |  |
| KIAA0913 | 10 | q22.2 | 1.74E-03 | 1.82E-02 | 0.44 |  | 0.76 |  |
| MRPS16 | 10 | q22.2 | 3.48E-03 | 3.00E-02 | 0.42 |  | 0.90 |  |
| FAM35A | 10 | q23.2 | 4.43E-03 | 3.57E-02 | 0.41 | 1.07 | 0.79 |  |
| PTEN | 10 | q23.31 | 5.52E-03 | 4.18E-02 | 0.40 | 1.21 | 0.74 |  |
| IDE | 10 | q23.33 | 2.87E-03 | 2.62E-02 | 0.43 |  | 0.87 |  |
| EXOC6 | 10 | q23.33 | 5.74E-03 | 4.31E-02 | 0.40 |  | 0.80 |  |
| PLCE1 | 10 | q23.33 | 5.99E-03 | 4.44E-02 | 0.40 |  | 0.75 |  |
| TCTN3 | 10 | q24.1 | 1.09E-05 | 3.93E-04 | 0.59 |  | 0.82 |  |
| UBTD1 | 10 | q24.1-q24.2 | 4.50E-03 | 3.61E-02 | 0.41 |  | 0.72 |  |
| CUEDC2 | 10 | q24.32 | 7.09E-04 | 9.36E-03 | 0.48 |  | 0.85 |  |
| FBXL15 | 10 | q24.32 | 3.08E-03 | 2.76E-02 | 0.42 |  | 0.77 |  |
| ZDHHC6 | 10 | q25.2 | 3.81E-04 | 5.91E-03 | 0.50 | 1.24 | 0.83 |  |
| TIAL1 | 10 | q26.11 | 1.47E-08 | 2.39E-06 | 0.72 | 1.41 | 0.76 |  |
| FAM45A | 10 | q26.11 | 2.65E-03 | 2.48E-02 | 0.43 | 1.40 | 0.80 |  |
| RGS10 | 10 | q26.11 | 5.36E-03 | 4.10E-02 | 0.40 | 2.09 | 0.91 |  |
| SEC23IP | 10 | q26.11-q26.12 | 2.99E-05 | 8.52E-04 | 0.57 | 1.49 | 0.76 |  |
| WDR11 | 10 | q26.12 | 1.51E-04 | 2.90E-03 | 0.53 | 1.49 | 0.87 | 2.42 |
| ATE1 | 10 | q26.13 | 0.00E+00 | 0.00E+00 | 0.89 | 2.12 | 0.93 | 5.27 |
| PLEKHA1 | 10 | q26.13 | 9.22E-13 | 9.06E-10 | 0.83 | 1.86 | 0.96 | 3.64 |
| BTBD16 | 10 | q26.13 | 1.59E-12 | 1.41E-09 | 0.82 | 1.52 | 0.94 | 2.27 |
| RP11-107C16.2 | 10 | q26.13 | 3.61E-11 | 1.74E-08 | 0.79 | 1.36 | 0.96 | 2.01 |
| C10orf120 | 10 | q26.13 | 9.74E-09 | 1.70E-06 | 0.72 | 1.31 | 0.98 | 1.88 |
| TACC2 | 10 | q26.13 | 6.04E-08 | 7.08E-06 | 0.69 | 3.41 | 0.65 | 6.78 |
| NSMCE4A | 10 | q26.13 | 7.81E-08 | 8.69E-06 | 0.69 | 1.88 | 0.81 | 4.83 |
| LHPP | 10 | q26.13 | 2.00E-06 | 1.07E-04 | 0.63 | 1.78 | 0.72 | 2.88 |
| C10orf88 | 10 | q26.13 | 1.57E-05 | 5.16E-04 | 0.59 | 1.84 | 0.89 | 2.98 |
| FAM24B | 10 | q26.13 | 1.56E-04 | 2.98E-03 | 0.52 | 1.20 | 0.96 | 1.56 |
| BUB3 | 10 | q26.13 | 2.57E-04 | 4.38E-03 | 0.51 | 1.59 | 0.87 | 2.81 |
| FAM175B | 10 | q26.13 | 1.02E-03 | 1.22E-02 | 0.46 | 1.64 | 0.91 | 2.78 |
| IKZF5 | 10 | q26.13 | 1.10E-03 | 1.29E-02 | 0.46 | 1.23 | 0.88 | 1.57 |
| CUZD1 | 10 | q26.13 | 1.23E-03 | 1.40E-02 | 0.46 | 1.14 | 1.00 | 1.30 |
| OAT | 10 | q26.13 | 6.84E-03 | 4.89E-02 | 0.39 | 2.47 | 0.87 | 3.67 |
| FOXI2 | 10 | q26.2 | 6.02E-03 | 4.46E-02 | 0.39 | 0.96 | 0.95 |  |
| ZNF511 | 10 | q26.3 | 6.91E-10 | 2.01E-07 | 0.76 | 2.45 | 0.80 | 4.70 |
| TUBGCP2 | 10 | q26.3 | 3.04E-07 | 2.63E-05 | 0.67 | 2.67 | 0.89 | 5.10 |
| PAOX | 10 | q26.3 | 1.68E-06 | 9.57E-05 | 0.63 | 1.59 | 0.89 | 3.09 |
| ECHS1 | 10 | q26.3 | 9.29E-06 | 3.49E-04 | 0.60 | 2.38 | 0.80 | 5.06 |
| RP11-108K14.4 | 10 | q26.3 | 1.55E-05 | 5.14E-04 | 0.59 | 1.28 | 0.99 | 1.63 |
| MTG1 | 10 | q26.3 | 2.68E-05 | 7.77E-04 | 0.57 | 1.86 | 1.03 | 3.73 |
| SPRN | 10 | q26.3 | 1.28E-04 | 2.59E-03 | 0.53 | 1.17 | 0.98 | 1.35 |
| PPP2R2D | 10 | q26.3 | 2.29E-04 | 4.00E-03 | 0.51 | 2.19 | 0.82 |  |
| CYP2E1 | 10 | q26.3 | 8.00E-04 | 1.02E-02 | 0.47 | 1.92 | 0.98 | 5.11 |
| STK32C | 10 | q26.3 | 5.65E-03 | 4.26E-02 | 0.40 | 1.19 | 0.89 |  |
| NDUFS3 | 11 | p11.2 | 9.49E-05 | 2.05E-03 | 0.54 | 1.32 | 0.85 |  |
| CKAP5 | 11 | p11.2 | 7.67E-04 | 9.96E-03 | 0.47 | 1.49 | 0.86 |  |
| NUP160 | 11 | p11.2 | 1.19E-03 | 1.37E-02 | 0.46 | 1.44 | 0.81 |  |
| HARBI1 | 11 | p11.2 | 1.20E-03 | 1.38E-02 | 0.46 | 1.13 | 0.97 |  |
| KBTBD4 | 11 | p11.2 | 2.49E-03 | 2.37E-02 | 0.43 | 1.14 | 0.80 |  |
| MADD | 11 | p11.2 | 2.62E-03 | 2.46E-02 | 0.43 | 1.36 | 0.86 |  |
| PSMC3 | 11 | p11.2 | 2.76E-03 | 2.55E-02 | 0.43 | 1.32 | 0.83 |  |
| RAPSN | 11 | p11.2 | 3.35E-03 | 2.92E-02 | -0.42 | 0.95 | 1.04 |  |
| PEX16 | 11 | p11.2 | 4.77E-03 | 3.75E-02 | 0.40 | 1.17 | 0.90 |  |
| C11orf74 | 11 | p12 | 3.78E-06 | 1.74E-04 | 0.62 | 1.70 | 0.58 |  |
| API5L1 | 11 | p12 | 3.82E-04 | 5.92E-03 | 0.50 | 1.27 | 0.85 |  |
| TRAF6 | 11 | p12 | 6.47E-04 | 8.82E-03 | 0.48 | 1.09 | 0.89 |  |
| LRRC4C | 11 | p12 | 1.72E-03 | 1.81E-02 | 0.45 | 1.12 | 0.77 |  |
| IMMP1L | 11 | p13 | 1.93E-09 | 4.47E-07 | 0.74 | 1.35 | 0.84 | 4.62 |
| ELP4 | 11 | p13 | 6.13E-09 | 1.22E-06 | 0.73 | 1.31 | 0.90 | 3.42 |
| EIF3M | 11 | p13 | 1.32E-07 | 1.33E-05 | 0.68 | 2.42 | 0.60 | 3.58 |
| PDHX | 11 | p13 | 2.02E-07 | 1.88E-05 | 0.67 | 1.54 | 0.77 | 2.44 |
| DNAJC24 | 11 | p13 | 1.05E-06 | 6.52E-05 | 0.64 | 1.16 | 0.78 | 2.99 |
| FBXO3 | 11 | p13 | 1.88E-06 | 1.03E-04 | 0.63 | 1.54 | 0.83 | 2.07 |
| NAT10 | 11 | p13 | 2.33E-05 | 6.96E-04 | 0.58 | 1.48 | 0.88 | 2.07 |
| CSTF3 | 11 | p13 | 3.47E-05 | 9.52E-04 | 0.57 | 1.71 | 0.85 | 2.89 |
| CAPRIN1 | 11 | p13 | 6.09E-05 | 1.47E-03 | 0.55 | 1.58 | 0.80 | 1.96 |
| PRRG4 | 11 | p13 | 8.04E-05 | 1.82E-03 | 0.54 | 1.51 | 0.86 | 2.74 |
| TCP11L1 | 11 | p13 | 8.52E-05 | 1.90E-03 | 0.54 | 1.18 | 0.87 | 2.07 |
| APIP | 11 | p13 | 3.28E-04 | 5.28E-03 | 0.50 | 1.46 | 0.72 | 1.62 |
| TRIM44 | 11 | p13 | 8.61E-04 | 1.08E-02 | 0.47 | 1.20 | 0.73 |  |
| HIPK3 | 11 | p13 | 3.40E-03 | 2.95E-02 | 0.42 | 1.08 | 1.01 | 1.15 |
| LMO2 | 11 | p13 | 6.42E-03 | 4.67E-02 | 0.39 | 1.23 | 0.79 | 1.88 |
| CCDC34 | 11 | p14.1 | 5.60E-03 | 4.23E-02 | 0.40 | 1.36 | 0.90 |  |
| BBOX1 | 11 | p14.2 | 5.65E-04 | 8.00E-03 | 0.48 | 1.37 | 0.81 |  |
| FANCF | 11 | p14.3 | 2.27E-04 | 3.97E-03 | 0.51 | 1.12 | 0.94 | 1.24 |
| SAAL1 | 11 | p15.1 | 6.94E-13 | 7.06E-10 | 0.83 | 1.71 | 0.67 | 4.25 |
| TSG101 | 11 | p15.1 | 3.67E-10 | 1.26E-07 | 0.77 | 2.19 | 0.64 | 4.29 |
| SPTY2D1 | 11 | p15.1 | 8.23E-09 | 1.51E-06 | 0.73 | 1.62 | 0.78 | 2.63 |
| LDHA | 11 | p15.1 | 3.19E-08 | 4.35E-06 | 0.70 | 1.65 | 0.75 | 3.02 |
| SERGEF | 11 | p15.1 | 3.85E-08 | 5.01E-06 | 0.70 | 1.77 | 0.66 | 8.76 |
| UEVLD | 11 | p15.1 | 1.03E-07 | 1.10E-05 | 0.69 | 1.48 | 0.84 | 1.93 |
| ZDHHC13 | 11 | p15.1 | 4.12E-07 | 3.23E-05 | 0.66 | 1.71 | 0.73 | 3.26 |
| PIK3C2A | 11 | p15.1 | 4.17E-06 | 1.87E-04 | 0.62 | 1.20 | 0.88 | 1.58 |
| LDHC | 11 | p15.1 | 4.69E-06 | 2.04E-04 | 0.61 | 1.19 | 0.99 | 1.75 |
| PRMT3 | 11 | p15.1 | 6.52E-06 | 2.62E-04 | 0.61 | 1.26 | 0.81 | 1.90 |
| C11orf58 | 11 | p15.1 | 8.33E-06 | 3.22E-04 | 0.60 | 1.20 | 0.90 | 1.49 |
| HTATIP2 | 11 | p15.1 | 4.04E-05 | 1.07E-03 | 0.56 | 1.49 | 0.82 | 5.49 |
| HPS5 | 11 | p15.1 | 9.62E-05 | 2.08E-03 | 0.54 | 1.31 | 0.88 | 2.32 |
| ABCC8 | 11 | p15.1 | 2.25E-04 | 3.96E-03 | 0.51 | 1.50 | 0.69 | 7.15 |
| RPS13P2 | 11 | p15.1 | 4.49E-04 | 6.72E-03 | 0.49 | 1.45 | 0.69 | 1.76 |
| PLEKHA7 | 11 | p15.1 | 8.04E-04 | 1.03E-02 | 0.47 | 1.21 | 0.87 | 1.46 |
| GTF2H1 | 11 | p15.1 | 1.23E-03 | 1.40E-02 | 0.46 | 1.43 | 0.90 | 1.67 |
| KCNJ11 | 11 | p15.1 | 1.75E-03 | 1.83E-02 | 0.44 | 1.23 | 0.90 | 1.39 |
| TMEM86A | 11 | p15.1 | 3.36E-03 | 2.93E-02 | 0.42 | 1.21 | 1.02 | 1.89 |
| USH1C | 11 | p15.1 | 5.13E-03 | 3.96E-02 | 0.40 | 1.03 | 1.00 | 1.28 |
| BTBD10 | 11 | p15.2 | 7.21E-07 | 4.92E-05 | 0.65 | 1.46 | 0.75 | 2.39 |
| COPB1 | 11 | p15.2 | 1.76E-05 | 5.56E-04 | 0.58 | 1.64 | 0.68 | 2.33 |
| PSMA1 | 11 | p15.2 | 1.65E-04 | 3.10E-03 | 0.52 | 1.40 | 0.74 | 2.04 |
| CYP2R1 | 11 | p15.2 | 1.73E-04 | 3.22E-03 | 0.52 | 1.77 | 0.77 | 3.09 |
| ARNTL | 11 | p15.2 | 2.94E-03 | 2.67E-02 | 0.42 | 1.36 | 0.86 | 1.58 |
| ZBED5 | 11 | p15.3 | 3.97E-07 | 3.14E-05 | 0.66 | 1.73 | 0.68 | 1.90 |
| CTR9 | 11 | p15.3 | 6.12E-05 | 1.47E-03 | 0.55 | 1.50 | 0.76 | 1.68 |
| EIF4G2 | 11 | p15.3 | 6.20E-05 | 1.48E-03 | 0.55 | 1.78 | 0.68 | 1.82 |
| USP47 | 11 | p15.3 | 3.10E-04 | 5.05E-03 | 0.50 | 1.44 | 0.84 | 1.83 |
| PARVA | 11 | p15.3 | 5.48E-03 | 4.16E-02 | 0.40 | 1.25 | 0.81 | 1.94 |
| RRP8 | 11 | p15.4 | 1.51E-10 | 5.95E-08 | 0.78 | 1.53 | 0.74 | 3.84 |
| TMEM9B | 11 | p15.4 | 8.28E-09 | 1.51E-06 | 0.72 | 1.42 | 0.68 |  |
| ZNF214 | 11 | p15.4 | 1.38E-08 | 2.30E-06 | 0.72 | 1.35 | 0.91 | 1.78 |
| TAF10 | 11 | p15.4 | 1.80E-08 | 2.77E-06 | 0.71 | 1.41 | 0.73 | 3.83 |
| MRPL17 | 11 | p15.4 | 1.93E-07 | 1.81E-05 | 0.68 | 1.61 | 0.74 | 2.98 |
| C11orf17 | 11 | p15.4 | 5.50E-07 | 4.05E-05 | 0.66 | 1.84 | 0.73 |  |
| RRM1 | 11 | p15.4 | 1.02E-06 | 6.41E-05 | 0.64 | 1.78 | 0.74 |  |
| DENND5A | 11 | p15.4 | 4.03E-06 | 1.82E-04 | 0.62 | 1.38 | 0.75 |  |
| CYB5R2 | 11 | p15.4 | 6.51E-06 | 2.62E-04 | 0.61 | 1.16 | 0.97 | 1.75 |
| TMEM41B | 11 | p15.4 | 9.03E-06 | 3.42E-04 | 0.60 | 1.56 | 0.70 |  |
| STIM1 | 11 | p15.4 | 1.99E-05 | 6.10E-04 | 0.58 | 1.89 | 0.76 |  |
| RNF141 | 11 | p15.4 | 3.06E-05 | 8.68E-04 | 0.57 | 1.62 | 0.83 | 2.00 |
| WEE1 | 11 | p15.4 | 3.41E-05 | 9.40E-04 | 0.57 | 2.42 | 0.75 |  |
| PPFIBP2 | 11 | p15.4 | 8.69E-05 | 1.92E-03 | 0.54 | 2.22 | 0.90 | 6.72 |
| SWAP70 | 11 | p15.4 | 1.97E-04 | 3.58E-03 | 0.52 | 1.64 | 0.72 |  |
| NLRP14 | 11 | p15.4 | 2.08E-04 | 3.74E-03 | 0.52 | 1.09 | 0.96 | 1.27 |
| TRIM68 | 11 | p15.4 | 3.36E-04 | 5.38E-03 | 0.50 | 1.58 | 0.73 |  |
| FAM160A2 | 11 | p15.4 | 5.51E-04 | 7.83E-03 | 0.48 | 1.31 | 0.79 |  |
| ILK | 11 | p15.4 | 6.03E-04 | 8.36E-03 | 0.48 | 1.23 | 0.88 | 2.26 |
| AC090587.2 | 11 | p15.4 | 6.49E-04 | 8.84E-03 | 0.48 | 1.55 | 0.72 |  |
| EIF3F | 11 | p15.4 | 6.72E-04 | 9.00E-03 | 0.48 | 1.54 | 0.67 |  |
| ZNF215 | 11 | p15.4 | 7.80E-04 | 1.01E-02 | 0.47 | 1.07 | 0.98 | 1.53 |
| ZNF195 | 11 | p15.4 | 8.07E-04 | 1.03E-02 | 0.47 | 1.37 | 0.88 |  |
| NUP98 | 11 | p15.4 | 3.23E-03 | 2.85E-02 | 0.42 | 1.17 | 0.89 |  |
| RIC8A | 11 | p15.5 | 2.43E-04 | 4.19E-03 | 0.51 | 1.22 | 0.79 |  |
| TOLLIP | 11 | p15.5 | 6.11E-04 | 8.46E-03 | 0.48 | 1.52 | 0.85 |  |
| PSMD13 | 11 | p15.5 | 1.13E-03 | 1.33E-02 | 0.46 | 1.20 | 0.84 |  |
| TSSC4 | 11 | p15.5 | 2.30E-03 | 2.24E-02 | 0.43 | 1.34 | 0.79 |  |
| HRAS | 11 | p15.5 | 2.44E-03 | 2.34E-02 | 0.43 | 1.62 | 0.85 |  |
| PDDC1 | 11 | p15.5 | 3.14E-03 | 2.79E-02 | 0.42 | 1.14 | 0.85 |  |
| TNKS1BP1 | 11 | q11 | 5.46E-05 | 1.34E-03 | 0.55 | 1.39 | 0.73 |  |
| OR5G5P | 11 | q11 | 1.21E-03 | 1.38E-02 | 0.46 | 1.01 | 0.93 |  |
| STX3 | 11 | q12.1 | 2.87E-09 | 6.18E-07 | 0.74 | 1.53 | 0.67 | 3.34 |
| PATL1 | 11 | q12.1 | 2.10E-08 | 3.12E-06 | 0.71 | 1.40 | 0.73 | 1.98 |
| MRPL16 | 11 | q12.1 | 4.62E-07 | 3.54E-05 | 0.66 | 1.21 | 0.70 | 2.25 |
| OSBP | 11 | q12.1 | 2.40E-06 | 1.22E-04 | 0.63 | 1.14 | 0.76 |  |
| ZFP91 | 11 | q12.1 | 2.51E-05 | 7.39E-04 | 0.57 | 1.14 | 0.73 |  |
| TMX2 | 11 | q12.1 | 6.68E-05 | 1.57E-03 | 0.55 | 1.55 | 0.68 |  |
| ZDHHC5 | 11 | q12.1 | 1.49E-04 | 2.88E-03 | 0.53 | 1.37 | 0.79 |  |
| CLP1 | 11 | q12.1 | 2.77E-04 | 4.62E-03 | 0.51 | 1.21 | 0.83 |  |
| UBE2L6 | 11 | q12.1 | 3.68E-04 | 5.75E-03 | 0.50 | 1.43 | 0.64 |  |
| MED19 | 11 | q12.1 | 8.12E-04 | 1.03E-02 | 0.47 | 1.38 | 0.75 |  |
| FAM111A | 11 | q12.1 | 1.86E-03 | 1.92E-02 | 0.44 | 1.20 | 0.72 |  |
| FAM111B | 11 | q12.1 | 2.00E-03 | 2.03E-02 | 0.44 | 1.15 | 0.86 |  |
| C11orf10 | 11 | q12.2 | 8.50E-10 | 2.31E-07 | 0.76 | 1.66 | 0.75 |  |
| C11orf79 | 11 | q12.2 | 1.53E-07 | 1.51E-05 | 0.68 | 1.83 | 0.71 |  |
| DAK | 11 | q12.2 | 2.30E-07 | 2.06E-05 | 0.67 | 2.05 | 0.71 |  |
| CYBASC3 | 11 | q12.2 | 1.11E-06 | 6.80E-05 | 0.64 | 1.95 | 0.75 |  |
| TMEM138 | 11 | q12.2 | 6.11E-06 | 2.51E-04 | 0.61 | 1.49 | 0.73 |  |
| PRPF19 | 11 | q12.2 | 2.22E-05 | 6.67E-04 | 0.58 | 1.53 | 0.65 |  |
| DDB1 | 11 | q12.2 | 1.78E-04 | 3.29E-03 | 0.52 | 1.97 | 0.80 |  |
| FEN1 | 11 | q12.2 | 4.54E-04 | 6.79E-03 | 0.49 | 2.25 | 0.89 |  |
| AP003108.3-2 | 11 | q12.2 | 1.42E-03 | 1.56E-02 | 0.45 | 1.10 | 0.95 |  |
| TMEM109 | 11 | q12.2 | 2.78E-03 | 2.56E-02 | 0.43 | 1.37 | 0.90 |  |
| DAGLA | 11 | q12.2 | 2.79E-03 | 2.57E-02 | 0.43 | 1.44 | 0.93 |  |
| CPSF7 | 11 | q12.2 | 6.82E-03 | 4.88E-02 | 0.39 | 1.06 | 0.96 |  |
| STX5 | 11 | q12.3 | 1.08E-07 | 1.14E-05 | 0.69 | 1.58 | 0.74 |  |
| TMEM179B | 11 | q12.3 | 1.05E-05 | 3.82E-04 | 0.59 | 2.22 | 0.77 |  |
| C11orf48 | 11 | q12.3 | 1.44E-05 | 4.84E-04 | 0.59 | 1.68 | 0.78 |  |
| POLR2G | 11 | q12.3 | 3.88E-05 | 1.03E-03 | 0.56 | 1.65 | 0.75 |  |
| EEF1G | 11 | q12.3 | 6.60E-05 | 1.55E-03 | 0.55 | 1.36 | 0.78 |  |
| GANAB | 11 | q12.3 | 1.58E-04 | 2.99E-03 | 0.52 | 2.00 | 0.66 |  |
| B3GAT3 | 11 | q12.3 | 1.64E-04 | 3.09E-03 | 0.52 | 1.16 | 0.76 |  |
| UBXN1 | 11 | q12.3 | 1.73E-04 | 3.22E-03 | 0.52 | 1.22 | 0.82 |  |
| WDR74 | 11 | q12.3 | 4.17E-04 | 6.36E-03 | 0.49 | 1.26 | 0.73 |  |
| TAF6L | 11 | q12.3 | 5.26E-04 | 7.56E-03 | 0.49 | 1.36 | 0.71 |  |
| INTS5 | 11 | q12.3 | 7.43E-04 | 9.71E-03 | 0.47 | 1.11 | 0.80 |  |
| AHNAK | 11 | q12.3 | 9.34E-04 | 1.14E-02 | 0.47 | 2.63 | 0.83 |  |
| BSCL2 | 11 | q12.3 | 1.49E-03 | 1.62E-02 | 0.45 | 1.67 | 0.70 |  |
| TTC9C | 11 | q12.3 | 4.00E-03 | 3.32E-02 | 0.41 | 1.16 | 0.91 |  |
| FTHL16 | 11 | q12.3 | 6.69E-03 | 4.80E-02 | 0.39 | 1.72 | 0.69 |  |
| ATL3 | 11 | q12.3-q13.1 | 3.80E-03 | 3.20E-02 | 0.41 | 0.81 | 0.65 |  |
| CFL1 | 11 | q13.1 | 2.39E-10 | 8.83E-08 | 0.77 | 1.47 | 0.77 |  |
| SF3B2 | 11 | q13.1 | 2.09E-09 | 4.79E-07 | 0.74 | 1.53 | 0.74 |  |
| FIBP | 11 | q13.1 | 3.18E-09 | 6.76E-07 | 0.74 | 1.78 | 0.64 |  |
| DPF2 | 11 | q13.1 | 4.45E-08 | 5.59E-06 | 0.70 | 1.34 | 0.74 |  |
| SSSCA1 | 11 | q13.1 | 5.60E-07 | 4.09E-05 | 0.66 | 1.82 | 0.81 |  |
| PRDX5 | 11 | q13.1 | 1.01E-06 | 6.38E-05 | 0.64 | 1.29 | 0.73 |  |
| RTN3 | 11 | q13.1 | 1.61E-06 | 9.19E-05 | 0.64 | 1.09 | 0.65 |  |
| DRAP1 | 11 | q13.1 | 2.49E-06 | 1.25E-04 | 0.63 | 1.56 | 0.75 |  |
| KAT5 | 11 | q13.1 | 3.94E-06 | 1.79E-04 | 0.62 | 1.35 | 0.86 |  |
| FAM89B | 11 | q13.1 | 6.35E-06 | 2.58E-04 | 0.61 | 1.52 | 0.68 |  |
| FAU | 11 | q13.1 | 9.53E-06 | 3.55E-04 | 0.60 | 1.24 | 0.84 |  |
| PPP1R14B | 11 | q13.1 | 1.04E-05 | 3.81E-04 | 0.59 | 1.08 | 0.68 |  |
| SCYL1 | 11 | q13.1 | 1.27E-05 | 4.42E-04 | 0.59 | 1.69 | 0.76 |  |
| C11orf68 | 11 | q13.1 | 1.30E-05 | 4.49E-04 | 0.59 | 1.35 | 0.77 |  |
| SAC3D1 | 11 | q13.1 | 1.45E-05 | 4.86E-04 | 0.59 | 1.16 | 0.75 |  |
| PCNXL3 | 11 | q13.1 | 1.78E-05 | 5.59E-04 | 0.58 | 1.53 | 0.74 |  |
| CCDC85B | 11 | q13.1 | 3.38E-05 | 9.36E-04 | 0.57 | 1.55 | 0.85 |  |
| COX8A | 11 | q13.1 | 4.44E-05 | 1.15E-03 | 0.56 | 1.07 | 0.79 |  |
| SYVN1 | 11 | q13.1 | 5.38E-05 | 1.33E-03 | 0.55 | 1.40 | 0.71 |  |
| FKBP2 | 11 | q13.1 | 6.01E-05 | 1.45E-03 | 0.55 | 0.96 | 0.66 |  |
| ZFPL1 | 11 | q13.1 | 6.99E-05 | 1.63E-03 | 0.55 | 1.14 | 0.77 |  |
| TRPT1 | 11 | q13.1 | 8.40E-05 | 1.88E-03 | 0.54 | 1.12 | 0.66 |  |
| MRPL49 | 11 | q13.1 | 8.48E-05 | 1.89E-03 | 0.54 | 1.71 | 0.75 |  |
| CDCA5 | 11 | q13.1 | 9.78E-05 | 2.10E-03 | 0.54 | 1.31 | 0.69 |  |
| AP005668.2 | 11 | q13.1 | 1.13E-04 | 2.34E-03 | 0.53 | 1.66 | 0.75 |  |
| SNX15 | 11 | q13.1 | 1.22E-04 | 2.48E-03 | 0.53 | 1.22 | 0.84 |  |
| AP001453.6-1 | 11 | q13.1 | 1.39E-04 | 2.73E-03 | 0.53 | 1.52 | 0.84 |  |
| POLA2 | 11 | q13.1 | 1.57E-04 | 2.99E-03 | 0.52 | 1.20 | 0.83 |  |
| RELA | 11 | q13.1 | 1.73E-04 | 3.21E-03 | 0.52 | 1.36 | 0.88 |  |
| GPR137 | 11 | q13.1 | 2.14E-04 | 3.81E-03 | 0.51 | 1.05 | 0.66 |  |
| MUS81 | 11 | q13.1 | 2.65E-04 | 4.48E-03 | 0.51 | 1.39 | 0.83 |  |
| EHD1 | 11 | q13.1 | 3.11E-04 | 5.05E-03 | 0.50 | 1.14 | 0.73 |  |
| MAP4K2 | 11 | q13.1 | 3.42E-04 | 5.45E-03 | 0.50 | 1.20 | 0.72 |  |
| NUDT22 | 11 | q13.1 | 4.68E-04 | 6.92E-03 | 0.49 | 1.04 | 0.72 |  |
| ATG2A | 11 | q13.1 | 4.69E-04 | 6.93E-03 | 0.49 | 1.01 | 0.79 |  |
| CAPN1 | 11 | q13.1 | 4.97E-04 | 7.25E-03 | 0.49 | 1.42 | 0.79 |  |
| C11orf2 | 11 | q13.1 | 1.32E-03 | 1.48E-02 | 0.45 | 1.08 | 0.79 |  |
| RPS6KA4 | 11 | q13.1 | 1.37E-03 | 1.53E-02 | 0.45 | 1.37 | 0.78 |  |
| SART1 | 11 | q13.1 | 1.91E-03 | 1.96E-02 | 0.44 | 1.09 | 0.95 |  |
| BAD | 11 | q13.1 | 2.34E-03 | 2.26E-02 | 0.43 | 1.01 | 0.82 |  |
| MAP3K11 | 11 | q13.1 | 2.57E-03 | 2.42E-02 | 0.43 | 1.19 | 0.80 |  |
| MACROD1 | 11 | q13.1 | 3.11E-03 | 2.78E-02 | 0.42 | 0.93 | 0.78 |  |
| BANF1 | 11 | q13.1 | 3.39E-03 | 2.94E-02 | 0.42 | 1.20 | 0.87 |  |
| ZNHIT2 | 11 | q13.1 | 3.45E-03 | 2.98E-02 | 0.42 | 1.16 | 0.80 |  |
| ARL2 | 11 | q13.1 | 4.43E-03 | 3.57E-02 | 0.41 | 1.36 | 0.68 |  |
| MARK2 | 11 | q13.1 | 4.59E-03 | 3.65E-02 | 0.41 | 1.19 | 0.90 |  |
| KDM2A | 11 | q13.2 | 4.84E-14 | 7.26E-11 | 0.85 | 2.07 | 0.67 | 3.72 |
| RCE1 | 11 | q13.2 | 6.17E-13 | 6.51E-10 | 0.83 | 1.89 | 0.74 | 2.96 |
| SSH3 | 11 | q13.2 | 1.20E-11 | 7.74E-09 | 0.80 | 2.24 | 0.60 | 4.82 |
| CABP4 | 11 | q13.2 | 1.69E-11 | 1.01E-08 | 0.80 | 2.58 | 0.77 | 6.31 |
| CHKA | 11 | q13.2 | 3.00E-11 | 1.56E-08 | 0.79 | 1.76 | 0.70 | 5.03 |
| CORO1B | 11 | q13.2 | 1.18E-10 | 4.98E-08 | 0.78 | 1.88 | 0.79 | 2.61 |
| YIF1A | 11 | q13.2 | 5.83E-10 | 1.75E-07 | 0.76 | 1.60 | 0.64 | 3.22 |
| NDUFS8 | 11 | q13.2 | 1.10E-09 | 2.87E-07 | 0.75 | 2.00 | 0.65 | 2.78 |
| CCS | 11 | q13.2 | 3.78E-09 | 7.85E-07 | 0.74 | 1.76 | 0.72 | 4.41 |
| RAD9A | 11 | q13.2 | 4.20E-09 | 8.66E-07 | 0.73 | 1.82 | 0.78 | 2.87 |
| POLD4 | 11 | q13.2 | 6.29E-09 | 1.24E-06 | 0.73 | 1.98 | 0.78 | 4.20 |
| KLC2 | 11 | q13.2 | 7.74E-09 | 1.45E-06 | 0.73 | 1.57 | 0.86 | 9.19 |
| NDUFV1 | 11 | q13.2 | 9.32E-09 | 1.66E-06 | 0.72 | 1.80 | 0.71 | 2.95 |
| RAB1B | 11 | q13.2 | 1.48E-08 | 2.39E-06 | 0.72 | 1.85 | 0.74 | 14.05 |
| BBS1 | 11 | q13.2 | 2.78E-08 | 3.93E-06 | 0.71 | 1.72 | 0.70 | 6.62 |
| C11orf80 | 11 | q13.2 | 3.61E-08 | 4.75E-06 | 0.70 | 2.37 | 0.74 | 5.85 |
| RBM4B | 11 | q13.2 | 5.43E-08 | 6.49E-06 | 0.70 | 1.61 | 0.80 | 2.37 |
| BRMS1 | 11 | q13.2 | 1.31E-07 | 1.33E-05 | 0.68 | 1.70 | 0.76 | 12.61 |
| TMEM134 | 11 | q13.2 | 2.11E-07 | 1.94E-05 | 0.67 | 1.94 | 0.73 | 2.44 |
| PPP1CA | 11 | q13.2 | 3.16E-07 | 2.68E-05 | 0.67 | 2.32 | 0.83 | 3.75 |
| RBM14 | 11 | q13.2 | 5.17E-07 | 3.88E-05 | 0.66 | 1.90 | 0.75 | 2.15 |
| SAPS3 | 11 | q13.2 | 8.36E-07 | 5.50E-05 | 0.65 | 1.58 | 0.69 | 1.99 |
| RPS6KB2 | 11 | q13.2 | 2.50E-06 | 1.25E-04 | 0.63 | 1.87 | 0.83 | 3.11 |
| AIP | 11 | q13.2 | 3.90E-06 | 1.79E-04 | 0.62 | 1.60 | 0.85 | 2.10 |
| PITPNM1 | 11 | q13.2 | 5.35E-06 | 2.27E-04 | 0.61 | 1.57 | 0.80 | 3.54 |
| CDK2AP2 | 11 | q13.2 | 5.65E-06 | 2.36E-04 | 0.61 | 1.66 | 0.67 | 2.49 |
| ATPGD1 | 11 | q13.2 | 1.20E-05 | 4.22E-04 | 0.59 | 1.21 | 0.94 | 1.51 |
| ZDHHC24 | 11 | q13.2 | 1.75E-05 | 5.53E-04 | 0.58 | 1.47 | 0.69 | 4.00 |
| SPTBN2 | 11 | q13.2 | 5.27E-05 | 1.31E-03 | 0.55 | 1.57 | 0.93 | 1.84 |
| C11orf24 | 11 | q13.2 | 5.68E-05 | 1.39E-03 | 0.55 | 1.63 | 0.73 | 2.05 |
| ANKRD13D | 11 | q13.2 | 6.18E-05 | 1.48E-03 | 0.55 | 1.52 | 0.65 | 2.57 |
| SYT12 | 11 | q13.2 | 7.59E-05 | 1.75E-03 | 0.54 | 1.56 | 0.79 | 2.77 |
| AP001107.5-1 | 11 | q13.2 | 8.85E-05 | 1.94E-03 | 0.54 | 1.39 | 0.78 | 5.00 |
| MRPL11 | 11 | q13.2 | 9.76E-05 | 2.10E-03 | 0.54 | 1.50 | 0.71 | 2.50 |
| CNIH2 | 11 | q13.2 | 1.77E-04 | 3.28E-03 | 0.52 | 1.42 | 0.75 | 2.13 |
| LRFN4 | 11 | q13.2 | 2.28E-04 | 3.99E-03 | 0.51 | 1.35 | 0.81 | 2.08 |
| AP003716.4-2 | 11 | q13.2 | 2.45E-04 | 4.23E-03 | 0.51 | 1.32 | 0.95 | 1.66 |
| SUV420H1 | 11 | q13.2 | 4.15E-04 | 6.34E-03 | 0.49 | 1.25 | 0.92 | 1.60 |
| PELI3 | 11 | q13.2 | 4.20E-04 | 6.39E-03 | 0.49 | 1.12 | 0.89 | 2.92 |
| TCIRG1 | 11 | q13.2 | 6.53E-04 | 8.87E-03 | 0.48 | 1.58 | 0.76 | 2.05 |
| ALDH3B1 | 11 | q13.2 | 3.23E-03 | 2.85E-02 | 0.42 | 1.30 | 0.93 | 2.13 |
| CTSF | 11 | q13.2 | 5.35E-03 | 4.09E-02 | 0.40 | 1.28 | 0.72 | 3.35 |
| ORAOV1 | 11 | q13.3 | 1.53E-13 | 1.82E-10 | 0.84 | 2.22 | 0.66 | 2.81 |
| FADD | 11 | q13.3 | 1.53E-13 | 1.82E-10 | 0.84 | 2.06 | 0.62 | 2.29 |
| MRPL21 | 11 | q13.3 | 1.62E-10 | 6.22E-08 | 0.78 | 1.94 | 0.66 | 2.42 |
| PPFIA1 | 11 | q13.3 | 1.27E-09 | 3.25E-07 | 0.75 | 1.73 | 0.61 | 1.93 |
| CTTN | 11 | q13.3 | 1.55E-08 | 2.50E-06 | 0.72 | 1.58 | 0.79 | 1.78 |
| IGHMBP2 | 11 | q13.3 | 2.27E-08 | 3.32E-06 | 0.71 | 1.31 | 0.83 | 1.52 |
| MTL5 | 11 | q13.3 | 8.60E-08 | 9.42E-06 | 0.69 | 1.65 | 0.81 | 2.29 |
| ANO1 | 11 | q13.3 | 1.28E-06 | 7.60E-05 | 0.64 | 1.99 | 0.71 | 2.23 |
| TPCN2 | 11 | q13.3 | 4.70E-06 | 2.04E-04 | 0.61 | 1.61 | 0.70 | 1.79 |
| CCND1 | 11 | q13.3 | 5.96E-05 | 1.44E-03 | 0.55 | 1.44 | 0.75 | 1.73 |
| FGF4 | 11 | q13.3 | 1.13E-03 | 1.32E-02 | 0.46 | 1.06 | 0.95 | 1.10 |
| CHCHD8 | 11 | q13.4 | 1.11E-13 | 1.57E-10 | 0.84 | 1.85 | 0.65 | 2.36 |
| PAAF1 | 11 | q13.4 | 6.80E-09 | 1.30E-06 | 0.73 | 1.49 | 0.68 | 2.02 |
| C11orf59 | 11 | q13.4 | 8.18E-09 | 1.51E-06 | 0.73 | 1.59 | 0.71 | 2.17 |
| DHCR7 | 11 | q13.4 | 1.99E-08 | 2.97E-06 | 0.71 | 2.26 | 0.59 | 3.27 |
| NADSYN1 | 11 | q13.4 | 6.69E-08 | 7.66E-06 | 0.69 | 1.86 | 0.63 | 2.96 |
| CHRDL2 | 11 | q13.4 | 1.33E-07 | 1.33E-05 | 0.68 | 1.18 | 0.90 | 1.96 |
| RAB6A | 11 | q13.4 | 1.37E-07 | 1.37E-05 | 0.68 | 1.49 | 0.70 | 2.12 |
| LRRC51 | 11 | q13.4 | 5.77E-07 | 4.18E-05 | 0.66 | 1.73 | 0.71 | 2.40 |
| C11orf51 | 11 | q13.4 | 6.84E-07 | 4.73E-05 | 0.65 | 1.39 | 0.77 | 2.27 |
| ATG16L2 | 11 | q13.4 | 1.33E-06 | 7.87E-05 | 0.64 | 1.57 | 0.66 | 2.35 |
| MRPL48 | 11 | q13.4 | 2.66E-06 | 1.31E-04 | 0.62 | 1.52 | 0.78 | 2.01 |
| INPPL1 | 11 | q13.4 | 4.56E-06 | 1.99E-04 | 0.61 | 1.84 | 0.62 | 1.96 |
| ARHGEF17 | 11 | q13.4 | 4.56E-06 | 1.99E-04 | 0.61 | 1.29 | 0.71 | 1.93 |
| UCP3 | 11 | q13.4 | 1.61E-05 | 5.23E-04 | 0.58 | 1.15 | 0.89 | 1.50 |
| POLD3 | 11 | q13.4 | 4.79E-05 | 1.22E-03 | 0.56 | 1.35 | 0.88 | 1.09 |
| XRRA1 | 11 | q13.4 | 5.56E-05 | 1.36E-03 | 0.55 | 1.10 | 0.95 | 1.46 |
| SPCS2 | 11 | q13.4 | 1.01E-04 | 2.14E-03 | 0.54 | 1.72 | 0.70 | 1.84 |
| RPS3P3 | 11 | q13.4 | 3.48E-04 | 5.52E-03 | 0.50 | 1.39 | 0.79 | 0.88 |
| C2CD3 | 11 | q13.4 | 1.11E-03 | 1.31E-02 | 0.46 | 1.09 | 0.91 | 1.60 |
| FAM86C | 11 | q13.4 | 1.22E-03 | 1.39E-02 | 0.46 | 1.31 | 0.94 | 1.46 |
| KLHL35 | 11 | q13.4 | 1.92E-03 | 1.97E-02 | 0.44 | 1.81 | 0.64 | 1.40 |
| RNF169 | 11 | q13.4 | 2.11E-03 | 2.11E-02 | 0.44 | 1.03 | 0.95 | 1.23 |
| PPME1 | 11 | q13.4 | 2.15E-03 | 2.13E-02 | 0.44 | 1.38 | 0.68 | 1.48 |
| P2RY2 | 11 | q13.4 | 3.50E-03 | 3.01E-02 | 0.42 | 1.08 | 0.93 | 1.14 |
| STARD10 | 11 | q13.4 | 5.70E-03 | 4.28E-02 | 0.40 | 1.96 | 0.75 | 1.37 |
| RNF121 | 11 | q13.4 | 6.36E-03 | 4.64E-02 | 0.39 | 1.21 | 0.84 | 0.76 |
| CAPN5 | 11 | q13.5 | 2.65E-04 | 4.47E-03 | 0.51 | 1.07 | 1.05 | 2.16 |
| ACER3 | 11 | q13.5 | 2.74E-04 | 4.59E-03 | 0.51 | 1.49 | 0.71 | 2.08 |
| PRKRIR | 11 | q13.5 | 2.99E-04 | 4.92E-03 | 0.50 | 1.36 | 0.69 | 1.62 |
| MYO7A | 11 | q13.5 | 3.34E-03 | 2.92E-02 | 0.42 | 1.48 | 0.78 | 2.31 |
| PAK1 | 11 | q13.5-q14.1 | 5.21E-07 | 3.90E-05 | 0.66 | 1.93 | 0.65 | 2.88 |
| INTS4 | 11 | q14.1 | 0.00E+00 | 0.00E+00 | 0.88 | 1.86 | 0.60 | 3.05 |
| RSF1 | 11 | q14.1 | 4.44E-16 | 2.11E-12 | 0.88 | 1.66 | 0.66 | 2.77 |
| C11orf67 | 11 | q14.1 | 3.55E-15 | 9.20E-12 | 0.87 | 2.41 | 0.54 | 5.43 |
| ALG8 | 11 | q14.1 | 1.07E-14 | 2.02E-11 | 0.86 | 1.98 | 0.62 | 2.26 |
| CLNS1A | 11 | q14.1 | 1.25E-10 | 5.14E-08 | 0.78 | 2.31 | 0.68 | 3.37 |
| CCDC90B | 11 | q14.1 | 1.30E-10 | 5.28E-08 | 0.78 | 1.20 | 0.90 | 2.53 |
| C11orf82 | 11 | q14.1 | 4.99E-10 | 1.58E-07 | 0.76 | 1.85 | 0.77 | 4.57 |
| KCTD21 | 11 | q14.1 | 1.56E-09 | 3.90E-07 | 0.75 | 1.35 | 0.79 | 1.69 |
| NARS2 | 11 | q14.1 | 1.73E-09 | 4.22E-07 | 0.75 | 1.53 | 0.75 | 1.68 |
| AQP11 | 11 | q14.1 | 2.56E-09 | 5.60E-07 | 0.74 | 1.51 | 0.82 | 3.13 |
| TMEM126A | 11 | q14.1 | 2.48E-07 | 2.20E-05 | 0.67 | 1.48 | 0.70 | 2.60 |
| USP35 | 11 | q14.1 | 3.14E-07 | 2.67E-05 | 0.67 | 1.30 | 0.72 | 1.65 |
| ANKRD42 | 11 | q14.1 | 4.01E-07 | 3.17E-05 | 0.66 | 1.22 | 0.90 | 1.83 |
| TMEM126B | 11 | q14.1 | 1.85E-06 | 1.02E-04 | 0.63 | 1.47 | 0.74 | 3.90 |
| CREBZF | 11 | q14.1 | 3.65E-06 | 1.70E-04 | 0.62 | 1.39 | 0.89 | 2.34 |
| NDUFC2 | 11 | q14.1 | 8.91E-06 | 3.38E-04 | 0.60 | 1.27 | 0.88 | 1.41 |
| GAB2 | 11 | q14.1 | 1.20E-04 | 2.46E-03 | 0.53 | 1.38 | 0.73 | 1.41 |
| PRCP | 11 | q14.1 | 2.68E-04 | 4.51E-03 | 0.51 | 1.30 | 0.73 | 1.50 |
| RAB30 | 11 | q14.1 | 3.97E-04 | 6.11E-03 | 0.50 | 1.51 | 0.86 | 2.74 |
| SYTL2 | 11 | q14.1 | 2.04E-03 | 2.06E-02 | 0.44 | 1.35 | 0.87 | 1.66 |
| ODZ4 | 11 | q14.1 | 6.50E-03 | 4.71E-02 | 0.39 | 1.05 | 0.96 | 1.13 |
| EED | 11 | q14.2 | 5.57E-07 | 4.07E-05 | 0.66 | 1.18 | 0.89 | 1.54 |
| C11orf73 | 11 | q14.2 | 5.90E-07 | 4.23E-05 | 0.65 | 1.50 | 0.80 | 2.69 |
| PICALM | 11 | q14.2 | 4.60E-05 | 1.18E-03 | 0.56 | 1.31 | 0.77 | 2.03 |
| TMEM135 | 11 | q14.2 | 8.16E-05 | 1.84E-03 | 0.54 | 1.32 | 0.67 | 1.84 |
| CCDC81 | 11 | q14.2 | 4.93E-04 | 7.22E-03 | 0.49 | 1.17 | 0.94 | 1.23 |
| CHORDC1 | 11 | q14.3 | 3.10E-03 | 2.77E-02 | 0.42 | 1.26 | 0.86 | 1.53 |
| CWC15 | 11 | q21 | 1.86E-12 | 1.61E-09 | 0.82 | 1.89 | 0.78 | 3.04 |
| KDM4D | 11 | q21 | 7.07E-11 | 3.14E-08 | 0.78 | 1.43 | 0.92 | 1.87 |
| ANKRD49 | 11 | q21 | 1.33E-07 | 1.33E-05 | 0.68 | 1.51 | 0.78 | 1.85 |
| SFRS2B | 11 | q21 | 2.23E-06 | 1.15E-04 | 0.63 | 1.70 | 0.74 | 2.20 |
| MTMR2 | 11 | q21 | 9.43E-06 | 3.52E-04 | 0.60 | 1.44 | 0.84 | 1.75 |
| FAM76B | 11 | q21 | 6.98E-05 | 1.63E-03 | 0.55 | 1.28 | 0.86 | 2.19 |
| PANX1 | 11 | q21 | 2.11E-04 | 3.77E-03 | 0.52 | 1.33 | 0.91 | 1.34 |
| MRE11A | 11 | q21 | 2.49E-04 | 4.28E-03 | 0.51 | 1.26 | 0.98 | 1.77 |
| GPR83 | 11 | q21 | 3.43E-04 | 5.45E-03 | 0.50 | 1.07 | 0.96 | 1.24 |
| SLC36A4 | 11 | q21 | 5.50E-04 | 7.82E-03 | 0.49 | 1.32 | 0.76 | 2.78 |
| SNORA25 | 11 | q21 | 1.61E-03 | 1.71E-02 | 0.45 | 1.41 | 0.79 | 1.12 |
| CEP57 | 11 | q21 | 4.52E-03 | 3.62E-02 | 0.41 | 1.10 | 0.90 | 1.81 |
| ENDOD1 | 11 | q21 | 4.89E-03 | 3.82E-02 | 0.40 | 1.26 | 0.72 | 1.59 |
| MED17 | 11 | q21 | 6.57E-03 | 4.75E-02 | 0.39 | 1.10 | 0.93 | 1.15 |
| TMEM133 | 11 | q22.1 | 9.33E-05 | 2.02E-03 | 0.54 | 1.17 | 0.98 | 1.79 |
| KIAA1377 | 11 | q22.1 | 2.98E-04 | 4.90E-03 | 0.50 | 1.09 | 0.96 | 1.24 |
| BIRC2 | 11 | q22.2 | 1.12E-03 | 1.32E-02 | 0.46 | 1.21 | 0.89 |  |
| CWF19L2 | 11 | q22.3 | 2.73E-06 | 1.33E-04 | 0.62 | 1.55 | 0.80 |  |
| DDX10 | 11 | q22.3 | 7.67E-04 | 9.96E-03 | 0.47 | 1.44 | 0.84 |  |
| ALKBH8 | 11 | q22.3 | 8.26E-04 | 1.05E-02 | 0.47 | 2.29 | 0.81 |  |
| DCUN1D5 | 11 | q22.3 | 8.44E-04 | 1.06E-02 | 0.47 | 2.04 | 0.79 |  |
| RDX | 11 | q22.3 | 9.25E-04 | 1.14E-02 | 0.47 | 1.49 | 0.74 |  |
| NPAT | 11 | q22.3 | 1.31E-03 | 1.47E-02 | 0.46 | 1.52 | 0.81 |  |
| SLC35F2 | 11 | q22.3 | 1.40E-03 | 1.54E-02 | 0.45 | 1.62 | 0.81 |  |
| CASP1 | 11 | q22.3 | 6.81E-03 | 4.88E-02 | 0.39 | 1.11 | 0.96 | 1.22 |
| TIMM8B | 11 | q23.1 | 3.91E-07 | 3.11E-05 | 0.66 | 2.47 | 0.71 |  |
| C11orf57 | 11 | q23.1 | 1.10E-04 | 2.30E-03 | 0.53 | 1.61 | 0.83 |  |
| FDXACB1 | 11 | q23.1 | 2.51E-03 | 2.38E-02 | 0.43 | 1.68 | 0.85 |  |
| SIK2 | 11 | q23.1 | 4.22E-03 | 3.45E-02 | 0.41 | 1.11 | 0.91 |  |
| C11orf71 | 11 | q23.2 | 1.70E-05 | 5.45E-04 | 0.58 | 1.98 | 0.75 |  |
| REXO2 | 11 | q23.2 | 1.86E-04 | 3.42E-03 | 0.52 | 1.38 | 0.69 |  |
| AP002373.3 | 11 | q23.2 | 1.63E-03 | 1.73E-02 | 0.45 | 1.29 | 0.71 |  |
| TECTA | 11 | q23.3 | 3.43E-07 | 2.84E-05 | 0.66 | 2.37 | 0.90 | 5.51 |
| HYOU1 | 11 | q23.3 | 7.34E-07 | 4.98E-05 | 0.65 | 2.91 | 0.63 |  |
| DPAGT1 | 11 | q23.3 | 1.91E-06 | 1.04E-04 | 0.63 | 1.69 | 0.78 |  |
| APOA1 | 11 | q23.3 | 7.49E-06 | 2.95E-04 | 0.60 | 2.30 | 0.80 |  |
| ZNF259 | 11 | q23.3 | 1.41E-05 | 4.78E-04 | 0.59 | 1.43 | 0.83 |  |
| PCSK7 | 11 | q23.3 | 4.51E-05 | 1.16E-03 | 0.56 | 2.26 | 0.77 |  |
| HMBS | 11 | q23.3 | 8.22E-05 | 1.85E-03 | 0.54 | 1.58 | 0.78 |  |
| SLC37A4 | 11 | q23.3 | 1.14E-04 | 2.35E-03 | 0.53 | 2.41 | 0.85 |  |
| ARCN1 | 11 | q23.3 | 2.32E-04 | 4.05E-03 | 0.51 | 2.18 | 0.75 |  |
| TRAPPC4 | 11 | q23.3 | 4.22E-04 | 6.40E-03 | 0.49 | 1.30 | 0.82 |  |
| ATP5L | 11 | q23.3 | 4.72E-04 | 6.98E-03 | 0.49 | 2.37 | 0.78 |  |
| HINFP | 11 | q23.3 | 4.73E-04 | 6.98E-03 | 0.49 | 1.97 | 0.81 |  |
| RNF214 | 11 | q23.3 | 4.77E-04 | 7.03E-03 | 0.49 | 1.21 | 0.91 |  |
| AP006216.9 | 11 | q23.3 | 6.17E-04 | 8.50E-03 | 0.48 | 2.16 | 0.61 |  |
| H2AFX | 11 | q23.3 | 1.33E-03 | 1.49E-02 | 0.45 | 3.21 | 0.86 |  |
| VPS11 | 11 | q23.3 | 2.35E-03 | 2.27E-02 | 0.43 | 1.15 | 0.89 |  |
| RPS25 | 11 | q23.3 | 2.42E-03 | 2.33E-02 | 0.43 | 1.49 | 0.85 |  |
| CBL | 11 | q23.3 | 2.75E-03 | 2.55E-02 | 0.43 | 1.75 | 0.88 |  |
| C11orf60 | 11 | q23.3 | 2.88E-03 | 2.63E-02 | 0.43 | 1.64 | 0.81 |  |
| MLL | 11 | q23.3 | 3.46E-03 | 2.99E-02 | 0.42 | 1.56 | 0.84 |  |
| RNF26 | 11 | q23.3 | 4.40E-03 | 3.55E-02 | 0.41 | 2.50 | 0.82 |  |
| UBE4A | 11 | q23.3 | 4.58E-03 | 3.65E-02 | 0.41 | 1.70 | 0.71 |  |
| CEP164 | 11 | q23.3 | 4.91E-03 | 3.83E-02 | 0.40 | 1.71 | 0.80 |  |
| POU2F3 | 11 | q23.3 | 5.01E-03 | 3.89E-02 | 0.40 | 1.16 | 0.91 |  |
| GRIK4 | 11 | q23.3 | 5.69E-03 | 4.28E-02 | 0.40 | 1.22 | 0.96 | 1.48 |
| TBCEL | 11 | q23.3 | 6.10E-03 | 4.50E-02 | 0.39 | 1.21 | 0.97 | 1.31 |
| BUD13 | 11 | q23.3 | 6.41E-03 | 4.66E-02 | 0.39 | 1.88 | 0.79 |  |
| HSPA8 | 11 | q24.1 | 2.43E-06 | 1.23E-04 | 0.63 | 1.34 | 0.92 | 1.82 |
| C11orf63 | 11 | q24.1 | 5.07E-04 | 7.37E-03 | 0.49 | 1.75 | 0.82 | 4.34 |
| ZNF202 | 11 | q24.1 | 1.79E-03 | 1.87E-02 | 0.44 | 1.38 | 0.81 | 1.03 |
| STT3A | 11 | q24.2 | 1.29E-06 | 7.65E-05 | 0.64 | 2.34 | 0.69 |  |
| PUS3 | 11 | q24.2 | 1.93E-06 | 1.05E-04 | 0.63 | 1.43 | 0.71 |  |
| EI24 | 11 | q24.2 | 4.37E-06 | 1.93E-04 | 0.61 | 1.92 | 0.79 |  |
| SRPR | 11 | q24.2 | 5.36E-06 | 2.27E-04 | 0.61 | 2.46 | 0.67 |  |
| FOXRED1 | 11 | q24.2 | 5.54E-06 | 2.33E-04 | 0.61 | 2.44 | 0.82 |  |
| RPUSD4 | 11 | q24.2 | 1.68E-05 | 5.42E-04 | 0.58 | 1.67 | 0.67 |  |
| DCPS | 11 | q24.2 | 3.01E-05 | 8.56E-04 | 0.57 | 2.03 | 0.80 |  |
| TIRAP | 11 | q24.2 | 4.51E-04 | 6.75E-03 | 0.49 | 1.32 | 0.85 |  |
| SIAE | 11 | q24.2 | 1.36E-03 | 1.52E-02 | 0.45 | 1.39 | 0.66 |  |
| OR8D2 | 11 | q24.2 | 6.68E-03 | 4.80E-02 | 0.39 | 1.09 | 0.97 | 1.18 |
| SNX19 | 11 | q24.3 | 3.63E-05 | 9.84E-04 | 0.56 | 2.19 | 0.76 |  |
| ST14 | 11 | q24.3 | 7.76E-05 | 1.78E-03 | 0.54 | 2.18 | 0.76 |  |
| NFRKB | 11 | q24.3 | 1.38E-03 | 1.53E-02 | 0.45 | 1.32 | 0.89 |  |
| VPS26B | 11 | q25 | 1.30E-05 | 4.49E-04 | 0.59 | 1.44 | 0.71 |  |
| THYN1 | 11 | q25 | 3.29E-05 | 9.16E-04 | 0.57 | 1.25 | 0.67 |  |
| NCAPD3 | 11 | q25 | 4.53E-03 | 3.62E-02 | 0.41 | 1.96 | 0.83 |  |
| B3GAT1 | 11 | q25 | 5.19E-03 | 4.01E-02 | 0.40 | 1.21 | 0.94 |  |
| YARS2 | 12 | p11.21 | 4.96E-04 | 7.24E-03 | 0.49 | 1.32 | 0.99 |  |
| C12orf35 | 12 | p11.21 | 6.94E-03 | 4.93E-02 | 0.39 | 1.97 | 0.98 |  |
| MRPS35 | 12 | p11.22 | 9.06E-06 | 3.42E-04 | 0.60 | 1.29 | 0.66 |  |
| MED21 | 12 | p11.23 | 2.29E-07 | 2.06E-05 | 0.67 | 1.27 | 0.85 | 2.06 |
| FGFR1OP2 | 12 | p11.23 | 7.97E-07 | 5.32E-05 | 0.65 | 1.17 | 0.91 | 1.99 |
| C12orf11 | 12 | p11.23 | 4.19E-05 | 1.10E-03 | 0.56 | 1.22 | 0.84 | 1.70 |
| TM7SF3 | 12 | p11.23 | 2.27E-03 | 2.21E-02 | 0.43 | 1.16 | 0.66 | 2.17 |
| STK38L | 12 | p11.23 | 4.64E-03 | 3.68E-02 | 0.41 | 1.25 | 0.93 | 1.16 |
| KRAS | 12 | p12.1 | 1.13E-08 | 1.96E-06 | 0.72 | 1.26 | 0.76 | 3.13 |
| KIAA0528 | 12 | p12.1 | 5.28E-06 | 2.24E-04 | 0.61 | 1.39 | 0.61 | 3.15 |
| LYRM5 | 12 | p12.1 | 1.52E-04 | 2.92E-03 | 0.52 | 1.28 | 0.69 | 3.39 |
| RECQL | 12 | p12.1 | 1.67E-04 | 3.14E-03 | 0.52 | 1.41 | 0.83 | 2.51 |
| ETNK1 | 12 | p12.1 | 5.58E-04 | 7.92E-03 | 0.48 | 1.35 | 0.72 | 2.97 |
| GOLT1B | 12 | p12.1 | 6.61E-04 | 8.92E-03 | 0.48 | 1.18 | 0.82 | 4.78 |
| CMAS | 12 | p12.1 | 5.90E-03 | 4.39E-02 | 0.40 | 1.06 | 0.83 | 2.63 |
| AEBP2 | 12 | p12.3 | 1.21E-05 | 4.25E-04 | 0.59 | 1.21 | 0.86 | 2.86 |
| PLEKHA5 | 12 | p12.3 | 9.29E-04 | 1.14E-02 | 0.47 | 1.62 | 0.66 | 1.18 |
| DERA | 12 | p12.3 | 6.57E-03 | 4.75E-02 | 0.39 | 1.15 | 0.81 |  |
| DDX47 | 12 | p13.1 | 3.11E-03 | 2.78E-02 | 0.42 | 1.36 | 0.82 |  |
| PEX5 | 12 | p13.31 | 5.90E-04 | 8.23E-03 | 0.48 |  | 0.71 |  |
| NECAP1 | 12 | p13.31 | 8.37E-04 | 1.06E-02 | 0.47 |  | 0.74 |  |
| IFFO1 | 12 | p13.31 | 9.09E-04 | 1.12E-02 | 0.47 | 1.32 | 0.80 |  |
| NCAPD2 | 12 | p13.31 | 2.44E-03 | 2.34E-02 | 0.43 | 0.83 | 0.68 |  |
| DDX12 | 12 | p13.31 | 2.94E-03 | 2.67E-02 | -0.42 | 0.94 | 1.04 |  |
| USP5 | 12 | p13.31 | 3.29E-03 | 2.89E-02 | 0.42 | 1.09 | 0.81 |  |
| EMG1 | 12 | p13.31 | 3.54E-03 | 3.04E-02 | 0.42 | 1.25 | 0.73 |  |
| ATN1 | 12 | p13.31 | 3.99E-03 | 3.31E-02 | 0.41 | 0.82 | 0.74 |  |
| PHC1 | 12 | p13.31 | 3.99E-03 | 3.31E-02 | 0.41 | 1.08 | 0.74 |  |
| MRPL51 | 12 | p13.31 | 4.09E-03 | 3.37E-02 | 0.41 | 1.68 | 0.83 |  |
| FOXJ2 | 12 | p13.31 | 4.13E-03 | 3.40E-02 | 0.41 |  | 0.78 |  |
| NDUFA9 | 12 | p13.32 | 2.09E-04 | 3.75E-03 | 0.52 | 1.11 | 0.76 |  |
| DYRK4 | 12 | p13.32 | 4.69E-03 | 3.71E-02 | 0.41 | 1.35 | 0.80 |  |
| C12orf32 | 12 | p13.33 | 3.23E-04 | 5.21E-03 | 0.50 | 1.02 | 0.80 |  |
| KDM5A | 12 | p13.33 | 1.14E-03 | 1.33E-02 | 0.46 | 1.23 | 0.78 |  |
| WNK1 | 12 | p13.33 | 3.66E-03 | 3.12E-02 | 0.42 | 1.45 | 0.83 |  |
| TWF1 | 12 | q12 | 5.14E-04 | 7.44E-03 | 0.49 | 1.14 | 0.91 |  |
| SLC38A1 | 12 | q13.11 | 4.09E-04 | 6.26E-03 | 0.49 | 1.63 | 0.37 |  |
| C12orf41 | 12 | q13.11 | 1.21E-03 | 1.38E-02 | 0.46 | 1.49 | 0.79 |  |
| SLC38A2 | 12 | q13.11 | 1.64E-03 | 1.74E-02 | 0.45 | 1.60 | 0.68 |  |
| TMEM106C | 12 | q13.11 | 2.38E-03 | 2.30E-02 | 0.43 | 1.62 | 0.85 |  |
| SLC11A2 | 12 | q13.12 | 8.18E-04 | 1.04E-02 | 0.47 | 1.77 | 0.76 |  |
| LIMA1 | 12 | q13.12 | 2.78E-03 | 2.56E-02 | 0.43 | 1.18 | 0.69 |  |
| CSRNP2 | 12 | q13.12 | 3.01E-03 | 2.72E-02 | 0.42 | 1.84 | 0.79 |  |
| TMBIM6 | 12 | q13.12 | 4.30E-03 | 3.49E-02 | 0.41 | 1.57 | 0.73 |  |
| ATF1 | 12 | q13.12 | 4.91E-03 | 3.83E-02 | 0.40 | 1.48 | 0.92 |  |
| KRT8P9 | 12 | q13.13 | 1.36E-04 | 2.70E-03 | 0.53 | 2.66 | 0.68 |  |
| TARBP2 | 12 | q13.13 | 6.23E-04 | 8.56E-03 | 0.48 | 1.45 | 0.79 |  |
| ACVR1B | 12 | q13.13 | 1.12E-03 | 1.32E-02 | 0.46 | 1.42 | 0.79 |  |
| ATP5G2 | 12 | q13.13 | 1.76E-03 | 1.84E-02 | 0.44 | 1.36 | 0.79 |  |
| MFSD5 | 12 | q13.13 | 4.42E-03 | 3.57E-02 | 0.41 | 1.65 | 0.86 |  |
| FAM62A | 12 | q13.2 | 3.15E-05 | 8.88E-04 | 0.57 | 1.23 | 0.78 |  |
| SARNP | 12 | q13.2 | 4.45E-05 | 1.15E-03 | 0.56 | 1.60 | 0.88 |  |
| ORMDL2 | 12 | q13.2 | 5.38E-05 | 1.33E-03 | 0.55 | 1.50 | 0.83 |  |
| RPL41 | 12 | q13.2 | 1.72E-04 | 3.21E-03 | 0.52 | 1.51 | 0.92 |  |
| CDK2 | 12 | q13.2 | 5.03E-04 | 7.32E-03 | 0.49 | 1.72 | 0.82 |  |
| MYL6 | 12 | q13.2 | 1.38E-03 | 1.53E-02 | 0.45 | 1.13 | 0.81 |  |
| RAB5B | 12 | q13.2 | 3.27E-03 | 2.87E-02 | 0.42 | 1.37 | 0.72 |  |
| BLOC1S1 | 12 | q13.2 | 3.59E-03 | 3.07E-02 | 0.42 | 1.42 | 0.82 |  |
| WIBG | 12 | q13.2 | 5.26E-03 | 4.06E-02 | 0.40 | 1.57 | 0.83 |  |
| DCTN2 | 12 | q13.3 | 9.84E-08 | 1.06E-05 | 0.69 | 1.45 | 0.82 |  |
| CNPY2 | 12 | q13.3 | 2.57E-05 | 7.52E-04 | 0.57 | 1.63 | 0.74 |  |
| OBFC2B | 12 | q13.3 | 4.26E-05 | 1.12E-03 | 0.56 | 1.79 | 0.76 |  |
| R3HDM2 | 12 | q13.3 | 5.95E-04 | 8.29E-03 | 0.48 | 1.29 | 0.85 |  |
| PIP4K2C | 12 | q13.3 | 1.02E-03 | 1.22E-02 | 0.46 | 1.49 | 0.77 |  |
| ZBTB39 | 12 | q13.3 | 1.06E-03 | 1.26E-02 | 0.46 | 1.25 | 0.92 |  |
| RDH16 | 12 | q13.3 | 1.39E-03 | 1.53E-02 | 0.45 | 2.84 | 0.67 |  |
| SPRYD4 | 12 | q13.3 | 1.57E-03 | 1.68E-02 | 0.45 | 1.45 | 0.82 |  |
| GEFT | 12 | q13.3 | 3.26E-03 | 2.87E-02 | 0.42 | 1.06 | 1.01 |  |
| SDR9C7 | 12 | q13.3 | 5.22E-03 | 4.03E-02 | 0.40 | 1.30 | 0.98 |  |
| TSFM | 12 | q14.1 | 3.45E-04 | 5.48E-03 | 0.50 | 1.48 | 0.87 |  |
| CDK4 | 12 | q14.1 | 4.58E-04 | 6.83E-03 | 0.49 | 1.60 | 0.92 |  |
| TSPAN31 | 12 | q14.1 | 1.46E-03 | 1.59E-02 | 0.45 | 1.40 | 0.65 |  |
| METTL1 | 12 | q14.1 | 3.77E-03 | 3.19E-02 | 0.41 | 1.42 | 0.85 |  |
| TBK1 | 12 | q14.2 | 1.96E-03 | 2.00E-02 | 0.44 | 1.17 |  | 1.21 |
| CNOT2 | 12 | q15 | 1.83E-03 | 1.90E-02 | 0.44 | 1.17 | 0.74 | 1.36 |
| AC025257.22 | 12 | q21.1 | 8.59E-06 | 3.29E-04 | 0.60 | 1.44 | 0.52 |  |
| ZDHHC17 | 12 | q21.2 | 5.73E-04 | 8.08E-03 | 0.48 | 1.12 | 0.63 |  |
| NAP1L1 | 12 | q21.2 | 1.03E-03 | 1.23E-02 | 0.46 | 1.21 | 0.61 |  |
| PHLDA1 | 12 | q21.2 | 2.93E-03 | 2.66E-02 | 0.42 | 1.31 | 0.75 |  |
| BBS10 | 12 | q21.2 | 6.38E-03 | 4.65E-02 | 0.39 | 1.08 | 0.87 |  |
| CCDC59 | 12 | q21.31 | 5.21E-04 | 7.51E-03 | 0.49 | 1.36 | 0.53 | 1.77 |
| METAP2 | 12 | q22 | 1.48E-05 | 4.93E-04 | 0.59 | 1.34 | 0.78 |  |
| UBE2N | 12 | q22 | 1.48E-04 | 2.87E-03 | 0.53 | 1.70 | 0.65 |  |
| NR2C1 | 12 | q22 | 3.83E-03 | 3.22E-02 | 0.41 | 1.26 | 0.93 |  |
| CCDC41 | 12 | q22 | 4.53E-03 | 3.62E-02 | 0.41 | 1.21 | 0.75 |  |
| ACTR6 | 12 | q23.1 | 4.72E-04 | 6.98E-03 | 0.49 | 1.27 | 0.64 |  |
| SNRPF | 12 | q23.1 | 6.38E-04 | 8.73E-03 | 0.48 | 1.39 | 0.91 |  |
| SLC25A3 | 12 | q23.1 | 6.93E-04 | 9.20E-03 | 0.48 | 1.25 | 0.83 |  |
| ANKS1B | 12 | q23.1 | 2.50E-03 | 2.38E-02 | 0.43 | 1.32 | 0.68 |  |
| NEDD1 | 12 | q23.1 | 3.61E-03 | 3.08E-02 | 0.42 | 1.06 | 0.97 |  |
| SCYL2 | 12 | q23.1 | 5.34E-03 | 4.09E-02 | 0.40 | 1.23 | 0.72 |  |
| LTA4H | 12 | q23.1 | 5.67E-03 | 4.27E-02 | 0.40 | 1.24 | 0.83 |  |
| CCDC53 | 12 | q23.2 | 3.42E-04 | 5.45E-03 | 0.50 | 1.21 | 0.73 |  |
| ARL1 | 12 | q23.2 | 1.27E-03 | 1.44E-02 | 0.46 | 1.41 | 0.73 |  |
| NUP37 | 12 | q23.2 | 3.34E-03 | 2.92E-02 | 0.42 | 1.14 | 0.82 |  |
| PRDM4 | 12 | q23.3 | 3.56E-05 | 9.72E-04 | 0.56 | 1.43 | 0.79 |  |
| TDG | 12 | q23.3 | 4.45E-05 | 1.15E-03 | 0.56 | 1.29 | 0.74 |  |
| RIC8B | 12 | q23.3 | 5.65E-04 | 8.00E-03 | 0.48 | 1.10 | 0.79 |  |
| NT5DC3 | 12 | q23.3 | 9.05E-04 | 1.12E-02 | 0.47 | 1.22 | 0.78 |  |
| ISCU | 12 | q23.3 | 9.34E-04 | 1.14E-02 | 0.47 | 1.11 | 0.84 |  |
| MTERFD3 | 12 | q23.3 | 5.76E-03 | 4.32E-02 | 0.40 | 1.11 | 0.98 |  |
| PWP1 | 12 | q23.3 | 6.10E-03 | 4.50E-02 | 0.39 | 1.14 | 0.81 |  |
| ARPC3 | 12 | q24.11 | 7.09E-05 | 1.65E-03 | 0.55 | 1.29 | 0.77 |  |
| TCTN1 | 12 | q24.11 | 1.18E-04 | 2.43E-03 | 0.53 | 1.40 | 0.56 |  |
| TCHP | 12 | q24.11 | 8.10E-04 | 1.03E-02 | 0.47 | 1.58 | 0.81 |  |
| ATP2A2 | 12 | q24.11 | 1.40E-03 | 1.54E-02 | 0.45 | 1.36 | 0.80 |  |
| ANKRD13A | 12 | q24.11 | 2.10E-03 | 2.10E-02 | 0.44 | 1.39 | 0.89 |  |
| UBE3B | 12 | q24.11 | 2.14E-03 | 2.12E-02 | 0.44 | 1.27 | 0.75 |  |
| UNG | 12 | q24.11 | 3.27E-03 | 2.87E-02 | 0.42 | 1.74 | 0.73 |  |
| ATXN2 | 12 | q24.12 | 1.28E-05 | 4.44E-04 | 0.59 |  | 0.70 |  |
| AC003029.1 | 12 | q24.12 | 1.23E-04 | 2.49E-03 | 0.53 | 1.44 | 0.83 |  |
| C12orf52 | 12 | q24.13 | 6.71E-04 | 9.00E-03 | 0.48 | 1.73 | 0.81 |  |
| TRAFD1 | 12 | q24.13 | 4.20E-03 | 3.43E-02 | 0.41 | 1.14 | 0.86 |  |
| C12orf30 | 12 | q24.13 | 4.30E-03 | 3.49E-02 | 0.41 | 1.15 | 0.87 |  |
| TMEM116 | 12 | q24.13 | 6.87E-03 | 4.91E-02 | 0.39 | 2.19 | 0.71 |  |
| FBXO21 | 12 | q24.22 | 5.90E-03 | 4.39E-02 | 0.40 | 1.51 | 0.80 |  |
| RAB35 | 12 | q24.23 | 4.12E-03 | 3.39E-02 | 0.41 | 1.20 | 0.91 |  |
| PEBP1 | 12 | q24.23 | 5.56E-03 | 4.21E-02 | 0.40 | 1.69 | 0.75 |  |
| RNF34 | 12 | q24.31 | 7.42E-05 | 1.72E-03 | 0.55 | 1.85 | 0.87 |  |
| BRI3BP | 12 | q24.31 | 1.78E-04 | 3.29E-03 | 0.52 | 1.27 | 0.89 |  |
| DIABLO | 12 | q24.31 | 1.10E-03 | 1.29E-02 | 0.46 | 1.51 | 0.89 |  |
| C12orf65 | 12 | q24.31 | 1.18E-03 | 1.37E-02 | 0.46 | 1.33 | 0.84 |  |
| ANAPC5 | 12 | q24.31 | 2.04E-03 | 2.05E-02 | 0.44 | 1.58 | 0.86 |  |
| SETD1B | 12 | q24.31 | 2.94E-03 | 2.67E-02 | 0.42 | 1.35 | 0.95 |  |
| C12orf43 | 12 | q24.31 | 3.17E-03 | 2.82E-02 | 0.42 | 1.55 | 0.91 |  |
| AACS | 12 | q24.31 | 3.86E-03 | 3.24E-02 | 0.41 | 1.45 | 0.81 |  |
| EIF2B1 | 12 | q24.31 | 5.39E-03 | 4.12E-02 | 0.40 | 1.51 | 0.80 |  |
| SETD8 | 12 | q24.31 | 6.26E-03 | 4.59E-02 | 0.39 | 1.54 | 0.90 |  |
| SCARB1 | 12 | q24.31 | 6.55E-03 | 4.74E-02 | 0.39 | 1.68 | 0.86 |  |
| EP400 | 12 | q24.33 | 2.66E-03 | 2.48E-02 | 0.43 | 1.28 | 0.84 |  |
| PGAM5 | 12 | q24.33 | 4.57E-03 | 3.65E-02 | 0.41 | 1.13 | 0.82 |  |
| GOLGA3 | 12 | q24.33 | 6.13E-03 | 4.51E-02 | 0.39 | 1.26 | 0.82 |  |
| EFHA1 | 13 | q12.11 | 8.84E-05 | 1.94E-03 | 0.54 | 1.33 | 0.66 |  |
| ZMYM2 | 13 | q12.11 | 2.77E-04 | 4.62E-03 | 0.51 | 1.66 | 0.72 |  |
| ZMYM5 | 13 | q12.11 | 1.34E-03 | 1.50E-02 | 0.45 | 1.11 | 0.90 |  |
| XPO4 | 13 | q12.11 | 2.65E-03 | 2.48E-02 | 0.43 | 1.32 | 0.79 |  |
| N6AMT2 | 13 | q12.11 | 4.62E-03 | 3.68E-02 | 0.41 | 1.19 | 0.82 |  |
| IFT88 | 13 | q12.11 | 5.37E-03 | 4.11E-02 | 0.40 | 1.07 | 0.87 |  |
| MIPEP | 13 | q12.12 | 2.35E-03 | 2.27E-02 | 0.43 | 2.17 | 0.81 |  |
| SPATA13 | 13 | q12.12 | 4.13E-03 | 3.39E-02 | 0.41 | 0.90 | 0.82 |  |
| CENPJ | 13 | q12.12 | 6.94E-03 | 4.93E-02 | 0.39 | 1.22 | 0.86 |  |
| POLR1D | 13 | q12.2 | 1.33E-03 | 1.49E-02 | 0.45 | 1.40 | 0.77 |  |
| GTF3A | 13 | q12.2 | 4.32E-03 | 3.50E-02 | 0.41 | 1.26 | 0.62 |  |
| KATNAL1 | 13 | q12.3 | 1.44E-07 | 1.43E-05 | 0.68 | 1.45 | 0.83 | 2.63 |
| POMP | 13 | q12.3 | 3.42E-07 | 2.84E-05 | 0.67 | 1.77 | 0.70 | 2.41 |
| USPL1 | 13 | q12.3 | 1.69E-05 | 5.44E-04 | 0.58 | 1.58 | 0.67 | 1.76 |
| HSPH1 | 13 | q12.3 | 1.30E-04 | 2.62E-03 | 0.53 | 2.07 | 0.60 | 2.27 |
| SLC7A1 | 13 | q12.3 | 1.34E-04 | 2.68E-03 | 0.53 | 2.12 | 0.63 | 2.74 |
| UBL3 | 13 | q12.3 | 5.68E-04 | 8.04E-03 | 0.48 | 1.37 | 0.79 | 2.09 |
| PDS5B | 13 | q13.1 | 3.95E-05 | 1.05E-03 | 0.56 | 1.38 | 0.72 |  |
| EXOSC8 | 13 | q13.3 | 9.29E-08 | 1.01E-05 | 0.69 | 1.26 | 0.68 |  |
| NHLRC3 | 13 | q13.3 | 6.50E-06 | 2.62E-04 | 0.61 | 1.31 | 0.89 |  |
| C13orf23 | 13 | q13.3 | 5.09E-05 | 1.28E-03 | 0.56 | 2.05 | 0.75 |  |
| ALG5 | 13 | q13.3 | 2.57E-04 | 4.37E-03 | 0.51 | 1.35 | 0.71 |  |
| NBEA | 13 | q13.3 | 3.07E-03 | 2.75E-02 | 0.42 | 2.04 | 0.80 |  |
| SMAD9 | 13 | q13.3 | 3.37E-03 | 2.93E-02 | 0.42 | 1.20 | 0.94 |  |
| MRPS31 | 13 | q14.11 | 5.64E-07 | 4.11E-05 | 0.66 | 1.13 | 0.72 |  |
| COG6 | 13 | q14.11 | 4.95E-05 | 1.25E-03 | 0.56 | 1.23 | 0.74 |  |
| WBP4 | 13 | q14.11 | 8.63E-04 | 1.08E-02 | 0.47 | 0.95 | 0.77 |  |
| AKAP11 | 13 | q14.11 | 1.71E-03 | 1.80E-02 | 0.45 | 1.23 | 0.92 |  |
| ELF1 | 13 | q14.11 | 5.35E-03 | 4.09E-02 | 0.40 | 1.28 | 0.74 |  |
| GTF2F2 | 13 | q14.12-q14.13 | 1.69E-03 | 1.79E-02 | 0.45 | 1.00 | 0.67 |  |
| COG3 | 13 | q14.13 | 1.61E-06 | 9.21E-05 | 0.64 | 1.45 | 0.78 |  |
| KPNA3 | 13 | q14.2 | 2.11E-04 | 3.77E-03 | 0.52 | 1.31 | 0.75 |  |
| PHF11 | 13 | q14.2 | 9.29E-04 | 1.14E-02 | 0.47 | 0.92 | 0.79 |  |
| TRIM13 | 13 | q14.2 | 9.30E-04 | 1.14E-02 | 0.47 | 1.40 | 0.80 |  |
| ESD | 13 | q14.2 | 1.89E-03 | 1.95E-02 | 0.44 | 1.00 | 0.68 |  |
| MED4 | 13 | q14.2 | 2.86E-03 | 2.62E-02 | 0.43 | 1.39 | 0.81 |  |
| SETDB2 | 13 | q14.2 | 3.22E-03 | 2.85E-02 | 0.42 | 1.08 | 0.82 |  |
| SUGT1 | 13 | q14.3 | 2.67E-08 | 3.81E-06 | 0.71 | 1.58 | 0.68 |  |
| UTP14C | 13 | q14.3 | 6.80E-05 | 1.59E-03 | 0.55 | 1.09 | 0.86 |  |
| NEK3 | 13 | q14.3 | 2.33E-04 | 4.06E-03 | 0.51 | 1.33 | 0.81 |  |
| INTS6 | 13 | q14.3 | 2.96E-04 | 4.87E-03 | 0.50 | 1.11 | 0.71 |  |
| VPS36 | 13 | q14.3 | 1.30E-03 | 1.47E-02 | 0.46 | 1.19 | 0.69 |  |
| DIAPH3 | 13 | q21.2 | 6.03E-03 | 4.46E-02 | 0.39 | 1.03 | 0.87 |  |
| C13orf34 | 13 | q22.1 | 4.27E-05 | 1.12E-03 | 0.56 | 1.19 | 0.75 |  |
| PIBF1 | 13 | q22.1 | 2.24E-03 | 2.19E-02 | 0.44 | 1.10 | 0.86 |  |
| UCHL3 | 13 | q22.2 | 1.48E-05 | 4.93E-04 | 0.59 | 1.49 | 0.67 |  |
| COMMD6 | 13 | q22.2 | 1.48E-05 | 4.94E-04 | 0.59 | 1.29 | 0.61 |  |
| CLN5 | 13 | q22.3 | 1.33E-04 | 2.65E-03 | 0.53 | 1.28 | 0.74 |  |
| FBXL3 | 13 | q22.3 | 2.93E-03 | 2.66E-02 | 0.42 | 1.05 | 0.74 |  |
| NDFIP2 | 13 | q31.1 | 1.25E-03 | 1.42E-02 | 0.46 | 1.22 | 0.69 |  |
| RBM26 | 13 | q31.1 | 6.44E-03 | 4.68E-02 | 0.39 | 1.10 | 0.82 |  |
| UGCGL2 | 13 | q32.1 | 2.04E-05 | 6.24E-04 | 0.58 | 1.23 | 0.72 |  |
| GPR180 | 13 | q32.1 | 3.58E-05 | 9.75E-04 | 0.56 | 1.20 | 0.76 |  |
| RAP2A | 13 | q32.1 | 4.48E-04 | 6.71E-03 | 0.49 | 1.41 | 0.75 |  |
| TGDS | 13 | q32.1 | 3.07E-03 | 2.75E-02 | 0.42 | 1.17 | 0.83 |  |
| STK24 | 13 | q32.2 | 3.10E-05 | 8.76E-04 | 0.57 | 1.34 | 0.72 |  |
| FARP1 | 13 | q32.2 | 1.47E-04 | 2.85E-03 | 0.53 | 1.72 | 0.70 |  |
| IPO5 | 13 | q32.2 | 5.88E-04 | 8.23E-03 | 0.48 | 1.39 | 0.81 |  |
| PHGDHL1 | 13 | q32.3 | 1.19E-06 | 7.22E-05 | 0.64 | 1.36 | 0.72 |  |
| TM9SF2 | 13 | q32.3 | 2.26E-04 | 3.97E-03 | 0.51 | 1.34 | 0.64 |  |
| PCCA | 13 | q32.3 | 1.95E-03 | 1.99E-02 | 0.44 | 1.14 | 0.86 |  |
| ERCC5 | 13 | q33.1 | 2.28E-03 | 2.22E-02 | 0.43 | 1.19 | 0.76 |  |
| ARGLU1 | 13 | q33.3 | 1.49E-04 | 2.87E-03 | 0.53 | 1.29 | 0.54 |  |
| PCID2 | 13 | q34 | 4.73E-07 | 3.60E-05 | 0.66 | 1.56 | 0.73 |  |
| CARKD | 13 | q34 | 5.21E-07 | 3.90E-05 | 0.66 | 1.80 | 0.73 |  |
| ARHGEF7 | 13 | q34 | 1.77E-06 | 9.88E-05 | 0.63 | 1.27 | 0.78 |  |
| CUL4A | 13 | q34 | 1.29E-05 | 4.46E-04 | 0.59 | 1.65 | 0.67 |  |
| TFDP1 | 13 | q34 | 7.65E-05 | 1.76E-03 | 0.54 | 1.56 | 0.72 |  |
| LAMP1 | 13 | q34 | 9.22E-05 | 2.01E-03 | 0.54 | 1.29 | 0.74 |  |
| CDC16 | 13 | q34 | 9.25E-05 | 2.01E-03 | 0.54 | 1.21 | 0.69 |  |
| C13orf8 | 13 | q34 | 1.57E-04 | 2.99E-03 | 0.52 | 1.35 | 0.71 |  |
| ING1 | 13 | q34 | 2.35E-04 | 4.09E-03 | 0.51 | 1.55 | 0.66 |  |
| TMCO3 | 13 | q34 | 1.69E-03 | 1.78E-02 | 0.45 | 1.43 | 0.53 |  |
| DCUN1D2 | 13 | q34 | 2.14E-03 | 2.12E-02 | 0.44 | 1.14 | 0.88 |  |
| CARS2 | 13 | q34 | 6.91E-03 | 4.92E-02 | 0.39 | 1.10 | 0.91 |  |
| OSGEP | 14 | q11.2 | 7.00E-04 | 9.26E-03 | 0.48 | 1.17 | 0.80 |  |
| TMEM55B | 14 | q11.2 | 1.12E-03 | 1.31E-02 | 0.46 | 1.12 | 0.76 |  |
| AP1G2 | 14 | q11.2 | 1.21E-03 | 1.38E-02 | 0.46 | 1.30 | 0.70 |  |
| PABPN1 | 14 | q11.2 | 1.39E-03 | 1.54E-02 | 0.45 | 1.12 | 0.79 |  |
| APEX1 | 14 | q11.2 | 2.45E-03 | 2.35E-02 | 0.43 | 1.15 | 0.67 |  |
| JUB | 14 | q11.2 | 4.51E-03 | 3.61E-02 | 0.41 | 1.07 | 0.96 |  |
| PARP2 | 14 | q11.2 | 6.61E-03 | 4.77E-02 | 0.39 | 1.13 | 0.85 |  |
| RABGGTA | 14 | q12 | 4.95E-04 | 7.24E-03 | 0.49 | 1.15 | 0.72 |  |
| PRKD1 | 14 | q12 | 1.69E-03 | 1.79E-02 | 0.45 | 1.29 | 0.94 |  |
| RNF31 | 14 | q12 | 4.43E-03 | 3.57E-02 | 0.41 | 1.14 | 0.71 |  |
| LTB4R | 14 | q12 | 6.10E-03 | 4.50E-02 | 0.39 | 1.42 | 0.66 |  |
| NPAS3 | 14 | q13.1 | 2.01E-03 | 2.03E-02 | -0.44 | 0.96 | 1.12 |  |
| SNX6 | 14 | q13.1 | 2.21E-03 | 2.17E-02 | 0.44 | 1.15 | 0.81 |  |
| BAZ1A | 14 | q13.1-q13.2 | 1.26E-03 | 1.43E-02 | 0.46 | 1.27 | 0.75 |  |
| KIAA0391 | 14 | q13.2 | 8.95E-04 | 1.11E-02 | 0.47 | 1.18 | 0.77 |  |
| SRP54 | 14 | q13.2 | 1.18E-03 | 1.37E-02 | 0.46 | 1.25 | 0.78 |  |
| FKBP3 | 14 | q21.2 | 2.17E-04 | 3.86E-03 | 0.51 | 1.22 | 0.98 |  |
| RPL10L | 14 | q21.2 | 5.57E-03 | 4.21E-02 | 0.40 | 1.03 | 0.98 | 1.08 |
| SOS2 | 14 | q21.3 | 3.65E-07 | 3.00E-05 | 0.66 | 1.13 | 0.89 | 2.18 |
| ATP5S | 14 | q21.3 | 5.03E-06 | 2.16E-04 | 0.61 | 1.23 | 0.85 | 3.31 |
| L2HGDH | 14 | q21.3 | 2.92E-05 | 8.32E-04 | 0.57 | 1.53 | 0.81 | 3.11 |
| SDCCAG1 | 14 | q21.3 | 4.18E-05 | 1.10E-03 | 0.56 | 1.40 | 1.03 | 2.46 |
| RPL36AL | 14 | q21.3 | 5.28E-04 | 7.56E-03 | 0.49 | 1.36 | 0.74 | 2.19 |
| PPIL5 | 14 | q21.3 | 8.13E-04 | 1.03E-02 | 0.47 | 1.41 | 0.86 | 2.42 |
| C14orf166 | 14 | q22.1 | 3.15E-03 | 2.80E-02 | 0.42 | 1.43 | 0.74 |  |
| ERO1L | 14 | q22.1 | 3.60E-03 | 3.07E-02 | 0.42 | 1.26 | 0.76 |  |
| GNPNAT1 | 14 | q22.1 | 5.89E-03 | 4.39E-02 | 0.40 | 1.39 | 0.77 |  |
| GMFB | 14 | q22.2 | 3.51E-03 | 3.02E-02 | 0.42 | 1.34 | 0.77 |  |
| MUDENG | 14 | q22.3 | 1.19E-04 | 2.44E-03 | 0.53 | 1.31 | 0.70 |  |
| DHRS7 | 14 | q23.1 | 3.40E-04 | 5.42E-03 | 0.50 | 1.50 | 0.69 |  |
| MNAT1 | 14 | q23.1 | 3.59E-04 | 5.63E-03 | 0.50 | 1.48 | 0.76 |  |
| DAAM1 | 14 | q23.1 | 9.65E-04 | 1.17E-02 | 0.47 | 1.50 | 0.60 |  |
| TOMM20L | 14 | q23.1 | 1.67E-03 | 1.77E-02 | 0.45 | 1.08 | 0.88 |  |
| KIAA0586 | 14 | q23.1 | 1.81E-03 | 1.89E-02 | 0.44 | 1.14 | 0.79 |  |
| ACTR10 | 14 | q23.1 | 5.21E-03 | 4.02E-02 | 0.40 | 1.33 | 0.83 |  |
| SNAPC1 | 14 | q23.2 | 9.58E-05 | 2.07E-03 | 0.54 | 1.12 | 0.85 |  |
| PPP2R5E | 14 | q23.2 | 5.57E-04 | 7.90E-03 | 0.48 | 1.17 | 0.78 |  |
| MTHFD1 | 14 | q23.2-q23.3 | 8.40E-04 | 1.06E-02 | 0.47 | 1.37 | 0.85 |  |
| PLEKHG3 | 14 | q23.3 | 9.83E-05 | 2.11E-03 | 0.54 | 1.77 | 0.82 |  |
| ATP6V1D | 14 | q23.3 | 3.21E-03 | 2.84E-02 | 0.42 | 1.34 | 0.86 |  |
| EIF2S1 | 14 | q23.3 | 4.65E-03 | 3.69E-02 | 0.41 | 1.40 | 0.79 |  |
| MAX | 14 | q23.3 | 5.86E-03 | 4.37E-02 | 0.40 | 1.01 | 0.94 |  |
| WDR22 | 14 | q24.1 | 3.10E-04 | 5.05E-03 | 0.50 | 1.16 | 0.71 |  |
| ERH | 14 | q24.1 | 3.52E-04 | 5.56E-03 | 0.50 | 1.50 | 0.77 |  |
| EXDL2 | 14 | q24.1 | 1.82E-03 | 1.89E-02 | 0.44 | 1.49 | 0.82 |  |
| SLC39A9 | 14 | q24.1 | 3.40E-03 | 2.95E-02 | 0.42 | 1.23 | 0.92 |  |
| ACTN1 | 14 | q24.1 | 4.45E-03 | 3.58E-02 | 0.41 | 1.34 | 0.80 |  |
| MED6 | 14 | q24.2 | 5.95E-05 | 1.44E-03 | 0.55 | 1.14 | 0.75 |  |
| COX16 | 14 | q24.2 | 9.36E-04 | 1.14E-02 | 0.47 | 1.48 | 0.69 |  |
| SFRS5 | 14 | q24.2 | 2.21E-03 | 2.18E-02 | 0.44 | 1.30 | 0.77 |  |
| ZFYVE1 | 14 | q24.2 | 2.49E-03 | 2.37E-02 | 0.43 | 1.24 | 0.83 |  |
| SYNJ2BP | 14 | q24.2 | 3.94E-03 | 3.28E-02 | 0.41 | 1.38 | 0.65 |  |
| WDR21A | 14 | q24.2 | 4.54E-03 | 3.63E-02 | 0.41 | 0.90 | 0.79 |  |
| NUMB | 14 | q24.2-q24.3 | 9.32E-04 | 1.14E-02 | 0.47 | 1.09 | 0.81 |  |
| COQ6 | 14 | q24.3 | 1.69E-05 | 5.44E-04 | 0.58 | 1.22 | 0.70 |  |
| ISCA2 | 14 | q24.3 | 1.74E-05 | 5.53E-04 | 0.58 | 1.33 | 0.66 |  |
| C14orf179 | 14 | q24.3 | 2.37E-05 | 7.03E-04 | 0.57 | 1.19 | 0.68 |  |
| C14orf169 | 14 | q24.3 | 2.23E-04 | 3.93E-03 | 0.51 | 1.15 | 0.70 |  |
| GSTZ1 | 14 | q24.3 | 6.63E-04 | 8.93E-03 | 0.48 | 1.08 | 0.79 |  |
| DLSTP | 14 | q24.3 | 8.28E-04 | 1.05E-02 | 0.47 | 1.01 | 0.74 |  |
| TGFB3 | 14 | q24.3 | 9.61E-04 | 1.17E-02 | 0.47 | 1.73 | 0.56 |  |
| MLH3 | 14 | q24.3 | 2.88E-03 | 2.63E-02 | 0.43 | 1.09 | 0.89 |  |
| ALKBH1 | 14 | q24.3 | 3.80E-03 | 3.20E-02 | 0.41 | 1.20 | 0.87 |  |
| ANGEL1 | 14 | q24.3 | 4.49E-03 | 3.61E-02 | 0.41 | 1.37 | 0.85 |  |
| TTC8 | 14 | q31.3 | 3.62E-03 | 3.09E-02 | 0.42 | 1.29 | 0.76 |  |
| PTPN21 | 14 | q31.3 | 6.20E-03 | 4.55E-02 | 0.39 | 1.20 | 0.90 |  |
| PSMC1 | 14 | q32.11 | 8.56E-05 | 1.90E-03 | 0.54 | 1.48 | 0.69 |  |
| TDP1 | 14 | q32.11 | 2.73E-03 | 2.53E-02 | 0.43 | 1.14 | 0.68 |  |
| DDX24 | 14 | q32.12 | 1.57E-03 | 1.69E-02 | 0.45 | 1.30 | 0.74 |  |
| OTUB2 | 14 | q32.12 | 3.99E-03 | 3.31E-02 | 0.41 | 1.30 | 0.81 |  |
| UBR7 | 14 | q32.12 | 6.31E-03 | 4.62E-02 | 0.39 | 1.23 | 0.69 |  |
| C14orf65 | 14 | q32.2 | 2.02E-04 | 3.66E-03 | 0.52 | 1.21 | 0.73 |  |
| SETD3 | 14 | q32.2 | 3.85E-04 | 5.95E-03 | 0.50 | 1.29 | 0.70 |  |
| WDR20 | 14 | q32.31 | 4.64E-05 | 1.19E-03 | 0.56 | 1.18 | 0.73 |  |
| AL137229.4-1 | 14 | q32.31 | 2.53E-04 | 4.32E-03 | 0.51 | 1.10 | 0.84 |  |
| ZNF839 | 14 | q32.31 | 1.99E-03 | 2.03E-02 | 0.44 | 1.39 | 0.81 |  |
| PPP2R5C | 14 | q32.31 | 6.27E-03 | 4.59E-02 | 0.39 | 1.19 | 0.85 |  |
| MARK3 | 14 | q32.32 | 2.64E-03 | 2.47E-02 | 0.43 | 1.23 | 0.83 |  |
| BTBD6 | 14 | q32.33 | 5.10E-04 | 7.40E-03 | 0.49 | 1.25 | 0.77 |  |
| ADSSL1 | 14 | q32.33 | 7.14E-04 | 9.41E-03 | 0.48 | 1.54 | 0.66 |  |
| ZFYVE21 | 14 | q32.33 | 1.12E-03 | 1.31E-02 | 0.46 | 1.25 | 0.66 |  |
| BAG5 | 14 | q32.33 | 3.20E-03 | 2.84E-02 | 0.42 | 1.16 | 0.86 |  |
| XRCC3 | 14 | q32.33 | 4.34E-03 | 3.52E-02 | 0.41 | 1.16 | 0.81 |  |
| CDCA4 | 14 | q32.33 | 6.30E-03 | 4.61E-02 | 0.39 | 1.21 | 0.85 |  |
| SNRPN | 15 | q11.2 | 2.07E-04 | 3.73E-03 | 0.52 | 1.34 | 0.66 |  |
| NIPA2 | 15 | q11.2 | 2.83E-04 | 4.71E-03 | 0.51 | 1.36 | 0.86 |  |
| NIPA1 | 15 | q11.2 | 5.81E-04 | 8.16E-03 | 0.48 | 1.46 | 0.71 |  |
| UBE3A | 15 | q11.2 | 5.66E-03 | 4.26E-02 | 0.40 | 1.22 | 0.79 |  |
| GABRB3 | 15 | q12 | 3.09E-03 | 2.76E-02 | -0.42 | 0.92 | 1.04 |  |
| ZNF770 | 15 | q14 | 9.98E-05 | 2.13E-03 | 0.54 | 1.10 | 0.72 |  |
| TMEM85 | 15 | q14 | 2.35E-03 | 2.27E-02 | 0.43 | 1.34 | 0.74 |  |
| AQR | 15 | q14 | 2.43E-03 | 2.33E-02 | 0.43 | 1.07 | 0.84 |  |
| C15orf24 | 15 | q14 | 2.88E-03 | 2.63E-02 | 0.43 | 1.20 | 0.68 |  |
| C15orf29 | 15 | q14 | 7.02E-03 | 4.98E-02 | 0.39 | 1.24 | 0.83 |  |
| LRRC57 | 15 | q15.1 | 9.69E-04 | 1.17E-02 | 0.47 | 1.58 | 0.85 |  |
| SPINT1 | 15 | q15.1 | 1.36E-03 | 1.51E-02 | 0.45 | 1.49 | 0.85 |  |
| SNAP23 | 15 | q15.1 | 1.51E-03 | 1.64E-02 | 0.45 | 1.81 | 0.69 |  |
| RPUSD2 | 15 | q15.1 | 4.10E-03 | 3.38E-02 | 0.41 | 1.48 | 0.92 |  |
| AC012652.1 | 15 | q15.1 | 4.73E-03 | 3.73E-02 | 0.41 | 1.17 | 0.76 |  |
| CDAN1 | 15 | q15.2 | 3.67E-03 | 3.12E-02 | 0.42 | 1.21 | 0.96 |  |
| UBR1 | 15 | q15.2 | 3.82E-03 | 3.21E-02 | 0.41 | 1.16 | 0.93 |  |
| LCMT2 | 15 | q15.3 | 1.99E-05 | 6.10E-04 | 0.58 | 3.54 | 0.72 |  |
| ZSCAN29 | 15 | q15.3 | 9.64E-05 | 2.08E-03 | 0.54 | 3.23 | 0.93 |  |
| ADAL | 15 | q15.3 | 6.52E-04 | 8.87E-03 | 0.48 | 2.11 | 0.89 |  |
| HISPPD2A | 15 | q15.3 | 1.02E-03 | 1.23E-02 | 0.46 | 1.41 | 0.79 |  |
| TUBGCP4 | 15 | q15.3 | 2.26E-03 | 2.21E-02 | 0.43 | 2.52 | 0.87 |  |
| SERINC4 | 15 | q15.3 | 4.50E-03 | 3.61E-02 | 0.41 | 1.95 | 0.94 |  |
| CKMT1A | 15 | q15.3 | 5.38E-03 | 4.11E-02 | 0.40 | 2.74 | 0.32 |  |
| SPATA5L1 | 15 | q21.1 | 4.43E-04 | 6.65E-03 | 0.49 | 1.53 | 0.82 | 1.75 |
| SQRDL | 15 | q21.1 | 1.51E-03 | 1.63E-02 | 0.45 | 1.33 | 0.77 | 2.53 |
| SPG11 | 15 | q21.1 | 6.53E-03 | 4.73E-02 | 0.39 | 1.60 | 0.82 |  |
| GABPB1 | 15 | q21.2 | 1.03E-06 | 6.41E-05 | 0.64 | 1.34 | 0.87 | 3.48 |
| MYO5C | 15 | q21.2 | 7.11E-05 | 1.65E-03 | 0.55 | 1.37 | 0.62 | 3.16 |
| AC012100.1 | 15 | q21.2 | 7.41E-04 | 9.68E-03 | 0.48 | 1.46 | 0.83 | 2.43 |
| AP4E1 | 15 | q21.2 | 8.36E-04 | 1.06E-02 | 0.47 | 1.23 | 1.01 | 2.23 |
| TMOD3 | 15 | q21.2 | 1.11E-03 | 1.31E-02 | 0.46 | 1.14 | 1.06 | 1.40 |
| ARPP19 | 15 | q21.2 | 1.79E-03 | 1.87E-02 | 0.44 | 1.25 | 0.96 | 2.24 |
| LEO1 | 15 | q21.2 | 3.83E-03 | 3.22E-02 | 0.41 | 1.58 | 1.00 | 2.13 |
| KIAA1370 | 15 | q21.2-q21.3 | 4.66E-03 | 3.69E-02 | 0.41 | 1.39 | 0.56 | 2.44 |
| RFX7 | 15 | q21.3 | 1.41E-06 | 8.25E-05 | 0.64 | 1.25 | 0.96 |  |
| ZNF280D | 15 | q21.3 | 1.30E-04 | 2.62E-03 | 0.53 | 1.37 | 0.88 |  |
| C15orf15 | 15 | q21.3 | 2.53E-03 | 2.40E-02 | 0.43 | 1.33 | 0.63 |  |
| PIGB | 15 | q21.3 | 6.66E-03 | 4.79E-02 | 0.39 | 1.20 | 0.96 |  |
| NEDD4 | 15 | q21.3 | 6.92E-03 | 4.92E-02 | 0.39 | 1.07 | 0.97 |  |
| NARG2 | 15 | q22.2 | 1.38E-06 | 8.08E-05 | 0.64 | 1.39 | 0.89 |  |
| MYO1E | 15 | q22.2 | 3.65E-05 | 9.88E-04 | 0.56 | 1.34 | 1.07 |  |
| ANXA2 | 15 | q22.2 | 4.37E-05 | 1.14E-03 | 0.56 | 1.65 | 1.04 |  |
| LACTB | 15 | q22.2 | 1.27E-03 | 1.43E-02 | 0.46 | 1.28 | 0.92 |  |
| CCNB2 | 15 | q22.2 | 3.71E-03 | 3.15E-02 | 0.42 | 2.07 | 0.95 |  |
| GTF2A2 | 15 | q22.2 | 3.90E-03 | 3.26E-02 | 0.41 | 1.52 | 0.91 |  |
| DIS3L | 15 | q22.31 | 4.23E-04 | 6.41E-03 | 0.49 | 1.65 | 0.89 |  |
| TIPIN | 15 | q22.31 | 6.62E-04 | 8.92E-03 | 0.48 | 1.40 | 0.95 |  |
| C15orf44 | 15 | q22.31 | 7.61E-04 | 9.88E-03 | 0.47 | 1.39 | 0.88 |  |
| PTPLAD1 | 15 | q22.31 | 1.50E-03 | 1.62E-02 | 0.45 | 1.57 | 0.62 |  |
| USP3 | 15 | q22.31 | 1.98E-03 | 2.02E-02 | 0.44 | 1.28 | 0.85 |  |
| RAB11A | 15 | q22.31 | 2.31E-03 | 2.24E-02 | 0.43 | 1.48 | 0.74 |  |
| MTFMT | 15 | q22.31 | 2.57E-03 | 2.42E-02 | 0.43 | 0.96 | 0.86 |  |
| DPP8 | 15 | q22.31 | 2.65E-03 | 2.48E-02 | 0.43 | 1.33 | 0.79 |  |
| ZNF609 | 15 | q22.31 | 2.69E-03 | 2.50E-02 | 0.43 | 1.73 | 0.90 |  |
| PDCD7 | 15 | q22.31 | 3.64E-03 | 3.10E-02 | 0.42 | 0.88 | 0.84 |  |
| SLC24A1 | 15 | q22.31 | 3.86E-03 | 3.24E-02 | 0.41 | 1.26 | 0.76 |  |
| CSNK1G1 | 15 | q22.31 | 4.24E-03 | 3.45E-02 | 0.41 | 1.49 | 0.74 |  |
| MAP2K1 | 15 | q22.31 | 5.30E-03 | 4.07E-02 | 0.40 | 1.47 | 0.94 |  |
| LARP6 | 15 | q23 | 1.46E-04 | 2.84E-03 | 0.53 | 1.74 | 0.82 |  |
| THAP10 | 15 | q23 | 2.06E-04 | 3.72E-03 | 0.52 | 1.51 | 0.81 |  |
| CALML4 | 15 | q23 | 6.19E-04 | 8.52E-03 | 0.48 | 1.56 | 0.91 |  |
| LRRC49 | 15 | q23 | 1.52E-03 | 1.65E-02 | 0.45 | 1.41 | 0.91 |  |
| SENP8 | 15 | q23 | 1.71E-03 | 1.80E-02 | 0.45 | 1.11 | 0.93 |  |
| RPLP1 | 15 | q23 | 2.01E-03 | 2.03E-02 | 0.44 | 1.42 | 0.40 |  |
| PARP6 | 15 | q23 | 6.18E-03 | 4.54E-02 | 0.39 | 1.46 | 0.77 |  |
| HIGD2B | 15 | q24.1 | 4.44E-16 | 2.11E-12 | 0.88 | 1.18 | 1.00 | 2.90 |
| NEO1 | 15 | q24.1 | 2.30E-09 | 5.21E-07 | 0.74 | 1.63 | 0.78 | 4.51 |
| ARIH1 | 15 | q24.1 | 7.47E-06 | 2.95E-04 | 0.60 | 1.42 | 0.85 |  |
| NPTN | 15 | q24.1 | 2.41E-05 | 7.13E-04 | 0.57 | 1.72 | 0.51 | 3.08 |
| ARID3B | 15 | q24.1 | 6.15E-05 | 1.48E-03 | 0.55 | 1.44 | 0.86 |  |
| GOLGA6C | 15 | q24.1 | 1.16E-03 | 1.35E-02 | 0.46 | 1.09 | 1.02 | 1.31 |
| UBL7 | 15 | q24.1 | 2.73E-03 | 2.53E-02 | 0.43 | 1.18 | 0.91 |  |
| C15orf17 | 15 | q24.1 | 2.81E-03 | 2.58E-02 | 0.43 | 1.39 | 0.91 |  |
| FBXO22 | 15 | q24.2 | 1.56E-05 | 5.14E-04 | 0.59 | 1.50 | 0.82 |  |
| IMP3 | 15 | q24.2 | 2.71E-05 | 7.84E-04 | 0.57 | 1.55 | 0.72 |  |
| SIN3A | 15 | q24.2 | 2.57E-03 | 2.42E-02 | 0.43 | 1.36 | 0.76 |  |
| ETFA | 15 | q24.2-q24.3 | 1.04E-03 | 1.24E-02 | 0.46 | 1.80 | 0.72 |  |
| RCN2 | 15 | q24.3 | 8.91E-06 | 3.38E-04 | 0.60 | 1.80 | 0.59 |  |
| HMG20A | 15 | q24.3 | 2.35E-03 | 2.27E-02 | 0.43 | 1.33 | 0.87 |  |
| SCAPER | 15 | q24.3 | 3.99E-03 | 3.31E-02 | 0.41 | 1.16 | 0.90 |  |
| PSMA4 | 15 | q25.1 | 2.46E-05 | 7.25E-04 | 0.57 | 1.49 | 0.84 |  |
| WDR61 | 15 | q25.1 | 9.97E-05 | 2.13E-03 | 0.54 | 1.65 | 0.75 |  |
| MORF4L1 | 15 | q25.1 | 1.05E-04 | 2.22E-03 | 0.54 | 2.06 | 0.51 |  |
| IDH3A | 15 | q25.1 | 1.38E-03 | 1.53E-02 | 0.45 | 1.74 | 0.90 |  |
| MTHFS | 15 | q25.1 | 2.79E-03 | 2.57E-02 | 0.43 | 1.38 | 0.76 |  |
| KIAA1024 | 15 | q25.1 | 4.36E-03 | 3.52E-02 | 0.41 | 1.50 | 0.96 |  |
| ARNT2 | 15 | q25.1 | 5.51E-03 | 4.17E-02 | 0.40 | 1.84 | 0.49 |  |
| RPS17 | 15 | q25.2 | 3.47E-03 | 2.99E-02 | 0.42 | 1.40 | 0.89 |  |
| WDR73 | 15 | q25.2 | 3.78E-03 | 3.19E-02 | 0.41 | 1.34 | 0.84 |  |
| RKHD3 | 15 | q25.2 | 4.38E-03 | 3.54E-02 | 0.41 | 1.21 | 0.96 |  |
| HOMER2 | 15 | q25.2 | 5.04E-03 | 3.90E-02 | 0.40 | 1.70 | 0.81 |  |
| SEC11B | 15 | q25.3 | 3.96E-03 | 3.29E-02 | 0.41 | 1.09 | 0.87 |  |
| FANCI | 15 | q26.1 | 3.38E-04 | 5.40E-03 | 0.50 | 1.58 | 0.72 |  |
| SEMA4B | 15 | q26.1 | 5.64E-04 | 7.99E-03 | 0.48 | 1.86 | 0.81 |  |
| FAM174B | 15 | q26.1 | 1.27E-03 | 1.44E-02 | 0.46 | 1.90 | 0.73 |  |
| PRC1 | 15 | q26.1 | 3.80E-03 | 3.20E-02 | 0.41 | 1.57 | 0.59 |  |
| NGRN | 15 | q26.1 | 5.86E-03 | 4.37E-02 | 0.40 | 1.29 | 0.85 |  |
| FES | 15 | q26.1 | 6.62E-03 | 4.77E-02 | -0.39 | 0.78 | 1.40 |  |
| IGF1R | 15 | q26.3 | 5.84E-10 | 1.75E-07 | 0.76 | 1.39 | 0.97 | 3.18 |
| TARSL2 | 15 | q26.3 | 2.73E-03 | 2.53E-02 | 0.43 | 1.20 | 1.02 |  |
| SNRPA1 | 15 | q26.3 | 3.42E-03 | 2.96E-02 | 0.42 | 1.44 | 0.87 |  |
| LINS1 | 15 | q26.3 | 4.18E-03 | 3.42E-02 | 0.41 | 1.16 | 0.95 |  |
| AC138915.1 | 16 | p11.1 | 2.64E-04 | 4.47E-03 | -0.51 | 0.96 | 1.09 | 0.84 |
| ZNF688 | 16 | p11.2 | 2.91E-07 | 2.52E-05 | 0.67 | 1.33 | 0.84 | 2.05 |
| AC074050.1 | 16 | p11.2 | 1.70E-06 | 9.67E-05 | 0.63 | 1.27 | 0.62 | 1.95 |
| HIRIP3 | 16 | p11.2 | 1.73E-06 | 9.79E-05 | 0.63 | 1.51 | 0.88 | 2.39 |
| CD2BP2 | 16 | p11.2 | 1.86E-06 | 1.02E-04 | 0.63 | 1.33 | 0.83 | 3.10 |
| ZNF764 | 16 | p11.2 | 2.12E-06 | 1.11E-04 | 0.63 | 1.21 | 0.88 | 1.91 |
| TUFM | 16 | p11.2 | 4.45E-06 | 1.96E-04 | 0.61 | 1.25 | 0.83 | 2.53 |
| SH2B1 | 16 | p11.2 | 8.89E-06 | 3.38E-04 | 0.60 | 1.42 | 1.06 | 1.92 |
| ZNF689 | 16 | p11.2 | 3.65E-05 | 9.88E-04 | 0.56 | 1.68 | 0.91 | 6.06 |
| CLN3 | 16 | p11.2 | 3.86E-05 | 1.03E-03 | 0.56 | 1.43 | 0.85 | 3.79 |
| BCKDK | 16 | p11.2 | 3.86E-05 | 1.03E-03 | 0.56 | 1.44 | 0.86 |  |
| MYST1 | 16 | p11.2 | 4.74E-05 | 1.21E-03 | 0.56 | 1.29 | 0.79 |  |
| AC025279.6-2 | 16 | p11.2 | 6.49E-05 | 1.54E-03 | 0.55 | 1.31 | 0.71 | 2.29 |
| SBK1 | 16 | p11.2 | 9.30E-05 | 2.02E-03 | 0.54 | 1.59 | 0.62 | 6.28 |
| SEPHS2 | 16 | p11.2 | 1.23E-04 | 2.51E-03 | 0.53 | 1.66 | 0.72 | 2.60 |
| NFATC2IP | 16 | p11.2 | 1.53E-04 | 2.94E-03 | 0.52 | 1.34 | 0.77 | 1.63 |
| SPNS1 | 16 | p11.2 | 2.27E-04 | 3.97E-03 | 0.51 | 1.41 | 0.82 | 2.52 |
| TBC1D10B | 16 | p11.2 | 2.43E-04 | 4.20E-03 | 0.51 | 1.33 | 0.76 | 2.79 |
| PPP4C | 16 | p11.2 | 2.66E-04 | 4.48E-03 | 0.51 | 1.31 | 0.81 | 1.94 |
| BCL7C | 16 | p11.2 | 2.66E-04 | 4.48E-03 | 0.51 | 1.45 | 0.72 |  |
| PRR14 | 16 | p11.2 | 3.84E-04 | 5.94E-03 | 0.50 | 1.51 | 0.82 |  |
| XPO6 | 16 | p11.2 | 4.18E-04 | 6.37E-03 | 0.49 | 1.32 | 0.81 | 2.03 |
| ZNF768 | 16 | p11.2 | 4.75E-04 | 7.01E-03 | 0.49 | 1.39 | 0.87 | 2.35 |
| ZNF48 | 16 | p11.2 | 4.96E-04 | 7.24E-03 | 0.49 | 1.10 | 0.90 | 2.01 |
| ZNF771 | 16 | p11.2 | 5.18E-04 | 7.47E-03 | 0.49 | 1.05 | 0.95 | 1.21 |
| INO80E | 16 | p11.2 | 7.93E-04 | 1.02E-02 | 0.47 | 1.60 | 0.70 | 1.52 |
| KCTD13 | 16 | p11.2 | 7.97E-04 | 1.02E-02 | 0.47 | 1.54 | 0.77 | 1.85 |
| SULT1A4 | 16 | p11.2 | 8.01E-04 | 1.02E-02 | 0.47 | 1.35 | 0.81 | 1.80 |
| PHKG2 | 16 | p11.2 | 8.06E-04 | 1.03E-02 | 0.47 | 1.28 | 0.90 |  |
| MAPK3 | 16 | p11.2 | 8.22E-04 | 1.04E-02 | 0.47 | 1.34 | 0.96 | 1.54 |
| AC133555.3-2 | 16 | p11.2 | 8.84E-04 | 1.10E-02 | 0.47 | 1.06 | 0.94 | 1.18 |
| DCTPP1 | 16 | p11.2 | 1.15E-03 | 1.34E-02 | 0.46 | 1.25 | 0.75 | 1.78 |
| CCDC101 | 16 | p11.2 | 2.57E-03 | 2.42E-02 | 0.43 | 1.06 | 0.87 | 1.56 |
| ZNF629 | 16 | p11.2 | 3.14E-03 | 2.79E-02 | 0.42 | 1.43 | 0.90 |  |
| C16orf58 | 16 | p11.2 | 3.41E-03 | 2.96E-02 | 0.42 | 1.27 | 0.93 |  |
| TAOK2 | 16 | p11.2 | 3.49E-03 | 3.00E-02 | 0.42 | 1.33 | 0.89 | 2.64 |
| TBX6 | 16 | p11.2 | 3.65E-03 | 3.11E-02 | 0.42 | 1.10 | 0.94 | 1.08 |
| ZNF747 | 16 | p11.2 | 4.10E-03 | 3.38E-02 | 0.41 | 1.12 | 0.95 | 1.79 |
| C16orf53 | 16 | p11.2 | 4.93E-03 | 3.85E-02 | 0.40 | 1.38 | 0.71 | 1.36 |
| AC009086.6-2 | 16 | p11.2 | 5.27E-03 | 4.06E-02 | 0.40 | 1.11 | 0.98 | 1.36 |
| RABEP2 | 16 | p11.2 | 5.31E-03 | 4.08E-02 | 0.40 | 1.05 | 0.94 | 1.40 |
| SETD1A | 16 | p11.2 | 6.53E-03 | 4.73E-02 | 0.39 | 1.29 | 0.86 |  |
| EIF3CL | 16 | p11.2 | 6.83E-03 | 4.88E-02 | 0.39 | 1.34 | 0.88 | 1.47 |
| TNRC6A | 16 | p12.1 | 1.18E-05 | 4.18E-04 | 0.59 | 1.24 | 1.01 | 1.56 |
| AC133552.3 | 16 | p12.1 | 2.24E-05 | 6.73E-04 | 0.58 | 1.10 | 0.92 | 2.69 |
| LCMT1 | 16 | p12.1 | 9.12E-05 | 1.99E-03 | 0.54 | 1.27 | 0.64 | 3.16 |
| ARHGAP17 | 16 | p12.1 | 3.33E-04 | 5.34E-03 | 0.50 | 1.18 | 1.02 | 1.76 |
| JMJD5 | 16 | p12.1 | 1.61E-03 | 1.72E-02 | 0.45 | 1.06 | 0.89 | 1.21 |
| NSMCE1 | 16 | p12.1 | 3.03E-03 | 2.73E-02 | 0.42 | 1.24 | 0.73 | 1.87 |
| GTF3C1 | 16 | p12.1 | 3.34E-03 | 2.92E-02 | 0.42 | 1.28 | 0.62 | 1.65 |
| KIAA0556 | 16 | p12.1-p11.2 | 4.82E-06 | 2.08E-04 | 0.61 | 1.34 | 0.86 | 1.71 |
| EARS2 | 16 | p12.2 | 5.29E-04 | 7.57E-03 | 0.49 | 1.21 | 0.85 | 1.77 |
| AC008740.7-1 | 16 | p12.2 | 5.72E-04 | 8.08E-03 | 0.48 | 1.52 |  | 3.02 |
| DCTN5 | 16 | p12.2 | 8.93E-04 | 1.11E-02 | 0.47 | 1.18 | 0.81 | 1.39 |
| EEF2K | 16 | p12.2 | 1.24E-03 | 1.41E-02 | 0.46 | 1.26 |  | 3.39 |
| POLR3E | 16 | p12.2 | 2.91E-03 | 2.65E-02 | 0.42 | 1.24 |  | 1.65 |
| COQ7 | 16 | p12.3 | 6.24E-05 | 1.49E-03 | 0.55 | 1.24 | 0.66 | 1.57 |
| SYT7 | 16 | p12.3 | 2.75E-03 | 2.54E-02 | 0.43 | 1.62 | 0.76 | 3.55 |
| ARL6IP1 | 16 | p12.3 | 3.25E-03 | 2.86E-02 | 0.42 | 1.30 | 0.48 | 1.73 |
| AC012621.2 | 16 | p12.3 | 4.73E-03 | 3.73E-02 | 0.41 | 1.25 | 0.77 | 1.67 |
| C16orf63 | 16 | p13.11 | 3.68E-05 | 9.93E-04 | 0.56 | 1.20 | 0.72 | 1.94 |
| NDE1 | 16 | p13.11 | 2.62E-04 | 4.44E-03 | 0.51 | 1.36 | 0.67 | 1.17 |
| NPIP | 16 | p13.11 | 1.40E-03 | 1.54E-02 | 0.45 | 1.35 | 1.01 | 1.14 |
| KIAA0430 | 16 | p13.11 | 2.14E-03 | 2.12E-02 | 0.44 | 1.20 | 0.75 | 1.40 |
| BFAR | 16 | p13.12 | 5.98E-07 | 4.27E-05 | 0.65 | 1.33 | 0.77 | 1.83 |
| PARN | 16 | p13.12 | 5.16E-06 | 2.20E-04 | 0.61 | 1.28 | 0.75 | 1.50 |
| ERCC4 | 16 | p13.12 | 2.78E-04 | 4.64E-03 | 0.51 | 1.04 | 0.92 | 1.21 |
| CLEC16A | 16 | p13.13 | 3.13E-10 | 1.10E-07 | 0.77 | 1.45 | 0.60 | 2.18 |
| C16orf75 | 16 | p13.13 | 3.87E-04 | 5.97E-03 | 0.50 | 1.58 | 0.82 | 2.00 |
| GSPT1 | 16 | p13.13 | 1.66E-03 | 1.75E-02 | 0.45 | 1.16 | 0.56 | 1.42 |
| ZC3H7A | 16 | p13.13 | 2.92E-03 | 2.65E-02 | 0.42 | 1.16 | 0.85 | 1.69 |
| NUBP1 | 16 | p13.13 | 3.12E-03 | 2.78E-02 | 0.42 | 1.17 | 0.71 | 1.58 |
| LITAF | 16 | p13.13 | 3.87E-03 | 3.24E-02 | 0.41 | 1.11 | 1.10 | 2.74 |
| TXNDC11 | 16 | p13.13 | 6.53E-03 | 4.73E-02 | 0.39 | 1.17 | 0.93 | 1.59 |
| C16orf68 | 16 | p13.2 | 8.57E-05 | 1.90E-03 | 0.54 | 1.32 | 0.69 | 1.59 |
| CARHSP1 | 16 | p13.2 | 7.74E-04 | 1.00E-02 | 0.47 | 1.42 | 0.56 | 1.71 |
| USP7 | 16 | p13.2 | 2.56E-03 | 2.42E-02 | 0.43 | 1.33 | 0.65 | 1.45 |
| C16orf51 | 16 | p13.2 | 5.17E-03 | 3.99E-02 | 0.40 | 1.21 | 0.96 | 1.22 |
| CLUAP1 | 16 | p13.3 | 1.98E-06 | 1.06E-04 | 0.63 | 1.39 | 0.75 |  |
| RAB11FIP3 | 16 | p13.3 | 2.58E-06 | 1.28E-04 | 0.63 | 1.36 | 0.56 |  |
| TCEB2 | 16 | p13.3 | 2.68E-06 | 1.31E-04 | 0.62 | 1.25 | 0.71 |  |
| BTBD12 | 16 | p13.3 | 1.18E-05 | 4.18E-04 | 0.59 | 1.24 | 0.71 |  |
| UBE2I | 16 | p13.3 | 2.31E-05 | 6.90E-04 | 0.58 | 1.25 | 0.78 | 1.74 |
| ZNF500 | 16 | p13.3 | 3.19E-05 | 8.96E-04 | 0.57 | 1.37 | 0.77 |  |
| HAGH | 16 | p13.3 | 3.57E-05 | 9.73E-04 | 0.56 | 1.60 | 0.58 |  |
| METRN | 16 | p13.3 | 4.50E-05 | 1.16E-03 | 0.56 | 1.60 | 0.38 |  |
| JMJD8 | 16 | p13.3 | 4.62E-05 | 1.19E-03 | 0.56 | 1.28 | 0.71 |  |
| NAT15 | 16 | p13.3 | 5.56E-05 | 1.36E-03 | 0.55 | 1.36 | 0.66 |  |
| ZNF263 | 16 | p13.3 | 5.65E-05 | 1.38E-03 | 0.55 | 1.33 | 0.77 |  |
| FAHD1 | 16 | p13.3 | 5.68E-05 | 1.39E-03 | 0.55 | 1.39 | 0.74 |  |
| DCI | 16 | p13.3 | 6.47E-05 | 1.54E-03 | 0.55 | 1.47 | 0.65 |  |
| UBN1 | 16 | p13.3 | 6.55E-05 | 1.55E-03 | 0.55 | 1.27 | 0.75 |  |
| CCDC154 | 16 | p13.3 | 7.18E-05 | 1.67E-03 | 0.55 | 1.26 | 0.80 | 2.01 |
| NTHL1 | 16 | p13.3 | 7.88E-05 | 1.79E-03 | 0.54 | 1.35 | 0.55 |  |
| C16orf91 | 16 | p13.3 | 1.00E-04 | 2.14E-03 | 0.54 | 1.27 | 0.73 | 1.71 |
| CREBBP | 16 | p13.3 | 1.00E-04 | 2.14E-03 | 0.54 | 1.35 | 0.77 |  |
| RNPS1 | 16 | p13.3 | 1.41E-04 | 2.77E-03 | 0.53 | 1.49 | 0.72 |  |
| WDR90 | 16 | p13.3 | 1.47E-04 | 2.85E-03 | 0.53 | 1.43 | 0.61 |  |
| AXIN1 | 16 | p13.3 | 1.62E-04 | 3.06E-03 | 0.52 | 1.26 | 0.69 |  |
| AC009171.3 | 16 | p13.3 | 1.70E-04 | 3.18E-03 | 0.52 | 1.41 | 0.64 |  |
| SRRM2 | 16 | p13.3 | 2.31E-04 | 4.03E-03 | 0.51 | 1.60 | 0.70 |  |
| GBL | 16 | p13.3 | 2.37E-04 | 4.12E-03 | 0.51 | 1.36 | 0.71 |  |
| CRAMP1L | 16 | p13.3 | 2.49E-04 | 4.28E-03 | 0.51 | 1.39 | 0.65 | 1.25 |
| FAM173A | 16 | p13.3 | 3.16E-04 | 5.13E-03 | 0.50 | 1.23 | 0.56 |  |
| ZNF174 | 16 | p13.3 | 3.78E-04 | 5.87E-03 | 0.50 | 1.13 | 0.87 |  |
| RHOT2 | 16 | p13.3 | 4.20E-04 | 6.40E-03 | 0.49 | 1.42 | 0.66 |  |
| HCFC1R1 | 16 | p13.3 | 4.58E-04 | 6.83E-03 | 0.49 | 1.37 | 0.60 |  |
| AC020663.7 | 16 | p13.3 | 4.99E-04 | 7.27E-03 | 0.49 | 1.33 | 0.75 |  |
| ROGDI | 16 | p13.3 | 5.08E-04 | 7.38E-03 | 0.49 | 1.56 | 0.66 |  |
| SPSB3 | 16 | p13.3 | 5.88E-04 | 8.23E-03 | 0.48 | 1.38 | 0.68 |  |
| LUC7L | 16 | p13.3 | 6.63E-04 | 8.93E-03 | 0.48 | 1.26 | 0.67 |  |
| SNRNP25 | 16 | p13.3 | 7.12E-04 | 9.40E-03 | 0.48 | 1.16 | 0.58 |  |
| DNAJA3 | 16 | p13.3 | 7.55E-04 | 9.83E-03 | 0.47 | 1.05 | 0.74 |  |
| LMF1 | 16 | p13.3 | 7.57E-04 | 9.85E-03 | 0.47 | 1.25 | 0.81 | 1.58 |
| BAIAP3 | 16 | p13.3 | 7.58E-04 | 9.85E-03 | 0.47 | 1.14 | 0.87 | 1.66 |
| ANKS3 | 16 | p13.3 | 8.84E-04 | 1.10E-02 | 0.47 | 1.41 | 0.81 |  |
| C16orf59 | 16 | p13.3 | 8.92E-04 | 1.11E-02 | 0.47 | 1.38 | 0.62 |  |
| WDR24 | 16 | p13.3 | 1.09E-03 | 1.29E-02 | 0.46 | 1.23 | 0.70 |  |
| MGRN1 | 16 | p13.3 | 1.12E-03 | 1.31E-02 | 0.46 | 1.42 | 0.69 |  |
| TPSB2 | 16 | p13.3 | 1.22E-03 | 1.40E-02 | 0.46 | 2.20 | 0.77 | 3.69 |
| ZNF434 | 16 | p13.3 | 1.27E-03 | 1.43E-02 | 0.46 | 1.23 | 0.72 |  |
| TRAP1 | 16 | p13.3 | 1.40E-03 | 1.54E-02 | 0.45 | 1.24 | 0.77 |  |
| MAPK8IP3 | 16 | p13.3 | 1.43E-03 | 1.56E-02 | 0.45 | 1.37 | 0.72 |  |
| STUB1 | 16 | p13.3 | 1.72E-03 | 1.80E-02 | 0.45 | 1.39 | 0.66 |  |
| PIGQ | 16 | p13.3 | 1.81E-03 | 1.89E-02 | 0.44 | 1.50 | 0.62 |  |
| RAB40C | 16 | p13.3 | 1.99E-03 | 2.02E-02 | 0.44 | 1.43 | 0.76 |  |
| NARFL | 16 | p13.3 | 1.99E-03 | 2.02E-02 | 0.44 | 1.25 | 0.77 |  |
| C16orf71 | 16 | p13.3 | 1.99E-03 | 2.02E-02 | 0.44 | 1.07 | 0.94 |  |
| FAM100A | 16 | p13.3 | 2.00E-03 | 2.03E-02 | 0.44 | 1.17 | 0.69 |  |
| NDUFB10 | 16 | p13.3 | 2.09E-03 | 2.09E-02 | 0.44 | 1.20 | 0.73 |  |
| NME3 | 16 | p13.3 | 2.10E-03 | 2.10E-02 | 0.44 | 1.29 | 0.44 |  |
| ADCY9 | 16 | p13.3 | 2.22E-03 | 2.18E-02 | 0.44 | 1.28 | 0.77 |  |
| ITFG3 | 16 | p13.3 | 2.47E-03 | 2.36E-02 | 0.43 | 1.28 | 0.62 |  |
| MRPS34 | 16 | p13.3 | 2.56E-03 | 2.42E-02 | 0.43 | 1.19 | 0.59 |  |
| E4F1 | 16 | p13.3 | 2.65E-03 | 2.48E-02 | 0.43 | 1.33 | 0.71 |  |
| POLR3K | 16 | p13.3 | 2.79E-03 | 2.57E-02 | 0.43 | 1.31 | 0.60 |  |
| C16orf79 | 16 | p13.3 | 2.94E-03 | 2.67E-02 | 0.42 | 1.34 | 0.73 |  |
| ATP6V0C | 16 | p13.3 | 3.20E-03 | 2.84E-02 | 0.42 | 1.19 | 0.83 |  |
| CCDC78 | 16 | p13.3 | 3.47E-03 | 2.99E-02 | 0.42 | 1.06 | 0.90 |  |
| TBL3 | 16 | p13.3 | 4.04E-03 | 3.33E-02 | 0.41 | 1.43 | 0.72 |  |
| RHBDF1 | 16 | p13.3 | 4.23E-03 | 3.45E-02 | 0.41 | 1.33 | 0.58 |  |
| TSC2 | 16 | p13.3 | 4.35E-03 | 3.52E-02 | 0.41 | 1.49 | 0.74 |  |
| NAGPA | 16 | p13.3 | 4.38E-03 | 3.54E-02 | 0.41 | 1.19 | 0.88 |  |
| CHTF18 | 16 | p13.3 | 4.64E-03 | 3.68E-02 | 0.41 | 1.39 | 0.71 |  |
| ZNF200 | 16 | p13.3 | 4.76E-03 | 3.75E-02 | 0.40 | 1.27 | 0.89 |  |
| CCNF | 16 | p13.3 | 4.85E-03 | 3.80E-02 | 0.40 | 1.28 | 0.84 |  |
| SOLH | 16 | p13.3 | 5.61E-03 | 4.23E-02 | 0.40 | 1.30 | 0.85 |  |
| NME4 | 16 | p13.3 | 5.81E-03 | 4.35E-02 | 0.40 | 1.36 | 0.72 |  |
| NUBP2 | 16 | p13.3 | 6.33E-03 | 4.63E-02 | 0.39 | 1.55 | 0.70 |  |
| C16orf28 | 16 | p13.3 | 6.36E-03 | 4.64E-02 | 0.39 | 1.26 | 0.71 | 1.63 |
| GNPTG | 16 | p13.3 | 6.46E-03 | 4.70E-02 | 0.39 | 1.34 | 0.69 | 1.37 |
| Z84723.2 | 16 | p13.3 | 6.53E-03 | 4.73E-02 | 0.39 | 1.08 | 0.89 |  |
| FLYWCH2 | 16 | p13.3 | 6.89E-03 | 4.91E-02 | 0.39 | 1.40 | 0.58 |  |
| AC141586.5 | 16 | p13.3 | 7.01E-03 | 4.97E-02 | 0.39 | 1.17 | 0.86 |  |
| ORC6L | 16 | q11.2 | 4.57E-10 | 1.48E-07 | 0.76 | 1.30 | 0.80 | 2.25 |
| C16orf87 | 16 | q11.2 | 2.64E-05 | 7.69E-04 | 0.57 | 1.59 | 0.78 | 2.36 |
| GPT2 | 16 | q11.2 | 1.00E-04 | 2.14E-03 | 0.54 | 1.72 | 0.55 | 3.54 |
| VPS35 | 16 | q11.2 | 3.19E-03 | 2.83E-02 | 0.42 | 1.98 | 0.61 | 3.81 |
| DNAJA2 | 16 | q11.2-q12.1 | 2.10E-07 | 1.94E-05 | 0.67 | 1.60 | 0.66 | 2.39 |
| HEATR3 | 16 | q12.1 | 7.59E-08 | 8.48E-06 | 0.69 | 1.40 | 0.70 |  |
| BRD7 | 16 | q12.1 | 6.44E-07 | 4.49E-05 | 0.65 | 1.54 | 0.67 |  |
| PHKB | 16 | q12.1 | 2.22E-06 | 1.15E-04 | 0.63 | 1.63 | 0.70 | 2.59 |
| ITFG1 | 16 | q12.1 | 5.77E-06 | 2.39E-04 | 0.61 | 1.56 | 0.62 | 3.18 |
| SIAH1 | 16 | q12.1 | 2.51E-04 | 4.30E-03 | 0.51 | 1.59 | 0.78 | 4.87 |
| TMEM188 | 16 | q12.1 | 2.54E-04 | 4.33E-03 | 0.51 | 1.40 | 0.73 |  |
| LONP2 | 16 | q12.1 | 1.00E-03 | 1.21E-02 | 0.46 | 1.60 | 0.71 | 3.22 |
| PAPD5 | 16 | q12.1 | 1.19E-03 | 1.37E-02 | 0.46 | 1.32 | 0.73 |  |
| OGFOD1 | 16 | q12.2 | 9.11E-06 | 3.43E-04 | 0.60 |  | 0.69 |  |
| MT1F | 16 | q12.2 | 4.64E-05 | 1.19E-03 | 0.56 |  | 0.48 |  |
| MT2A | 16 | q12.2 | 8.85E-04 | 1.10E-02 | 0.47 |  | 0.70 |  |
| FTS | 16 | q12.2 | 5.56E-03 | 4.21E-02 | 0.40 | 1.07 | 0.77 |  |
| MT1A | 16 | q12.2-q13 | 3.88E-05 | 1.03E-03 | 0.56 |  | 0.59 |  |
| NUP93 | 16 | q13 | 7.58E-09 | 1.43E-06 | 0.73 |  | 0.69 |  |
| MT1X | 16 | q13 | 2.18E-03 | 2.16E-02 | 0.44 |  | 0.62 |  |
| AC009090.12 | 16 | q13 | 4.66E-03 | 3.69E-02 | 0.41 |  | 0.78 |  |
| ARL2BP | 16 | q13 | 5.30E-03 | 4.07E-02 | 0.40 |  | 0.75 |  |
| COQ9 | 16 | q21 | 5.56E-11 | 2.55E-08 | 0.79 |  | 0.63 |  |
| POLR2C | 16 | q21 | 4.12E-08 | 5.27E-06 | 0.70 |  | 0.64 |  |
| CIAPIN1 | 16 | q21 | 1.58E-07 | 1.54E-05 | 0.68 |  | 0.73 |  |
| C16orf80 | 16 | q21 | 1.91E-07 | 1.80E-05 | 0.68 |  | 0.79 |  |
| KATNB1 | 16 | q21 | 2.15E-06 | 1.12E-04 | 0.63 |  | 0.67 |  |
| CSNK2A2 | 16 | q21 | 2.96E-06 | 1.41E-04 | 0.62 |  | 0.70 |  |
| CNOT1 | 16 | q21 | 3.45E-05 | 9.49E-04 | 0.57 |  | 0.72 |  |
| GPR56 | 16 | q21 | 5.42E-05 | 1.34E-03 | 0.55 |  | 0.56 |  |
| ZNF319 | 16 | q21 | 5.80E-05 | 1.41E-03 | 0.55 |  | 0.81 |  |
| C16orf57 | 16 | q21 | 6.00E-05 | 1.45E-03 | 0.55 |  | 0.77 |  |
| GOT2 | 16 | q21 | 1.82E-03 | 1.89E-02 | 0.44 |  | 0.72 |  |
| SLC38A7 | 16 | q21 | 2.12E-03 | 2.11E-02 | 0.44 |  | 0.84 |  |
| VPS4A | 16 | q22.1 | 1.59E-09 | 3.94E-07 | 0.75 |  | 0.71 |  |
| TMEM208 | 16 | q22.1 | 1.72E-09 | 4.22E-07 | 0.75 |  | 0.63 |  |
| FAM96B | 16 | q22.1 | 1.79E-08 | 2.77E-06 | 0.71 |  | 0.65 |  |
| NUTF2 | 16 | q22.1 | 5.63E-08 | 6.71E-06 | 0.70 |  | 0.69 |  |
| DUS2L | 16 | q22.1 | 4.32E-07 | 3.34E-05 | 0.66 |  | 0.73 |  |
| ACD | 16 | q22.1 | 4.76E-07 | 3.61E-05 | 0.66 |  | 0.70 |  |
| CYB5B | 16 | q22.1 | 6.59E-06 | 2.65E-04 | 0.61 |  | 0.79 |  |
| E2F4 | 16 | q22.1 | 1.94E-05 | 6.00E-04 | 0.58 |  | 0.83 |  |
| CIRH1A | 16 | q22.1 | 2.59E-05 | 7.57E-04 | 0.57 |  | 0.71 |  |
| AARS | 16 | q22.1 | 3.40E-05 | 9.39E-04 | 0.57 | 1.78 | 0.78 |  |
| PSKH1 | 16 | q22.1 | 4.87E-05 | 1.23E-03 | 0.56 |  | 0.88 |  |
| NIP7 | 16 | q22.1 | 5.72E-05 | 1.39E-03 | 0.55 |  | 0.79 |  |
| RANBP10 | 16 | q22.1 | 1.22E-04 | 2.48E-03 | 0.53 |  | 0.82 |  |
| FUK | 16 | q22.1 | 1.26E-04 | 2.55E-03 | 0.53 | 1.61 | 0.72 |  |
| APPBP1 | 16 | q22.1 | 1.31E-04 | 2.63E-03 | 0.53 |  | 0.69 |  |
| EDC4 | 16 | q22.1 | 2.89E-04 | 4.79E-03 | 0.51 |  | 0.75 |  |
| CENPT | 16 | q22.1 | 3.08E-04 | 5.02E-03 | 0.50 |  | 0.75 |  |
| PDPR | 16 | q22.1 | 3.67E-04 | 5.73E-03 | 0.50 | 1.56 | 0.73 |  |
| CES2 | 16 | q22.1 | 4.93E-04 | 7.22E-03 | 0.49 |  | 0.75 |  |
| PARD6A | 16 | q22.1 | 5.27E-04 | 7.56E-03 | 0.49 |  | 0.69 |  |
| DDX19A | 16 | q22.1 | 5.76E-04 | 8.12E-03 | 0.48 | 1.57 | 0.86 |  |
| TRADD | 16 | q22.1 | 5.84E-04 | 8.19E-03 | 0.48 |  | 0.80 |  |
| COG4 | 16 | q22.1 | 6.19E-04 | 8.52E-03 | 0.48 | 1.11 | 0.72 |  |
| ELMO3 | 16 | q22.1 | 6.71E-04 | 9.00E-03 | 0.48 |  | 0.53 |  |
| PRMT7 | 16 | q22.1 | 7.48E-04 | 9.75E-03 | 0.47 |  | 0.73 |  |
| SLC7A6OS | 16 | q22.1 | 1.02E-03 | 1.23E-02 | 0.46 |  | 0.92 |  |
| ATP6V0D1 | 16 | q22.1 | 1.13E-03 | 1.32E-02 | 0.46 |  | 0.71 |  |
| EXOSC6 | 16 | q22.1 | 1.27E-03 | 1.44E-02 | 0.46 | 1.90 | 0.72 |  |
| NOL3 | 16 | q22.1 | 1.50E-03 | 1.63E-02 | 0.45 |  | 0.55 |  |
| FBXL8 | 16 | q22.1 | 1.67E-03 | 1.77E-02 | 0.45 |  | 0.82 |  |
| DYNC1LI2 | 16 | q22.1 | 2.17E-03 | 2.15E-02 | 0.44 |  | 0.78 |  |
| WWP2 | 16 | q22.1 | 2.49E-03 | 2.37E-02 | 0.43 |  | 0.79 |  |
| SF3B3 | 16 | q22.1 | 2.77E-03 | 2.56E-02 | 0.43 | 1.95 | 0.83 |  |
| TERF2 | 16 | q22.1 | 2.82E-03 | 2.59E-02 | 0.43 |  | 0.82 |  |
| NOB1 | 16 | q22.1 | 3.71E-03 | 3.15E-02 | 0.42 |  | 0.79 |  |
| LYPLA3 | 16 | q22.1 | 5.88E-03 | 4.39E-02 | 0.40 |  | 0.84 |  |
| DHX38 | 16 | q22.2 | 2.53E-06 | 1.27E-04 | 0.63 | 1.42 | 0.81 |  |
| ZNF23 | 16 | q22.2 | 6.58E-05 | 1.55E-03 | 0.55 | 1.51 | 0.84 |  |
| PHLPPL | 16 | q22.2 | 8.76E-04 | 1.10E-02 | 0.47 | 1.32 | 0.88 |  |
| KIAA0174 | 16 | q22.2 | 1.91E-03 | 1.96E-02 | 0.44 | 1.56 | 0.82 |  |
| ATXN1L | 16 | q22.2 | 2.21E-03 | 2.17E-02 | 0.44 | 1.75 | 0.83 |  |
| TXNL4B | 16 | q22.2 | 2.93E-03 | 2.66E-02 | 0.42 | 1.17 | 0.86 |  |
| AP1G1 | 16 | q22.2 | 3.23E-03 | 2.85E-02 | 0.42 | 1.58 | 0.80 |  |
| DHODH | 16 | q22.2 | 4.66E-03 | 3.69E-02 | 0.41 | 1.08 | 0.90 |  |
| MON1B | 16 | q23.1 | 1.05E-06 | 6.53E-05 | 0.64 | 1.62 | 0.78 |  |
| KARS | 16 | q23.1 | 2.99E-06 | 1.42E-04 | 0.62 | 1.32 | 0.73 |  |
| TERF2IP | 16 | q23.1 | 1.00E-05 | 3.67E-04 | 0.60 | 1.41 | 0.70 |  |
| RFWD3 | 16 | q23.1 | 1.59E-05 | 5.19E-04 | 0.58 |  | 0.70 |  |
| PSMD7 | 16 | q23.1 | 6.11E-05 | 1.47E-03 | 0.55 | 1.36 | 0.68 |  |
| WDR59 | 16 | q23.1 | 1.02E-04 | 2.16E-03 | 0.54 |  | 0.79 |  |
| BCAR1 | 16 | q23.1 | 3.11E-03 | 2.78E-02 | 0.42 | 1.62 | 0.82 |  |
| GLG1 | 16 | q23.1 | 3.34E-03 | 2.92E-02 | 0.42 | 1.29 | 0.80 |  |
| TMEM170A | 16 | q23.1 | 3.51E-03 | 3.02E-02 | 0.42 | 1.74 | 0.86 |  |
| C16orf61 | 16 | q23.2 | 1.92E-08 | 2.92E-06 | 0.71 |  | 0.62 |  |
| CENPN | 16 | q23.2 | 2.76E-03 | 2.55E-02 | 0.43 | 1.15 | 0.57 |  |
| HSBP1 | 16 | q23.3 | 2.51E-04 | 4.31E-03 | 0.51 |  | 0.66 |  |
| HSDL1 | 16 | q23.3 | 1.03E-03 | 1.23E-02 | 0.46 |  | 0.75 |  |
| MBTPS1 | 16 | q23.3 | 2.07E-03 | 2.08E-02 | 0.44 |  | 0.71 |  |
| MLYCD | 16 | q23.3 | 5.97E-03 | 4.44E-02 | 0.40 |  | 0.85 |  |
| COX4I1 | 16 | q24.1 | 5.53E-07 | 4.06E-05 | 0.66 |  | 0.72 |  |
| ZDHHC7 | 16 | q24.1 | 2.01E-05 | 6.16E-04 | 0.58 |  | 0.85 |  |
| C16orf44 | 16 | q24.1 | 1.98E-04 | 3.59E-03 | 0.52 |  | 0.77 |  |
| COX4NB | 16 | q24.1 | 3.70E-04 | 5.76E-03 | 0.50 |  | 0.81 |  |
| USP10 | 16 | q24.1 | 6.68E-04 | 8.98E-03 | 0.48 |  | 0.79 |  |
| TAF1C | 16 | q24.1 | 1.93E-03 | 1.98E-02 | 0.44 |  | 0.74 |  |
| ATP2C2 | 16 | q24.1 | 6.08E-03 | 4.49E-02 | 0.39 |  | 0.59 |  |
| ZFPM1 | 16 | q24.2 | 4.34E-05 | 1.13E-03 | 0.56 | 1.05 | 0.69 |  |
| FBXO31 | 16 | q24.2 | 8.27E-05 | 1.86E-03 | 0.54 | 1.52 | 0.81 |  |
| ZCCHC14 | 16 | q24.2 | 1.35E-04 | 2.68E-03 | 0.53 | 1.68 | 0.76 |  |
| ZC3H18 | 16 | q24.2 | 2.87E-04 | 4.76E-03 | 0.51 | 1.39 | 0.87 |  |
| AC010536.8-2 | 16 | q24.2 | 3.87E-04 | 5.97E-03 | 0.50 | 1.09 | 0.80 |  |
| MAP1LC3B | 16 | q24.2 | 4.63E-03 | 3.68E-02 | 0.41 | 0.96 | 0.80 |  |
| DEF8 | 16 | q24.3 | 1.55E-07 | 1.52E-05 | 0.68 | 1.58 | 0.81 |  |
| APRT | 16 | q24.3 | 1.75E-06 | 9.82E-05 | 0.63 | 2.14 | 0.56 |  |
| CHMP1A | 16 | q24.3 | 2.88E-06 | 1.39E-04 | 0.62 | 1.28 | 0.70 |  |
| MVD | 16 | q24.3 | 4.20E-06 | 1.88E-04 | 0.62 | 1.84 | 0.67 |  |
| RPL13 | 16 | q24.3 | 1.77E-05 | 5.58E-04 | 0.58 | 3.04 | 0.61 |  |
| CDT1 | 16 | q24.3 | 1.80E-05 | 5.64E-04 | 0.58 | 1.67 | 0.61 |  |
| DBNDD1 | 16 | q24.3 | 8.56E-05 | 1.90E-03 | 0.54 | 0.97 | 0.50 |  |
| C16orf84 | 16 | q24.3 | 1.16E-04 | 2.39E-03 | 0.53 | 1.72 | 0.82 |  |
| TCF25 | 16 | q24.3 | 2.12E-04 | 3.78E-03 | 0.52 | 1.64 | 0.73 |  |
| FAM38A | 16 | q24.3 | 1.18E-03 | 1.36E-02 | 0.46 | 1.14 | 0.78 |  |
| C16orf7 | 16 | q24.3 | 3.22E-03 | 2.84E-02 | 0.42 | 1.68 | 0.80 |  |
| CDK10 | 16 | q24.3 | 3.34E-03 | 2.92E-02 | 0.42 | 1.15 | 0.92 |  |
| TRAPPC2L | 16 | q24.3 | 3.79E-03 | 3.19E-02 | 0.41 | 0.89 | 0.71 |  |
| AFG3L1 | 16 | q24.3 | 5.97E-03 | 4.44E-02 | 0.40 | 1.52 | 0.83 |  |
| MAPK7 | 17 | p11.2 | 1.28E-06 | 7.61E-05 | 0.64 | 2.09 | 0.88 | 2.09 |
| COPS3 | 17 | p11.2 | 4.14E-06 | 1.86E-04 | 0.62 |  | 0.77 |  |
| EPN2 | 17 | p11.2 | 8.00E-05 | 1.81E-03 | 0.54 | 2.50 | 0.79 | 2.50 |
| ALDH3A2 | 17 | p11.2 | 1.25E-04 | 2.52E-03 | 0.53 | 2.06 | 0.78 | 2.80 |
| SREBF1 | 17 | p11.2 | 1.57E-04 | 2.99E-03 | 0.52 |  | 0.58 |  |
| FLII | 17 | p11.2 | 1.72E-04 | 3.21E-03 | 0.52 |  | 0.82 |  |
| ATPAF2 | 17 | p11.2 | 2.60E-04 | 4.40E-03 | 0.51 |  | 0.85 |  |
| DRG2 | 17 | p11.2 | 5.70E-04 | 8.06E-03 | 0.48 |  | 0.78 |  |
| USP22 | 17 | p11.2 | 6.13E-04 | 8.47E-03 | 0.48 | 2.32 | 0.84 |  |
| AKAP10 | 17 | p11.2 | 6.95E-04 | 9.22E-03 | 0.48 | 2.03 | 0.89 |  |
| NT5M | 17 | p11.2 | 1.36E-03 | 1.52E-02 | 0.45 |  | 0.82 |  |
| CENPV | 17 | p11.2 | 1.44E-03 | 1.57E-02 | 0.45 |  | 0.65 |  |
| TMEM11 | 17 | p11.2 | 1.99E-03 | 2.02E-02 | 0.44 | 1.74 | 0.83 |  |
| SMCR8 | 17 | p11.2 | 2.18E-03 | 2.15E-02 | 0.44 |  | 0.96 |  |
| PEMT | 17 | p11.2 | 2.37E-03 | 2.29E-02 | 0.43 |  | 0.82 |  |
| B9D1 | 17 | p11.2 | 2.54E-03 | 2.41E-02 | 0.43 | 1.43 | 0.83 | 1.43 |
| LRRC48 | 17 | p11.2 | 2.83E-03 | 2.59E-02 | 0.43 |  | 0.79 |  |
| DHRS7B | 17 | p11.2 | 3.37E-03 | 2.93E-02 | 0.42 | 1.44 | 0.83 |  |
| ALKBH5 | 17 | p11.2 | 5.35E-03 | 4.09E-02 | 0.40 |  | 0.85 |  |
| TOP3A | 17 | p11.2 | 6.06E-03 | 4.48E-02 | 0.39 |  | 0.91 |  |
| PRPSAP2 | 17 | p11.2 | 6.09E-03 | 4.50E-02 | 0.39 |  | 0.85 |  |
| RAI1 | 17 | p11.2 | 6.69E-03 | 4.80E-02 | 0.39 |  | 0.75 |  |
| MAP2K3 | 17 | p11.2 | 7.01E-03 | 4.97E-02 | 0.39 | 1.55 | 0.84 |  |
| ZNF18 | 17 | p12 | 3.26E-05 | 9.11E-04 | 0.57 |  | 0.70 |  |
| TTC19 | 17 | p12 | 1.68E-04 | 3.14E-03 | 0.52 |  | 0.71 |  |
| MAP2K4 | 17 | p12 | 4.63E-04 | 6.86E-03 | 0.49 |  | 0.61 |  |
| COX10 | 17 | p12 | 3.85E-03 | 3.23E-02 | 0.41 | 1.42 | 0.91 | 1.42 |
| ELAC2 | 17 | p12 | 4.12E-03 | 3.38E-02 | 0.41 |  | 0.74 |  |
| FAM18B2 | 17 | p12 | 7.01E-03 | 4.97E-02 | 0.39 | 0.98 | 0.71 |  |
| NCOR1 | 17 | p12-p11.2 | 8.92E-04 | 1.11E-02 | 0.47 |  | 0.80 |  |
| RNASEK | 17 | p13.1 | 3.66E-06 | 1.70E-04 | 0.62 |  | 0.76 |  |
| CHRNB1 | 17 | p13.1 | 9.06E-06 | 3.42E-04 | 0.60 |  | 0.83 |  |
| LSMD1 | 17 | p13.1 | 1.60E-05 | 5.21E-04 | 0.58 |  | 0.73 |  |
| MPDU1 | 17 | p13.1 | 1.66E-05 | 5.39E-04 | 0.58 |  | 0.63 |  |
| DVL2 | 17 | p13.1 | 1.98E-05 | 6.10E-04 | 0.58 |  | 0.79 |  |
| NDEL1 | 17 | p13.1 | 2.62E-04 | 4.44E-03 | 0.51 |  | 0.81 |  |
| TRAPPC1 | 17 | p13.1 | 2.90E-04 | 4.80E-03 | 0.51 |  | 0.83 |  |
| GPS2 | 17 | p13.1 | 6.52E-04 | 8.87E-03 | 0.48 |  | 0.73 |  |
| C17orf59 | 17 | p13.1 | 7.93E-04 | 1.02E-02 | 0.47 |  | 0.78 |  |
| EIF5A | 17 | p13.1 | 9.00E-04 | 1.11E-02 | 0.47 |  | 0.81 |  |
| RANGRF | 17 | p13.1 | 1.64E-03 | 1.74E-02 | 0.45 |  | 0.82 |  |
| TMEM107 | 17 | p13.1 | 1.73E-03 | 1.82E-02 | 0.44 |  | 0.78 |  |
| SAT2 | 17 | p13.1 | 2.19E-03 | 2.16E-02 | 0.44 |  | 0.80 |  |
| SCO1 | 17 | p13.1 | 2.19E-03 | 2.16E-02 | 0.44 | 1.88 | 0.81 |  |
| SENP3 | 17 | p13.1 | 2.27E-03 | 2.22E-02 | 0.43 |  | 0.92 |  |
| DULLARD | 17 | p13.1 | 2.30E-03 | 2.24E-02 | 0.43 |  | 0.85 |  |
| CYB5D1 | 17 | p13.1 | 3.59E-03 | 3.07E-02 | 0.42 |  | 0.76 |  |
| GABARAP | 17 | p13.1 | 4.08E-03 | 3.37E-02 | 0.41 |  | 0.82 |  |
| ACADVL | 17 | p13.1 | 4.20E-03 | 3.43E-02 | 0.41 |  | 0.73 |  |
| AC113189.5 | 17 | p13.1 | 4.88E-03 | 3.82E-02 | 0.40 |  | 0.82 |  |
| C17orf61 | 17 | p13.1 | 6.02E-03 | 4.46E-02 | 0.39 |  | 0.67 |  |
| AC135178.8 | 17 | p13.1 | 6.93E-03 | 4.93E-02 | 0.39 |  | 0.75 |  |
| TMEM93 | 17 | p13.2 | 1.21E-08 | 2.06E-06 | 0.72 | 2.36 | 0.75 |  |
| CTNS | 17 | p13.2 | 1.25E-08 | 2.11E-06 | 0.72 | 2.06 | 0.78 |  |
| ZZEF1 | 17 | p13.2 | 3.06E-08 | 4.23E-06 | 0.71 | 1.75 | 0.72 |  |
| ITGAE | 17 | p13.2 | 3.52E-07 | 2.91E-05 | 0.66 | 2.28 | 0.80 |  |
| C17orf85 | 17 | p13.2 | 8.44E-07 | 5.54E-05 | 0.65 | 1.79 | 0.78 |  |
| PELP1 | 17 | p13.2 | 1.26E-06 | 7.55E-05 | 0.64 | 1.72 | 0.65 |  |
| PSMB6 | 17 | p13.2 | 6.13E-06 | 2.51E-04 | 0.61 | 1.60 | 0.83 |  |
| SLC25A11 | 17 | p13.2 | 2.27E-05 | 6.80E-04 | 0.58 | 1.53 | 0.73 |  |
| TRPV1 | 17 | p13.2 | 5.07E-05 | 1.28E-03 | 0.56 | 1.74 | 0.79 |  |
| MYBBP1A | 17 | p13.2 | 7.87E-05 | 1.79E-03 | 0.54 | 1.57 | 0.89 |  |
| RPAIN | 17 | p13.2 | 1.29E-04 | 2.59E-03 | 0.53 | 1.79 | 0.77 |  |
| ANKFY1 | 17 | p13.2 | 1.36E-04 | 2.70E-03 | 0.53 | 1.65 | 0.82 |  |
| NUP88 | 17 | p13.2 | 1.77E-04 | 3.28E-03 | 0.52 | 2.17 | 0.83 |  |
| TAX1BP3 | 17 | p13.2 | 2.86E-04 | 4.76E-03 | 0.51 | 2.00 | 0.77 |  |
| DERL2 | 17 | p13.2 | 3.90E-04 | 6.01E-03 | 0.50 | 1.64 | 0.81 |  |
| AC087742.9 | 17 | p13.2 | 4.78E-04 | 7.04E-03 | 0.49 | 1.42 | 0.85 |  |
| RNF167 | 17 | p13.2 | 5.53E-04 | 7.85E-03 | 0.48 | 1.34 | 0.79 |  |
| C1QBP | 17 | p13.2 | 7.83E-04 | 1.01E-02 | 0.47 | 1.20 | 0.82 |  |
| OR1E2 | 17 | p13.2 | 9.10E-04 | 1.12E-02 | 0.47 | 1.11 | 0.96 | 1.22 |
| SPAG7 | 17 | p13.2 | 1.20E-03 | 1.38E-02 | 0.46 | 2.09 | 0.78 |  |
| MED11 | 17 | p13.2 | 1.57E-03 | 1.68E-02 | 0.45 | 1.39 | 0.87 |  |
| CXCL16 | 17 | p13.2 | 2.18E-03 | 2.16E-02 | 0.44 | 1.46 | 0.76 |  |
| KIF1C | 17 | p13.2 | 3.19E-03 | 2.83E-02 | 0.42 | 1.47 | 0.89 |  |
| CAMTA2 | 17 | p13.2 | 3.89E-03 | 3.26E-02 | 0.41 | 1.24 | 0.93 |  |
| DHX33 | 17 | p13.2 | 4.45E-03 | 3.58E-02 | 0.41 | 1.62 | 0.84 |  |
| KIAA0753 | 17 | p13.2-p13.1 | 4.38E-03 | 3.54E-02 | 0.41 |  | 0.82 |  |
| PRPF8 | 17 | p13.3 | 1.19E-10 | 4.98E-08 | 0.78 | 2.30 | 0.72 |  |
| PITPNA | 17 | p13.3 | 2.02E-07 | 1.88E-05 | 0.67 | 1.44 | 0.84 |  |
| GARNL4 | 17 | p13.3 | 1.57E-05 | 5.17E-04 | 0.58 | 1.87 | 0.88 |  |
| RNMTL1 | 17 | p13.3 | 3.56E-05 | 9.72E-04 | 0.56 | 2.51 | 0.71 |  |
| KIAA0664 | 17 | p13.3 | 3.68E-05 | 9.93E-04 | 0.56 | 2.26 | 0.71 |  |
| YWHAE | 17 | p13.3 | 6.63E-05 | 1.56E-03 | 0.55 | 1.59 | 0.83 |  |
| MYO1C | 17 | p13.3 | 9.25E-05 | 2.01E-03 | 0.54 | 2.45 | 0.70 |  |
| PAFAH1B1 | 17 | p13.3 | 1.43E-04 | 2.79E-03 | 0.53 | 1.66 | 0.90 |  |
| CRK | 17 | p13.3 | 2.51E-04 | 4.30E-03 | 0.51 | 1.71 | 0.79 |  |
| METT10D | 17 | p13.3 | 3.37E-04 | 5.39E-03 | 0.50 | 1.68 | 0.79 |  |
| TSR1 | 17 | p13.3 | 4.62E-04 | 6.86E-03 | 0.49 | 2.01 | 0.86 |  |
| TIMM22 | 17 | p13.3 | 6.65E-04 | 8.95E-03 | 0.48 | 1.36 | 0.89 |  |
| SGSM2 | 17 | p13.3 | 1.45E-03 | 1.58E-02 | 0.45 | 2.24 | 0.76 |  |
| FAM57A | 17 | p13.3 | 2.22E-03 | 2.18E-02 | 0.44 | 2.70 | 0.85 |  |
| GEMIN4 | 17 | p13.3 | 2.39E-03 | 2.30E-02 | 0.43 | 1.60 | 0.88 |  |
| GLOD4 | 17 | p13.3 | 3.67E-03 | 3.12E-02 | 0.42 | 1.54 | 0.76 |  |
| ABR | 17 | p13.3 | 4.21E-03 | 3.44E-02 | 0.41 | 1.73 | 0.86 |  |
| C17orf97 | 17 | p13.3 | 6.75E-03 | 4.84E-02 | 0.39 | 1.29 | 0.66 |  |
| WSB1 | 17 | q11.1 | 5.89E-08 | 6.96E-06 | 0.70 | 1.53 | 0.91 | 1.84 |
| TNFAIP1 | 17 | q11.2 | 4.44E-16 | 2.11E-12 | 0.88 | 2.20 | 0.75 | 3.90 |
| KIAA0100 | 17 | q11.2 | 1.78E-15 | 6.32E-12 | 0.87 | 1.97 | 0.73 | 3.11 |
| FLOT2 | 17 | q11.2 | 3.11E-15 | 8.85E-12 | 0.87 | 2.24 | 0.78 | 3.96 |
| C17orf63 | 17 | q11.2 | 1.07E-14 | 2.02E-11 | 0.86 | 2.12 | 0.75 | 3.00 |
| ERAL1 | 17 | q11.2 | 9.77E-15 | 2.02E-11 | 0.86 | 2.37 | 0.67 | 3.91 |
| PHF12 | 17 | q11.2 | 1.55E-14 | 2.77E-11 | 0.86 | 2.31 | 0.64 | 3.31 |
| DHRS13 | 17 | q11.2 | 2.44E-14 | 4.09E-11 | 0.85 | 2.65 | 0.69 | 4.69 |
| SUPT6H | 17 | q11.2 | 3.11E-14 | 4.92E-11 | 0.85 | 1.97 | 0.80 | 2.97 |
| TRAF4 | 17 | q11.2 | 1.15E-13 | 1.57E-10 | 0.84 | 2.85 | 0.78 | 4.35 |
| IFT20 | 17 | q11.2 | 3.44E-12 | 2.80E-09 | 0.81 | 1.94 | 0.75 | 2.83 |
| SDF2 | 17 | q11.2 | 2.19E-11 | 1.22E-08 | 0.80 | 2.14 | 0.66 | 2.50 |
| TLCD1 | 17 | q11.2 | 3.25E-11 | 1.60E-08 | 0.79 | 2.88 | 0.76 | 4.04 |
| snoZ17 | 17 | q11.2 | 2.70E-10 | 9.59E-08 | 0.77 | 1.79 | 0.81 | 2.09 |
| NEK8 | 17 | q11.2 | 6.14E-10 | 1.82E-07 | 0.76 | 2.05 | 0.67 | 2.75 |
| SPAG5 | 17 | q11.2 | 8.05E-10 | 2.25E-07 | 0.76 | 2.42 | 0.69 | 3.77 |
| C17orf32 | 17 | q11.2 | 8.30E-10 | 2.27E-07 | 0.76 | 1.89 | 0.72 | 2.79 |
| PIGS | 17 | q11.2 | 1.06E-09 | 2.80E-07 | 0.75 | 1.88 | 0.73 | 3.06 |
| TP53I13 | 17 | q11.2 | 1.43E-09 | 3.60E-07 | 0.75 | 2.18 | 0.77 | 3.03 |
| GIT1 | 17 | q11.2 | 1.76E-09 | 4.25E-07 | 0.75 | 2.11 | 0.87 | 3.82 |
| POLDIP2 | 17 | q11.2 | 9.44E-09 | 1.66E-06 | 0.72 | 1.69 | 0.80 | 2.22 |
| GOSR1 | 17 | q11.2 | 2.15E-08 | 3.18E-06 | 0.71 | 1.41 | 0.80 | 1.65 |
| UNC119 | 17 | q11.2 | 4.81E-08 | 5.91E-06 | 0.70 | 1.52 | 0.80 | 2.22 |
| MYO18A | 17 | q11.2 | 1.96E-07 | 1.83E-05 | 0.67 | 1.68 | 0.77 | 2.83 |
| SSH2 | 17 | q11.2 | 2.83E-06 | 1.38E-04 | 0.62 | 1.67 | 0.97 | 2.10 |
| NUFIP2 | 17 | q11.2 | 3.92E-06 | 1.79E-04 | 0.62 | 1.99 | 0.70 | 2.17 |
| MYO1D | 17 | q11.2 | 6.29E-06 | 2.57E-04 | 0.61 | 1.49 | 0.74 | 2.61 |
| AC130289.2 | 17 | q11.2 | 1.32E-05 | 4.52E-04 | 0.59 | 1.53 | 0.73 | 1.96 |
| CCDC55 | 17 | q11.2 | 1.87E-05 | 5.83E-04 | 0.58 | 1.87 | 0.70 | 1.61 |
| C17orf79 | 17 | q11.2 | 3.24E-05 | 9.05E-04 | 0.57 | 2.68 | 0.78 |  |
| RPL23A | 17 | q11.2 | 3.29E-05 | 9.16E-04 | 0.57 | 2.01 | 0.76 | 2.25 |
| AC005726.6 | 17 | q11.2 | 6.50E-05 | 1.54E-03 | 0.55 | 1.07 | 1.01 | 1.16 |
| RHOT1 | 17 | q11.2 | 8.70E-05 | 1.92E-03 | 0.54 | 1.65 | 0.67 |  |
| PSMD11 | 17 | q11.2 | 8.84E-05 | 1.94E-03 | 0.54 | 1.46 | 0.89 |  |
| NF1 | 17 | q11.2 | 1.41E-04 | 2.77E-03 | 0.53 | 1.19 | 0.93 | 1.28 |
| ALDOC | 17 | q11.2 | 5.03E-04 | 7.32E-03 | 0.49 | 1.81 | 1.05 | 3.78 |
| ANKRD13B | 17 | q11.2 | 6.12E-04 | 8.46E-03 | 0.48 | 1.13 | 0.92 | 1.38 |
| CDK5R1 | 17 | q11.2 | 1.05E-03 | 1.26E-02 | 0.46 | 1.30 | 0.86 |  |
| UTP6 | 17 | q11.2 | 1.19E-03 | 1.38E-02 | 0.46 | 1.66 | 0.73 |  |
| ZNF207 | 17 | q11.2 | 1.40E-03 | 1.54E-02 | 0.45 | 1.38 | 0.72 |  |
| NLK | 17 | q11.2 | 2.05E-03 | 2.06E-02 | 0.44 | 1.41 | 0.74 | 1.90 |
| RNF135 | 17 | q11.2 | 2.08E-03 | 2.08E-02 | 0.44 | 1.99 | 0.91 |  |
| TMEM98 | 17 | q11.2 | 2.13E-03 | 2.12E-02 | 0.44 | 1.29 | 0.43 | 2.67 |
| SNORD42 | 17 | q11.2 | 2.34E-03 | 2.26E-02 | 0.43 | 1.07 | 0.94 | 1.10 |
| TAOK1 | 17 | q11.2 | 2.86E-03 | 2.62E-02 | 0.43 | 1.65 | 0.84 | 2.04 |
| ABHD15 | 17 | q11.2 | 3.46E-03 | 2.99E-02 | 0.42 | 1.43 | 0.82 | 1.35 |
| SUZ12 | 17 | q11.2 | 3.72E-03 | 3.16E-02 | 0.42 | 1.25 | 0.70 |  |
| CCDC49 | 17 | q12 | 0.00E+00 | 0.00E+00 | 0.93 | 2.49 | 0.73 | 5.84 |
| PSMB3 | 17 | q12 | 1.30E-13 | 1.68E-10 | 0.84 | 2.76 | 0.72 | 4.42 |
| PIP5K2B | 17 | q12 | 3.03E-12 | 2.53E-09 | 0.82 | 1.48 | 0.83 | 2.73 |
| PCGF2 | 17 | q12 | 6.02E-09 | 1.21E-06 | 0.73 | 2.16 | 0.58 | 5.50 |
| C17orf37 | 17 | q12 | 1.17E-08 | 2.00E-06 | 0.72 | 3.39 | 0.59 | 4.76 |
| TADA2L | 17 | q12 | 7.15E-08 | 8.08E-06 | 0.69 | 1.30 | 0.90 | 2.12 |
| PERLD1 | 17 | q12 | 1.83E-07 | 1.74E-05 | 0.68 | 4.19 | 0.51 | 5.50 |
| DUSP14 | 17 | q12 | 4.03E-07 | 3.17E-05 | 0.66 | 1.85 | 0.76 | 2.59 |
| TCAP | 17 | q12 | 4.59E-07 | 3.52E-05 | 0.66 | 1.75 | 0.83 | 2.16 |
| ERBB2 | 17 | q12 | 4.95E-07 | 3.75E-05 | 0.66 | 2.49 | 0.70 | 2.92 |
| GSDMB | 17 | q12 | 6.53E-07 | 4.54E-05 | 0.65 | 4.08 | 0.74 | 6.28 |
| TBC1D3C | 17 | q12 | 8.07E-07 | 5.36E-05 | 0.65 | 1.54 | 0.85 | 2.32 |
| MRPL45 | 17 | q12 | 9.74E-07 | 6.21E-05 | 0.65 | 1.79 | 0.69 | 2.87 |
| GRB7 | 17 | q12 | 1.24E-06 | 7.44E-05 | 0.64 | 2.42 | 0.71 | 3.44 |
| MLLT6 | 17 | q12 | 1.59E-06 | 9.13E-05 | 0.64 | 2.63 | 0.71 | 2.64 |
| STARD3 | 17 | q12 | 1.74E-06 | 9.79E-05 | 0.63 | 3.03 | 0.72 | 4.17 |
| DDX52 | 17 | q12 | 1.83E-06 | 1.01E-04 | 0.63 | 1.19 | 0.92 | 1.24 |
| ZNF403 | 17 | q12 | 2.31E-06 | 1.18E-04 | 0.63 | 1.52 | 0.71 | 4.25 |
| DHRS11 | 17 | q12 | 6.48E-06 | 2.62E-04 | 0.61 | 1.59 | 0.89 | 6.74 |
| LASP1 | 17 | q12 | 1.20E-05 | 4.21E-04 | 0.59 | 2.27 | 0.62 | 3.10 |
| TBC1D3 | 17 | q12 | 1.35E-05 | 4.59E-04 | 0.59 | 1.31 | 0.91 | 1.89 |
| AATF | 17 | q12 | 2.48E-05 | 7.28E-04 | 0.57 | 1.51 | 0.92 | 3.19 |
| ORMDL3 | 17 | q12 | 2.80E-05 | 8.05E-04 | 0.57 | 3.59 | 0.63 | 4.70 |
| RPL23 | 17 | q12 | 3.56E-05 | 9.72E-04 | 0.56 | 2.19 | 0.50 | 3.36 |
| PPARBP | 17 | q12 | 3.93E-05 | 1.05E-03 | 0.56 | 2.52 | 0.59 | 2.71 |
| ACACA | 17 | q12 | 5.11E-05 | 1.28E-03 | 0.56 | 2.07 | 0.70 | 3.03 |
| ZNHIT3 | 17 | q12 | 8.69E-05 | 1.92E-03 | 0.54 | 1.49 | 0.73 | 2.42 |
| AP1GBP1 | 17 | q12 | 1.25E-04 | 2.52E-03 | 0.53 | 1.10 | 0.95 | 1.67 |
| FBXL20 | 17 | q12 | 2.38E-04 | 4.13E-03 | 0.51 | 3.22 | 0.64 | 3.67 |
| SOCS7 | 17 | q12 | 4.26E-04 | 6.44E-03 | 0.49 | 1.22 | 0.92 | 1.49 |
| PNMT | 17 | q12 | 4.61E-04 | 6.85E-03 | 0.49 | 2.37 | 0.75 | 2.68 |
| AC183087.2-1 | 17 | q12 | 9.65E-04 | 1.17E-02 | 0.47 | 1.25 | 0.98 | 1.34 |
| LIG3 | 17 | q12 | 2.31E-03 | 2.24E-02 | 0.43 | 1.51 | 0.83 |  |
| PPP1R1B | 17 | q12 | 2.39E-03 | 2.30E-02 | 0.43 | 1.40 | 0.94 | 1.64 |
| RAD51L3 | 17 | q12 | 3.21E-03 | 2.84E-02 | 0.42 | 1.26 | 0.92 |  |
| MYO19 | 17 | q12 | 3.77E-03 | 3.19E-02 | 0.41 | 1.19 | 0.98 | 1.54 |
| AC115090.8 | 17 | q12 | 4.77E-03 | 3.75E-02 | 0.40 | 1.13 | 0.99 | 1.14 |
| ARHGAP23 | 17 | q12 | 5.16E-03 | 3.98E-02 | 0.40 | 1.37 | 0.79 | 1.54 |
| PIGW | 17 | q12 | 5.71E-03 | 4.29E-02 | 0.40 | 1.30 | 0.92 | 1.60 |
| TBC1D3F | 17 | q12 | 5.84E-03 | 4.37E-02 | 0.40 | 1.07 | 0.98 | 1.18 |
| THRAP4 | 17 | q21.1 | 1.18E-07 | 1.23E-05 | 0.68 | 2.96 | 0.72 | 4.13 |
| PSMD3 | 17 | q21.1 | 1.31E-07 | 1.33E-05 | 0.68 | 3.35 | 0.64 | 5.22 |
| CASC3 | 17 | q21.1 | 4.42E-06 | 1.95E-04 | 0.61 | 2.33 | 0.67 | 2.70 |
| THRA | 17 | q21.1 | 1.22E-05 | 4.27E-04 | 0.59 | 2.00 | 0.74 | 3.56 |
| RAPGEFL1 | 17 | q21.1 | 1.01E-04 | 2.15E-03 | 0.54 | 2.10 | 0.67 | 2.23 |
| WIPF2 | 17 | q21.1-q21.2 | 3.93E-06 | 1.79E-04 | 0.62 | 1.80 | 0.78 | 2.00 |
| RARA | 17 | q21.2 | 4.14E-06 | 1.86E-04 | 0.62 | 2.13 | 0.67 | 3.58 |
| SMARCE1 | 17 | q21.2 | 1.25E-05 | 4.35E-04 | 0.59 | 1.95 | 0.67 |  |
| CDC6 | 17 | q21.2 | 1.45E-05 | 4.86E-04 | 0.59 | 1.19 | 0.98 | 1.35 |
| GHDC | 17 | q21.2 | 8.25E-05 | 1.85E-03 | 0.54 | 1.21 | 0.71 |  |
| AC130686.6 | 17 | q21.2 | 9.04E-05 | 1.98E-03 | 0.54 | 1.64 | 0.70 |  |
| DNAJC7 | 17 | q21.2 | 1.57E-04 | 2.99E-03 | 0.52 | 1.39 | 0.81 |  |
| TUBG1 | 17 | q21.2 | 7.15E-04 | 9.42E-03 | 0.48 | 1.76 | 0.72 |  |
| JUP | 17 | q21.2 | 9.31E-04 | 1.14E-02 | 0.47 | 1.59 | 0.72 |  |
| KRT10 | 17 | q21.2 | 1.52E-03 | 1.64E-02 | 0.45 | 1.78 | 0.64 |  |
| TMEM99 | 17 | q21.2 | 1.91E-03 | 1.96E-02 | 0.44 | 2.29 | 0.69 |  |
| AC003958.1 | 17 | q21.2 | 2.23E-03 | 2.19E-02 | 0.44 | 1.70 | 0.87 |  |
| TUBG2 | 17 | q21.2 | 2.51E-03 | 2.38E-02 | 0.43 | 1.96 | 0.73 |  |
| EIF1 | 17 | q21.2 | 2.91E-03 | 2.65E-02 | 0.42 | 1.38 | 0.82 |  |
| KRT31 | 17 | q21.2 | 3.23E-03 | 2.85E-02 | 0.42 | 1.85 | 0.86 |  |
| NT5C3L | 17 | q21.2 | 5.43E-03 | 4.14E-02 | 0.40 | 1.47 | 0.76 |  |
| TTC25 | 17 | q21.2 | 5.47E-03 | 4.16E-02 | 0.40 | 1.60 | 0.76 |  |
| NSF | 17 | q21.31 | 1.15E-11 | 7.64E-09 | 0.80 | 1.63 | 0.71 | 6.49 |
| WNT3 | 17 | q21.31 | 6.20E-07 | 4.36E-05 | 0.65 | 1.48 | 0.79 | 3.54 |
| CCDC43 | 17 | q21.31 | 4.29E-06 | 1.91E-04 | 0.61 | 1.90 | 0.77 |  |
| BECN1 | 17 | q21.31 | 2.13E-05 | 6.46E-04 | 0.58 | 2.70 | 0.73 |  |
| G6PC3 | 17 | q21.31 | 4.03E-05 | 1.07E-03 | 0.56 | 1.56 | 0.69 |  |
| RUNDC1 | 17 | q21.31 | 9.86E-05 | 2.11E-03 | 0.54 | 2.61 | 0.66 |  |
| CCDC103 | 17 | q21.31 | 3.04E-04 | 4.97E-03 | 0.50 | 1.70 | 0.83 |  |
| PSME3 | 17 | q21.31 | 6.61E-04 | 8.92E-03 | 0.48 | 2.13 | 0.75 |  |
| VPS25 | 17 | q21.31 | 1.21E-03 | 1.38E-02 | 0.46 | 1.70 | 0.78 |  |
| AC126544.2 | 17 | q21.31 | 1.76E-03 | 1.85E-02 | 0.44 | 1.54 | 0.72 |  |
| RPL27 | 17 | q21.31 | 3.32E-03 | 2.91E-02 | 0.42 | 1.17 | 0.89 |  |
| PLCD3 | 17 | q21.31 | 4.67E-03 | 3.70E-02 | 0.41 | 0.97 | 0.82 |  |
| TMUB2 | 17 | q21.31 | 4.90E-03 | 3.83E-02 | 0.40 | 1.47 | 0.84 |  |
| LSM12 | 17 | q21.31 | 6.39E-03 | 4.66E-02 | 0.39 | 1.93 | 0.92 |  |
| DCAKD | 17 | q21.31 | 6.82E-03 | 4.88E-02 | 0.39 | 1.39 | 0.78 |  |
| CNTD1 | 17 | q21.31 | 7.05E-03 | 4.99E-02 | 0.39 | 1.33 | 0.90 |  |
| GOSR2 | 17 | q21.32 | 2.22E-15 | 7.03E-12 | 0.87 | 1.56 | 0.87 | 3.83 |
| UBE2Z | 17 | q21.32 | 8.26E-08 | 9.16E-06 | 0.69 | 1.70 | 0.77 | 1.98 |
| CALCOCO2 | 17 | q21.32 | 8.74E-05 | 1.93E-03 | 0.54 | 1.66 | 0.80 | 1.83 |
| SNF8 | 17 | q21.32 | 1.54E-04 | 2.94E-03 | 0.52 | 1.91 | 0.76 | 1.89 |
| SNX11 | 17 | q21.32 | 4.61E-04 | 6.85E-03 | 0.49 | 1.41 | 0.84 | 1.18 |
| PNPO | 17 | q21.32 | 5.98E-04 | 8.31E-03 | 0.48 | 1.84 | 0.62 | 1.87 |
| ATAD4 | 17 | q21.32 | 4.97E-03 | 3.86E-02 | 0.40 | 1.66 | 0.63 | 1.82 |
| ATP5G1 | 17 | q21.32 | 6.01E-03 | 4.45E-02 | 0.39 | 1.52 | 0.76 | 0.95 |
| PHB | 17 | q21.33 | 6.38E-09 | 1.25E-06 | 0.73 | 2.09 | 0.72 | 2.67 |
| NME1-NME2 | 17 | q21.33 | 3.87E-07 | 3.11E-05 | 0.66 | 1.88 | 0.64 | 2.27 |
| SLC35B1 | 17 | q21.33 | 9.04E-07 | 5.86E-05 | 0.65 | 1.65 | 0.78 | 2.02 |
| EPN3 | 17 | q21.33 | 1.36E-06 | 8.02E-05 | 0.64 | 1.97 | 0.78 | 3.30 |
| EME1 | 17 | q21.33 | 1.55E-05 | 5.14E-04 | 0.59 | 1.35 | 0.90 | 1.47 |
| SPATA20 | 17 | q21.33 | 1.69E-05 | 5.44E-04 | 0.58 | 1.97 | 0.75 | 2.89 |
| TOB1 | 17 | q21.33 | 6.40E-05 | 1.52E-03 | 0.55 | 2.22 | 0.61 | 1.98 |
| LRRC59 | 17 | q21.33 | 6.91E-05 | 1.61E-03 | 0.55 | 1.22 | 0.92 | 1.39 |
| AC005921.3-2 | 17 | q21.33 | 8.81E-05 | 1.94E-03 | 0.54 | 1.66 | 0.67 | 1.96 |
| ANKRD40 | 17 | q21.33 | 1.87E-04 | 3.43E-03 | 0.52 | 1.33 | 0.89 | 1.36 |
| XYLT2 | 17 | q21.33 | 3.22E-03 | 2.84E-02 | 0.42 | 1.48 | 0.75 | 1.27 |
| MRPL27 | 17 | q21.33 | 3.57E-03 | 3.06E-02 | 0.42 | 1.23 | 0.96 | 1.31 |
| MYCBPAP | 17 | q21.33 | 4.08E-03 | 3.37E-02 | 0.41 | 1.07 | 0.93 | 1.10 |
| SUPT4H1 | 17 | q22 | 5.74E-10 | 1.75E-07 | 0.76 | 1.80 | 0.73 | 2.06 |
| TRIM37 | 17 | q22 | 1.19E-09 | 3.09E-07 | 0.75 | 1.88 | 0.58 | 2.82 |
| C17orf71 | 17 | q22 | 3.30E-09 | 6.90E-07 | 0.74 | 1.32 | 0.88 | 1.83 |
| RNF43 | 17 | q22 | 1.59E-08 | 2.54E-06 | 0.72 | 2.03 | 0.69 | 2.90 |
| MRPS23 | 17 | q22 | 8.58E-08 | 9.42E-06 | 0.69 | 1.65 | 0.73 | 1.84 |
| STXBP4 | 17 | q22 | 3.69E-07 | 3.02E-05 | 0.66 | 1.20 | 0.79 | 1.40 |
| PRR11 | 17 | q22 | 3.89E-07 | 3.11E-05 | 0.66 | 1.59 | 0.92 | 2.05 |
| COIL | 17 | q22 | 1.01E-06 | 6.41E-05 | 0.64 | 1.55 | 0.73 | 2.15 |
| TOM1L1 | 17 | q22 | 1.11E-06 | 6.80E-05 | 0.64 | 1.46 | 0.72 | 2.61 |
| GDPD1 | 17 | q22 | 2.15E-06 | 1.12E-04 | 0.63 | 1.25 | 0.86 | 1.34 |
| RAD51C | 17 | q22 | 3.45E-06 | 1.61E-04 | 0.62 | 1.59 | 0.75 | 2.59 |
| MTMR4 | 17 | q22 | 7.16E-06 | 2.86E-04 | 0.60 | 1.54 | 0.68 | 1.79 |
| FAM33A | 17 | q22 | 9.07E-06 | 3.42E-04 | 0.60 | 1.24 | 0.94 | 1.76 |
| MKS1 | 17 | q22 | 9.94E-06 | 3.67E-04 | 0.60 | 1.18 | 0.95 | 1.27 |
| DYNLL2 | 17 | q22 | 1.00E-05 | 3.67E-04 | 0.60 | 1.65 | 0.88 | 1.56 |
| MSI2 | 17 | q22 | 6.36E-05 | 1.52E-03 | 0.55 | 1.63 | 0.67 | 1.54 |
| AKAP1 | 17 | q22 | 7.91E-05 | 1.80E-03 | 0.54 | 1.21 | 0.82 | 1.53 |
| YPEL2 | 17 | q22 | 1.50E-04 | 2.89E-03 | 0.53 | 1.29 | 0.75 | 1.76 |
| SFRS1 | 17 | q22 | 1.73E-04 | 3.21E-03 | 0.52 | 1.18 | 0.92 | 1.37 |
| PCTP | 17 | q22 | 2.42E-04 | 4.17E-03 | 0.51 | 1.18 | 0.88 | 1.70 |
| COX11 | 17 | q22 | 3.58E-04 | 5.63E-03 | 0.50 | 1.13 | 0.85 | 1.31 |
| BZRAP1 | 17 | q22 | 4.87E-04 | 7.15E-03 | 0.49 | 1.21 | 0.83 | 1.65 |
| CUEDC1 | 17 | q22 | 6.34E-04 | 8.69E-03 | 0.48 | 1.59 | 0.73 | 1.70 |
| TMEM49 | 17 | q23.1 | 2.27E-09 | 5.18E-07 | 0.74 | 1.77 | 0.82 | 1.96 |
| PTRH2 | 17 | q23.1 | 1.27E-08 | 2.13E-06 | 0.72 | 2.19 | 0.69 | 2.40 |
| RPS6KB1 | 17 | q23.1 | 1.12E-05 | 4.00E-04 | 0.59 | 2.11 | 0.72 | 3.00 |
| DHX40 | 17 | q23.1 | 3.18E-05 | 8.93E-04 | 0.57 | 1.22 | 0.93 | 1.40 |
| TUBD1 | 17 | q23.1 | 1.04E-04 | 2.20E-03 | 0.54 | 1.52 | 0.83 | 1.53 |
| CLTC | 17 | q23.1 | 1.22E-04 | 2.48E-03 | 0.53 | 1.37 | 0.84 | 1.59 |
| HEATR6 | 17 | q23.1 | 1.81E-03 | 1.88E-02 | 0.44 | 1.78 | 0.81 | 2.14 |
| USP32 | 17 | q23.1-q23.2 | 3.69E-07 | 3.02E-05 | 0.66 | 1.27 | 0.89 | 1.50 |
| METTL2A | 17 | q23.2 | 1.88E-13 | 2.14E-10 | 0.84 | 1.60 | 0.78 | 2.24 |
| TLK2 | 17 | q23.2 | 9.35E-12 | 6.65E-09 | 0.80 | 1.42 | 0.86 | 1.95 |
| PPM1D | 17 | q23.2 | 1.22E-06 | 7.33E-05 | 0.64 | 1.56 | 0.75 | 2.40 |
| INTS2 | 17 | q23.2 | 2.26E-06 | 1.16E-04 | 0.63 | 1.55 | 0.68 | 1.82 |
| BCAS3 | 17 | q23.2 | 1.09E-05 | 3.92E-04 | 0.59 | 1.19 | 0.95 | 1.23 |
| APPBP2 | 17 | q23.2 | 4.34E-04 | 6.54E-03 | 0.49 | 1.13 | 0.93 | 1.34 |
| C17orf82 | 17 | q23.2 | 6.09E-04 | 8.44E-03 | 0.48 | 1.09 | 0.96 | 1.12 |
| MED13 | 17 | q23.2 | 1.19E-03 | 1.37E-02 | 0.46 | 1.09 | 1.00 | 1.19 |
| TANC2 | 17 | q23.2-q23.3 | 3.84E-03 | 3.22E-02 | 0.41 | 1.08 | 0.94 | 1.13 |
| POLG2 | 17 | q23.3 | 9.86E-12 | 6.85E-09 | 0.80 | 1.46 | 0.78 | 2.02 |
| CYB561 | 17 | q23.3 | 1.69E-11 | 1.01E-08 | 0.80 | 2.30 | 0.75 | 3.00 |
| DDX42 | 17 | q23.3 | 2.39E-11 | 1.28E-08 | 0.80 | 1.49 | 0.89 | 1.99 |
| CCDC47 | 17 | q23.3 | 7.28E-11 | 3.19E-08 | 0.78 | 1.49 | 0.98 | 2.03 |
| PSMC5 | 17 | q23.3 | 1.91E-10 | 7.16E-08 | 0.77 | 1.68 | 0.75 | 2.19 |
| CCDC45 | 17 | q23.3 | 4.28E-10 | 1.40E-07 | 0.76 | 1.71 | 0.71 | 2.59 |
| SMARCD2 | 17 | q23.3 | 6.76E-10 | 1.98E-07 | 0.76 | 2.02 | 0.82 | 2.44 |
| TEX2 | 17 | q23.3 | 1.01E-09 | 2.68E-07 | 0.75 | 1.70 | 0.75 | 2.69 |
| FTSJ3 | 17 | q23.3 | 1.28E-09 | 3.26E-07 | 0.75 | 1.74 | 0.86 | 2.55 |
| CCDC44 | 17 | q23.3 | 2.68E-08 | 3.81E-06 | 0.71 | 2.18 | 0.76 | 2.49 |
| ERN1 | 17 | q23.3 | 3.42E-08 | 4.57E-06 | 0.70 | 1.50 | 0.91 | 2.07 |
| WDR68 | 17 | q23.3 | 4.70E-08 | 5.82E-06 | 0.70 | 1.83 | 0.73 | 1.98 |
| DDX5 | 17 | q23.3 | 1.69E-07 | 1.64E-05 | 0.68 | 1.49 | 0.68 | 2.44 |
| STRADA | 17 | q23.3 | 1.11E-06 | 6.80E-05 | 0.64 | 1.49 | 0.80 | 1.82 |
| MAP3K3 | 17 | q23.3 | 1.76E-04 | 3.26E-03 | 0.52 | 1.25 | 1.05 | 1.34 |
| AC132812.9 | 17 | q24.1 | 5.02E-08 | 6.13E-06 | 0.70 | 1.76 | 0.90 | 3.07 |
| GNA13 | 17 | q24.1 | 1.09E-05 | 3.92E-04 | 0.59 | 1.50 | 0.90 | 3.27 |
| AC103810.1 | 17 | q24.1 | 3.91E-03 | 3.27E-02 | 0.41 | 1.37 | 0.69 | 1.03 |
| AXIN2 | 17 | q24.1 | 5.50E-03 | 4.17E-02 | 0.40 | 1.21 | 0.78 | 1.73 |
| NOL11 | 17 | q24.2 | 4.84E-09 | 9.78E-07 | 0.73 | 1.59 | 0.77 | 2.27 |
| HELZ | 17 | q24.2 | 3.40E-08 | 4.57E-06 | 0.70 | 1.38 | 0.89 | 2.13 |
| CACNG4 | 17 | q24.2 | 2.03E-06 | 1.08E-04 | 0.63 | 2.35 | 0.59 | 4.95 |
| KPNA2 | 17 | q24.2 | 3.81E-06 | 1.75E-04 | 0.62 | 1.67 | 0.90 | 3.30 |
| PRKAR1A | 17 | q24.2 | 9.31E-06 | 3.49E-04 | 0.60 | 1.44 | 0.74 | 3.30 |
| FALZ | 17 | q24.2 | 1.05E-05 | 3.82E-04 | 0.59 | 1.32 | 0.85 | 1.47 |
| C17orf58 | 17 | q24.2 | 8.30E-05 | 1.86E-03 | 0.54 | 1.16 | 0.98 | 1.61 |
| CACNG1 | 17 | q24.2 | 2.34E-03 | 2.27E-02 | 0.43 | 1.51 | 0.90 | 1.97 |
| SLC16A6 | 17 | q24.2 | 3.09E-03 | 2.76E-02 | 0.42 | 1.14 | 0.75 | 2.91 |
| ARSG | 17 | q24.2 | 6.08E-03 | 4.49E-02 | 0.39 | 1.13 | 0.74 | 1.70 |
| ABCA9 | 17 | q24.2 | 6.50E-03 | 4.71E-02 | -0.39 | 0.92 | 1.06 | 0.82 |
| ABCA5 | 17 | q24.3 | 9.46E-07 | 6.08E-05 | 0.65 | 1.17 | 0.85 | 1.88 |
| SRP68 | 17 | q25.1 | 1.76E-11 | 1.01E-08 | 0.80 | 1.66 | 0.83 | 2.85 |
| SAP30BP | 17 | q25.1 | 6.51E-09 | 1.27E-06 | 0.73 | 1.74 | 0.92 | 3.17 |
| GGA3 | 17 | q25.1 | 6.74E-09 | 1.30E-06 | 0.73 | 1.42 | 0.85 | 2.51 |
| NUP85 | 17 | q25.1 | 9.38E-09 | 1.66E-06 | 0.72 | 1.73 | 0.84 | 3.78 |
| NT5C | 17 | q25.1 | 1.89E-08 | 2.89E-06 | 0.71 | 1.57 | 0.76 | 2.67 |
| MRPS7 | 17 | q25.1 | 1.90E-08 | 2.89E-06 | 0.71 | 2.09 | 0.71 | 3.16 |
| ICT1 | 17 | q25.1 | 6.36E-08 | 7.36E-06 | 0.69 | 1.73 | 0.77 |  |
| MRPL38 | 17 | q25.1 | 7.32E-08 | 8.24E-06 | 0.69 | 2.05 | 0.76 | 3.35 |
| ACOX1 | 17 | q25.1 | 7.39E-08 | 8.29E-06 | 0.69 | 1.47 | 0.87 | 4.09 |
| TSEN54 | 17 | q25.1 | 1.54E-07 | 1.51E-05 | 0.68 | 1.66 | 0.79 | 2.59 |
| RNF157 | 17 | q25.1 | 3.44E-07 | 2.84E-05 | 0.66 | 1.92 | 0.98 | 10.83 |
| KIAA0195 | 17 | q25.1 | 5.14E-07 | 3.88E-05 | 0.66 | 1.86 | 0.79 | 4.14 |
| RECQL5 | 17 | q25.1 | 7.84E-06 | 3.07E-04 | 0.60 | 1.80 | 0.81 | 2.50 |
| ATP5H | 17 | q25.1 | 1.12E-05 | 4.00E-04 | 0.59 | 1.60 | 0.78 |  |
| UBE2O | 17 | q25.1 | 2.00E-05 | 6.12E-04 | 0.58 | 1.64 | 0.88 |  |
| C17orf95 | 17 | q25.1 | 2.87E-05 | 8.20E-04 | 0.57 | 1.63 | 0.76 |  |
| RPL38 | 17 | q25.1 | 3.39E-05 | 9.37E-04 | 0.57 | 2.01 | 0.61 |  |
| GRB2 | 17 | q25.1 | 4.25E-05 | 1.11E-03 | 0.56 | 1.66 | 0.86 | 2.94 |
| FDXR | 17 | q25.1 | 1.11E-04 | 2.31E-03 | 0.53 | 2.02 | 0.71 |  |
| WBP2 | 17 | q25.1 | 1.55E-04 | 2.96E-03 | 0.52 | 1.61 | 0.80 | 2.57 |
| SUMO2 | 17 | q25.1 | 1.56E-04 | 2.98E-03 | 0.52 | 1.54 | 0.80 | 5.03 |
| KCTD2 | 17 | q25.1 | 1.86E-04 | 3.43E-03 | 0.52 | 1.59 | 0.90 |  |
| FAM104A | 17 | q25.1 | 1.93E-04 | 3.52E-03 | 0.52 | 1.69 | 0.83 |  |
| LLGL2 | 17 | q25.1 | 1.93E-04 | 3.53E-03 | 0.52 | 1.23 | 0.88 | 2.03 |
| EXOC7 | 17 | q25.1 | 2.53E-04 | 4.32E-03 | 0.51 | 1.68 | 0.91 | 3.26 |
| SLC9A3R1 | 17 | q25.1 | 2.68E-04 | 4.51E-03 | 0.51 | 2.34 | 0.70 |  |
| H3F3B | 17 | q25.1 | 2.90E-04 | 4.80E-03 | 0.51 | 1.66 | 0.82 | 8.13 |
| PRPSAP1 | 17 | q25.1 | 7.96E-04 | 1.02E-02 | 0.47 | 1.19 | 0.78 |  |
| SLC25A19 | 17 | q25.1 | 8.80E-04 | 1.10E-02 | 0.47 | 1.49 | 0.93 | 2.50 |
| NAT9 | 17 | q25.1 | 9.91E-04 | 1.20E-02 | 0.47 | 1.56 | 0.89 |  |
| HN1 | 17 | q25.1 | 2.25E-03 | 2.20E-02 | 0.43 | 1.57 | 0.88 | 2.33 |
| ARMC7 | 17 | q25.1 | 3.18E-03 | 2.82E-02 | 0.42 | 1.45 | 0.94 | 1.72 |
| MIF4GD | 17 | q25.1 | 5.99E-03 | 4.44E-02 | 0.40 | 1.36 | 0.80 | 1.47 |
| GPS1 | 17 | q25.3 | 2.49E-10 | 9.09E-08 | 0.77 | 1.64 | 0.70 | 5.02 |
| DUS1L | 17 | q25.3 | 5.60E-10 | 1.73E-07 | 0.76 | 2.23 | 0.77 | 8.32 |
| NPB | 17 | q25.3 | 9.22E-10 | 2.48E-07 | 0.75 | 1.64 | 0.81 | 6.86 |
| C17orf101 | 17 | q25.3 | 4.43E-09 | 9.08E-07 | 0.73 | 1.35 | 0.79 | 3.24 |
| PCYT2 | 17 | q25.3 | 1.24E-08 | 2.10E-06 | 0.72 | 1.75 | 0.72 | 5.33 |
| SIRT7 | 17 | q25.3 | 3.21E-08 | 4.35E-06 | 0.70 | 1.57 | 0.76 | 5.12 |
| WDR45L | 17 | q25.3 | 3.91E-07 | 3.11E-05 | 0.66 | 1.94 | 0.74 | 3.03 |
| C17orf70 | 17 | q25.3 | 5.49E-07 | 4.05E-05 | 0.66 | 1.72 | 0.75 | 2.86 |
| MRPL12 | 17 | q25.3 | 7.16E-07 | 4.90E-05 | 0.65 | 2.19 | 0.67 | 4.27 |
| LRRC45 | 17 | q25.3 | 7.61E-07 | 5.13E-05 | 0.65 | 1.99 | 0.77 | 7.47 |
| NARF | 17 | q25.3 | 1.19E-06 | 7.20E-05 | 0.64 | 1.73 | 0.78 | 3.52 |
| ASPSCR1 | 17 | q25.3 | 1.20E-06 | 7.24E-05 | 0.64 | 1.55 | 0.80 | 6.31 |
| B3GNTL1 | 17 | q25.3 | 2.69E-06 | 1.31E-04 | 0.62 | 1.27 | 0.87 | 3.06 |
| DCXR | 17 | q25.3 | 3.04E-06 | 1.44E-04 | 0.62 | 1.99 | 0.60 | 6.10 |
| RFNG | 17 | q25.3 | 3.68E-06 | 1.70E-04 | 0.62 | 1.42 | 0.76 | 5.60 |
| AC127496.5-1 | 17 | q25.3 | 4.33E-06 | 1.92E-04 | 0.61 | 1.50 | 0.89 | 3.07 |
| STRA13 | 17 | q25.3 | 6.58E-06 | 2.65E-04 | 0.61 | 1.70 | 0.68 | 5.04 |
| RAB40B | 17 | q25.3 | 1.08E-05 | 3.92E-04 | 0.59 | 1.90 | 0.82 | 5.10 |
| PYCR1 | 17 | q25.3 | 1.35E-05 | 4.60E-04 | 0.59 | 1.31 | 0.89 | 2.73 |
| ANAPC11 | 17 | q25.3 | 1.44E-05 | 4.84E-04 | 0.59 | 1.45 | 0.71 | 2.94 |
| CSNK1D | 17 | q25.3 | 1.69E-05 | 5.44E-04 | 0.58 | 1.51 | 0.74 | 2.97 |
| HGS | 17 | q25.3 | 1.93E-05 | 6.00E-04 | 0.58 | 1.44 | 0.79 | 2.74 |
| RAC3 | 17 | q25.3 | 2.12E-05 | 6.43E-04 | 0.58 | 1.85 | 0.73 | 9.30 |
| ARHGDIA | 17 | q25.3 | 2.17E-05 | 6.55E-04 | 0.58 | 1.70 | 0.77 | 3.21 |
| SLC38A10 | 17 | q25.3 | 3.29E-05 | 9.16E-04 | 0.57 | 2.06 | 0.72 | 2.45 |
| THOC4 | 17 | q25.3 | 3.39E-05 | 9.37E-04 | 0.57 | 1.08 | 0.95 | 1.52 |
| AZI1 | 17 | q25.3 | 5.34E-05 | 1.33E-03 | 0.55 | 1.52 | 0.81 | 3.08 |
| EIF4A3 | 17 | q25.3 | 5.62E-05 | 1.37E-03 | 0.55 | 1.54 | 0.82 | 2.81 |
| FN3KRP | 17 | q25.3 | 7.42E-05 | 1.72E-03 | 0.55 | 1.46 | 0.78 | 2.76 |
| FN3K | 17 | q25.3 | 1.09E-04 | 2.28E-03 | 0.53 | 1.16 | 0.96 | 1.39 |
| ARL16 | 17 | q25.3 | 1.23E-04 | 2.49E-03 | 0.53 | 1.55 | 0.79 | 1.88 |
| TBC1D16 | 17 | q25.3 | 1.76E-04 | 3.26E-03 | 0.52 | 1.92 | 0.77 | 2.97 |
| SGSH | 17 | q25.3 | 1.90E-04 | 3.48E-03 | 0.52 | 1.36 | 0.86 | 3.63 |
| AC174470.1-1 | 17 | q25.3 | 2.19E-04 | 3.87E-03 | 0.51 | 1.75 | 0.70 | 4.34 |
| SLC26A11 | 17 | q25.3 | 3.46E-04 | 5.48E-03 | 0.50 | 1.21 | 0.87 | 1.87 |
| CHMP6 | 17 | q25.3 | 4.64E-04 | 6.88E-03 | 0.49 | 1.14 | 0.86 | 1.94 |
| FSCN2 | 17 | q25.3 | 5.84E-04 | 8.19E-03 | 0.48 | 1.06 | 0.97 | 1.52 |
| CCDC137 | 17 | q25.3 | 6.89E-04 | 9.17E-03 | 0.48 | 1.25 | 0.86 | 2.03 |
| C17orf90 | 17 | q25.3 | 8.04E-04 | 1.03E-02 | 0.47 | 1.19 | 0.75 | 3.33 |
| TBCD | 17 | q25.3 | 8.46E-04 | 1.06E-02 | 0.47 | 1.28 | 0.87 | 1.72 |
| FASN | 17 | q25.3 | 8.80E-04 | 1.10E-02 | 0.47 | 1.91 | 0.65 | 5.83 |
| ENGASE | 17 | q25.3 | 1.00E-03 | 1.21E-02 | 0.46 | 1.10 | 0.94 |  |
| TNRC6C | 17 | q25.3 | 1.11E-03 | 1.30E-02 | 0.46 | 1.17 | 0.99 | 2.46 |
| NPLOC4 | 17 | q25.3 | 1.91E-03 | 1.96E-02 | 0.44 | 1.49 | 0.75 | 2.29 |
| USP36 | 17 | q25.3 | 2.12E-03 | 2.12E-02 | 0.44 | 1.34 | 0.85 |  |
| GCGR | 17 | q25.3 | 2.14E-03 | 2.13E-02 | 0.44 | 1.68 | 0.98 | 3.41 |
| C17orf62 | 17 | q25.3 | 2.26E-03 | 2.21E-02 | 0.43 | 1.51 | 0.86 | 3.44 |
| P4HB | 17 | q25.3 | 2.27E-03 | 2.22E-02 | 0.43 | 1.71 | 0.78 | 2.09 |
| HEXDC | 17 | q25.3 | 3.14E-03 | 2.79E-02 | 0.42 | 1.22 | 0.87 | 2.06 |
| CCDC57 | 17 | q25.3 | 3.28E-03 | 2.88E-02 | 0.42 | 1.31 | 0.88 | 1.97 |
| TK1 | 17 | q25.3 | 4.50E-03 | 3.61E-02 | 0.41 | 1.79 | 0.75 |  |
| BAIAP2 | 17 | q25.3 | 6.05E-03 | 4.48E-02 | 0.39 | 1.36 | 0.70 | 2.40 |
| CARD14 | 17 | q25.3 | 6.91E-03 | 4.92E-02 | 0.39 | 1.12 | 0.93 | 1.61 |
| PTPN2 | 18 | p11.21 | 1.08E-04 | 2.27E-03 | 0.53 | 1.39 | 0.85 | 1.71 |
| RNMT | 18 | p11.21 | 5.88E-04 | 8.23E-03 | 0.48 | 1.20 | 0.82 | 1.37 |
| MPPE1 | 18 | p11.21 | 2.41E-03 | 2.32E-02 | 0.43 | 1.42 | 0.82 |  |
| NDUFV2 | 18 | p11.22 | 6.19E-05 | 1.48E-03 | 0.55 | 1.53 | 0.70 |  |
| ANKRD12 | 18 | p11.22 | 1.23E-03 | 1.40E-02 | 0.46 | 1.30 | 0.78 |  |
| RALBP1 | 18 | p11.22 | 1.41E-03 | 1.55E-02 | 0.45 | 1.48 | 0.79 |  |
| PPP4R1 | 18 | p11.22 | 3.66E-03 | 3.12E-02 | 0.42 | 1.42 | 0.77 |  |
| ENOSF1 | 18 | p11.32 | 1.59E-05 | 5.19E-04 | 0.58 | 2.09 | 0.63 |  |
| THOC1 | 18 | p11.32 | 2.80E-03 | 2.57E-02 | 0.43 | 1.19 | 0.73 |  |
| METTL4 | 18 | p11.32 | 4.05E-03 | 3.35E-02 | 0.41 | 1.25 | 0.89 |  |
| LAMA3 | 18 | q11.2 | 3.37E-03 | 2.93E-02 | 0.42 | 1.09 | 0.96 |  |
| C18orf21 | 18 | q12.2 | 1.13E-06 | 6.85E-05 | 0.64 | 1.51 | 0.80 |  |
| MOCOS | 18 | q12.2 | 2.28E-04 | 3.99E-03 | 0.51 | 1.23 | 0.69 |  |
| CXXC1 | 18 | q21.1 | 1.69E-05 | 5.44E-04 | 0.58 | 1.39 | 0.76 |  |
| DYM | 18 | q21.1 | 4.50E-05 | 1.16E-03 | 0.56 | 1.43 | 0.78 | 1.45 |
| ACAA2 | 18 | q21.1 | 4.22E-04 | 6.40E-03 | 0.49 | 1.82 | 0.75 | 3.07 |
| ATP5A1 | 18 | q21.1 | 6.77E-04 | 9.07E-03 | 0.48 | 1.34 | 0.81 |  |
| KATNAL2 | 18 | q21.1 | 8.42E-04 | 1.06E-02 | 0.47 | 1.43 | 0.83 |  |
| C18orf25 | 18 | q21.1 | 1.42E-03 | 1.56E-02 | 0.45 | 1.68 | 0.72 |  |
| SNORD58B | 18 | q21.1 | 2.51E-03 | 2.38E-02 | 0.43 | 1.64 | 0.67 | 1.50 |
| SMAD4 | 18 | q21.2 | 1.36E-03 | 1.51E-02 | 0.45 | 1.32 | 0.68 |  |
| FECH | 18 | q21.31 | 1.00E-03 | 1.21E-02 | 0.46 | 1.43 | 0.72 |  |
| WDR7 | 18 | q21.31 | 1.05E-03 | 1.26E-02 | 0.46 | 1.31 | 0.90 |  |
| NARS | 18 | q21.31 | 5.44E-03 | 4.14E-02 | 0.40 | 1.78 | 0.75 |  |
| SEC11L3 | 18 | q21.32 | 4.75E-05 | 1.21E-03 | 0.56 | 2.00 | 0.63 |  |
| PHLPP | 18 | q21.33 | 6.39E-07 | 4.47E-05 | 0.65 | 1.46 | 0.88 | 2.17 |
| KIAA1468 | 18 | q21.33 | 4.25E-06 | 1.90E-04 | 0.61 | 1.40 | 0.79 |  |
| BCL2 | 18 | q21.33 | 1.18E-05 | 4.17E-04 | 0.59 | 1.63 | 0.49 | 4.66 |
| VPS4B | 18 | q21.33 | 2.52E-04 | 4.31E-03 | 0.51 | 1.35 | 0.67 | 1.76 |
| FVT1 | 18 | q21.33 | 1.34E-03 | 1.50E-02 | 0.45 | 1.22 | 0.83 | 1.26 |
| PIGN | 18 | q21.33 | 5.98E-03 | 4.44E-02 | 0.40 | 1.14 | 0.79 |  |
| C18orf55 | 18 | q22.3 | 1.39E-04 | 2.73E-03 | 0.53 | 1.30 | 0.84 |  |
| AC116904.7 | 18 | q22.3 | 2.19E-03 | 2.16E-02 | 0.44 | 1.14 | 0.81 |  |
| CNDP2 | 18 | q22.3 | 6.48E-03 | 4.71E-02 | 0.39 | 1.48 | 0.88 |  |
| C18orf22 | 18 | q23 | 9.60E-04 | 1.16E-02 | 0.47 | 1.18 | 0.81 |  |
| AC090360.9 | 18 | q23 | 2.99E-03 | 2.70E-02 | 0.42 | 1.08 | 0.77 |  |
| TXNL4A | 18 | q23 | 7.06E-03 | 5.00E-02 | 0.39 | 1.33 | 0.92 |  |
| DDA1 | 19 | p13.11 | 2.29E-05 | 6.85E-04 | 0.58 | 1.97 | 0.83 |  |
| PGLS | 19 | p13.11 | 2.75E-05 | 7.94E-04 | 0.57 | 2.46 | 0.85 |  |
| C19orf62 | 19 | p13.11 | 4.59E-05 | 1.18E-03 | 0.56 | 1.99 | 0.91 |  |
| NDUFA13 | 19 | p13.11 | 6.53E-05 | 1.54E-03 | 0.55 | 1.88 | 0.80 |  |
| MAP1S | 19 | p13.11 | 1.71E-04 | 3.19E-03 | 0.52 | 1.89 | 0.86 |  |
| SIN3B | 19 | p13.11 | 1.96E-04 | 3.58E-03 | 0.52 | 2.32 | 0.97 |  |
| COPE | 19 | p13.11 | 1.99E-04 | 3.61E-03 | 0.52 | 1.66 | 0.75 |  |
| NR2F6 | 19 | p13.11 | 3.05E-04 | 5.00E-03 | 0.50 | 2.73 | 0.97 |  |
| USE1 | 19 | p13.11 | 3.22E-04 | 5.20E-03 | 0.50 | 1.62 | 0.90 |  |
| ABHD8 | 19 | p13.11 | 5.42E-04 | 7.74E-03 | 0.49 | 1.87 | 0.82 |  |
| MRPL34 | 19 | p13.11 | 6.79E-04 | 9.07E-03 | 0.48 | 2.08 | 0.89 |  |
| CALR3 | 19 | p13.11 | 7.17E-04 | 9.44E-03 | 0.48 | 1.59 | 0.89 |  |
| ARRDC2 | 19 | p13.11 | 1.24E-03 | 1.41E-02 | 0.46 | 2.22 | 0.89 |  |
| ARMC6 | 19 | p13.11 | 1.49E-03 | 1.62E-02 | 0.45 | 1.47 | 0.84 |  |
| LPAR2 | 19 | p13.11 | 1.54E-03 | 1.66E-02 | 0.45 | 1.84 | 0.95 |  |
| MYO9B | 19 | p13.11 | 2.06E-03 | 2.07E-02 | 0.44 | 1.53 | 0.95 |  |
| TM6SF2 | 19 | p13.11 | 2.80E-03 | 2.57E-02 | 0.43 | 1.12 | 0.95 |  |
| C19orf60 | 19 | p13.11 | 3.18E-03 | 2.82E-02 | 0.42 | 2.44 | 0.95 |  |
| HAUS8 | 19 | p13.11 | 3.43E-03 | 2.97E-02 | 0.42 | 1.21 | 0.92 |  |
| NR2C2AP | 19 | p13.11 | 3.92E-03 | 3.27E-02 | 0.41 | 1.74 | 0.87 |  |
| EPS15L1 | 19 | p13.11 | 4.07E-03 | 3.36E-02 | 0.41 | 1.67 | 0.98 |  |
| OCEL1 | 19 | p13.11 | 4.09E-03 | 3.37E-02 | 0.41 | 2.28 | 0.94 |  |
| GATAD2A | 19 | p13.11 | 4.33E-03 | 3.51E-02 | 0.41 | 1.50 | 0.86 |  |
| UPF1 | 19 | p13.11 | 5.29E-03 | 4.06E-02 | 0.40 | 1.83 | 0.82 |  |
| SF4 | 19 | p13.11 | 5.65E-03 | 4.26E-02 | 0.40 | 1.38 | 0.93 |  |
| CCDC124 | 19 | p13.11 | 6.16E-03 | 4.53E-02 | 0.39 | 1.84 | 0.84 |  |
| DDX39 | 19 | p13.12 | 2.09E-04 | 3.74E-03 | 0.52 | 1.76 | 0.91 |  |
| IL27RA | 19 | p13.12 | 4.01E-03 | 3.32E-02 | 0.41 | 4.55 | 0.68 |  |
| PKN1 | 19 | p13.12 | 4.33E-03 | 3.50E-02 | 0.41 | 2.13 | 0.78 |  |
| NDUFB7 | 19 | p13.12 | 5.49E-03 | 4.17E-02 | 0.40 |  | 0.89 |  |
| BRD4 | 19 | p13.12 | 6.67E-03 | 4.80E-02 | 0.39 | 1.57 | 0.77 |  |
| FAM32A | 19 | p13.12-p13.11 | 5.32E-03 | 4.08E-02 | 0.40 | 1.26 | 0.92 |  |
| TRMT1 | 19 | p13.2 | 5.73E-06 | 2.38E-04 | 0.61 | 2.31 | 0.91 |  |
| TMED1 | 19 | p13.2 | 7.17E-06 | 2.86E-04 | 0.60 | 1.75 | 0.80 |  |
| ELOF1 | 19 | p13.2 | 2.31E-05 | 6.91E-04 | 0.58 | 1.73 | 0.74 |  |
| C19orf52 | 19 | p13.2 | 4.73E-05 | 1.21E-03 | 0.56 | 1.65 | 0.82 |  |
| PIN1 | 19 | p13.2 | 9.66E-05 | 2.08E-03 | 0.54 | 1.68 | 0.83 |  |
| PPAN | 19 | p13.2 | 1.10E-04 | 2.29E-03 | 0.53 | 3.38 | 0.82 |  |
| C19orf43 | 19 | p13.2 | 3.69E-04 | 5.76E-03 | 0.50 | 1.35 | 0.85 |  |
| AC010422.7-1 | 19 | p13.2 | 4.21E-04 | 6.40E-03 | 0.49 | 1.76 | 0.87 |  |
| DNM2 | 19 | p13.2 | 6.45E-04 | 8.80E-03 | 0.48 | 1.67 | 0.76 |  |
| ECSIT | 19 | p13.2 | 6.55E-04 | 8.88E-03 | 0.48 | 1.70 | 0.87 |  |
| DHPS | 19 | p13.2 | 7.11E-04 | 9.38E-03 | 0.48 | 1.97 | 0.83 |  |
| RAD23A | 19 | p13.2 | 8.07E-04 | 1.03E-02 | 0.47 | 1.67 | 0.91 |  |
| CDKN2D | 19 | p13.2 | 8.39E-04 | 1.06E-02 | 0.47 | 2.33 | 0.84 |  |
| FARSA | 19 | p13.2 | 9.18E-04 | 1.13E-02 | 0.47 | 1.78 | 0.86 |  |
| EIF3G | 19 | p13.2 | 1.02E-03 | 1.23E-02 | 0.46 | 2.35 | 0.80 |  |
| MAN2B1 | 19 | p13.2 | 1.06E-03 | 1.26E-02 | 0.46 | 1.91 | 0.83 |  |
| TIMM44 | 19 | p13.2 | 1.17E-03 | 1.36E-02 | 0.46 |  | 0.78 |  |
| EVI5L | 19 | p13.2 | 1.27E-03 | 1.43E-02 | 0.46 |  | 0.75 |  |
| EMR4P | 19 | p13.2 | 1.39E-03 | 1.54E-02 | 0.45 | 1.07 | 0.95 |  |
| ZNF20 | 19 | p13.2 | 1.96E-03 | 2.00E-02 | 0.44 | 1.51 | 0.95 |  |
| C19orf39 | 19 | p13.2 | 2.21E-03 | 2.18E-02 | 0.44 | 1.87 | 0.92 |  |
| ZNF136 | 19 | p13.2 | 2.38E-03 | 2.30E-02 | 0.43 | 1.33 | 0.92 |  |
| PRKCSH | 19 | p13.2 | 3.36E-03 | 2.93E-02 | 0.42 | 2.01 | 0.77 |  |
| FBXW9 | 19 | p13.2 | 3.55E-03 | 3.04E-02 | 0.42 | 1.65 | 0.90 |  |
| MRI1 | 19 | p13.2 | 3.78E-03 | 3.19E-02 | 0.41 | 1.52 | 0.82 |  |
| NACC1 | 19 | p13.2 | 5.33E-03 | 4.09E-02 | 0.40 | 1.24 | 0.91 |  |
| DUS3L | 19 | p13.3 | 1.21E-06 | 7.33E-05 | 0.64 | 2.07 | 0.81 |  |
| RPL36P14 | 19 | p13.3 | 1.35E-06 | 7.95E-05 | 0.64 | 1.78 | 0.79 |  |
| LONP1 | 19 | p13.3 | 1.77E-06 | 9.88E-05 | 0.63 | 2.20 | 0.74 |  |
| UBXN6 | 19 | p13.3 | 1.18E-05 | 4.18E-04 | 0.59 | 3.11 | 0.78 |  |
| CLPP | 19 | p13.3 | 2.12E-05 | 6.44E-04 | 0.58 | 1.61 | 0.72 |  |
| AES | 19 | p13.3 | 3.04E-05 | 8.64E-04 | 0.57 | 2.77 | 0.70 |  |
| CCDC94 | 19 | p13.3 | 4.02E-05 | 1.07E-03 | 0.56 | 2.75 | 0.82 |  |
| SLC39A3 | 19 | p13.3 | 7.97E-05 | 1.81E-03 | 0.54 | 3.10 | 0.74 |  |
| C19orf70 | 19 | p13.3 | 9.67E-05 | 2.08E-03 | 0.54 | 2.12 | 0.84 |  |
| RAX2 | 19 | p13.3 | 1.36E-04 | 2.70E-03 | 0.53 |  | 0.80 |  |
| NDUFA11 | 19 | p13.3 | 1.64E-04 | 3.09E-03 | 0.52 | 1.78 | 0.73 |  |
| RANBP3 | 19 | p13.3 | 1.72E-04 | 3.21E-03 | 0.52 | 1.58 | 0.83 |  |
| SGTA | 19 | p13.3 | 2.94E-04 | 4.86E-03 | 0.51 | 2.14 | 0.73 |  |
| DOHH | 19 | p13.3 | 2.96E-04 | 4.87E-03 | 0.50 | 2.09 | 0.74 |  |
| LSM7 | 19 | p13.3 | 3.28E-04 | 5.28E-03 | 0.50 | 1.34 | 0.80 |  |
| MPND | 19 | p13.3 | 5.01E-04 | 7.30E-03 | 0.49 | 2.23 | 0.74 |  |
| NCLN | 19 | p13.3 | 6.37E-04 | 8.72E-03 | 0.48 | 3.21 | 0.83 |  |
| SF3A2 | 19 | p13.3 | 7.65E-04 | 9.94E-03 | 0.47 | 1.72 | 0.80 |  |
| GNA11 | 19 | p13.3 | 9.97E-04 | 1.20E-02 | 0.46 | 2.28 | 0.86 |  |
| SH2D3A | 19 | p13.3 | 1.15E-03 | 1.34E-02 | 0.46 | 1.37 | 0.84 |  |
| ALKBH7 | 19 | p13.3 | 1.34E-03 | 1.50E-02 | 0.45 | 1.67 | 0.79 |  |
| TICAM1 | 19 | p13.3 | 1.43E-03 | 1.57E-02 | 0.45 | 1.84 | 0.92 |  |
| AC004410.1 | 19 | p13.3 | 2.19E-03 | 2.16E-02 | 0.44 | 1.23 | 0.92 |  |
| NFIC | 19 | p13.3 | 2.46E-03 | 2.35E-02 | 0.43 | 2.41 | 0.84 |  |
| POLR2E | 19 | p13.3 | 2.84E-03 | 2.60E-02 | 0.43 | 1.48 | 0.75 |  |
| DAPK3 | 19 | p13.3 | 3.43E-03 | 2.97E-02 | 0.42 |  | 0.75 |  |
| LMNB2 | 19 | p13.3 | 3.44E-03 | 2.97E-02 | 0.42 | 1.20 | 0.81 |  |
| MOBKL2A | 19 | p13.3 | 3.72E-03 | 3.16E-02 | 0.42 | 1.35 | 0.85 |  |
| CRB3 | 19 | p13.3 | 3.93E-03 | 3.28E-02 | 0.41 | 1.54 | 0.77 |  |
| THOP1 | 19 | p13.3 | 4.68E-03 | 3.70E-02 | 0.41 | 1.91 | 0.79 |  |
| MRPL54 | 19 | p13.3 | 4.69E-03 | 3.71E-02 | 0.41 |  | 0.77 |  |
| GTF2F1 | 19 | p13.3 | 4.90E-03 | 3.83E-02 | 0.40 | 1.48 | 0.79 |  |
| C19orf21 | 19 | p13.3 | 5.32E-03 | 4.08E-02 | -0.40 | 0.81 | 1.20 |  |
| TMPRSS9 | 19 | p13.3 | 5.46E-03 | 4.15E-02 | 0.40 | 1.14 | 0.97 |  |
| CHAF1A | 19 | p13.3 | 6.20E-03 | 4.55E-02 | 0.39 | 1.44 | 0.90 |  |
| FAM108A4 | 19 | p13.3 | 6.62E-03 | 4.77E-02 | 0.39 | 1.18 | 0.85 |  |
| POP4 | 19 | q12 | 5.90E-04 | 8.23E-03 | 0.48 | 1.17 | 0.81 |  |
| C19orf12 | 19 | q12 | 3.78E-03 | 3.19E-02 | 0.41 | 1.28 | 0.81 |  |
| C19orf2 | 19 | q12 | 6.01E-03 | 4.45E-02 | 0.39 | 1.33 | 0.82 |  |
| PDCD5 | 19 | q13.11 | 1.20E-04 | 2.46E-03 | 0.53 | 1.38 | 0.69 |  |
| CEBPG | 19 | q13.11 | 3.10E-04 | 5.05E-03 | 0.50 | 1.69 | 0.85 |  |
| PDCD2L | 19 | q13.11 | 5.79E-04 | 8.15E-03 | 0.48 | 1.26 | 0.78 |  |
| GRAMD1A | 19 | q13.11-q13.12 | 1.47E-04 | 2.85E-03 | 0.53 | 1.82 | 0.79 |  |
| ZNF570 | 19 | q13.12 | 5.00E-12 | 3.96E-09 | 0.81 | 1.28 | 0.88 | 2.02 |
| ZNF585B | 19 | q13.12 | 6.25E-08 | 7.26E-06 | 0.69 | 1.61 | 0.87 | 3.23 |
| ZNF585A | 19 | q13.12 | 2.85E-07 | 2.49E-05 | 0.67 | 1.67 | 0.87 | 4.16 |
| ZNF569 | 19 | q13.12 | 3.35E-07 | 2.81E-05 | 0.67 | 1.59 | 0.81 | 3.07 |
| TMEM147 | 19 | q13.12 | 1.87E-05 | 5.83E-04 | 0.58 | 1.57 | 0.79 |  |
| AC008806.4 | 19 | q13.12 | 4.74E-05 | 1.21E-03 | 0.56 | 1.36 | 0.90 | 2.13 |
| ZNF540 | 19 | q13.12 | 6.58E-05 | 1.55E-03 | 0.55 | 1.20 | 0.78 | 3.11 |
| ZNF420 | 19 | q13.12 | 1.26E-04 | 2.55E-03 | 0.53 | 1.18 | 1.00 | 1.69 |
| LIN37 | 19 | q13.12 | 1.39E-04 | 2.73E-03 | 0.53 | 1.34 | 0.92 |  |
| TBCB | 19 | q13.12 | 1.63E-04 | 3.07E-03 | 0.52 | 1.56 | 0.77 |  |
| ZFP30 | 19 | q13.12 | 2.80E-04 | 4.67E-03 | 0.51 | 1.30 | 0.94 | 2.43 |
| ZNF383 | 19 | q13.12 | 6.30E-04 | 8.65E-03 | 0.48 | 1.15 | 0.92 | 1.49 |
| RBM42 | 19 | q13.12 | 6.76E-04 | 9.06E-03 | 0.48 | 1.29 | 0.82 |  |
| WDR62 | 19 | q13.12 | 1.29E-03 | 1.46E-02 | 0.46 | 1.27 | 0.87 |  |
| USF2 | 19 | q13.12 | 1.40E-03 | 1.54E-02 | 0.45 | 1.20 | 0.82 |  |
| ZNF529 | 19 | q13.12 | 1.49E-03 | 1.62E-02 | 0.45 | 1.13 | 0.73 |  |
| POLR2I | 19 | q13.12 | 1.53E-03 | 1.65E-02 | 0.45 | 1.35 | 0.76 |  |
| C19orf55 | 19 | q13.12 | 1.59E-03 | 1.70E-02 | 0.45 | 1.22 | 0.83 |  |
| PSENEN | 19 | q13.12 | 2.31E-03 | 2.24E-02 | 0.43 | 1.34 | 0.79 |  |
| CAPNS1 | 19 | q13.12 | 5.08E-03 | 3.94E-02 | 0.40 | 1.96 | 0.81 |  |
| ZFP82 | 19 | q13.12 | 6.87E-03 | 4.91E-02 | 0.39 | 1.45 | 0.88 |  |
| COX6B1 | 19 | q13.12 | 6.99E-03 | 4.96E-02 | 0.39 | 1.49 | 0.89 |  |
| ZNF573 | 19 | q13.12-q13.13 | 4.63E-03 | 3.68E-02 | 0.41 | 1.24 | 0.88 |  |
| EIF3K | 19 | q13.2 | 1.78E-10 | 6.77E-08 | 0.77 | 1.59 | 0.72 |  |
| PSMD8 | 19 | q13.2 | 1.45E-08 | 2.38E-06 | 0.72 | 1.83 | 0.78 |  |
| YIF1B | 19 | q13.2 | 3.94E-07 | 3.13E-05 | 0.66 | 1.70 | 0.76 |  |
| LGALS4 | 19 | q13.2 | 1.79E-06 | 9.94E-05 | 0.63 | 1.95 | 0.96 |  |
| ACTN4 | 19 | q13.2 | 1.34E-05 | 4.57E-04 | 0.59 | 2.26 | 0.77 |  |
| FAM98C | 19 | q13.2 | 1.61E-05 | 5.23E-04 | 0.58 | 1.60 | 0.87 |  |
| MRPS12 | 19 | q13.2 | 1.88E-05 | 5.86E-04 | 0.58 |  | 0.74 |  |
| DEDD2 | 19 | q13.2 | 2.43E-05 | 7.19E-04 | 0.57 |  | 0.77 |  |
| EXOSC5 | 19 | q13.2 | 8.08E-05 | 1.83E-03 | 0.54 |  | 0.72 |  |
| FBL | 19 | q13.2 | 1.43E-04 | 2.80E-03 | 0.53 | 1.26 | 0.82 |  |
| ATP5SL | 19 | q13.2 | 1.51E-04 | 2.91E-03 | 0.52 |  | 0.76 |  |
| SPINT2 | 19 | q13.2 | 2.50E-04 | 4.28E-03 | 0.51 | 1.90 | 0.85 |  |
| CCDC97 | 19 | q13.2 | 4.42E-04 | 6.64E-03 | 0.49 |  | 0.80 |  |
| TMEM91 | 19 | q13.2 | 4.88E-04 | 7.16E-03 | 0.49 |  | 0.69 |  |
| ZNF574 | 19 | q13.2 | 6.37E-04 | 8.72E-03 | 0.48 |  | 0.83 |  |
| PAF1 | 19 | q13.2 | 7.92E-04 | 1.02E-02 | 0.47 | 1.83 | 0.84 |  |
| DLL3 | 19 | q13.2 | 9.39E-04 | 1.14E-02 | 0.47 | 1.54 | 0.83 |  |
| ECH1 | 19 | q13.2 | 1.02E-03 | 1.22E-02 | 0.46 | 3.55 | 0.72 |  |
| EID2B | 19 | q13.2 | 1.19E-03 | 1.37E-02 | 0.46 | 1.97 | 0.93 |  |
| PAFAH1B3 | 19 | q13.2 | 1.30E-03 | 1.46E-02 | 0.46 |  | 0.61 |  |
| CEACAM1 | 19 | q13.2 | 1.56E-03 | 1.67E-02 | 0.45 |  | 0.88 |  |
| MAP3K10 | 19 | q13.2 | 1.99E-03 | 2.02E-02 | 0.44 | 1.47 | 0.91 |  |
| C19orf15 | 19 | q13.2 | 2.05E-03 | 2.07E-02 | 0.44 | 1.42 | 0.89 |  |
| SNRPA | 19 | q13.2 | 2.14E-03 | 2.13E-02 | 0.44 | 1.68 | 0.88 |  |
| ITPKC | 19 | q13.2 | 2.19E-03 | 2.16E-02 | 0.44 | 1.43 | 0.91 |  |
| PAK4 | 19 | q13.2 | 2.49E-03 | 2.37E-02 | 0.43 |  | 0.84 |  |
| FBXO17 | 19 | q13.2 | 3.63E-03 | 3.10E-02 | 0.42 |  | 0.77 |  |
| SUPT5H | 19 | q13.2 | 4.64E-03 | 3.68E-02 | 0.41 | 1.15 | 0.78 |  |
| C19orf47 | 19 | q13.2 | 6.16E-03 | 4.53E-02 | 0.39 | 1.03 | 0.88 |  |
| SAMD4B | 19 | q13.2 | 6.80E-03 | 4.87E-02 | 0.39 | 1.05 | 0.87 |  |
| ZNF225 | 19 | q13.31 | 7.44E-05 | 1.72E-03 | 0.55 | 1.15 | 0.92 |  |
| CADM4 | 19 | q13.31 | 1.08E-03 | 1.28E-02 | 0.46 |  | 0.66 |  |
| AC005392.2-1 | 19 | q13.31 | 1.11E-03 | 1.31E-02 | -0.46 |  | 1.05 |  |
| ZNF576 | 19 | q13.31 | 2.16E-03 | 2.14E-02 | 0.44 |  | 0.88 |  |
| ZNF227 | 19 | q13.31 | 2.21E-03 | 2.18E-02 | 0.44 | 1.16 | 0.91 |  |
| CLPTM1 | 19 | q13.32 | 3.62E-05 | 9.84E-04 | 0.56 |  | 0.70 |  |
| ERCC1 | 19 | q13.32 | 1.24E-03 | 1.41E-02 | 0.46 |  | 0.79 |  |
| KPTN | 19 | q13.32 | 1.53E-03 | 1.65E-02 | 0.45 |  | 0.84 |  |
| TOMM40 | 19 | q13.32 | 1.65E-03 | 1.75E-02 | 0.45 |  | 0.77 |  |
| MYPOP | 19 | q13.32 | 2.07E-03 | 2.08E-02 | 0.44 |  | 0.86 |  |
| STRN4 | 19 | q13.32 | 2.43E-03 | 2.33E-02 | 0.43 |  | 0.79 |  |
| SAE1 | 19 | q13.32 | 2.88E-03 | 2.63E-02 | 0.43 |  | 0.85 |  |
| PPP1R13L | 19 | q13.32 | 3.03E-03 | 2.73E-02 | 0.42 |  | 0.73 |  |
| SFRS16 | 19 | q13.32 | 4.52E-03 | 3.62E-02 | 0.41 |  | 0.88 |  |
| TRAPPC6A | 19 | q13.32 | 4.65E-03 | 3.69E-02 | 0.41 |  | 0.80 |  |
| PRMT1 | 19 | q13.33 | 4.42E-08 | 5.57E-06 | 0.70 | 2.28 | 0.70 |  |
| RUVBL2 | 19 | q13.33 | 6.02E-07 | 4.28E-05 | 0.65 | 2.02 | 0.70 |  |
| LIN7B | 19 | q13.33 | 5.79E-06 | 2.39E-04 | 0.61 | 1.40 | 0.72 |  |
| BCL2L12 | 19 | q13.33 | 3.61E-04 | 5.66E-03 | 0.50 | 1.62 | 0.83 |  |
| C19orf48 | 19 | q13.33 | 6.48E-04 | 8.83E-03 | 0.48 | 1.73 | 0.82 |  |
| PTOV1 | 19 | q13.33 | 1.32E-03 | 1.48E-02 | 0.45 | 1.44 | 0.79 |  |
| RPL13A | 19 | q13.33 | 1.38E-03 | 1.53E-02 | 0.45 | 2.59 | 0.77 |  |
| NOSIP | 19 | q13.33 | 1.67E-03 | 1.77E-02 | 0.45 | 2.15 | 0.82 |  |
| CYTH2 | 19 | q13.33 | 1.89E-03 | 1.95E-02 | 0.44 |  | 0.81 |  |
| GLTSCR1 | 19 | q13.33 | 1.95E-03 | 1.99E-02 | 0.44 |  | 0.83 |  |
| AKT1S1 | 19 | q13.33 | 1.98E-03 | 2.02E-02 | 0.44 | 1.63 | 0.92 |  |
| VRK3 | 19 | q13.33 | 3.31E-03 | 2.90E-02 | 0.42 | 1.16 | 0.78 |  |
| GRWD1 | 19 | q13.33 | 4.13E-03 | 3.39E-02 | 0.41 |  | 0.85 |  |
| NR1H2 | 19 | q13.33 | 5.95E-03 | 4.42E-02 | 0.40 | 1.36 | 0.80 |  |
| ZNF615 | 19 | q13.41 | 3.92E-06 | 1.79E-04 | 0.62 | 1.36 | 0.82 |  |
| ATPBD3 | 19 | q13.41 | 1.31E-05 | 4.51E-04 | 0.59 | 1.30 | 0.93 | 1.39 |
| ZNF480 | 19 | q13.41 | 1.58E-05 | 5.17E-04 | 0.58 | 1.41 | 0.76 |  |
| PPP2R1A | 19 | q13.41 | 4.76E-05 | 1.21E-03 | 0.56 | 1.37 | 0.72 |  |
| ZNF766 | 19 | q13.41 | 4.79E-03 | 3.77E-02 | 0.40 | 1.10 | 0.83 |  |
| ZNF28 | 19 | q13.41-q13.42 | 1.05E-03 | 1.25E-02 | 0.46 | 1.11 | 0.80 |  |
| TSEN34 | 19 | q13.42 | 2.07E-05 | 6.32E-04 | 0.58 | 1.38 | 0.79 |  |
| ISOC2 | 19 | q13.42 | 2.25E-05 | 6.76E-04 | 0.58 | 1.30 | 0.73 |  |
| NDUFA3 | 19 | q13.42 | 2.39E-04 | 4.14E-03 | 0.51 | 1.03 | 0.77 |  |
| EPN1 | 19 | q13.42 | 2.41E-04 | 4.17E-03 | 0.51 | 1.27 | 0.80 |  |
| ZNF581 | 19 | q13.42 | 6.86E-04 | 9.15E-03 | 0.48 | 1.22 | 0.73 |  |
| U2AF2 | 19 | q13.42 | 6.98E-04 | 9.24E-03 | 0.48 | 1.28 | 0.92 |  |
| ZNF524 | 19 | q13.42 | 9.07E-04 | 1.12E-02 | 0.47 | 1.24 | 0.73 |  |
| NAT14 | 19 | q13.42 | 1.22E-03 | 1.40E-02 | 0.46 | 1.10 | 0.70 |  |
| PRPF31 | 19 | q13.42 | 2.26E-03 | 2.21E-02 | 0.43 | 1.06 | 0.76 |  |
| ZNF579 | 19 | q13.42 | 2.66E-03 | 2.48E-02 | 0.43 | 1.17 | 0.77 |  |
| CNOT3 | 19 | q13.42 | 3.42E-03 | 2.96E-02 | 0.42 | 1.06 | 0.82 |  |
| SAPS1 | 19 | q13.42 | 3.45E-03 | 2.98E-02 | 0.42 | 1.19 | 0.80 |  |
| FIZ1 | 19 | q13.42 | 4.18E-03 | 3.42E-02 | 0.41 | 1.16 | 0.73 |  |
| TMEM86B | 19 | q13.42 | 4.28E-03 | 3.48E-02 | 0.41 | 1.21 | 0.88 |  |
| AC020922.9 | 19 | q13.42 | 6.26E-03 | 4.59E-02 | 0.39 | 1.24 | 0.68 |  |
| RPL28 | 19 | q13.42 | 6.93E-03 | 4.93E-02 | 0.39 | 1.70 | 0.87 |  |
| ZNF17 | 19 | q13.43 | 1.23E-07 | 1.26E-05 | 0.68 | 1.59 | 0.79 |  |
| ZNF787 | 19 | q13.43 | 4.18E-05 | 1.10E-03 | 0.56 | 1.27 | 0.76 |  |
| ZNF416 | 19 | q13.43 | 6.14E-04 | 8.48E-03 | 0.48 | 1.23 | 0.87 |  |
| UBE2MP1 | 19 | q13.43 | 1.64E-03 | 1.74E-02 | 0.45 | 1.34 | 0.84 |  |
| ZNF419 | 19 | q13.43 | 2.00E-03 | 2.03E-02 | 0.44 | 1.64 | 0.81 |  |
| AC020915.4 | 19 | q13.43 | 2.79E-03 | 2.57E-02 | 0.43 | 1.89 | 0.73 |  |
| ZNF550 | 19 | q13.43 | 2.98E-03 | 2.69E-02 | 0.42 | 1.39 | 0.91 |  |
| ZNF606 | 19 | q13.43 | 4.15E-03 | 3.40E-02 | 0.41 | 1.54 | 0.81 |  |
| C20orf3 | 20 | p11.21 | 1.35E-03 | 1.50E-02 | 0.45 | 1.50 | 0.82 |  |
| NXT1 | 20 | p11.21 | 5.62E-03 | 4.24E-02 | 0.40 | 1.29 | 0.84 |  |
| ACSS1 | 20 | p11.21 | 5.83E-03 | 4.36E-02 | 0.40 | 1.58 | 0.64 |  |
| RBBP9 | 20 | p11.23 | 1.58E-03 | 1.69E-02 | 0.45 | 1.23 | 0.68 |  |
| ZNF133 | 20 | p11.23 | 2.20E-03 | 2.16E-02 | 0.44 | 1.17 | 0.76 |  |
| SLC24A3 | 20 | p11.23 | 2.47E-03 | 2.36E-02 | 0.43 | 1.71 | 0.62 |  |
| CSRP2BP | 20 | p11.23 | 7.01E-03 | 4.97E-02 | 0.39 | 1.16 | 0.99 |  |
| C20orf7 | 20 | p12.1 | 2.16E-03 | 2.14E-02 | 0.44 | 1.37 | 0.83 |  |
| RRBP1 | 20 | p12.1 | 2.43E-03 | 2.33E-02 | 0.43 | 1.30 | 0.48 |  |
| IDH3B | 20 | p13 | 1.09E-06 | 6.72E-05 | 0.64 | 1.58 | 0.81 |  |
| RNF24 | 20 | p13 | 5.67E-06 | 2.37E-04 | 0.61 | 2.26 | 0.52 |  |
| ZNF343 | 20 | p13 | 7.69E-06 | 3.03E-04 | 0.60 | 1.21 | 0.75 |  |
| ITPA | 20 | p13 | 4.11E-05 | 1.08E-03 | 0.56 | 1.26 | 0.69 |  |
| NSFL1C | 20 | p13 | 1.09E-04 | 2.28E-03 | 0.53 | 1.25 | 0.73 |  |
| TBC1D20 | 20 | p13 | 1.22E-04 | 2.49E-03 | 0.53 | 1.11 | 0.77 |  |
| RBCK1 | 20 | p13 | 2.38E-04 | 4.13E-03 | 0.51 | 1.35 | 0.75 |  |
| PSMF1 | 20 | p13 | 3.34E-04 | 5.36E-03 | 0.50 | 1.33 | 0.77 |  |
| NOP56 | 20 | p13 | 3.88E-04 | 5.98E-03 | 0.50 | 1.41 | 0.65 |  |
| DDRGK1 | 20 | p13 | 4.09E-04 | 6.26E-03 | 0.49 | 1.42 | 0.54 |  |
| STK35 | 20 | p13 | 5.80E-04 | 8.16E-03 | 0.48 | 1.28 | 0.65 |  |
| PANK2 | 20 | p13 | 1.22E-03 | 1.40E-02 | 0.46 | 1.42 | 0.75 |  |
| CSNK2A1 | 20 | p13 | 3.19E-03 | 2.83E-02 | 0.42 | 1.19 | 0.69 |  |
| C20orf27 | 20 | p13 | 3.42E-03 | 2.96E-02 | 0.42 | 1.19 | 0.82 |  |
| MAVS | 20 | p13 | 4.03E-03 | 3.33E-02 | 0.41 | 1.20 | 0.81 |  |
| FKBP1A | 20 | p13 | 4.36E-03 | 3.52E-02 | 0.41 | 1.40 | 0.85 |  |
| MRPS26 | 20 | p13 | 4.97E-03 | 3.87E-02 | 0.40 | 1.18 | 0.74 |  |
| VPS16 | 20 | p13 | 5.28E-03 | 4.06E-02 | 0.40 | 1.17 | 0.76 |  |
| C20orf96 | 20 | p13 | 5.78E-03 | 4.33E-02 | 0.40 | 1.27 | 0.77 |  |
| UBOX5 | 20 | p13 | 6.28E-03 | 4.60E-02 | 0.39 | 1.28 | 0.77 |  |
| EFCAB8 | 20 | q11.21 | 4.39E-08 | 5.56E-06 | 0.70 | 1.16 | 0.94 |  |
| CDK5RAP1 | 20 | q11.21 | 3.62E-04 | 5.67E-03 | 0.50 | 1.37 | 0.92 |  |
| COMMD7 | 20 | q11.21 | 3.56E-03 | 3.05E-02 | 0.42 | 1.29 | 1.00 |  |
| RBM39 | 20 | q11.22 | 3.84E-10 | 1.29E-07 | 0.76 | 1.22 | 0.83 | 2.16 |
| ROMO1 | 20 | q11.22 | 7.31E-10 | 2.10E-07 | 0.76 | 1.48 | 0.70 | 4.17 |
| ERGIC3 | 20 | q11.22 | 3.78E-07 | 3.07E-05 | 0.66 | 1.35 | 0.83 | 3.20 |
| CEP250 | 20 | q11.22 | 5.39E-06 | 2.28E-04 | 0.61 | 1.21 | 0.92 | 2.12 |
| EIF6 | 20 | q11.22 | 4.85E-05 | 1.23E-03 | 0.56 | 1.40 | 0.74 | 1.86 |
| UQCC | 20 | q11.22 | 5.57E-05 | 1.36E-03 | 0.55 | 1.59 | 0.95 | 2.91 |
| CDC91L1 | 20 | q11.22 | 6.49E-05 | 1.54E-03 | 0.55 | 1.56 | 0.97 |  |
| GSS | 20 | q11.22 | 1.35E-04 | 2.68E-03 | 0.53 | 1.30 | 0.85 | 2.74 |
| RALY | 20 | q11.22 | 1.57E-04 | 2.99E-03 | 0.52 | 1.44 | 0.77 |  |
| TRPC4AP | 20 | q11.22 | 3.50E-04 | 5.53E-03 | 0.50 | 1.15 | 0.77 | 2.09 |
| CPNE1 | 20 | q11.22 | 3.93E-04 | 6.04E-03 | 0.50 | 1.39 | 0.69 | 1.74 |
| EDEM2 | 20 | q11.22 | 8.36E-04 | 1.06E-02 | 0.47 | 1.23 | 0.70 | 2.74 |
| NCOA6 | 20 | q11.22 | 2.97E-03 | 2.68E-02 | 0.42 | 1.38 | 0.89 |  |
| C20orf4 | 20 | q11.23 | 2.75E-09 | 5.99E-07 | 0.74 | 1.43 | 0.81 | 2.68 |
| C20orf152 | 20 | q11.23 | 1.74E-07 | 1.67E-05 | 0.68 | 1.12 | 0.93 | 2.20 |
| CTNNBL1 | 20 | q11.23 | 1.42E-06 | 8.27E-05 | 0.64 | 1.44 | 0.69 |  |
| KIAA0406 | 20 | q11.23 | 1.45E-05 | 4.86E-04 | 0.59 | 1.34 | 0.90 |  |
| C20orf24 | 20 | q11.23 | 2.35E-05 | 7.01E-04 | 0.58 | 1.47 | 0.72 | 2.75 |
| SCAND1 | 20 | q11.23 | 1.57E-04 | 2.99E-03 | 0.52 | 1.40 | 0.75 | 3.24 |
| SNHG11 | 20 | q11.23 | 8.49E-04 | 1.07E-02 | 0.47 | 1.30 | 0.87 |  |
| DLGAP4 | 20 | q11.23 | 9.87E-04 | 1.19E-02 | 0.47 | 1.29 | 0.66 | 1.90 |
| C20orf117 | 20 | q11.23 | 1.09E-03 | 1.29E-02 | 0.46 | 1.16 | 0.91 |  |
| KIAA1219 | 20 | q11.23 | 2.51E-03 | 2.38E-02 | 0.43 | 1.20 | 0.92 |  |
| EPB41L1 | 20 | q11.23 | 3.12E-03 | 2.78E-02 | 0.42 | 1.56 | 1.00 | 2.33 |
| NDRG3 | 20 | q11.23 | 5.92E-03 | 4.40E-02 | 0.40 | 1.15 | 0.80 |  |
| MANBAL | 20 | q11.23 | 6.52E-03 | 4.72E-02 | 0.39 | 1.25 | 0.86 |  |
| C20orf102 | 20 | q11.23 | 6.61E-03 | 4.77E-02 | 0.39 | 1.25 | 0.84 |  |
| ZHX3 | 20 | q12 | 1.27E-05 | 4.41E-04 | 0.59 | 1.15 | 1.05 | 3.11 |
| ZSWIM1 | 20 | q13.12 | 4.51E-06 | 1.98E-04 | 0.61 | 1.36 | 0.91 |  |
| DNTTIP1 | 20 | q13.12 | 3.22E-05 | 9.00E-04 | 0.57 | 1.22 | 0.88 |  |
| UBE2C | 20 | q13.12 | 7.64E-05 | 1.76E-03 | 0.54 | 1.91 | 0.80 |  |
| SLC13A3 | 20 | q13.12 | 1.78E-04 | 3.29E-03 | 0.52 | 1.10 | 0.95 |  |
| ACOT8 | 20 | q13.12 | 1.97E-04 | 3.58E-03 | 0.52 | 1.22 | 0.84 |  |
| SLC35C2 | 20 | q13.12 | 2.54E-04 | 4.33E-03 | 0.51 | 1.30 | 0.85 |  |
| SERINC3 | 20 | q13.12 | 4.18E-04 | 6.37E-03 | 0.49 | 1.26 | 1.03 | 2.02 |
| PCIF1 | 20 | q13.12 | 6.67E-04 | 8.97E-03 | 0.48 | 1.16 | 0.91 |  |
| STK4 | 20 | q13.12 | 7.50E-04 | 9.77E-03 | 0.47 | 1.32 | 0.82 |  |
| NCOA3 | 20 | q13.12 | 1.09E-03 | 1.29E-02 | 0.46 | 1.31 | 0.88 | 2.61 |
| TTPAL | 20 | q13.12 | 5.48E-03 | 4.16E-02 | 0.40 | 1.17 | 1.07 | 1.74 |
| CSE1L | 20 | q13.13 | 6.01E-07 | 4.28E-05 | 0.65 | 1.29 | 0.89 |  |
| TMEM189-UBE2V1 | 20 | q13.13 | 2.04E-06 | 1.08E-04 | 0.63 | 1.13 | 0.98 | 1.93 |
| SPATA2 | 20 | q13.13 | 2.76E-05 | 7.94E-04 | 0.57 | 1.22 | 0.81 | 2.15 |
| STAU1 | 20 | q13.13 | 5.10E-04 | 7.40E-03 | 0.49 | 1.31 | 0.84 |  |
| B4GALT5 | 20 | q13.13 | 5.35E-04 | 7.65E-03 | 0.49 | 1.27 | 0.92 | 2.75 |
| ARFGEF2 | 20 | q13.13 | 5.86E-04 | 8.21E-03 | 0.48 | 1.26 | 0.91 |  |
| PARD6B | 20 | q13.13 | 8.82E-04 | 1.10E-02 | 0.47 | 1.19 | 0.86 | 3.93 |
| DDX27 | 20 | q13.13 | 9.24E-04 | 1.13E-02 | 0.47 | 1.18 | 0.85 |  |
| CEBPB | 20 | q13.13 | 1.60E-03 | 1.71E-02 | 0.45 | 1.29 | 0.94 | 2.92 |
| RNF114 | 20 | q13.13 | 2.40E-03 | 2.31E-02 | 0.43 | 1.21 | 0.85 | 1.63 |
| DPM1 | 20 | q13.13 | 2.43E-03 | 2.33E-02 | 0.43 | 1.48 | 0.77 | 2.29 |
| MOCS3 | 20 | q13.13 | 3.58E-03 | 3.06E-02 | 0.42 | 1.12 | 0.91 | 1.06 |
| AURKA | 20 | q13.2 | 2.59E-04 | 4.39E-03 | 0.51 | 1.82 | 0.83 |  |
| ZNF217 | 20 | q13.2 | 9.26E-04 | 1.14E-02 | 0.47 | 1.43 | 1.28 | 2.66 |
| RAE1 | 20 | q13.31 | 1.72E-05 | 5.47E-04 | 0.58 | 1.47 | 0.90 |  |
| C20orf43 | 20 | q13.31 | 5.23E-04 | 7.52E-03 | 0.49 | 1.27 | 0.87 |  |
| CTCFL | 20 | q13.31 | 7.34E-04 | 9.62E-03 | 0.48 | 1.04 | 0.97 | 1.19 |
| TH1L | 20 | q13.32 | 2.06E-05 | 6.30E-04 | 0.58 | 1.32 | 0.91 | 1.75 |
| RAB22A | 20 | q13.32 | 4.31E-05 | 1.13E-03 | 0.56 | 1.41 | 0.74 | 2.06 |
| NPEPL1 | 20 | q13.32 | 2.26E-04 | 3.97E-03 | 0.51 | 1.44 | 0.80 | 1.95 |
| STX16 | 20 | q13.32 | 6.59E-04 | 8.90E-03 | 0.48 | 1.43 | 1.12 | 3.18 |
| KCNQ2 | 20 | q13.33 | 4.25E-10 | 1.40E-07 | 0.76 | 1.13 | 0.96 | 6.75 |
| YTHDF1 | 20 | q13.33 | 4.24E-07 | 3.31E-05 | 0.66 | 1.40 | 0.94 | 3.60 |
| ZGPAT | 20 | q13.33 | 2.95E-06 | 1.41E-04 | 0.62 | 1.23 | 0.74 | 3.15 |
| TPD52L2 | 20 | q13.33 | 3.76E-06 | 1.73E-04 | 0.62 | 1.36 | 0.74 | 1.68 |
| SS18L1 | 20 | q13.33 | 5.42E-06 | 2.29E-04 | 0.61 | 1.50 | 0.69 | 1.85 |
| C20orf20 | 20 | q13.33 | 1.95E-05 | 6.02E-04 | 0.58 | 1.34 | 0.92 | 2.51 |
| PSMA7 | 20 | q13.33 | 1.37E-04 | 2.72E-03 | 0.53 | 1.53 | 0.90 | 2.31 |
| DNAJC5 | 20 | q13.33 | 4.85E-04 | 7.12E-03 | 0.49 | 1.18 | 0.86 | 1.74 |
| ADRM1 | 20 | q13.33 | 6.44E-04 | 8.79E-03 | 0.48 | 1.26 | 0.88 | 2.54 |
| C20orf149 | 20 | q13.33 | 9.37E-04 | 1.14E-02 | 0.47 | 1.29 | 1.01 | 3.78 |
| TAF4 | 20 | q13.33 | 1.18E-03 | 1.37E-02 | 0.46 | 1.38 | 0.72 | 1.36 |
| RTEL1 | 20 | q13.33 | 1.35E-03 | 1.50E-02 | 0.45 | 1.11 | 0.94 | 1.73 |
| NCRNA00176 | 20 | q13.33 | 2.61E-03 | 2.45E-02 | 0.43 | 1.11 | 1.04 | 1.84 |
| C20orf11 | 20 | q13.33 | 3.24E-03 | 2.85E-02 | 0.42 | 1.39 | 0.86 | 2.13 |
| OSBPL2 | 20 | q13.33 | 3.30E-03 | 2.90E-02 | 0.42 | 1.29 | 0.79 | 1.34 |
| PPP1R3D | 20 | q13.33 | 3.34E-03 | 2.92E-02 | 0.42 | 1.20 | 0.79 |  |
| GTPBP5 | 20 | q13.33 | 4.09E-03 | 3.37E-02 | 0.41 | 1.07 | 0.92 | 1.27 |
| ARF1GAP | 20 | q13.33 | 5.28E-03 | 4.06E-02 | 0.40 | 1.30 | 0.85 | 2.38 |
| UCKL1 | 20 | q13.33 | 5.65E-03 | 4.26E-02 | 0.40 | 1.22 | 1.04 | 1.50 |
| SLC2A4RG | 20 | q13.33 | 6.89E-03 | 4.91E-02 | 0.39 | 1.27 | 0.81 | 1.49 |
| USP16 | 21 | q21.3 | 3.42E-04 | 5.45E-03 | 0.50 | 1.08 | 0.75 |  |
| HEMK2 | 21 | q21.3 | 1.44E-03 | 1.58E-02 | 0.45 | 1.24 | 0.87 |  |
| ATP5J | 21 | q21.3 | 2.99E-03 | 2.70E-02 | 0.42 | 1.36 | 0.77 |  |
| RNF160 | 21 | q21.3 | 3.52E-03 | 3.03E-02 | 0.42 | 1.07 | 0.85 |  |
| C21orf119 | 21 | q22.11 | 5.06E-04 | 7.35E-03 | 0.49 | 1.35 | 0.87 |  |
| SOD1 | 21 | q22.11 | 1.41E-03 | 1.55E-02 | 0.45 | 1.77 | 0.80 |  |
| SFRS15 | 21 | q22.11 | 3.17E-03 | 2.81E-02 | 0.42 | 1.02 | 0.84 |  |
| TCP10L | 21 | q22.11 | 6.02E-03 | 4.46E-02 | 0.39 | 1.30 | 0.94 |  |
| PIGP | 21 | q22.13 | 7.03E-03 | 4.98E-02 | 0.39 | 1.57 | 0.95 |  |
| HLCS | 21 | q22.13 | 7.04E-03 | 4.99E-02 | 0.39 | 1.30 | 0.86 |  |
| SH3BGR | 21 | q22.2 | 4.07E-03 | 3.36E-02 | 0.41 | 1.14 | 0.97 |  |
| C21orf57 | 21 | q22.3 | 1.93E-03 | 1.98E-02 | 0.44 | 1.27 | 0.74 |  |
| C21orf56 | 21 | q22.3 | 2.63E-03 | 2.46E-02 | 0.43 | 1.53 | 0.84 |  |
| PKNOX1 | 21 | q22.3 | 2.99E-03 | 2.70E-02 | 0.42 | 1.27 | 0.84 |  |
| PWP2H | 21 | q22.3 | 3.23E-03 | 2.85E-02 | 0.42 | 1.81 | 0.86 |  |
| LSS | 21 | q22.3 | 3.30E-03 | 2.90E-02 | 0.42 | 1.91 | 0.88 |  |
| C21orf90 | 21 | q22.3 | 5.12E-03 | 3.96E-02 | -0.40 | 0.94 | 1.03 |  |
| LA16c-23H5.6 | 22 | q11.1 | 5.18E-03 | 4.00E-02 | 0.40 | 1.05 | 0.99 | 1.22 |
| UBE2L3 | 22 | q11.21 | 1.50E-06 | 8.72E-05 | 0.64 |  | 0.74 |  |
| COMT | 22 | q11.21 | 9.98E-06 | 3.67E-04 | 0.60 |  | 0.72 |  |
| CRKL | 22 | q11.21 | 1.72E-05 | 5.47E-04 | 0.58 |  | 0.82 |  |
| BCL2L13 | 22 | q11.21 | 3.07E-05 | 8.70E-04 | 0.57 |  | 0.83 |  |
| TXNRD2 | 22 | q11.21 | 1.05E-04 | 2.22E-03 | 0.54 |  | 0.75 |  |
| HIC2 | 22 | q11.21 | 6.84E-04 | 9.13E-03 | 0.48 |  | 0.88 |  |
| CDC45L | 22 | q11.21 | 9.13E-04 | 1.12E-02 | 0.47 |  | 0.89 |  |
| DGCR14 | 22 | q11.21 | 1.85E-03 | 1.91E-02 | 0.44 | 0.96 | 0.88 |  |
| RPL7AP70 | 22 | q11.21 | 1.94E-03 | 1.98E-02 | 0.44 |  | 0.90 |  |
| DGCR8 | 22 | q11.21 | 4.19E-03 | 3.42E-02 | 0.41 |  | 0.78 |  |
| CLTCL1 | 22 | q11.21 | 6.57E-03 | 4.75E-02 | 0.39 |  | 0.92 |  |
| PI4KA | 22 | q11.21-q11.22 | 5.46E-04 | 7.78E-03 | 0.49 |  | 0.86 |  |
| ZNF280B | 22 | q11.22 | 1.27E-03 | 1.43E-02 | 0.46 | 2.70 | 0.95 |  |
| SLC2A11 | 22 | q11.23 | 2.11E-05 | 6.43E-04 | 0.58 | 0.89 | 0.69 |  |
| LRP5L | 22 | q11.23 | 3.41E-04 | 5.45E-03 | 0.50 | 0.91 | 0.79 |  |
| SMARCB1 | 22 | q11.23 | 3.59E-04 | 5.63E-03 | 0.50 | 1.58 | 0.83 |  |
| CHCHD10 | 22 | q11.23 | 5.74E-04 | 8.09E-03 | 0.48 | 3.34 | 0.84 |  |
| DDT | 22 | q11.23 | 1.31E-03 | 1.47E-02 | 0.46 | 2.17 | 0.87 |  |
| SNRPD3 | 22 | q11.23 | 2.03E-03 | 2.05E-02 | 0.44 | 1.63 | 0.82 |  |
| CYTSA | 22 | q11.23 | 3.95E-03 | 3.28E-02 | 0.41 | 0.91 | 0.79 |  |
| CTA-221G9.5 | 22 | q11.23 | 3.96E-03 | 3.29E-02 | 0.41 | 3.21 | 0.79 |  |
| CRYBB2 | 22 | q11.23-q12.1 | 4.89E-03 | 3.82E-02 | 0.40 | 1.12 | 0.84 |  |
| TFIP11 | 22 | q12.1 | 7.58E-05 | 1.75E-03 | 0.54 | 1.01 | 0.88 |  |
| PITPNB | 22 | q12.1 | 9.43E-05 | 2.04E-03 | 0.54 | 1.49 | 0.79 |  |
| HSCB | 22 | q12.1 | 9.32E-04 | 1.14E-02 | 0.47 | 1.56 | 0.90 |  |
| SRRD | 22 | q12.1 | 3.26E-03 | 2.87E-02 | 0.42 | 1.62 | 0.94 |  |
| HPS4 | 22 | q12.1 | 4.14E-03 | 3.40E-02 | 0.41 | 1.13 | 0.95 |  |
| DRG1 | 22 | q12.2 | 3.97E-06 | 1.80E-04 | 0.62 |  | 0.85 |  |
| PES1 | 22 | q12.2 | 1.09E-04 | 2.27E-03 | 0.53 |  | 0.90 |  |
| ASCC2 | 22 | q12.2 | 2.95E-04 | 4.87E-03 | 0.50 | 0.87 | 0.79 |  |
| UCRC | 22 | q12.2 | 1.03E-03 | 1.23E-02 | 0.46 | 1.34 | 0.92 |  |
| DUSP18 | 22 | q12.2 | 1.06E-03 | 1.26E-02 | 0.46 |  | 0.86 |  |
| THOC5 | 22 | q12.2 | 3.38E-03 | 2.94E-02 | 0.42 | 1.08 | 0.94 |  |
| SLC35E4 | 22 | q12.2 | 6.14E-03 | 4.52E-02 | 0.39 |  | 0.96 |  |
| MORC2 | 22 | q12.2 | 6.42E-03 | 4.67E-02 | 0.39 |  | 0.80 |  |
| EIF3D | 22 | q12.3 | 2.64E-05 | 7.69E-04 | 0.57 |  | 0.82 |  |
| RBM9 | 22 | q12.3 | 6.77E-04 | 9.07E-03 | 0.48 |  | 0.69 |  |
| FBXO7 | 22 | q12.3 | 7.92E-04 | 1.02E-02 | 0.47 |  | 0.92 |  |
| MCM5 | 22 | q12.3 | 7.97E-04 | 1.02E-02 | 0.47 |  | 0.82 |  |
| TXN2 | 22 | q12.3 | 1.27E-03 | 1.44E-02 | 0.46 |  | 0.88 |  |
| RABL4 | 22 | q12.3 | 2.53E-03 | 2.40E-02 | 0.43 |  | 0.83 |  |
| MPST | 22 | q12.3 | 2.72E-03 | 2.53E-02 | 0.43 |  | 0.87 |  |
| POLR2F | 22 | q13.1 | 7.79E-07 | 5.22E-05 | 0.65 |  | 0.73 |  |
| SMCR7L | 22 | q13.1 | 2.54E-06 | 1.27E-04 | 0.63 |  | 0.82 |  |
| NOL12 | 22 | q13.1 | 6.88E-06 | 2.75E-04 | 0.60 |  | 0.80 |  |
| CBY1 | 22 | q13.1 | 1.89E-05 | 5.89E-04 | 0.58 |  | 0.76 |  |
| TOMM22 | 22 | q13.1 | 2.22E-05 | 6.68E-04 | 0.58 |  | 0.84 |  |
| MICALL1 | 22 | q13.1 | 6.85E-05 | 1.60E-03 | 0.55 |  | 0.82 |  |
| ANKRD54 | 22 | q13.1 | 7.86E-05 | 1.79E-03 | 0.54 |  | 0.84 |  |
| RPS19BP1 | 22 | q13.1 | 1.32E-04 | 2.65E-03 | 0.53 |  | 0.79 |  |
| MAP3K7IP1 | 22 | q13.1 | 1.63E-04 | 3.07E-03 | 0.52 |  | 0.89 |  |
| EIF3EIP | 22 | q13.1 | 4.81E-04 | 7.07E-03 | 0.49 |  | 0.80 |  |
| CSNK1E | 22 | q13.1 | 5.15E-04 | 7.45E-03 | 0.49 |  | 0.74 |  |
| GCAT | 22 | q13.1 | 5.21E-04 | 7.50E-03 | 0.49 |  | 0.82 |  |
| UNC84B | 22 | q13.1 | 1.59E-03 | 1.70E-02 | 0.45 |  | 0.88 |  |
| JOSD1 | 22 | q13.1 | 1.87E-03 | 1.93E-02 | 0.44 |  | 0.92 |  |
| TNRC6B | 22 | q13.1 | 3.93E-03 | 3.27E-02 | 0.41 |  | 0.79 |  |
| PLA2G6 | 22 | q13.1 | 5.55E-03 | 4.20E-02 | -0.40 |  | 1.03 |  |
| CDC42EP1 | 22 | q13.1 | 5.81E-03 | 4.35E-02 | 0.40 |  | 0.90 |  |
| TSPO | 22 | q13.2 | 3.26E-06 | 1.53E-04 | 0.62 |  | 0.86 |  |
| POLDIP3 | 22 | q13.2 | 2.67E-05 | 7.76E-04 | 0.57 |  | 0.85 |  |
| NHP2L1 | 22 | q13.2 | 7.36E-05 | 1.71E-03 | 0.55 |  | 0.87 |  |
| NDUFA6 | 22 | q13.2 | 7.87E-05 | 1.79E-03 | 0.54 |  | 0.80 |  |
| ARFGAP3 | 22 | q13.2 | 3.22E-04 | 5.20E-03 | 0.50 |  | 0.87 |  |
| PHF5A | 22 | q13.2 | 5.63E-04 | 7.99E-03 | 0.48 |  | 0.91 |  |
| C22orf32 | 22 | q13.2 | 1.16E-03 | 1.35E-02 | 0.46 |  | 0.79 |  |
| EP300 | 22 | q13.2 | 1.63E-03 | 1.73E-02 | 0.45 |  | 0.92 |  |
| CENPM | 22 | q13.2 | 2.13E-03 | 2.12E-02 | 0.44 |  | 0.96 |  |
| RANGAP1 | 22 | q13.2 | 2.66E-03 | 2.48E-02 | 0.43 |  | 0.90 |  |
| RRP7A | 22 | q13.2 | 3.52E-03 | 3.03E-02 | 0.42 |  | 0.87 |  |
| ST13 | 22 | q13.2 | 4.19E-03 | 3.42E-02 | 0.41 |  | 0.86 |  |
| SREBF2 | 22 | q13.2 | 4.51E-03 | 3.61E-02 | 0.41 |  | 0.89 |  |
| TRMU | 22 | q13.31 | 8.55E-04 | 1.07E-02 | 0.47 |  | 0.85 |  |
| TTC38 | 22 | q13.31 | 1.25E-03 | 1.42E-02 | 0.46 |  | 0.78 |  |
| PRR5 | 22 | q13.31 | 1.48E-03 | 1.61E-02 | 0.45 |  | 0.90 |  |
| LDOC1L | 22 | q13.31 | 2.23E-03 | 2.18E-02 | 0.44 |  | 0.82 |  |
| TBC1D22A | 22 | q13.31 | 3.54E-03 | 3.04E-02 | 0.42 |  | 0.90 |  |
| SBF1 | 22 | q13.33 | 2.52E-05 | 7.39E-04 | 0.57 |  | 0.79 |  |
| TRABD | 22 | q13.33 | 5.95E-05 | 1.44E-03 | 0.55 |  | 0.74 |  |
| BRD1 | 22 | q13.33 | 2.50E-04 | 4.28E-03 | 0.51 |  | 0.87 |  |
| RP3-402G11.5 | 22 | q13.33 | 2.04E-03 | 2.06E-02 | 0.44 |  | 0.91 |  |
| PIM3 | 22 | q13.33 | 3.04E-03 | 2.73E-02 | 0.42 |  | 0.81 |  |
| ZBED4 | 22 | q13.33 | 4.31E-03 | 3.49E-02 | 0.41 |  | 0.90 |  |
| CRELD2 | 22 | q13.33 | 6.44E-03 | 4.68E-02 | 0.39 |  | 0.91 |  |
| RRAGB | 23 | p11.1 | 1.38E-04 | 2.73E-03 | 0.53 | 1.50 | 0.62 |  |
| MAGED2 | 23 | p11.21 | 6.65E-03 | 4.78E-02 | 0.39 | 1.29 | 0.91 |  |
| ELK1 | 23 | p11.23 | 4.31E-03 | 3.49E-02 | -0.41 | 0.93 | 1.26 |  |
| MED14 | 23 | p11.4 | 3.72E-03 | 3.16E-02 | 0.42 | 1.12 | 0.90 |  |
| DDX3X | 23 | p11.4 | 6.87E-03 | 4.91E-02 | 0.39 | 1.15 | 0.73 |  |
| TRAPPC2 | 23 | p22.2 | 1.20E-03 | 1.38E-02 | 0.46 | 1.11 | 0.92 |  |
| HDHD1A | 23 | p22.31 | 3.14E-03 | 2.80E-02 | 0.42 | 1.33 | 0.79 |  |
| DHRSX | 23 | p22.33 | 1.59E-03 | 1.70E-02 | 0.45 | 2.20 | 0.83 |  |
| RP11-706O15.1 | 23 | p22.33 | 2.12E-03 | 2.11E-02 | 0.44 | 1.49 | 0.81 |  |
| ARSD | 23 | p22.33 | 2.24E-03 | 2.19E-02 | 0.44 | 2.62 | 0.73 |  |
| CRLF2 | 23 | p22.33 | 4.20E-03 | 3.43E-02 | -0.41 |  | 1.05 |  |
| GTPBP6 | 23 | p22.33 | 5.69E-03 | 4.28E-02 | 0.40 | 1.32 | 0.85 |  |
| XIST | 23 | q13.2 | 2.06E-04 | 3.72E-03 | 0.52 | 1.15 | 0.39 |  |
| ARMCX5 | 23 | q22.1 | 2.41E-03 | 2.32E-02 | 0.43 | 1.09 | 0.81 |  |
| PLP1 | 23 | q22.2 | 1.93E-03 | 1.98E-02 | -0.44 | 0.97 | 1.06 |  |
| TSC22D3 | 23 | q22.3 | 5.97E-04 | 8.31E-03 | 0.48 | 1.16 | 0.93 |  |
| CXorf41 | 23 | q22.3 | 3.30E-03 | 2.89E-02 | -0.42 | 0.96 | 1.04 |  |
| ALG13 | 23 | q23 | 5.18E-05 | 1.29E-03 | 0.55 | 1.17 | 0.84 |  |
| NDUFA1 | 23 | q24 | 5.81E-03 | 4.34E-02 | 0.40 | 1.49 | 0.82 |  |
| RBMX2 | 23 | q26.1 | 1.73E-03 | 1.82E-02 | 0.44 | 1.18 | 0.95 |  |
| ZNF75D | 23 | q26.3 | 9.40E-04 | 1.14E-02 | 0.47 | 1.19 | 0.91 |  |
| MAGEA10 | 23 | q28 | 8.93E-04 | 1.11E-02 | -0.47 | 0.96 | 1.04 |  |
| MTM1 | 23 | q28 | 3.00E-03 | 2.71E-02 | 0.42 | 1.26 | 0.88 |  |
| CYorf14 | 24 | q11.222 | 5.76E-03 | 4.32E-02 | 0.40 | 1.04 |  |  |

**Table S5. List of 628 genes that are significantly overexpressed when amplified, derived from a gene by gene Wilcoxon analysis of expression data from 47 ER-positive breast cancer samples using aCGH copy number states as a grouping variable.**

| **Symbol** | **Chromosome** | **Cytoband** | **Number Amp cases** | **Amp Fold** | **Wilcox p value** |
| --- | --- | --- | --- | --- | --- |
| FAM72B | 1 | p11.2 | 2 | 0.69 | 0.0019 |
| FCGR1B | 1 | p11.2 | 2 | 0.91 | 0.0074 |
| NOTCH2 | 1 | p12 | 7 | 1.68 | 0.0102 |
| ADAM30 | 1 | p12 | 2 | 1.07 | 0.0463 |
| NGF | 1 | p13.2 | 2 | 1.13 | 0.0037 |
| PPIH | 1 | p34.2 | 2 | 2.37 | 0.0019 |
| C1orf50 | 1 | p34.2 | 2 | 2.23 | 0.0037 |
| FOXJ3 | 1 | p34.2 | 2 | 2.39 | 0.0037 |
| PPCS | 1 | p34.2 | 2 | 2.31 | 0.0037 |
| BCL9 | 1 | q21.1 | 8 | 1.96 | 0.0001 |
| ACP6 | 1 | q21.1 | 8 | 1.93 | 0.0001 |
| CHD1L | 1 | q21.1 | 8 | 1.43 | 0.0077 |
| FMO5 | 1 | q21.1 | 8 | 1.79 | 0.0122 |
| NBPF9 | 1 | q21.1 | 5 | 1.10 | 0.0182 |
| PRKAB2 | 1 | q21.1 | 8 | 1.25 | 0.0306 |
| AL592284.2 | 1 | q21.1 | 5 | 1.25 | 0.0307 |
| PEX11B | 1 | q21.1 | 5 | 1.29 | 0.0372 |
| AL356004.9-2 | 1 | q21.1 | 8 | 1.06 | 0.0478 |
| PDIA3P | 1 | q21.1 | 8 | 1.19 | 0.0478 |
| VPS45 | 1 | q21.2 | 3 | 2.02 | 0.0102 |
| NBPF14 | 1 | q21.2 | 8 | 1.19 | 0.0306 |
| BOLA1 | 1 | q21.2 | 5 | 1.50 | 0.0448 |
| MRPL9 | 1 | q21.3 | 7 | 1.46 | 0.0004 |
| ENSA | 1 | q21.3 | 3 | 3.21 | 0.0009 |
| SNX27 | 1 | q21.3 | 7 | 1.52 | 0.0009 |
| BNIPL | 1 | q21.3 | 3 | 1.87 | 0.0020 |
| SNAPIN | 1 | q21.3 | 4 | 1.63 | 0.0040 |
| SHC1 | 1 | q21.3 | 3 | 1.76 | 0.0065 |
| LCE6A | 1 | q21.3 | 8 | 0.92 | 0.0174 |
| CTSK | 1 | q21.3 | 3 | 1.91 | 0.0252 |
| SPRR2E | 1 | q21.3 | 8 | 0.94 | 0.0262 |
| ARNT | 1 | q21.3 | 3 | 1.86 | 0.0292 |
| THEM4 | 1 | q21.3 | 8 | 1.16 | 0.0357 |
| MUC1 | 1 | q22 | 2 | 3.63 | 0.0019 |
| PAQR6 | 1 | q22 | 2 | 1.13 | 0.0167 |
| SSR2 | 1 | q22 | 2 | 1.92 | 0.0222 |
| CCT3 | 1 | q22 | 2 | 1.98 | 0.0296 |
| EFNA1 | 1 | q22 | 2 | 2.21 | 0.0370 |
| KIAA0907 | 1 | q22 | 2 | 1.68 | 0.0463 |
| ARHGEF11 | 1 | q23.1 | 5 | 1.29 | 0.0115 |
| MNDA | 1 | q23.1 | 8 | 0.69 | 0.0174 |
| ISG20L2 | 1 | q23.1 | 3 | 1.38 | 0.0215 |
| C1orf66 | 1 | q23.1 | 3 | 1.63 | 0.0292 |
| AIM2 | 1 | q23.1-q23.2 | 7 | 0.78 | 0.0330 |
| NCSTN | 1 | q23.2 | 7 | 1.54 | 0.0002 |
| VANGL2 | 1 | q23.2 | 7 | 2.44 | 0.0027 |
| WDR42A | 1 | q23.2 | 7 | 1.47 | 0.0031 |
| RP11-226L15.1 | 1 | q23.2 | 7 | 1.09 | 0.0075 |
| PEX19 | 1 | q23.2 | 7 | 1.35 | 0.0125 |
| COPA | 1 | q23.2 | 7 | 1.37 | 0.0137 |
| SLAMF6 | 1 | q23.2 | 7 | 0.85 | 0.0151 |
| PIGM | 1 | q23.2 | 7 | 1.47 | 0.0165 |
| IGSF9 | 1 | q23.2 | 7 | 1.67 | 0.0236 |
| PEA15 | 1 | q23.2 | 7 | 1.25 | 0.0419 |
| CCDC19 | 1 | q23.2 | 7 | 1.27 | 0.0488 |
| PPOX | 1 | q23.3 | 5 | 1.71 | 0.0001 |
| USP21 | 1 | q23.3 | 5 | 1.64 | 0.0003 |
| DUSP12 | 1 | q23.3 | 7 | 1.40 | 0.0006 |
| ATF6 | 1 | q23.3 | 7 | 1.38 | 0.0014 |
| SDHC | 1 | q23.3 | 4 | 1.93 | 0.0017 |
| UFC1 | 1 | q23.3 | 5 | 1.50 | 0.0061 |
| KLHDC9 | 1 | q23.3 | 5 | 1.82 | 0.0061 |
| UAP1 | 1 | q23.3 | 7 | 1.21 | 0.0125 |
| PBX1 | 1 | q23.3 | 7 | 1.53 | 0.0387 |
| NDUFS2 | 1 | q23.3 | 5 | 1.25 | 0.0409 |
| TMCO1 | 1 | q24.1 | 7 | 1.80 | 0.0003 |
| ALDH9A1 | 1 | q24.1 | 7 | 1.57 | 0.0016 |
| POGK | 1 | q24.1 | 6 | 1.43 | 0.0076 |
| MGST3 | 1 | q24.1 | 7 | 1.31 | 0.0198 |
| FAM78B | 1 | q24.1 | 7 | 0.95 | 0.0216 |
| GORAB | 1 | q24.2 | 7 | 1.39 | 0.0003 |
| SCYL3 | 1 | q24.2 | 8 | 1.57 | 0.0004 |
| ATP1B1 | 1 | q24.2 | 7 | 2.46 | 0.0008 |
| IQWD1 | 1 | q24.2 | 7 | 1.77 | 0.0011 |
| MPZL1 | 1 | q24.2 | 7 | 1.54 | 0.0039 |
| RP1-206D15.3 | 1 | q24.2 | 8 | 1.05 | 0.0042 |
| NME7 | 1 | q24.2 | 7 | 1.44 | 0.0049 |
| C1orf112 | 1 | q24.2 | 8 | 1.61 | 0.0063 |
| BLZF1 | 1 | q24.2 | 8 | 1.44 | 0.0102 |
| BRP44 | 1 | q24.2 | 7 | 1.50 | 0.0137 |
| C1orf156 | 1 | q24.2 | 8 | 1.29 | 0.0223 |
| F5 | 1 | q24.2 | 8 | 0.91 | 0.0284 |
| TIPRL | 1 | q24.2 | 7 | 1.30 | 0.0330 |
| SLC19A2 | 1 | q24.2 | 8 | 1.06 | 0.0414 |
| SELL | 1 | q24.2 | 8 | 0.78 | 0.0445 |
| PIGC | 1 | q24.3 | 9 | 1.66 | 0.0002 |
| VAMP4 | 1 | q24.3 | 9 | 1.67 | 0.0004 |
| METTL13 | 1 | q24.3 | 9 | 1.63 | 0.0007 |
| DARS2 | 1 | q25.1 | 9 | 1.74 | 0.0002 |
| KIAA0040 | 1 | q25.1 | 9 | 1.61 | 0.0028 |
| MRPS14 | 1 | q25.1 | 9 | 1.27 | 0.0257 |
| KLHL20 | 1 | q25.1 | 9 | 1.26 | 0.0257 |
| SERPINC1 | 1 | q25.1 | 9 | 1.08 | 0.0257 |
| PRDX6 | 1 | q25.1 | 9 | 1.35 | 0.0321 |
| ZBTB37 | 1 | q25.1 | 9 | 1.06 | 0.0345 |
| CENPL | 1 | q25.1 | 9 | 1.31 | 0.0370 |
| RP11-222A5.1 | 1 | q25.1 | 9 | 0.97 | 0.0370 |
| RFWD2 | 1 | q25.1-q25.2 | 9 | 1.39 | 0.0001 |
| RALGPS2 | 1 | q25.2 | 7 | 1.34 | 0.0003 |
| CEP350 | 1 | q25.2 | 7 | 1.56 | 0.0003 |
| FAM20B | 1 | q25.2 | 7 | 1.53 | 0.0035 |
| TOR3A | 1 | q25.2 | 7 | 1.45 | 0.0093 |
| TOR1AIP1 | 1 | q25.2 | 7 | 1.60 | 0.0102 |
| RP11-545A16.3 | 1 | q25.2 | 8 | 1.05 | 0.0331 |
| C1orf26 | 1 | q25.3 | 8 | 1.46 | 0.0000 |
| STX6 | 1 | q25.3 | 7 | 1.44 | 0.0011 |
| FAM129A | 1 | q25.3 | 8 | 2.64 | 0.0013 |
| C1orf25 | 1 | q25.3 | 8 | 1.28 | 0.0034 |
| GS1-115G20.1 | 1 | q25.3 | 8 | 1.18 | 0.0052 |
| RNASEL | 1 | q25.3 | 8 | 1.26 | 0.0052 |
| GLUL | 1 | q25.3 | 8 | 1.22 | 0.0077 |
| TSEN15 | 1 | q25.3 | 8 | 1.43 | 0.0122 |
| MR1 | 1 | q25.3 | 7 | 1.45 | 0.0198 |
| IVNS1ABP | 1 | q25.3 | 8 | 1.25 | 0.0223 |
| DHX9 | 1 | q25.3 | 8 | 1.14 | 0.0242 |
| SMG7 | 1 | q25.3 | 9 | 1.21 | 0.0321 |
| LAMC1 | 1 | q25.3 | 8 | 1.44 | 0.0357 |
| C1orf21 | 1 | q25.3 | 8 | 1.30 | 0.0445 |
| TPR | 1 | q31.1 | 8 | 1.61 | 0.0000 |
| C1orf27 | 1 | q31.1 | 8 | 1.24 | 0.0024 |
| FAM5C | 1 | q31.1 | 3 | 3.49 | 0.0038 |
| ZBTB41 | 1 | q31.3 | 6 | 1.51 | 0.0262 |
| CFHR3 | 1 | q31.3 | 8 | 0.95 | 0.0306 |
| DDX59 | 1 | q32.1 | 8 | 1.48 | 0.0015 |
| LGTN | 1 | q32.1 | 8 | 1.58 | 0.0038 |
| ZNF281 | 1 | q32.1 | 8 | 1.55 | 0.0112 |
| CYB5R1 | 1 | q32.1 | 6 | 1.50 | 0.0119 |
| NUCKS1 | 1 | q32.1 | 7 | 1.43 | 0.0137 |
| JARID1B | 1 | q32.1 | 6 | 1.57 | 0.0146 |
| SOX13 | 1 | q32.1 | 6 | 1.59 | 0.0162 |
| PLEKHA6 | 1 | q32.1 | 7 | 1.82 | 0.0198 |
| CTSE | 1 | q32.1 | 7 | 0.95 | 0.0236 |
| LAD1 | 1 | q32.1 | 6 | 1.10 | 0.0239 |
| ATP2B4 | 1 | q32.1 | 6 | 1.47 | 0.0262 |
| RNPEP | 1 | q32.1 | 6 | 1.48 | 0.0314 |
| KLHL12 | 1 | q32.1 | 6 | 1.43 | 0.0343 |
| RP11-31E23.1 | 1 | q32.1 | 9 | 1.05 | 0.0370 |
| KIF14 | 1 | q32.1 | 8 | 1.25 | 0.0414 |
| AVPR1B | 1 | q32.1 | 7 | 1.04 | 0.0419 |
| IPO9 | 1 | q32.1 | 6 | 1.30 | 0.0443 |
| SRGAP2 | 1 | q32.1 | 7 | 1.26 | 0.0452 |
| RCOR3 | 1 | q32.2 | 8 | 1.93 | 0.0000 |
| C1orf74 | 1 | q32.2 | 9 | 1.44 | 0.0002 |
| HHAT | 1 | q32.2 | 9 | 1.54 | 0.0003 |
| CD46 | 1 | q32.2 | 9 | 1.73 | 0.0018 |
| TRAF3IP3 | 1 | q32.2 | 9 | 0.80 | 0.0025 |
| IRF6 | 1 | q32.2 | 9 | 1.56 | 0.0028 |
| SYT14 | 1 | q32.2 | 9 | 0.95 | 0.0056 |
| KCNH1 | 1 | q32.2 | 8 | 1.05 | 0.0102 |
| hsa-mir-29c | 1 | q32.2 | 9 | 1.46 | 0.0277 |
| TRAF5 | 1 | q32.2-q32.3 | 7 | 1.79 | 0.0075 |
| C1orf97 | 1 | q32.3 | 7 | 1.57 | 0.0027 |
| LPGAT1 | 1 | q32.3 | 7 | 1.53 | 0.0027 |
| NSL1 | 1 | q32.3 | 7 | 1.54 | 0.0031 |
| SLC30A1 | 1 | q32.3 | 7 | 1.35 | 0.0031 |
| ANGEL2 | 1 | q32.3 | 7 | 1.45 | 0.0049 |
| TATDN3 | 1 | q32.3 | 7 | 1.25 | 0.0055 |
| INTS7 | 1 | q32.3 | 7 | 1.31 | 0.0075 |
| RPS6KC1 | 1 | q32.3 | 7 | 1.25 | 0.0102 |
| SNFT | 1 | q32.3 | 7 | 0.87 | 0.0113 |
| PPP2R5A | 1 | q32.3 | 7 | 1.45 | 0.0257 |
| ATF3 | 1 | q32.3 | 7 | 0.69 | 0.0358 |
| NENF | 1 | q32.3 | 7 | 1.17 | 0.0419 |
| PROX1 | 1 | q32.3 | 10 | 0.93 | 0.0486 |
| IARS2 | 1 | q41 | 9 | 1.70 | 0.0001 |
| GPATCH2 | 1 | q41 | 9 | 1.56 | 0.0002 |
| RAB3GAP2 | 1 | q41 | 10 | 1.46 | 0.0004 |
| ESRRG | 1 | q41 | 9 | 2.49 | 0.0004 |
| CAPN8 | 1 | q41 | 5 | 3.48 | 0.0015 |
| SPATA17 | 1 | q41 | 9 | 1.30 | 0.0038 |
| EPRS | 1 | q41 | 9 | 1.37 | 0.0038 |
| KCTD3 | 1 | q41 | 10 | 1.68 | 0.0040 |
| TP53BP2 | 1 | q41 | 5 | 1.46 | 0.0079 |
| DISP1 | 1 | q41 | 7 | 1.21 | 0.0181 |
| CENPF | 1 | q41 | 10 | 1.45 | 0.0212 |
| MOSC1 | 1 | q41 | 10 | 1.42 | 0.0229 |
| RP11-365D23.1 | 1 | q41 | 10 | 0.82 | 0.0264 |
| MOSC2 | 1 | q41 | 10 | 1.37 | 0.0326 |
| CAPN2 | 1 | q41 | 5 | 1.38 | 0.0338 |
| RP11-176D17.3 | 1 | q41 | 10 | 1.13 | 0.0399 |
| LYPLAL1 | 1 | q41 | 9 | 1.27 | 0.0426 |
| BPNT1 | 1 | q41 | 9 | 1.33 | 0.0456 |
| WDR26 | 1 | q42.11-q42.12 | 5 | 1.37 | 0.0182 |
| C1orf55 | 1 | q42.12 | 4 | 1.40 | 0.0189 |
| LEFTY1 | 1 | q42.12 | 4 | 1.39 | 0.0341 |
| PARP1 | 1 | q42.12 | 6 | 1.26 | 0.0443 |
| NUP133 | 1 | q42.13 | 7 | 1.49 | 0.0003 |
| MRPL55 | 1 | q42.13 | 6 | 1.44 | 0.0076 |
| ARF1 | 1 | q42.13 | 6 | 1.31 | 0.0107 |
| C1orf69 | 1 | q42.13 | 6 | 1.20 | 0.0107 |
| SNAP47 | 1 | q42.13 | 5 | 1.36 | 0.0130 |
| URB2 | 1 | q42.13 | 7 | 1.25 | 0.0151 |
| C1orf35 | 1 | q42.13 | 6 | 1.38 | 0.0198 |
| HIST3H3 | 1 | q42.13 | 6 | 0.94 | 0.0198 |
| CABC1 | 1 | q42.13 | 6 | 1.29 | 0.0217 |
| RHOU | 1 | q42.13 | 6 | 0.55 | 0.0239 |
| C1orf57 | 1 | q42.2 | 7 | 1.76 | 0.0007 |
| COG2 | 1 | q42.2 | 6 | 1.55 | 0.0010 |
| TARBP1 | 1 | q42.2 | 7 | 1.68 | 0.0027 |
| GNPAT | 1 | q42.2 | 7 | 1.35 | 0.0068 |
| EXOC8 | 1 | q42.2 | 7 | 1.36 | 0.0068 |
| DISC1 | 1 | q42.2 | 7 | 1.07 | 0.0068 |
| C1orf131 | 1 | q42.2 | 7 | 1.41 | 0.0075 |
| ARV1 | 1 | q42.2 | 6 | 1.39 | 0.0198 |
| TTC13 | 1 | q42.2 | 6 | 1.30 | 0.0239 |
| GGPS1 | 1 | q42.3 | 6 | 1.31 | 0.0132 |
| TOMM20 | 1 | q42.3 | 6 | 1.41 | 0.0262 |
| B3GALNT2 | 1 | q42.3 | 6 | 1.15 | 0.0481 |
| SDCCAG8 | 1 | q43 | 6 | 1.18 | 0.0287 |
| FH | 1 | q43 | 7 | 1.38 | 0.0304 |
| HEATR1 | 1 | q43 | 7 | 1.32 | 0.0387 |
| MAP1LC3C | 1 | q43 | 7 | 1.10 | 0.0452 |
| AHCTF1 | 1 | q44 | 8 | 1.33 | 0.0000 |
| ZNF672 | 1 | q44 | 11 | 1.49 | 0.0004 |
| PPPDE1 | 1 | q44 | 6 | 1.54 | 0.0032 |
| C1orf229 | 1 | q44 | 8 | 1.18 | 0.0057 |
| SH3BP5L | 1 | q44 | 11 | 1.45 | 0.0062 |
| ZNF692 | 1 | q44 | 11 | 1.42 | 0.0073 |
| ZNF669 | 1 | q44 | 8 | 1.15 | 0.0085 |
| PGBD2 | 1 | q44 | 11 | 1.17 | 0.0109 |
| FAM36A | 1 | q44 | 6 | 1.45 | 0.0119 |
| HNRNPU | 1 | q44 | 6 | 1.32 | 0.0179 |
| ZNF238 | 1 | q44 | 7 | 1.04 | 0.0236 |
| TFB2M | 1 | q44 | 8 | 1.36 | 0.0357 |
| EFCAB2 | 1 | q44 | 6 | 1.15 | 0.0481 |
| SERF1B | 5 | q13.2 | 2 | 2.08 | 0.0167 |
| SEC63 | 6 | q21 | 2 | 3.35 | 0.0019 |
| FOXO3 | 6 | q21 | 2 | 3.55 | 0.0019 |
| SNX3 | 6 | q21 | 2 | 1.23 | 0.0019 |
| QRSL1 | 6 | q21 | 2 | 1.58 | 0.0019 |
| LACE1 | 6 | q21 | 2 | 1.55 | 0.0037 |
| C6orf203 | 6 | q21 | 2 | 2.08 | 0.0037 |
| PDSS2 | 6 | q21 | 2 | 2.09 | 0.0074 |
| OSTM1 | 6 | q21 | 2 | 2.10 | 0.0074 |
| AIM1 | 6 | q21 | 2 | 2.36 | 0.0074 |
| RTN4IP1 | 6 | q21 | 2 | 2.54 | 0.0074 |
| C6orf185 | 6 | q21 | 2 | 1.93 | 0.0111 |
| HIVEP2 | 6 | q24.2 | 2 | 2.61 | 0.0019 |
| PEX3 | 6 | q24.2 | 2 | 2.23 | 0.0037 |
| AIG1 | 6 | q24.2 | 2 | 2.24 | 0.0074 |
| FUCA2 | 6 | q24.2 | 2 | 2.11 | 0.0463 |
| HGSNAT | 8 | p11.1 | 2 | 1.31 | 0.0167 |
| FNTA | 8 | p11.1 | 2 | 1.96 | 0.0296 |
| C8orf40 | 8 | p11.21 | 2 | 2.27 | 0.0037 |
| GINS4 | 8 | p11.21 | 3 | 1.51 | 0.0065 |
| GOLGA7 | 8 | p11.21 | 3 | 2.49 | 0.0083 |
| RNF170 | 8 | p11.21 | 2 | 1.91 | 0.0222 |
| AP3M2 | 8 | p11.21 | 2 | 1.97 | 0.0296 |
| VDAC3 | 8 | p11.21 | 2 | 1.73 | 0.0463 |
| TM2D2 | 8 | p11.22 | 4 | 1.93 | 0.0080 |
| LSM1 | 8 | p11.23 | 4 | 4.38 | 0.0008 |
| PROSC | 8 | p11.23 | 4 | 2.71 | 0.0008 |
| BRF2 | 8 | p11.23 | 4 | 3.56 | 0.0011 |
| ASH2L | 8 | p11.23 | 4 | 3.10 | 0.0011 |
| ZNF703 | 8 | p11.23 | 5 | 2.68 | 0.0021 |
| ERLIN2 | 8 | p11.23 | 5 | 1.80 | 0.0053 |
| WHSC1L1 | 8 | p11.23 | 5 | 1.58 | 0.0090 |
| DDHD2 | 8 | p11.23 | 4 | 2.55 | 0.0126 |
| RAB11FIP1 | 8 | p11.23 | 3 | 2.24 | 0.0152 |
| LETM2 | 8 | p11.23 | 5 | 1.14 | 0.0278 |
| BAG4 | 8 | p11.23 | 4 | 2.27 | 0.0305 |
| UBE2V2 | 8 | q11.21 | 2 | 2.26 | 0.0074 |
| AC013701.6 | 8 | q11.21 | 2 | 1.15 | 0.0222 |
| ATP6V1H | 8 | q11.23 | 2 | 2.38 | 0.0111 |
| TCEA1 | 8 | q11.23 | 2 | 1.55 | 0.0111 |
| RB1CC1 | 8 | q11.23 | 2 | 1.65 | 0.0222 |
| RAB2 | 8 | q12.1 | 4 | 1.74 | 0.0080 |
| IMPAD1 | 8 | q12.1 | 3 | 1.59 | 0.0102 |
| MOS | 8 | q12.1 | 2 | 0.78 | 0.0167 |
| TGS1 | 8 | q12.1 | 2 | 1.13 | 0.0222 |
| SDCBP | 8 | q12.1 | 6 | 1.51 | 0.0408 |
| YTHDF3 | 8 | q12.3 | 3 | 1.72 | 0.0038 |
| ASPH | 8 | q12.3 | 3 | 1.26 | 0.0126 |
| COPS5 | 8 | q13.1 | 4 | 2.12 | 0.0001 |
| RRS1 | 8 | q13.1 | 4 | 2.39 | 0.0011 |
| VCPIP1 | 8 | q13.1 | 4 | 1.38 | 0.0094 |
| MTFR1 | 8 | q13.1 | 4 | 1.67 | 0.0166 |
| SGK3 | 8 | q13.1 | 4 | 1.05 | 0.0272 |
| PDE7A | 8 | q13.1 | 4 | 1.16 | 0.0469 |
| CSPP1 | 8 | q13.1-q13.2 | 4 | 1.23 | 0.0109 |
| ARFGEF1 | 8 | q13.2 | 4 | 2.02 | 0.0006 |
| LACTB2 | 8 | q13.3 | 3 | 2.74 | 0.0002 |
| TRAM1 | 8 | q13.3 | 3 | 1.50 | 0.0038 |
| NCOA2 | 8 | q13.3 | 3 | 1.14 | 0.0102 |
| PXMP3 | 8 | q21.11 | 4 | 2.07 | 0.0000 |
| UBE2W | 8 | q21.11 | 3 | 1.89 | 0.0001 |
| STAU2 | 8 | q21.11 | 3 | 1.86 | 0.0014 |
| TCEB1 | 8 | q21.11 | 3 | 1.94 | 0.0028 |
| GDAP1 | 8 | q21.11 | 4 | 1.13 | 0.0423 |
| MRPS28 | 8 | q21.13 | 4 | 1.90 | 0.0008 |
| ZFAND1 | 8 | q21.13 | 3 | 1.94 | 0.0038 |
| ZBTB10 | 8 | q21.13 | 3 | 1.26 | 0.0083 |
| HEY1 | 8 | q21.13 | 4 | 1.71 | 0.0094 |
| ZNF704 | 8 | q21.13 | 3 | 1.09 | 0.0387 |
| E2F5 | 8 | q21.2 | 3 | 2.04 | 0.0038 |
| OTUD6B | 8 | q21.3 | 4 | 1.62 | 0.0003 |
| FAM82B | 8 | q21.3 | 4 | 1.71 | 0.0014 |
| TMEM55A | 8 | q21.3 | 4 | 1.83 | 0.0014 |
| DECR1 | 8 | q21.3 | 3 | 1.73 | 0.0020 |
| SLC7A13 | 8 | q21.3 | 4 | 1.12 | 0.0048 |
| OSGIN2 | 8 | q21.3 | 3 | 1.50 | 0.0126 |
| RIPK2 | 8 | q21.3 | 3 | 1.79 | 0.0252 |
| NBN | 8 | q21.3 | 3 | 1.57 | 0.0252 |
| TMEM67 | 8 | q22.1 | 3 | 1.40 | 0.0009 |
| CCNE2 | 8 | q22.1 | 3 | 2.11 | 0.0020 |
| DPY19L4 | 8 | q22.1 | 2 | 1.77 | 0.0037 |
| PPM2C | 8 | q22.1 | 3 | 1.77 | 0.0038 |
| LAPTM4B | 8 | q22.1 | 4 | 2.75 | 0.0040 |
| PTDSS1 | 8 | q22.1 | 3 | 1.92 | 0.0065 |
| TP53INP1 | 8 | q22.1 | 3 | 2.26 | 0.0102 |
| RAD54B | 8 | q22.1 | 2 | 1.53 | 0.0111 |
| GEM | 8 | q22.1 | 2 | 1.40 | 0.0111 |
| C8orf38 | 8 | q22.1 | 4 | 1.93 | 0.0145 |
| TSPYL5 | 8 | q22.1 | 4 | 2.67 | 0.0145 |
| MTERFD1 | 8 | q22.1 | 3 | 1.83 | 0.0252 |
| KIAA1429 | 8 | q22.1 | 3 | 1.41 | 0.0292 |
| MTDH | 8 | q22.1 | 4 | 1.73 | 0.0305 |
| RPL30 | 8 | q22.2 | 4 | 1.76 | 0.0008 |
| ANKRD46 | 8 | q22.2 | 4 | 2.23 | 0.0014 |
| SPAG1 | 8 | q22.2 | 4 | 1.31 | 0.0040 |
| HRSP12 | 8 | q22.2 | 4 | 1.94 | 0.0094 |
| STK3 | 8 | q22.2 | 4 | 1.73 | 0.0272 |
| RNF19A | 8 | q22.2 | 4 | 1.39 | 0.0272 |
| OSR2 | 8 | q22.2 | 4 | 1.06 | 0.0381 |
| VPS13B | 8 | q22.2 | 4 | 1.08 | 0.0381 |
| NIPAL2 | 8 | q22.2 | 4 | 1.20 | 0.0381 |
| COX6C | 8 | q22.2 | 4 | 1.67 | 0.0469 |
| UBR5 | 8 | q22.3 | 5 | 2.00 | 0.0001 |
| RRM2B | 8 | q22.3 | 5 | 1.74 | 0.0002 |
| AZIN1 | 8 | q22.3 | 5 | 2.30 | 0.0004 |
| ZNF706 | 8 | q22.3 | 5 | 2.41 | 0.0021 |
| YWHAZ | 8 | q22.3 | 4 | 1.27 | 0.0022 |
| BAALC | 8 | q22.3 | 5 | 1.33 | 0.0025 |
| ATP6V1C1 | 8 | q22.3 | 5 | 1.44 | 0.0251 |
| FZD6 | 8 | q22.3 | 5 | 1.98 | 0.0278 |
| SLC25A32 | 8 | q22.3 | 5 | 1.11 | 0.0278 |
| PABPCP5 | 8 | q22.3 | 4 | 1.61 | 0.0469 |
| ENY2 | 8 | q23.1 | 5 | 2.24 | 0.0000 |
| OXR1 | 8 | q23.1 | 6 | 1.74 | 0.0003 |
| NUDCD1 | 8 | q23.1 | 5 | 1.42 | 0.0115 |
| TTC35 | 8 | q23.1 | 5 | 1.31 | 0.0278 |
| EBAG9 | 8 | q23.2 | 5 | 1.89 | 0.0002 |
| AC079061.8 | 8 | q23.2 | 5 | 2.21 | 0.0372 |
| CSMD3 | 8 | q23.3 | 6 | 1.14 | 0.0198 |
| MED30 | 8 | q24.11 | 7 | 2.32 | 0.0000 |
| UTP23 | 8 | q24.11 | 7 | 1.56 | 0.0039 |
| EXT1 | 8 | q24.11 | 6 | 1.27 | 0.0217 |
| MAL2 | 8 | q24.12 | 7 | 2.24 | 0.0024 |
| MRPL13 | 8 | q24.12 | 6 | 1.84 | 0.0047 |
| TAF2 | 8 | q24.12 | 5 | 1.71 | 0.0182 |
| DSCC1 | 8 | q24.12 | 5 | 1.35 | 0.0278 |
| MTBP | 8 | q24.12 | 6 | 1.16 | 0.0481 |
| C8orf76 | 8 | q24.13 | 4 | 1.88 | 0.0006 |
| KIAA0196 | 8 | q24.13 | 6 | 1.89 | 0.0012 |
| SQLE | 8 | q24.13 | 6 | 1.69 | 0.0021 |
| DERL1 | 8 | q24.13 | 5 | 1.67 | 0.0046 |
| ATAD2 | 8 | q24.13 | 4 | 1.91 | 0.0145 |
| NSMCE2 | 8 | q24.13 | 6 | 1.54 | 0.0146 |
| WDYHV1 | 8 | q24.13 | 4 | 1.49 | 0.0214 |
| WDR67 | 8 | q24.13 | 4 | 1.36 | 0.0214 |
| TATDN1 | 8 | q24.13 | 6 | 1.64 | 0.0217 |
| ZNF572 | 8 | q24.13 | 6 | 1.10 | 0.0262 |
| ZHX2 | 8 | q24.13 | 6 | 1.44 | 0.0374 |
| AC103819.3-2 | 8 | q24.21 | 7 | 1.24 | 0.0009 |
| FAM84B | 8 | q24.21 | 6 | 2.51 | 0.0025 |
| MYC | 8 | q24.21 | 7 | 1.84 | 0.0151 |
| EFR3A | 8 | q24.22 | 6 | 1.60 | 0.0012 |
| PHF20L1 | 8 | q24.22 | 6 | 1.61 | 0.0021 |
| AF186191.6-2 | 8 | q24.22 | 5 | 1.87 | 0.0090 |
| LY6D | 8 | q24.3 | 3 | 1.36 | 0.0102 |
| CHRAC1 | 8 | q24.3 | 3 | 1.29 | 0.0387 |
| FBXO3 | 11 | p13 | 2 | 2.07 | 0.0037 |
| NAT10 | 11 | p13 | 2 | 2.07 | 0.0037 |
| CAT | 11 | p13 | 2 | 2.02 | 0.0037 |
| C11orf91 | 11 | p13 | 2 | 1.15 | 0.0167 |
| HIPK3 | 11 | p13 | 2 | 1.15 | 0.0296 |
| CAPRIN1 | 11 | p13 | 2 | 1.96 | 0.0370 |
| ZBED5 | 11 | p15.3 | 2 | 1.90 | 0.0074 |
| SAPS3 | 11 | q13.2 | 5 | 1.99 | 0.0001 |
| KDM2A | 11 | q13.2 | 3 | 3.72 | 0.0001 |
| RCE1 | 11 | q13.2 | 3 | 2.96 | 0.0001 |
| SSH3 | 11 | q13.2 | 3 | 4.82 | 0.0001 |
| CORO1B | 11 | q13.2 | 3 | 2.61 | 0.0001 |
| POLD4 | 11 | q13.2 | 3 | 4.20 | 0.0001 |
| C11orf80 | 11 | q13.2 | 3 | 5.85 | 0.0001 |
| RAD9A | 11 | q13.2 | 3 | 2.87 | 0.0002 |
| RBM4B | 11 | q13.2 | 3 | 2.37 | 0.0002 |
| PPP1CA | 11 | q13.2 | 3 | 3.75 | 0.0002 |
| RPS6KB2 | 11 | q13.2 | 3 | 3.11 | 0.0009 |
| SPTBN2 | 11 | q13.2 | 3 | 1.84 | 0.0014 |
| CABP4 | 11 | q13.2 | 2 | 6.31 | 0.0019 |
| CHKA | 11 | q13.2 | 2 | 5.03 | 0.0019 |
| NDUFS8 | 11 | q13.2 | 2 | 2.78 | 0.0019 |
| CCS | 11 | q13.2 | 2 | 4.41 | 0.0019 |
| PITPNM1 | 11 | q13.2 | 2 | 3.54 | 0.0019 |
| ANKRD13D | 11 | q13.2 | 3 | 2.57 | 0.0028 |
| ZDHHC24 | 11 | q13.2 | 2 | 4.00 | 0.0037 |
| ATPGD1 | 11 | q13.2 | 3 | 1.51 | 0.0051 |
| AIP | 11 | q13.2 | 2 | 2.10 | 0.0074 |
| LRP5 | 11 | q13.2 | 3 | 1.73 | 0.0102 |
| TMEM134 | 11 | q13.2 | 2 | 2.44 | 0.0111 |
| CDK2AP2 | 11 | q13.2 | 2 | 2.49 | 0.0167 |
| SUV420H1 | 11 | q13.2 | 2 | 1.60 | 0.0167 |
| LRFN4 | 11 | q13.2 | 3 | 2.08 | 0.0181 |
| SYT12 | 11 | q13.2 | 3 | 2.77 | 0.0252 |
| PC | 11 | q13.2 | 3 | 1.30 | 0.0252 |
| RBM14 | 11 | q13.2 | 3 | 2.15 | 0.0338 |
| ORAOV1 | 11 | q13.3 | 13 | 2.81 | 0.0000 |
| FADD | 11 | q13.3 | 13 | 2.29 | 0.0000 |
| MRPL21 | 11 | q13.3 | 9 | 2.42 | 0.0000 |
| IGHMBP2 | 11 | q13.3 | 9 | 1.52 | 0.0000 |
| PPFIA1 | 11 | q13.3 | 13 | 1.93 | 0.0000 |
| CCND1 | 11 | q13.3 | 13 | 1.73 | 0.0001 |
| CTTN | 11 | q13.3 | 13 | 1.78 | 0.0002 |
| TPCN2 | 11 | q13.3 | 12 | 1.79 | 0.0004 |
| MTL5 | 11 | q13.3 | 7 | 2.29 | 0.0006 |
| ANO1 | 11 | q13.3 | 13 | 2.23 | 0.0007 |
| MRGPRD | 11 | q13.3 | 11 | 0.96 | 0.0199 |
| SHANK2 | 11 | q13.3-q13.4 | 8 | 1.39 | 0.0384 |
| ARHGEF17 | 11 | q13.4 | 4 | 1.93 | 0.0001 |
| PAAF1 | 11 | q13.4 | 3 | 2.02 | 0.0005 |
| C2CD3 | 11 | q13.4 | 3 | 1.60 | 0.0005 |
| ATG16L2 | 11 | q13.4 | 4 | 2.35 | 0.0006 |
| CHCHD8 | 11 | q13.4 | 3 | 2.36 | 0.0009 |
| C11orf51 | 11 | q13.4 | 2 | 2.27 | 0.0019 |
| RAB6A | 11 | q13.4 | 3 | 2.12 | 0.0020 |
| UCP3 | 11 | q13.4 | 3 | 1.50 | 0.0020 |
| NADSYN1 | 11 | q13.4 | 2 | 2.96 | 0.0037 |
| MRPL48 | 11 | q13.4 | 3 | 2.01 | 0.0038 |
| C11orf59 | 11 | q13.4 | 2 | 2.17 | 0.0074 |
| FOLR1 | 11 | q13.4 | 2 | 8.49 | 0.0074 |
| DHCR7 | 11 | q13.4 | 2 | 3.27 | 0.0222 |
| CHRDL2 | 11 | q13.4 | 2 | 1.96 | 0.0222 |
| LRRC51 | 11 | q13.4 | 2 | 2.40 | 0.0296 |
| FOLR2 | 11 | q13.4 | 2 | 0.57 | 0.0370 |
| P2RY2 | 11 | q13.4 | 4 | 1.14 | 0.0381 |
| FCHSD2 | 11 | q13.4 | 4 | 1.46 | 0.0381 |
| INPPL1 | 11 | q13.4 | 2 | 1.96 | 0.0463 |
| SPCS2 | 11 | q13.4 | 2 | 1.84 | 0.0463 |
| ACER3 | 11 | q13.5 | 4 | 2.08 | 0.0189 |
| CAPN5 | 11 | q13.5 | 4 | 2.16 | 0.0214 |
| INTS4 | 11 | q14.1 | 4 | 3.05 | 0.0000 |
| RSF1 | 11 | q14.1 | 4 | 2.77 | 0.0000 |
| C11orf67 | 11 | q14.1 | 4 | 5.43 | 0.0000 |
| AQP11 | 11 | q14.1 | 4 | 3.13 | 0.0000 |
| CLNS1A | 11 | q14.1 | 4 | 3.37 | 0.0001 |
| ALG8 | 11 | q14.1 | 4 | 2.26 | 0.0014 |
| USP35 | 11 | q14.1 | 4 | 1.65 | 0.0017 |
| CCDC90B | 11 | q14.1 | 2 | 2.53 | 0.0019 |
| C11orf82 | 11 | q14.1 | 2 | 4.57 | 0.0019 |
| ANKRD42 | 11 | q14.1 | 2 | 1.83 | 0.0019 |
| ODZ4 | 11 | q14.1 | 3 | 1.13 | 0.0065 |
| RAB30 | 11 | q14.1 | 2 | 2.74 | 0.0074 |
| NARS2 | 11 | q14.1 | 5 | 1.68 | 0.0182 |
| NDUFC2 | 11 | q14.1 | 4 | 1.41 | 0.0242 |
| KCTD21 | 11 | q14.1 | 4 | 1.69 | 0.0272 |
| PCF11 | 11 | q14.1 | 2 | 1.48 | 0.0370 |
| THRSP | 11 | q14.1 | 4 | 3.29 | 0.0381 |
| CWC15 | 11 | q21 | 2 | 3.04 | 0.0222 |
| KDM4D | 11 | q21 | 2 | 1.87 | 0.0296 |
| FGFR1OP2 | 12 | p11.23 | 2 | 1.99 | 0.0019 |
| C12orf11 | 12 | p11.23 | 2 | 1.70 | 0.0222 |
| KRAS | 12 | p12.1 | 2 | 3.13 | 0.0019 |
| LYRM5 | 12 | p12.1 | 2 | 3.39 | 0.0019 |
| CASC1 | 12 | p12.1 | 2 | 1.80 | 0.0370 |
| HSPH1 | 13 | q12.3 | 2 | 2.27 | 0.0296 |
| OR4K2 | 14 | q11.2 | 2 | 1.10 | 0.0074 |
| OR4N4 | 15 | q11.2 | 3 | 0.90 | 0.0338 |
| AC074050.1 | 16 | p11.2 | 2 | 1.95 | 0.0019 |
| XPO6 | 16 | p11.2 | 2 | 2.03 | 0.0037 |
| HS3ST4 | 16 | p12.1 | 3 | 1.12 | 0.0065 |
| NSMCE1 | 16 | p12.1 | 2 | 1.87 | 0.0111 |
| JMJD5 | 16 | p12.1 | 2 | 1.21 | 0.0167 |
| GTF3C1 | 16 | p12.1 | 2 | 1.65 | 0.0463 |
| IL21R | 16 | p12.1 | 2 | 0.94 | 0.0463 |
| LITAF | 16 | p13.13 | 2 | 2.74 | 0.0019 |
| SNN | 16 | p13.13 | 2 | 1.52 | 0.0222 |
| DEXI | 16 | p13.13 | 2 | 1.51 | 0.0370 |
| C16orf75 | 16 | p13.13 | 2 | 2.00 | 0.0463 |
| WSB1 | 17 | q11.1 | 3 | 1.84 | 0.0005 |
| KIAA0100 | 17 | q11.2 | 4 | 3.11 | 0.0000 |
| FLOT2 | 17 | q11.2 | 4 | 3.96 | 0.0000 |
| ERAL1 | 17 | q11.2 | 4 | 3.91 | 0.0000 |
| DHRS13 | 17 | q11.2 | 4 | 4.69 | 0.0000 |
| SPAG5 | 17 | q11.2 | 4 | 3.77 | 0.0000 |
| PHF12 | 17 | q11.2 | 4 | 3.31 | 0.0000 |
| NEK8 | 17 | q11.2 | 4 | 2.75 | 0.0000 |
| SUPT6H | 17 | q11.2 | 4 | 2.97 | 0.0000 |
| C17orf63 | 17 | q11.2 | 4 | 3.00 | 0.0001 |
| TRAF4 | 17 | q11.2 | 4 | 4.35 | 0.0001 |
| TLCD1 | 17 | q11.2 | 4 | 4.04 | 0.0001 |
| TNFAIP1 | 17 | q11.2 | 3 | 3.90 | 0.0001 |
| IFT20 | 17 | q11.2 | 3 | 2.83 | 0.0001 |
| C17orf32 | 17 | q11.2 | 3 | 2.79 | 0.0001 |
| POLDIP2 | 17 | q11.2 | 3 | 2.22 | 0.0001 |
| SDF2 | 17 | q11.2 | 4 | 2.50 | 0.0001 |
| snoZ17 | 17 | q11.2 | 4 | 2.09 | 0.0001 |
| PIGS | 17 | q11.2 | 4 | 3.06 | 0.0001 |
| GIT1 | 17 | q11.2 | 3 | 3.82 | 0.0002 |
| NUFIP2 | 17 | q11.2 | 4 | 2.17 | 0.0003 |
| RPL23A | 17 | q11.2 | 4 | 2.25 | 0.0006 |
| TP53I13 | 17 | q11.2 | 3 | 3.03 | 0.0009 |
| AC005726.6 | 17 | q11.2 | 4 | 1.16 | 0.0011 |
| UNC119 | 17 | q11.2 | 4 | 2.22 | 0.0017 |
| ANKRD13B | 17 | q11.2 | 3 | 1.38 | 0.0028 |
| ALDOC | 17 | q11.2 | 4 | 3.78 | 0.0094 |
| PIPOX | 17 | q11.2 | 4 | 1.24 | 0.0094 |
| GOSR1 | 17 | q11.2 | 2 | 1.65 | 0.0111 |
| TAOK1 | 17 | q11.2 | 3 | 2.04 | 0.0181 |
| LGALS9 | 17 | q11.2 | 4 | 2.25 | 0.0214 |
| SNORD42 | 17 | q11.2 | 4 | 1.10 | 0.0272 |
| NLK | 17 | q11.2 | 3 | 1.90 | 0.0387 |
| SSH2 | 17 | q11.2 | 3 | 2.10 | 0.0442 |
| TCAP | 17 | q12 | 7 | 2.16 | 0.0000 |
| GRB7 | 17 | q12 | 7 | 3.44 | 0.0000 |
| PERLD1 | 17 | q12 | 7 | 5.50 | 0.0000 |
| C17orf37 | 17 | q12 | 7 | 4.76 | 0.0000 |
| ERBB2 | 17 | q12 | 7 | 2.92 | 0.0000 |
| CCDC49 | 17 | q12 | 3 | 5.84 | 0.0001 |
| PSMB3 | 17 | q12 | 3 | 4.42 | 0.0001 |
| PIP5K2B | 17 | q12 | 3 | 2.73 | 0.0001 |
| PCGF2 | 17 | q12 | 3 | 5.50 | 0.0001 |
| RPL23 | 17 | q12 | 3 | 3.36 | 0.0001 |
| STARD3 | 17 | q12 | 7 | 4.17 | 0.0003 |
| LASP1 | 17 | q12 | 3 | 3.10 | 0.0005 |
| FBXL20 | 17 | q12 | 5 | 3.67 | 0.0005 |
| MRPL45 | 17 | q12 | 3 | 2.87 | 0.0014 |
| GSDMB | 17 | q12 | 6 | 6.28 | 0.0018 |
| TBC1D3 | 17 | q12 | 3 | 1.89 | 0.0028 |
| PPARBP | 17 | q12 | 6 | 2.71 | 0.0032 |
| AC115090.8 | 17 | q12 | 3 | 1.14 | 0.0038 |
| PNMT | 17 | q12 | 7 | 2.68 | 0.0084 |
| MLLT6 | 17 | q12 | 4 | 2.64 | 0.0109 |
| TBC1D3C | 17 | q12 | 2 | 2.32 | 0.0111 |
| ZNHIT3 | 17 | q12 | 2 | 2.42 | 0.0167 |
| ORMDL3 | 17 | q12 | 6 | 4.70 | 0.0198 |
| CRKRS | 17 | q12 | 6 | 2.07 | 0.0217 |
| AC183087.2-1 | 17 | q12 | 3 | 1.34 | 0.0292 |
| GPR179 | 17 | q12 | 3 | 0.93 | 0.0387 |
| MYO19 | 17 | q12 | 2 | 1.54 | 0.0463 |
| THRA | 17 | q21.1 | 5 | 3.56 | 0.0001 |
| PSMD3 | 17 | q21.1 | 6 | 5.22 | 0.0010 |
| THRAP4 | 17 | q21.1 | 6 | 4.13 | 0.0047 |
| RAPGEFL1 | 17 | q21.1 | 5 | 2.23 | 0.0251 |
| CASC3 | 17 | q21.1 | 5 | 2.70 | 0.0278 |
| RARA | 17 | q21.2 | 3 | 3.58 | 0.0038 |
| CDC6 | 17 | q21.2 | 3 | 1.35 | 0.0065 |
| UBE2Z | 17 | q21.32 | 2 | 1.98 | 0.0111 |
| SNF8 | 17 | q21.32 | 2 | 1.89 | 0.0296 |
| CALCOCO2 | 17 | q21.32 | 2 | 1.83 | 0.0370 |
| EPN3 | 17 | q21.33 | 3 | 3.30 | 0.0005 |
| PHB | 17 | q21.33 | 2 | 2.67 | 0.0111 |
| EME1 | 17 | q21.33 | 2 | 1.47 | 0.0111 |
| SPATA20 | 17 | q21.33 | 3 | 2.89 | 0.0152 |
| SLC35B1 | 17 | q21.33 | 2 | 2.02 | 0.0222 |
| MRPL27 | 17 | q21.33 | 2 | 1.31 | 0.0370 |
| WFIKKN2 | 17 | q21.33 | 2 | 0.91 | 0.0370 |
| LRRC59 | 17 | q21.33 | 2 | 1.39 | 0.0463 |
| C17orf71 | 17 | q22 | 4 | 1.83 | 0.0001 |
| TRIM37 | 17 | q22 | 4 | 2.82 | 0.0001 |
| COIL | 17 | q22 | 4 | 2.15 | 0.0002 |
| FAM33A | 17 | q22 | 4 | 1.76 | 0.0003 |
| SFRS1 | 17 | q22 | 4 | 1.37 | 0.0008 |
| TOM1L1 | 17 | q22 | 5 | 2.61 | 0.0012 |
| SUPT4H1 | 17 | q22 | 3 | 2.06 | 0.0014 |
| RNF43 | 17 | q22 | 3 | 2.90 | 0.0014 |
| PRR11 | 17 | q22 | 4 | 2.05 | 0.0014 |
| RAD51C | 17 | q22 | 3 | 2.59 | 0.0014 |
| STXBP4 | 17 | q22 | 5 | 1.40 | 0.0018 |
| AKAP1 | 17 | q22 | 4 | 1.53 | 0.0058 |
| MTMR4 | 17 | q22 | 4 | 1.79 | 0.0094 |
| TRIM25 | 17 | q22 | 4 | 1.17 | 0.0109 |
| YPEL2 | 17 | q22 | 6 | 1.76 | 0.0119 |
| MKS1 | 17 | q22 | 3 | 1.27 | 0.0126 |
| MRPS23 | 17 | q22 | 3 | 1.84 | 0.0152 |
| GDPD1 | 17 | q22 | 4 | 1.34 | 0.0189 |
| COX11 | 17 | q22 | 5 | 1.31 | 0.0251 |
| AC102948.1 | 17 | q22 | 4 | 0.94 | 0.0305 |
| Sep-04 | 17 | q22 | 4 | 1.47 | 0.0305 |
| OR4D1 | 17 | q22 | 3 | 1.05 | 0.0338 |
| VEZF1 | 17 | q22 | 3 | 1.57 | 0.0387 |
| PTRH2 | 17 | q23.1 | 6 | 2.40 | 0.0000 |
| TMEM49 | 17 | q23.1 | 6 | 1.96 | 0.0001 |
| RPS6KB1 | 17 | q23.1 | 5 | 3.00 | 0.0003 |
| HEATR6 | 17 | q23.1 | 5 | 2.14 | 0.0034 |
| RNFT1 | 17 | q23.1 | 5 | 1.52 | 0.0053 |
| DHX40 | 17 | q23.1 | 6 | 1.40 | 0.0343 |
| USP32 | 17 | q23.1-q23.2 | 5 | 1.50 | 0.0004 |
| METTL2A | 17 | q23.2 | 4 | 2.24 | 0.0000 |
| TLK2 | 17 | q23.2 | 4 | 1.95 | 0.0000 |
| APPBP2 | 17 | q23.2 | 4 | 1.34 | 0.0006 |
| MED13 | 17 | q23.2 | 6 | 1.19 | 0.0016 |
| BCAS3 | 17 | q23.2 | 6 | 1.23 | 0.0047 |
| NACA2 | 17 | q23.2 | 6 | 1.07 | 0.0217 |
| INTS2 | 17 | q23.2 | 6 | 1.82 | 0.0343 |
| C17orf82 | 17 | q23.2 | 6 | 1.12 | 0.0374 |
| DDX42 | 17 | q23.3 | 6 | 1.99 | 0.0000 |
| CYB561 | 17 | q23.3 | 6 | 3.00 | 0.0000 |
| SMARCD2 | 17 | q23.3 | 6 | 2.44 | 0.0000 |
| TEX2 | 17 | q23.3 | 5 | 2.69 | 0.0000 |
| FTSJ3 | 17 | q23.3 | 6 | 2.55 | 0.0000 |
| WDR68 | 17 | q23.3 | 6 | 1.98 | 0.0000 |
| PSMC5 | 17 | q23.3 | 6 | 2.19 | 0.0000 |
| CCDC45 | 17 | q23.3 | 4 | 2.59 | 0.0001 |
| CCDC47 | 17 | q23.3 | 6 | 2.03 | 0.0001 |
| CCDC44 | 17 | q23.3 | 6 | 2.49 | 0.0001 |
| DDX5 | 17 | q23.3 | 4 | 2.44 | 0.0001 |
| POLG2 | 17 | q23.3 | 4 | 2.02 | 0.0002 |
| STRADA | 17 | q23.3 | 6 | 1.82 | 0.0014 |
| MAP3K3 | 17 | q23.3 | 6 | 1.34 | 0.0021 |
| ERN1 | 17 | q23.3 | 6 | 2.07 | 0.0076 |
| CSH2 | 17 | q23.3 | 6 | 0.93 | 0.0343 |
| AC113554.3 | 17 | q23.3 | 6 | 0.94 | 0.0343 |
| GNA13 | 17 | q24.1 | 2 | 3.27 | 0.0019 |
| AC132812.9 | 17 | q24.1 | 2 | 3.07 | 0.0037 |
| KPNA2 | 17 | q24.2 | 3 | 3.30 | 0.0001 |
| PRKAR1A | 17 | q24.2 | 3 | 3.30 | 0.0001 |
| NOL11 | 17 | q24.2 | 3 | 2.27 | 0.0002 |
| HELZ | 17 | q24.2 | 2 | 2.13 | 0.0019 |
| C17orf58 | 17 | q24.2 | 3 | 1.61 | 0.0020 |
| SLC16A6 | 17 | q24.2 | 3 | 2.91 | 0.0038 |
| ARSG | 17 | q24.2 | 3 | 1.70 | 0.0083 |
| CACNG4 | 17 | q24.2 | 2 | 4.95 | 0.0167 |
| CACNG1 | 17 | q24.2 | 2 | 1.97 | 0.0222 |
| FALZ | 17 | q24.2 | 3 | 1.47 | 0.0292 |
| SEH1L | 18 | p11.21 | 2 | 1.59 | 0.0370 |
| ZNF217 | 20 | q13.2 | 2 | 2.66 | 0.0167 |
| RAB22A | 20 | q13.32 | 2 | 2.06 | 0.0037 |

**Table S6. List of regions and genes whose copy number (cbs-smoothed ratio) is positively (6A) or negatively (6B) correlated with the decrease in Ki67 labelling index after 2 weeks of AI therapy.**

**Table S6A**

| **Chromosome** | **Cytobands** | **Start** | **End** | **BACs** | **Length MB** | **Cases Gain** | **Cases Loss** | **Cases Amp** | **Spearman Rho** | **Genes** |
| --- | --- | --- | --- | --- | --- | --- | --- | --- | --- | --- |
| 2 | q11.1 | 95377432 | 95652499 | 3 | 0.28 | 7 | 8 |  | 0.34 | TEKT4 |
| 3 | p14.1-p12.3 | 68366963 | 74369634 | 52 | 6.00 | 4 | 18 |  | 0.36 | FAM19A1, FAM19A4, C3orf64, TMF1, UBA3, ARL6IP5, LMOD3, FRMD4B, MITF, FOXP1, EIF4E3, GPR27, PROK2, RYBP, SHQ1, GLT8D4, PPP4R2, PDZRN3, CNTN3 |
| 3 | p14.2 | 59424726 | 60304452 | 10 | 0.88 | 6 | 14 |  | 0.36 | FHIT, NPCDR1 |
| 3 | p14.2-p14.1 | 60767725 | 67288774 | 54 | 6.52 | 6 | 14 |  | 0.36 | PTPRG, ID2B, C3orf14, FEZF2, CADPS, SYNPR, SNTN, C3orf49, THOC7, ATXN7, PSMD6, PRICKLE2, ADAMTS9, SLC25A26, LRIG1, KBTBD8 |
| 3 | p14.3 | 55698308 | 56234603 | 3 | 0.54 | 2 | 16 |  | 0.32 | ERC2 |
| 3 | p14.3-p14.2 | 56522233 | 58836651 | 17 | 2.31 | 5 | 15 |  | 0.35 | CCDC66, C3orf63, ARHGEF3, SPATA12, IL17RD, HESX1, APPL1, DNAH12, PDE12, ARF4, FAM116A, SLMAP, FLNB, DNASE1L3, ABHD6, RPP14, PXK, PDHB, KCTD6, ACOX2, FAM107A, FAM3D, C3orf67 |
| 3 | p21.1 | 52697081 | 53459548 | 10 | 0.76 | 1 | 16 |  | 0.31 | PBRM1, GNL3, GLT8D1, SPCS1, NEK4, ITIH1, ITIH3, ITIH4, TMEM110, SFMBT1, RFT1, PRKCD, TKT, DCP1A |
| 3 | p21.31 | 46277063 | 46569369 | 3 | 0.29 | 1 | 17 |  | 0.33 | CCR3, CCR2, CCR5, CCRL2, LTF, RTP3, LRRC2 |
| 3 | p21.31-p21.2 | 46562202 | 52031178 | 60 | 5.47 | 3 | 23 |  | 0.35 | LRRC2, LUZPP1, TDGF1, ALS2CL, TMIE, MYL3, PTH1R, CCDC12, NBEAL2, SETD2, KIF9, KLHL18, PTPN23, SCAP, C3orf75, CSPG5, SMARCC1, DHX30, MAP4, CDC25A, CAMP, ZNF589, NME6, SPINK8, FBXW12, PLXNB1, CCDC51, CCDC72, ATRIP, TREX1, SHISA5, PFKFB4, UCN2, COL7A1, UQCRC1, TMEM89, SLC26A6, CELSR3, NCKIPSD, IP6K2, PRKAR2A, SLC25A20, C3orf71, ARIH2, P4HTM, WDR6, DALRD3, NDUFAF3, IMPDH2, QRICH1, QARS, USP19, LAMB2, CCDC71, KLHDC8B, CCDC36, C3orf62, USP4, GPX1, RHOA, TCTA, AMT, NICN1, DAG1, BSN, APEH, MST1, RNF123, AMIGO3, GMPPB, IP6K1, CDH29, C3orf54, UBA7, TRAIP, CAMKV, MST1R, MON1A, RBM6, RBM5, SEMA3F, GNAT1, GNAI2, SEMA3B, C3orf45, IFRD2, NAT6, HYAL1, HYAL2, TUSC2, RASSF1, ZMYND10, TUSC4, CYB561D2, TMEM115, CACNA2D2, C3orf18, HEMK1, CISH, MAPKAPK3, DOCK3, ARMET, RBM15B, VPRBP, RAD54L2, TEX264, GRM2, IQCF6, IQCF3, IQCF2, IQCF5, IQCF1, RRP9, PARP3, GPR62, PCBP4, ABHD14B, ACY1, RPL29 |
| 3 | p22.1 | 42827281 | 43459898 | 10 | 0.63 | 2 | 11 |  | 0.32 | CCDC13, CCBP2, CYP8B1, ZNF662, C3orf39, SNRK, ANO10 |
| 4 | p15.2 | 22437451 | 23334492 | 8 | 0.90 | 6 | 9 |  | 0.31 | GPR125 |
| 4 | q12 | 57401111 | 58082758 | 6 | 0.68 | 7 | 6 |  | 0.32 | HOPX, SPINK2, REST, C4orf14, POLR2B, IGFBP7 |
| 6 | q27 | 168272746 | 168761427 | 4 | 0.49 | 5 | 9 |  | 0.32 | MLLT4, C6orf54, KIF25, FRMD1, DACT2 |
| 7 | p11.2-p11.1 | 56498410 | 57769424 | 20 | 1.27 | 6 | 4 |  | 0.37 | ZNF479, ZNF716 |
| 7 | q11.21 | 62487075 | 65016125 | 43 | 2.53 | 11 | 6 |  | 0.35 | ZNF679, ZNF680, ZNF107, ZNF138, ZNF273, ZNF117, ERV3, ZNF92 |
| 9 | p11.1-q11 | 39090138 | 40771683 | 9 | 1.68 | 10 | 16 |  | 0.40 | CNTNAP3, FAM75A1, ZNF658B, FAM75A2, FAM74A1, FAM75A3, FAM74A3, ZNF658 |
| 9 | q11-q21.11 | 42925808 | 66604141 | 26 | 23.68 | 12 | 15 |  | 0.39 | ANKRD20A1, FAM75A6, CNTNAP3B, FAM27C, FAM27A, FAM27E2, FAM27E1, FAM27D1, FAM75A7 |
| 10 | q23.1 | 82902084 | 85188135 | 23 | 2.29 | 5 | 9 |  | 0.32 | NRG3 |
| 11 | p15.3-p15.1 | 10732673 | 17177533 | 65 | 6.44 | 9 | 17 | 2 | 0.38 | CTR9, EIF4G2, ZBED5, GALNTL4, USP47, DKK3, MICAL2, MICALCL, PARVA, TEAD1, RASSF10, ARNTL, BTBD10, PTH, FAR1, SPON1, RRAS2, COPB1, PSMA1, PDE3B, CYP2R1, CALCB, CALCA, INSC, SOX6, C11orf58, PLEKHA7, RPS13, PIK3C2A |
| 11 | p15.4 | 3524947 | 7993105 | 41 | 4.47 | 6 | 20 | 1 | 0.34 | ART5, ART1, CHRNA10, NUP98, RHOG, STIM1, RRM1, OR52B4, TRIM21, OR52K2, OR52K3P, OR52K1, OR52M1, C11orf40, OR52I2, OR52I1, TRIM68, OR51D1, OR51E1, OR51E2, OR51C1P, MMP26, OR51F1, OR52R1, OR51F2, OR51S1, OR51H1P, OR51T1, OR51A6P, OR51A7, OR51G2, OR51G1, OR51A4, OR51A2, OR51L1, OR52J3, OR52E2, OR52A4, OR52A5, OR52A1, OR51V1, HBB, HBG1, HBG2, HBE1, OR51B4, OR51B5, OR51B2, OR51B6, OR51M1, OR51J1, OR51Q1, OR51I1, OR51I2, OR52D1, UBQLN3, UBQLNL, OR52H1, OR52B6, TRIM34, TRIM5, TRIM22, OR56B1, OR52N4, OR52N5, OR52N1, OR52N2, OR52E6, OR52E8, OR52E4, OR52E5, OR56A3, OR52L1, OR56A4, OR56A1, OR52L2P, OR56B4, OR52W1, C11orf42, FAM160A2, CNGA4, CCKBR, PRKCDBP, SMPD1, APBB1, HPX, TRIM3, ARFIP2, FXC1, DNHD1, RRP8, ILK, TAF10, TPP1, DCHS1, MRPL17, OR2AG2, OR2AG1, OR6A2, OR10A5, OR10A2, OR10A4, OR2D2, OR2D3, ZNF215, ZNF214, NLRP14, RBMXL2, SYT9, OLFML1, PPFIBP2, CYB5R2, OVCH2, OR10AB1P, OR5P2, OR5P3, OR10A6, OR10A3, NLRP10 |
| 11 | p15.4 | 8902428 | 9468291 | 6 | 0.57 | 5 | 18 |  | 0.35 | ST5, C11orf17, C11orf16, ASCL3, TMEM9B, NRIP3, SCUBE2, DENND5A, TMEM41B, IPO7 |
| 11 | p15.5 | 727631 | 1520440 | 5 | 0.79 | 4 | 17 |  | 0.36 | EPS8L2, TALDO1, PDDC1, CEND1, SLC25A22, LRDD, RPLP2, PNPLA2, EFCAB4A, CD151, POLR2L, TSPAN4, CHID1, AP2A2, MUC6, MUC2, MUC5B, TOLLIP, BRSK2 |
| 12 | q13.11 | 48085728 | 48427284 | 3 | 0.34 | 8 | 6 |  | 0.33 | RPAP3, RAPGEF3, SLC48A1, HDAC7, VDR, TMEM106C, COL2A1 |
| 12 | q13.11 | 48395289 | 48867224 | 4 | 0.47 | 8 | 3 |  | 0.34 | COL2A1, SENP1, PFKM, ASB8, C12orf68, OR10AD1, H1FNT, ZNF641, ANP32D |
| 12 | q13.13 | 52051871 | 52699966 | 8 | 0.65 | 3 | 8 |  | 0.36 | SCN8A, FIGNL2, ANKRD33, ACVRL1, ACVR1B, GRASP, NR4A1, C12orf44, KRT80, KRT7, KRT81, KRT86 |
| 12 | q13.13-q13.2 | 54410626 | 55084402 | 9 | 0.67 | 2 | 11 |  | 0.34 | HOXC4, HOXC6, HOXC5, SMUG1, CBX5, HNRNPA1, NFE2, COPZ1, GPR84, ZNF385A, ITGA5, GTSF1, NCKAP1L, PDE1B, PPP1R1A, GLYCAM1, LACRT, DCD |
| 12 | q13.2 | 55736347 | 56007952 | 4 | 0.27 | 12 | 2 |  | 0.33 | LACRT, DCD, MUCL1 |
| 12 | q13.2 | 55021225 | 55267324 | 2 | 0.25 | 5 | 6 |  | 0.33 | OR6C75, OR6C65, PHC1B, OR6C76, OR6C2, OR6C70, OR6C68, OR6C4, OR2AP1 |
| 16 | p13.3 | 1146652 | 2567229 | 15 | 1.42 | 12 | 6 | 1 | 0.32 | CACNA1H, TPSG1, TPSB2, TPSAB1, TPSD1, PRSS29P, UBE2I, BAIAP3, C16orf42, GNPTG, UNKL, C16orf91, CCDC154, CLCN7, C16orf38, TELO2, IFT140, TMEM204, CRAMP1L, HN1L, MAPK8IP3, NME3, MRPS34, EME2, SPSB3, NUBP2, IGFALS, HAGH, FAHD1, C16orf73, HS3ST6, SEPX1, RPL3L, NDUFB10, RPS2, RNF151, TBL3, NOXO1, GFER, SYNGR3, ZNF598, NPW, NTHL1, TSC2, PKD1, RAB26, TRAF7, CASKIN1, C16orf79, PGP, E4F1, DNASE1L2, DCI, RNPS1, ABCA3, ABCA17P, CCNF, C16orf59, NTN3, TBC1D24, ATP6V0C |
| 17 | q21.2-q21.31 | 40437784 | 41248190 | 12 | 0.81 | 2 | 26 |  | 0.34 | STAT5A, STAT3, PTRF, ATP6V0A1, NAGLU, HSD17BP1, HSD17B1, COASY, MLX, PSMC3IP, FAM134C, TUBG1, TUBG2, PLEKHH3, CCR10, CNTNAP1, EZH1, RAMP2, VPS25, WNK4, CCDC56, CNTD1, BECN1, PSME3, AOC2, AOC3, G6PC, AARSD1, RUNDC1, RPL27, IFI35, VAT1, RND2, BRCA1 |
| 17 | q21.31 | 43673995 | 43987259 | 5 | 0.31 | 5 | 24 |  | 0.33 | DHX8, ETV4, MEOX1, SOST, DUSP3, MPP3, CD300LG, MPP2, C17orf88, PPY, PYY, NAGS, TMEM101, LSM12, G6PC3, HDAC5, C17orf53, ASB16, C17orf65, TMUB2, ATXN7L3, UBTF, SLC4A1, RUNDC3A, SLC25A39, GRN, FAM171A2, ITGA2B, GPATCH8, FZD2, CCDC43, DBF4B, ADAM11, GJC1, HIGD1B, EFTUD2, CCDC103, FAM187A, GFAP, KIF18B, C1QL1, DCAKD, NMT1, PLCD3, ACBD4, HEXIM1, HEXIM2, FMNL1, C17orf46, MAP3K14, SH3D20, PLEKHM1 |
| 17 | q21.31 | 41514509 | 43584577 | 23 | 2.07 | 2 | 25 |  | 0.35 | C17orf69, CRHR1, MAPT |
| 17 | q21.31-q21.32 | 44595516 | 45203537 | 6 | 0.61 | 12 | 19 | 1 | 0.34 | LRRC37A2, ARL17P1, NSF, WNT3, WNT9B, GOSR2, RPRML, LRRC37A4, CDC27 |
| 17 | q21.32-q21.33 | 47165185 | 47489001 | 2 | 0.32 | 7 | 20 | 1 | 0.35 | B4GALNT2, GNGT2, ABI3, PHOSPHO1, ZNF652, PHB |
| 17 | q21.33 | 48595241 | 48801049 | 2 | 0.21 | 9 | 17 | 2 | 0.34 | MYCBPAP, EPN3, SPATA20, CACNA1G, ABCC3, ANKRD40 |
| 23 | p21.1 | 33153976 | 34098692 | 10 | 0.94 | 7 | 8 |  | 0.33 | DMD |
| 23 | p21.3-p21.1 | 28034585 | 32189933 | 44 | 4.16 | 6 | 9 |  | 0.35 | IL1RAPL1, MAGEB2, MAGEB3, MAGEB4, MAGEB1, NR0B1, CXorf21, GK, MAP3K7IP3, CXorf29, FTHL17, DMD |
| 23 | q11.1 | 57452750 | 58269325 | 8 | 0.82 | 9 |  |  | 0.32 | FAAH2, ZXDB, ZXDA |
| 23 | q11.1-q11.2 | 62252192 | 63374832 | 9 | 1.12 | 8 | 2 |  | 0.31 | SPIN4, ARHGEF9 |
| 23 | q11.2-q12 | 63991799 | 64666488 | 10 | 0.67 | 7 | 2 |  | 0.31 | ZC4H2 |
| 23 | q12 | 65516329 | 67240867 | 19 | 1.72 | 9 | 4 |  | 0.37 | EDA2R, AR |

**Table S6B**

| **Chromosome** | **Cytobands** | **Start** | **End** | **BACs** | **Length MB** | **Cases Gain** | **Cases Loss** | **Cases Amp** | **Spearman Rho** | **Genes** |
| --- | --- | --- | --- | --- | --- | --- | --- | --- | --- | --- |
| 2 | q14.1-q14.2 | 118688910 | 119042691 | 2 | 0.35 | 4 | 10 |  | -0.33 | CCDC93, INSIG2 |
| 2 | q31.1 | 175334234 | 175746377 | 5 | 0.41 | 4 | 7 |  | -0.32 | GPR155, WIPF1, CHRNA1, CHN1 |
| 7 | q22.1 | 100873507 | 101376032 | 5 | 0.50 |  | 24 |  | -0.32 | CLDN15, FIS1, RABL5, EMID2, MYL10 |
| 11 | q13.2-q13.3 | 67755774 | 68473611 | 6 | 0.72 | 14 | 16 | 7 | -0.36 | UNC93B1, ALDH3B1, NDUFS8, TCIRG1, CHKA, SUV420H1, C11orf24, LRP5, SAPS3, GAL |
| 12 | p12.2-p12.1 | 20402713 | 22204685 | 18 | 1.80 | 14 | 8 | 1 | -0.33 | PDE3A, SLCO1C1, SLCO1B3, SLCO1B1, SLCO1A2, IAPP, PYROXD1, RECQL, GOLT1B, C12orf39, GYS2, LDHB, KCNJ8, ABCC9, CMAS |
| 12 | p13.1-p12.3 | 14409888 | 16633839 | 20 | 2.22 | 13 | 8 |  | -0.35 | ATF7IP, PLBD1, GUCY2C, HIST4H4, H2AFJ, WBP11, C12orf60, C12orf69, ART4, MGP, ERP27, ARHGDIB, PDE6H, RERG, PTPRO, EPS8, STRAP, DERA, SLC15A5, MGST1 |
| 12 | p13.33 | 1254108 | 2206260 | 9 | 0.95 | 6 | 11 |  | -0.40 | ERC1, FBXL14, WNT5B, ADIPOR2, LRTM2, DCP1B, CACNA1C |

**REFERENCES**

1. Natrajan R, Weigelt B, Mackay A, Geyer FC, Grigoriadis A, Tan DS, Jones C, Lord CJ, Vatcheva R, Rodriguez-Pinilla SM *et al*: **An integrative genomic and transcriptomic analysis reveals molecular pathways and networks regulated by copy number aberrations in basal-like, HER2 and luminal cancers**. *Breast Cancer Res Treat* 2010, **121**(3):575-589.

2. Mackay A, Tamber N, Fenwick K, Iravani M, Grigoriadis A, Dexter T, Lord CJ, Reis-Filho JS, Ashworth A: **A high-resolution integrated analysis of genetic and expression profiles of breast cancer cell lines**. *Breast Cancer Res Treat* 2009, **118**(3):481-498.

3. Natrajan R, Lambros MB, Rodriguez-Pinilla SM, Moreno-Bueno G, Tan DS, Marchio C, Vatcheva R, Rayter S, Mahler-Araujo B, Fulford LG *et al*: **Tiling path genomic profiling of grade 3 invasive ductal breast cancers**. *Clin Cancer Res* 2009, **15**(8):2711-2722.

4. Reis-Filho JS, Drury S, Lambros MB, Marchio C, Johnson N, Natrajan R, Salter J, Levey P, Fletcher O, Peto J *et al*: **ESR1 gene amplification in breast cancer: a common phenomenon?** *Nat Genet* 2008, **40**(7):809-810; author reply 810-802.

5. Geyer FC, Weigelt B, Natrajan R, Lambros MB, de Biase D, Vatcheva R, Savage K, Mackay A, Ashworth A, Reis-Filho JS: **Molecular analysis reveals a genetic basis for the phenotypic diversity of metaplastic breast carcinomas**. *J Pathol* 2010, **220**(5):562-573.

6. Marchio C, Iravani M, Natrajan R, Lambros MB, Savage K, Tamber N, Fenwick K, Mackay A, Senetta R, Di Palma S *et al*: **Genomic and immunophenotypical characterization of pure micropapillary carcinomas of the breast**. *J Pathol* 2008, **215**(4):398-410.

7. Marchio C, Natrajan R, Shiu KK, Lambros MB, Rodriguez-Pinilla SM, Tan DS, Lord CJ, Hungermann D, Fenwick K, Tamber N *et al*: **The genomic profile of HER2-amplified breast cancers: the influence of ER status**. *J Pathol* 2008, **216**(4):399-407.

8. Lacroix-Triki M, Suarez PH, MacKay A, Lambros MB, Natrajan R, Savage K, Geyer FC, Weigelt B, Ashworth A, Reis-Filho JS: **Mucinous carcinoma of the breast is genomically distinct from invasive ductal carcinomas of no special type**. *J Pathol* 2010, **222**(3):282-298.

9. Hicks J, Krasnitz A, Lakshmi B, Navin NE, Riggs M, Leibu E, Esposito D, Alexander J, Troge J, Grubor V *et al*: **Novel patterns of genome rearrangement and their association with survival in breast cancer**. *Genome research* 2006, **16**(12):1465-1479.
