# Supplementary figures and images for: Integrative analyses identify modulators of response to neoadjuvant aromatase inhibitors in patients with early breast cancer
Source: Breast Cancer Res. 2015 Mar 11;17(1):35. doi: 10.1186/s13058-015-0532-0 (PMC4406016; doi:10.1186/s13058-015-0532-0)

Figure S2

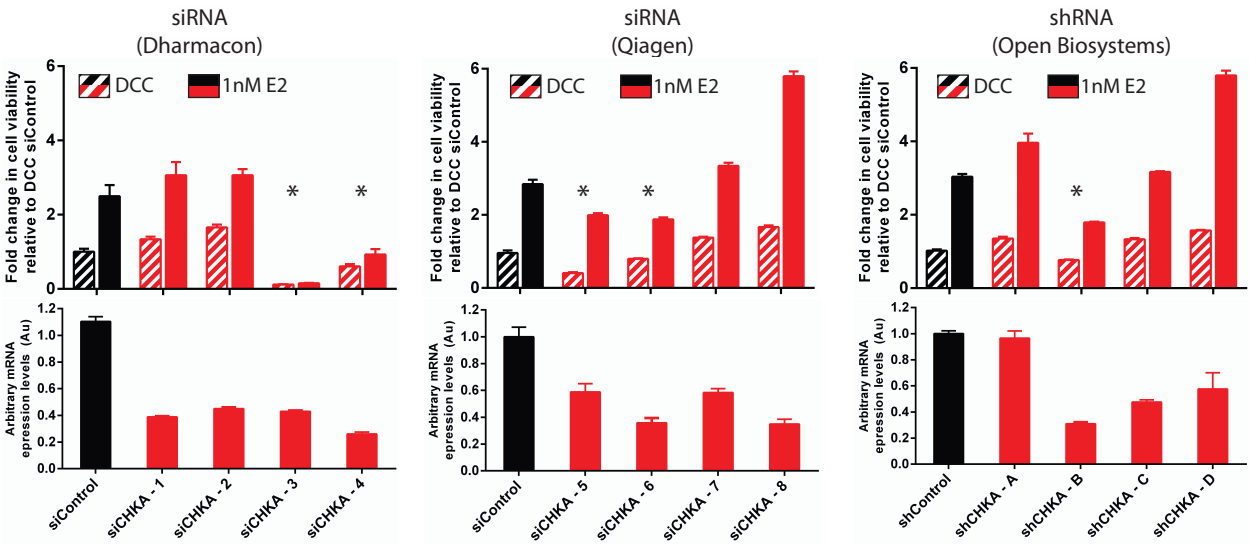

Supplement: Supplementary file 3 — Deconvolution of CHKA siRNA and shRNA pools. SUM44 cells were transfected with Individual siRNA and shRNA from various manufacturers and the impact on proliferation measured after 6 days. CHKA knockdown was confirmed in each case by quantitative real-time PCR. [file 13058_2015_532_MOESM3_ESM.pdf]
