# Supplementary material for: Integrative analyses identify modulators of response to neoadjuvant aromatase inhibitors in patients with early breast cancer
Source: Breast Cancer Res. 2015 Mar 11;17(1):35. doi: 10.1186/s13058-015-0532-0 (PMC4406016; doi:10.1186/s13058-015-0532-0)
Supplement: Supplementary file 4 — Copy number analysis of SUM44, MDA-MB134-VI, T47D, MCF7 and long-term estrogen deprived (LTED) by quantitative real-time PCR (qRT-PCR) (A) and transcript levels of CHKA in the target cell lines used for functional analysis measured by qRT-PCR (B). [file 13058_2015_532_MOESM4_ESM.pptx]

## Slide 1
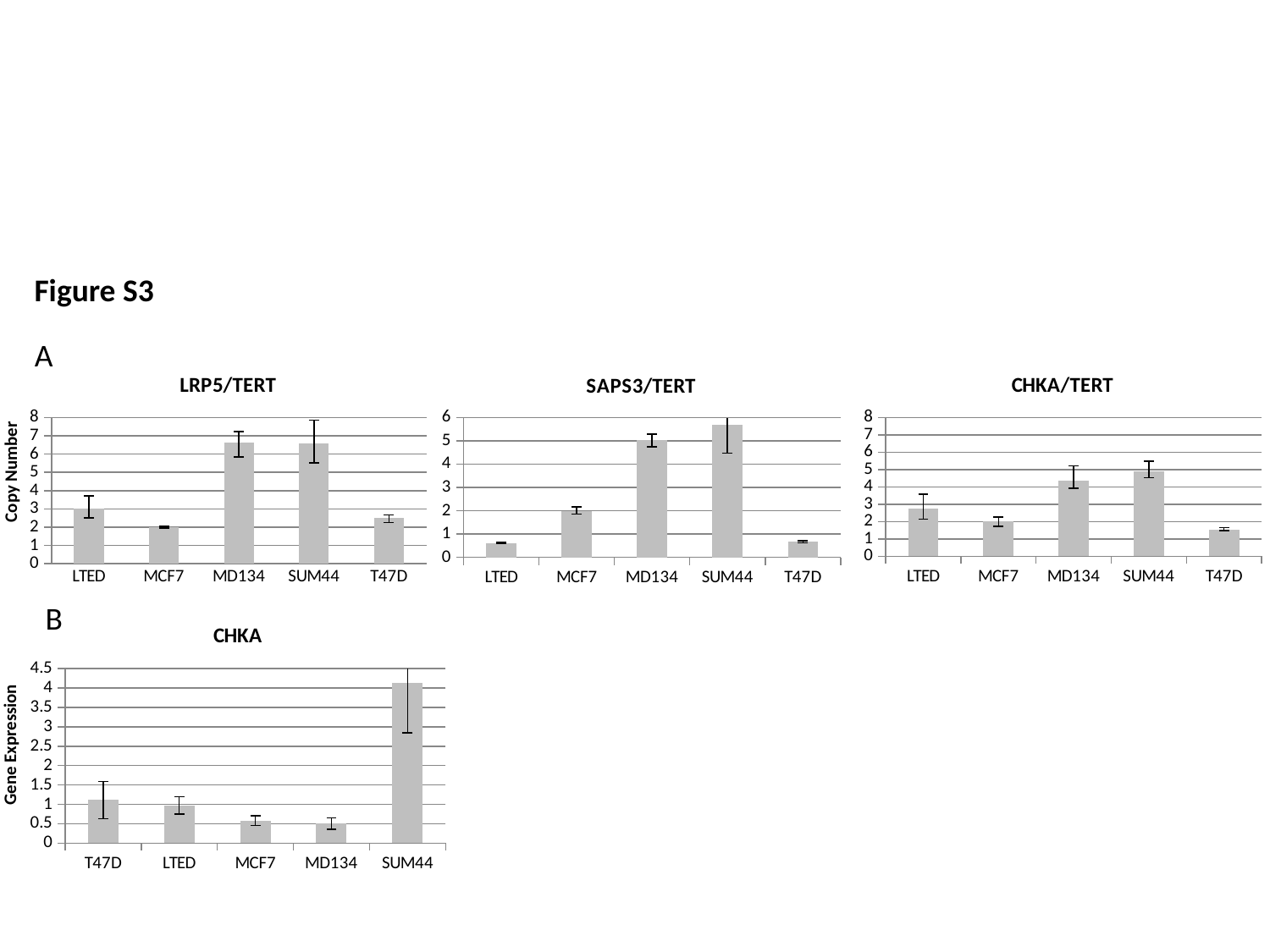

Figure S3
A
### Chart:
| Category | LRP5/TERT |
|---|---|
| LTED | 3.03 |
| MCF7 | 2.0 |
| MD134 | 6.619999999999999 |
| SUM44 | 6.59 |
| T47D | 2.49 |
### Chart:
| Category | SAPS3/TERT |
|---|---|
| LTED | 0.61 |
| MCF7 | 2.0 |
| MD134 | 5.05 |
| SUM44 | 5.68 |
| T47D | 0.67 |
### Chart:
| Category | CHKA/TERT |
|---|---|
| LTED | 2.76 |
| MCF7 | 2.0 |
| MD134 | 4.38 |
| SUM44 | 4.88 |
| T47D | 1.53 |Copy Number
B
### Chart:
| Category | CHKA |
|---|---|
| T47D | 1.1092125297576607 |
| LTED | 0.9750351816029893 |
| MCF7 | 0.5826578242107704 |
| MD134 | 0.5044455626245272 |
| SUM44 | 4.132373899150201 |Gene Expression
